# Halogen-directed chemical sialylation: pseudo-stereodivergent access to marine ganglioside epitopes†‡

**DOI:** 10.1039/d0sc01219j

**Published:** 2020-03-26

**Authors:** Taiki Hayashi, Alexander Axer, Gerald Kehr, Klaus Bergander, Ryan Gilmour

**Affiliations:** Organisch-Chemisches Institut, Westfälische Wilhelms-Universität Münster Corrensstrasse 40 Münster Germany ryan.gilmour@uni-muenster.de

## Abstract

Sialic acids are conspicuous structural components of the complex gangliosides that regulate cellular processes. Their importance in molecular recognition manifests itself in drug design (*e.g.* Tamiflu®) and continues to stimulate the development of effective chemical sialylation strategies to complement chemoenzymatic technologies. Stereodivergent approaches that enable the α- or β-anomer to be generated at will are particularly powerful to attenuate hydrogen bond networks and interrogate function. Herein, we demonstrate that site-selective halogenation (F and Br) at C3 of the *N*-glycolyl units common to marine Neu2,6Glu epitopes enables pseudo-stereodivergent sialylation. α-Selective sialylation results from fluorination, whereas traceless bromine-guided sialylation generates the β-adduct. This concept is validated in the synthesis of HLG-1 and Hp-s1 analogues.

## Abstract 2

Sialic acids are conspicuous structural components of the complex gangliosides that regulate cellular processes.

## Graphical abstract

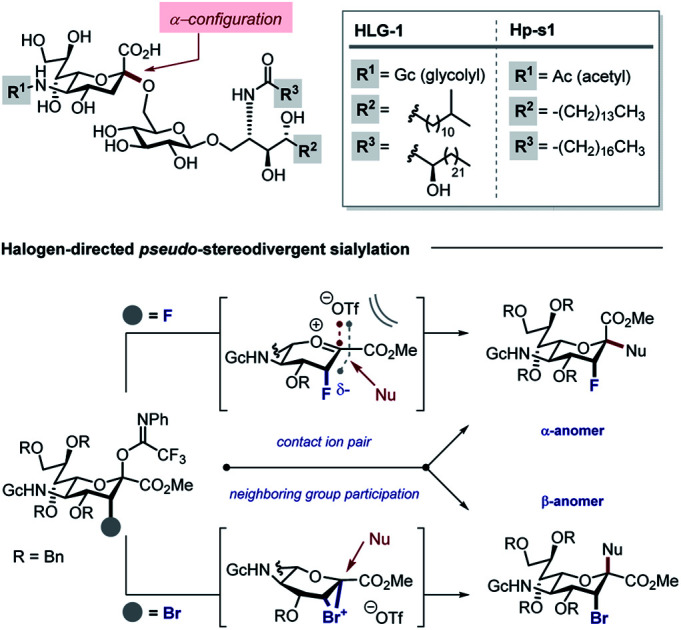

Gangliosides are pervasive in terrestrial and marine organisms where their structural heterogeneity manifests itself in the regulation of a broad spectrum of cellular processes.^1^ These intriguing natural products are conspicuous on account of their amphiphilic nature, where the simplicity of the lipid anchor contrasts sharply with the increasing stereochemical complexity of the carbohydrate epitope. Whilst the core of the epitope consists of common monosaccharides, the periphery is functionalised with sialic acids units that may vary in their N-substitution.^2^ The topology of the epitope encodes for a precise hydrogen bond network that underpins function,^3^ and thus the stereocontrolled construction of gangliosides is pivotal in delineating structure–function interplay across the glycosciences.^4^ The ubiquity and exterior position of sialic acid units in bioactive gangliosides therefore requires that efficient and selective (α or β) chemical sialylation strategies be devised to complement chemoenzymatic approaches.^5^

Gangliosides derived from marine echinoderms are particularly attractive targets on account of their complexity and importance in chemical neurology.^6,7^ Our interest in modulating non-neuronal glial cell behaviour with fluorinated carbohydrates^8^ led us to identify the Neu2,6Glu fragment as a promising target for neuropathy.^9^ This structural feature is common to a plenum of bioactive gangliosides including HLG-1, isolated from the sea cucumber *Holothuria leucospilota*,^6^ and Hp-s1, isolated from sea urchin *Hemicentrotus pulcherrimus*^7*a*,*b*^ and *Diadema setosum*^7*c*^ (Fig. 1, top).

**Fig. 1:**
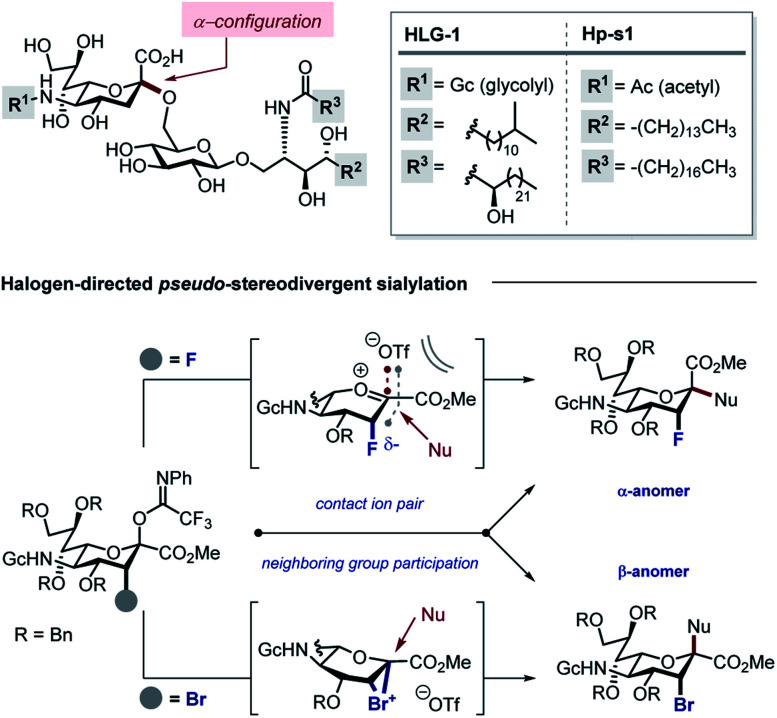
Top: Naturally occurring gangliosides HLG-1 and Hp-s1. Bottom: Regulating the stereochemical outcome of sialylation by halogen introduction at the C3 position.

In developing a pseudo-stereodivergent platform to access the Neu2,6Glu epitope, the concept of halogen-dependent sialylation was investigated (Fig. 1, bottom). It was envisaged that halogen installation at C3 (ref. 10) would provide a steering group to direct the stereochemical course of sialylation (α or β) and offer the possibility to simultaneously modulate the physicochemical profile of the product. This strategy was motivated by the observation that fluorination of the GM4 epitope upregulates oligodendrocyte differentiation,^8^ and that this process of molecular editing can direct sialylation in the *N*-acetyl derivatives common to terrestrial gangliosides. As a working hypothesis, it was reasoned that sialylation with the fluorinated *N*-Gc derivative would proceed *via* a contact ion pair with the counterion distal from the F^*δ*−^ to minimize electrostatic repulsion.^11,12^ This strategy to access the α-anomer with the axial C(sp^3^)–F bond would also circumvent competing elimination that is often observed in the parent scaffolds. In contrast, C3 bromination would allow anchimeric assistance to be invoked to favour formation of the β-anomer.^13^ Moreover, the axial-bromo substituent would mitigate elimination but also provide the opportunity to devise a traceless protocol by virtue of a subsequent reduction step. To validate this pseudo-stereodivergent synthesis of the Neu2,6Glu unit, the fluorinated and brominated analogues of the HLG-1 epitope were prepared from the N–Ac glycal (Scheme 1). Mild and efficient conversion of the commercially available N–Ac derivative to the requisite to *N*-Gc (Gc = glycolyl) derivative was achieved through conversion of the amide to the carbamate as reported by Burk *et al.*^14^ and applied to sialic acid chemistry by Izumi *et al.*^15^ Initial Boc protection of glycal **1** (Boc_2_O, DMAP, in THF, reflux) followed by methanolysis of the acetyl group afforded *N*-Boc methyl ester **3** (24%) along with recovered carboxylic acid **2** (73%), which could be reprocessed to **3** by methylation with MeI and K_2_CO_3_. Exposure of **3** to a TMSCl–phenol combination^16^ effectively cleaved the *N*-Boc group to generate the free amine: this was processed further with treatment with benzyloxyacetyl chloride {(Bn)GcCl} in the presence of NEt_3_ to afford *N*-Gc glycal **4**. Hydroxyfluorination of **4** was carried out with Selectfluor® in wet DMF. The diastereoisomers were easily separated by column chromatography to give the axial-F **5** (61%) and equatorial-F **6** (19%) donors. Installation of the C3 bromo group proved facile by exposing **4** to NBS in wet DMF. The diastereomers axial-Br **7** and equatorial-Br **8** were isolated in 58 and 32% yield, respectively. Given the lower levels of selectivity observed with equatorial-F donors in N–Ac systems,^11*d*^ compound **6** was not investigated further and the remainder of the synthesis was completed with the axial derivative **5**.

**Scheme 1:**
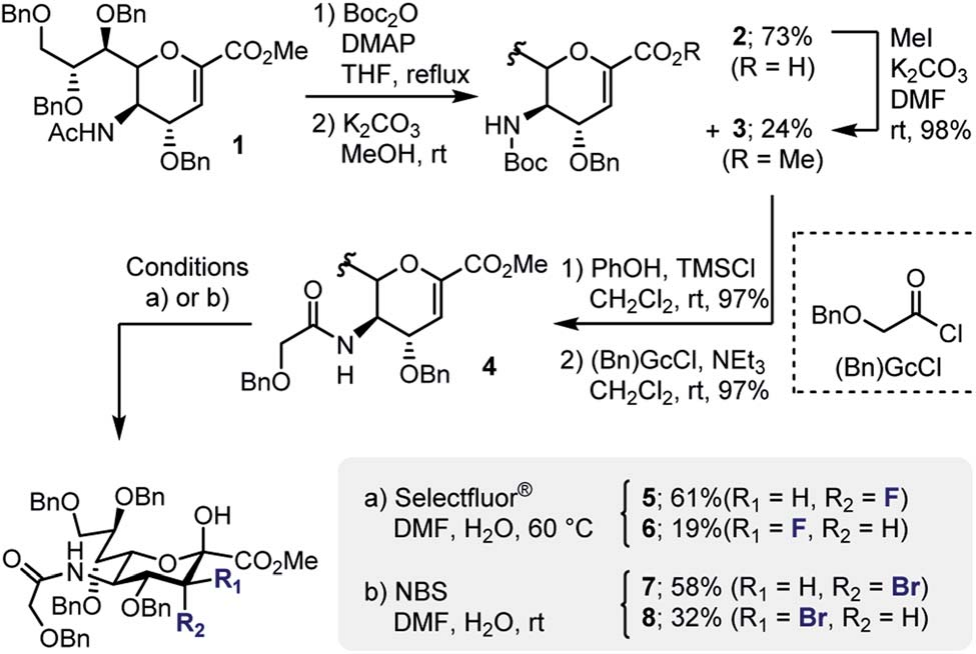
Site selective halogenation of the sialyl donors.

Prior to imidate formation, the anomeric configuration of the sialoside donors was defined based on the ^3^*J*(^13^C1–C2–C3–^19^F_ax/eq_) and/or ^3^*J*(^13^C1–C2–C3–^1^H_ax/eq_) coupling constant from the ^13^C NMR. Given the coupling constant (^3^*J*_CF(H)_) dependence on torsion angle, values of *ca.* 3.7 and *ca.* 5.5 Hz were expected for the α-configuration, reflecting the antiperiplanar arrangements of ^13^C1 and ^19^F or ^1^H, respectively, at C3. Small values around 1 Hz (perhaps not even resolved in the NMR spectrum) should result from the *syn*- and *anti*-clinal relationships of ^13^C and ^19^F/^1^H in both the α- and in the β-configuration.^10*a*,*c*,17^ To that end, selective decoupling experiments were carried out in order to determine the requisite coupling constants. Fig. 2 demonstrates two representative examples, one for compound **6** and the other for compound **8** thereby establishing the β-configuration indicated. In both cases the carbonyl C1(sp^2^)-atom of the ester substituent was investigated. Standard broadband ^1^H decoupled (CPD) ^13^C experiments directly revealed the ^3^*J*_FC_ (1.1 Hz) coupling constant of compound **6**. Determination of the respective ^3^*J*_CH_ coupling constant (1.5 Hz) was achieved by simultaneous selective decoupling of ^19^F and the ^1^H resonance of the OMe group. For compound **8**, the ^3^*J*_CH_ coupling constant was not resolved by a selective ^1^H decoupling ^13^C NMR experiment, but its value could be estimated to be less than 1 Hz due to the line width. These data indicate that C3 halogenated donors favour the β-configuration, likely due to an enhanced anomeric effect.

**Fig. 2:**
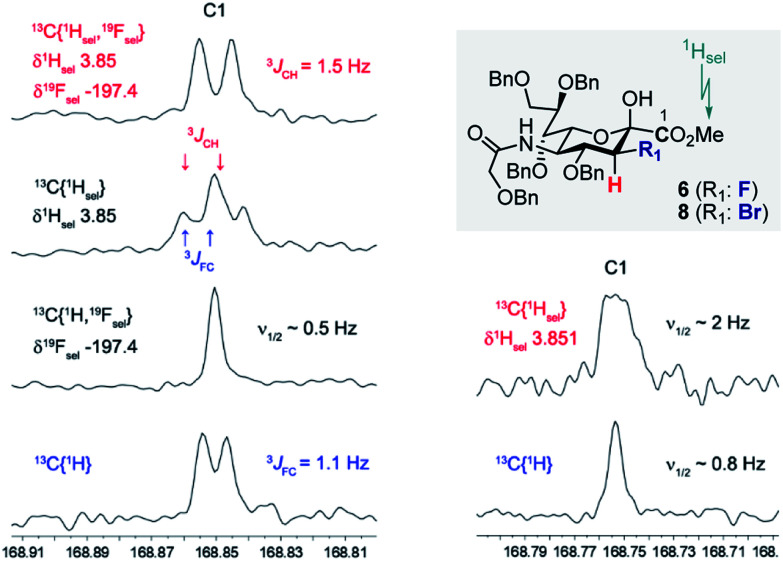
Right: Decoupling experiments carried out on compound **6** to determine ^3^*J*_CH_ and ^3^*J*_FC_. Left: Decoupling experiments carried out on compound **8** to determine ^3^*J*_CH_. In both examples, the OMe group of the ester was selectively decoupled (^1^H_sel_).

Attempted generation of the imidate derivative of **7** (ax-Br) under standard conditions (2,2,2-trifloroacetimidoyl chloride, K_2_CO_3_, acetone, rt),^18^ proved to be problematic, resulting in the formation of epoxide **12** as the major product (Scheme 2). This is consistent with intramolecular cyclisation *via* the anomeric alkoxide generated by exposure to K_2_CO_3_ (**A**). However, through a process of optimisation it was possible to generate donor **9** exclusively (86%) and these conditions were extended to generate **10** (71%, from **8**). In the case of the fluorinated substrate **5**, imidate **11** was generated under standard conditions described above (93%). In all cases, β-anomers were generated as established by NMR analyses (see ESI‡).

**Scheme 2:**
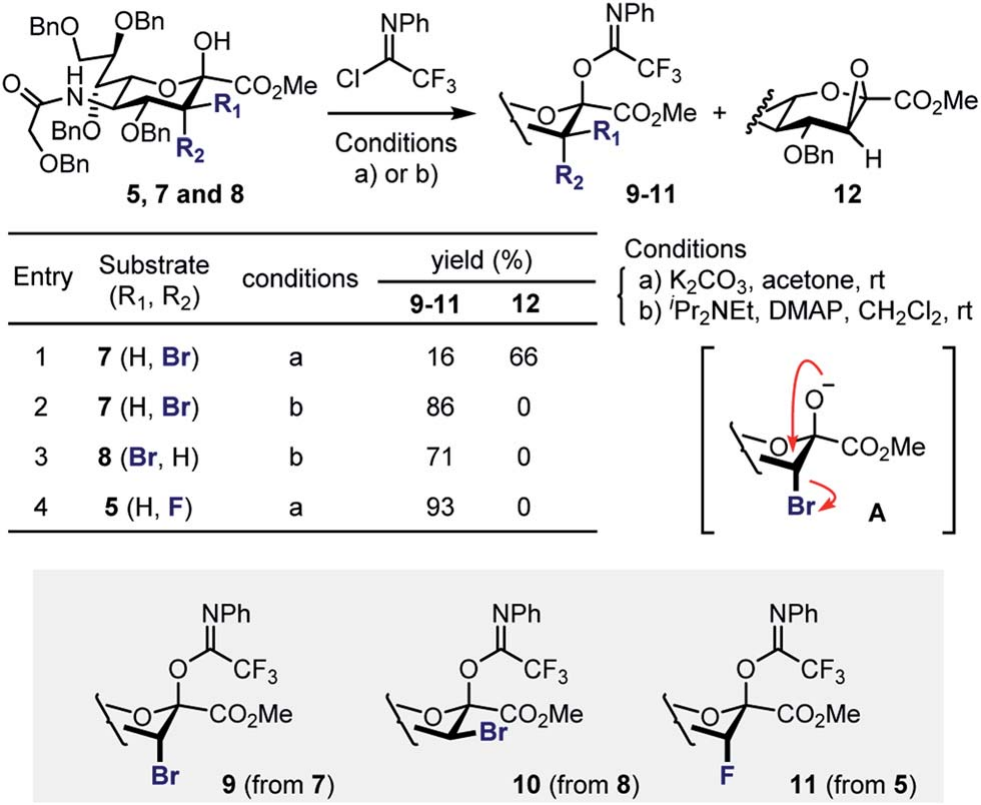
Preparation of the sialyl donors **9–11**.

With the halogenated donors **9–11** in hand, formation of the protected Neu2,6Glu fragment was attempted *via* coupling with **13**, which was prepared from d-glucose (Scheme 3). Initially the fluorinated donor **11** and acceptor **13** were treated with TMSOTf in CH_2_Cl_2_ at 0 °C. Under these conditions, the sialylation reaction proved to be completely stereospecific, affording the disaccharide **14** 61% exclusively as the α-anomer. The stereochemical course is consistent with the induction model described in Fig. 1.^11*d*^

**Scheme 3:**
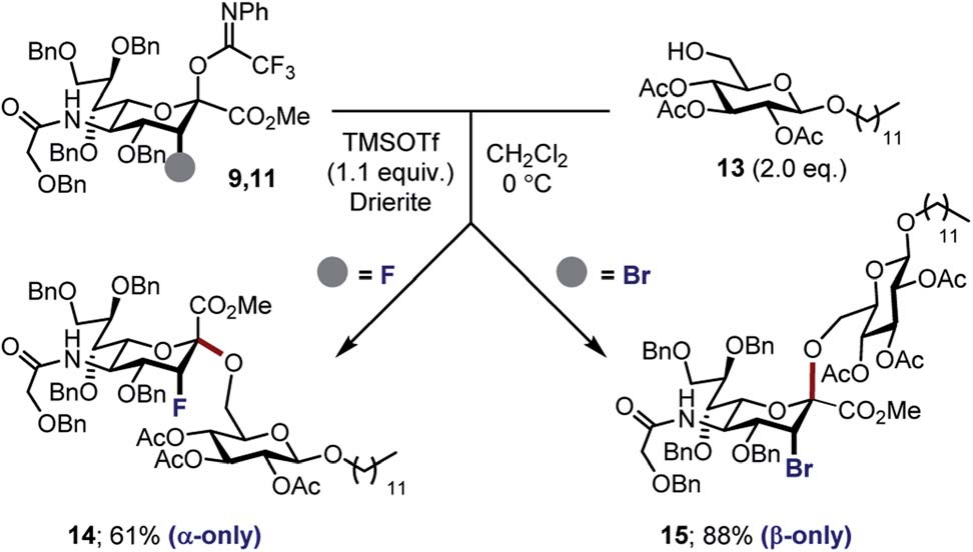
Sialylation of 3-Br_ax_ donor **9** and 3-F_ax_ donor **11**.

By comparison, sialylation using the brominated donor **9** under identical conditions generated the β-anomer **15** (88%). This can be rationalized by invoking anchimeric assistance *via* the bromonium ion intermediate with concomitant ring opening. These data indicate that C3 halogen-directed pseudo-stereodivergence is efficient and stereospecific in steering chemical sialylation. As a control experiment, donor **10** was subjected to the sialylation conditions. This system, in which the C(sp^3^)–Br bond is pseudo-equatorial, proved to be challenging and gave an inseparable mixture of disaccharides. However, a subsequent radical reduction (Bu_3_SnH, AIBN, toluene, reflux) enabled the separation of both anomers. The sialylation is clearly sensitive to the configuration at C3, with an overall drop in selectivity and efficiency being observed. Nonetheless, it was possible to isolate the α-anomer **16** in 30% yield, and the β-anomer **17** in 4% (Scheme 4). The predominant generation of the α-anomer is also consistent with neighbouring group participation, but the pseudo-equatorial orientation no longer mitigates potential elimination following activation and this may be reflected in yield. The reduction does, however, validate the use of the C3 bromo substituent as a traceless directing group.

**Scheme 4:**
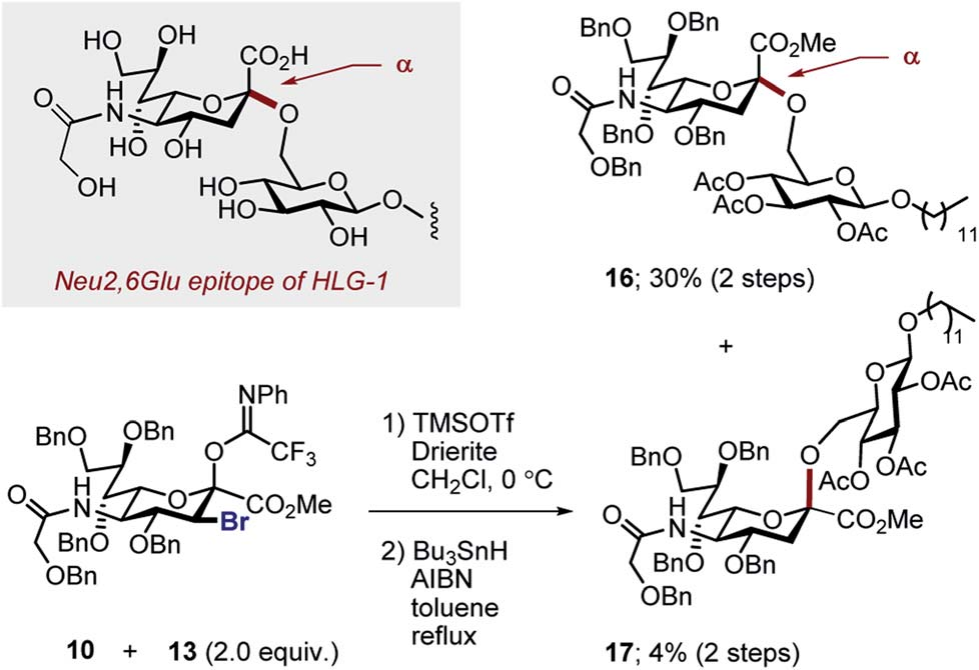
Comparative sialylation of 3-Br_eq_ donor **10**.

With this preliminary validation of halogen-dependent pseudo-stereodivergence, it was necessary to demonstrate (a) that the fluorinated epitope is compatible with standard deprotection conditions, and (b) that the selectivity observed represents a methodological advance over the non-fluorinated case.

To that end, the tolerance of **14** towards standard global deprotection conditions was investigated (Scheme 5). Gratifyingly, hydrogenolysis yielded the target disaccharide **18** (81% yield), and this could be further hydrolysed to **19** in 94% yield.

**Scheme 5:**
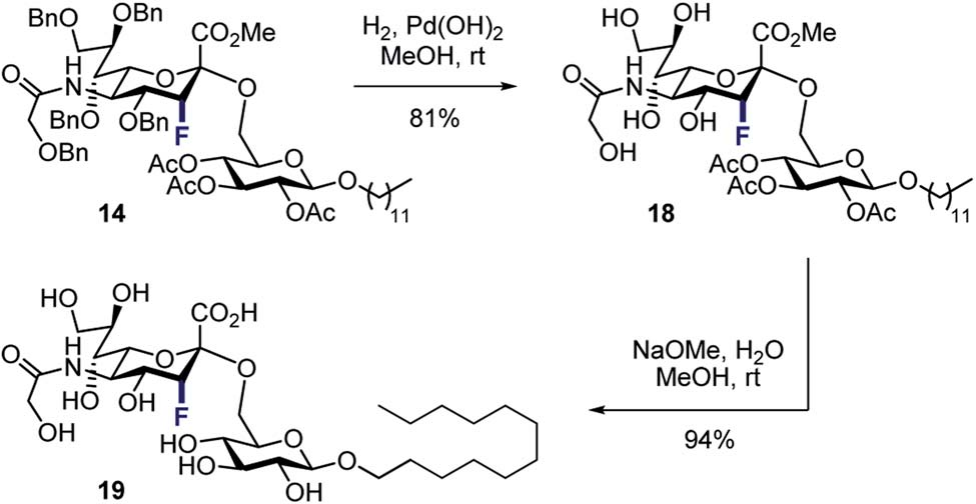
Deprotection of disaccharide **14** to generate **19**.

Finally, to ensure a direct comparison of the fluorinated *versus* non-fluorinated donors, the commonly employed phosphite donor **20** was employed for simplicity (Scheme 6). Moreover, performing this with the N–Ac derivatives allowed the fluorinated and non-fluorinated epitopes of Hp-s1 to be accessed. Glycosylation of N–Ac fluorinated phosphite donor **20** with **13**, followed by hydrogenolysis of benzyl groups afforded disaccharide **21** exclusively as the α-anomer in 58% (2 steps). Subsequent hydrolysis liberated the target scaffold **22** in 85% yield.

**Scheme 6:**
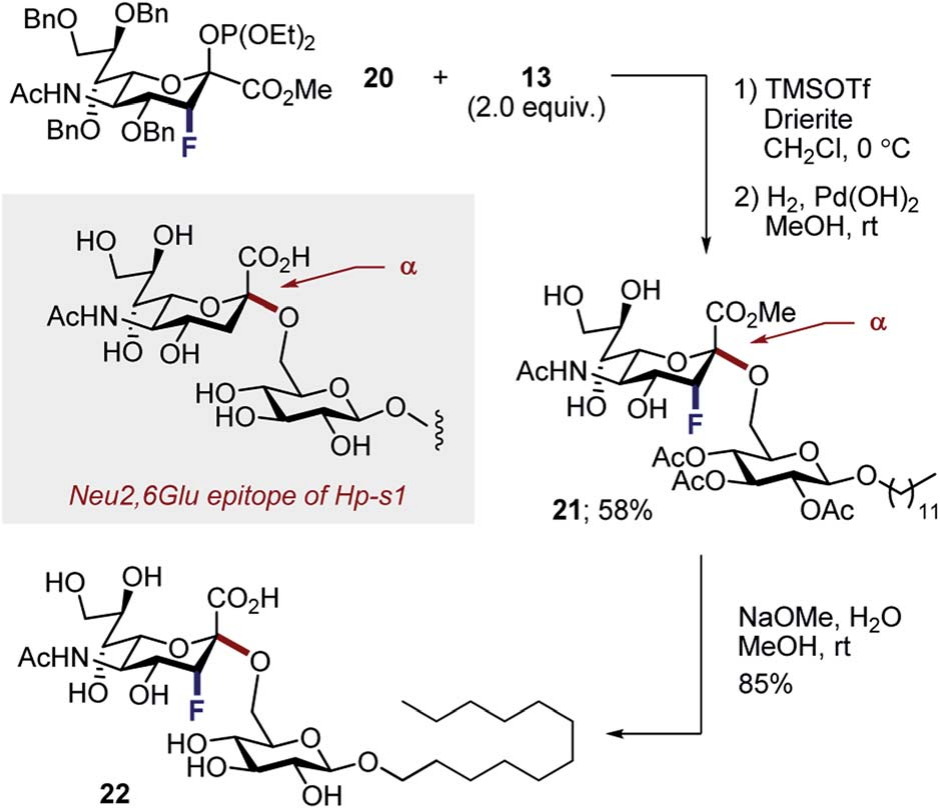
Synthesis of the fluorinated Hp-s1 analogue **22**.

For comparison, the non-fluorinated Hp-s1 was prepared by exposure of phosphite **23** and the benzyl-protected donor **24** to TMSOTf in a mixed solvent (MeCN, CH_2_Cl_2_, v/v = 2/1). The reaction proceeded non-selectively to give an approximate 1 : 1 mixture of anomers (α-anomer **25**, 25%; β-anomer **26**, 21%. Scheme 7). For completeness, global deprotection by sequential hydrogenolysis/hydrolysis furnished the α- and β-anomers **27** and **28**, respectively.

**Scheme 7:**
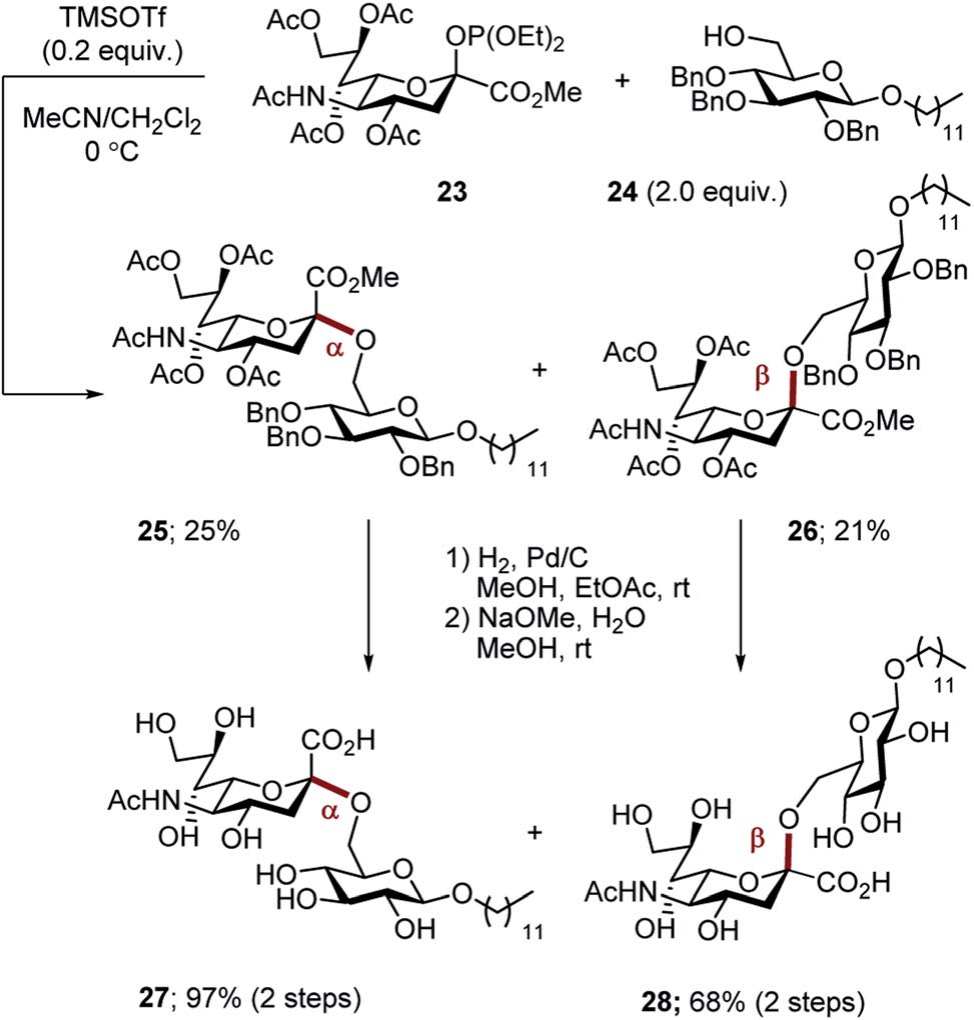
Synthesis of non-fluorinated Hp-s1 analogues **27** and **28**.

## Conclusions

**Scheme 8:**
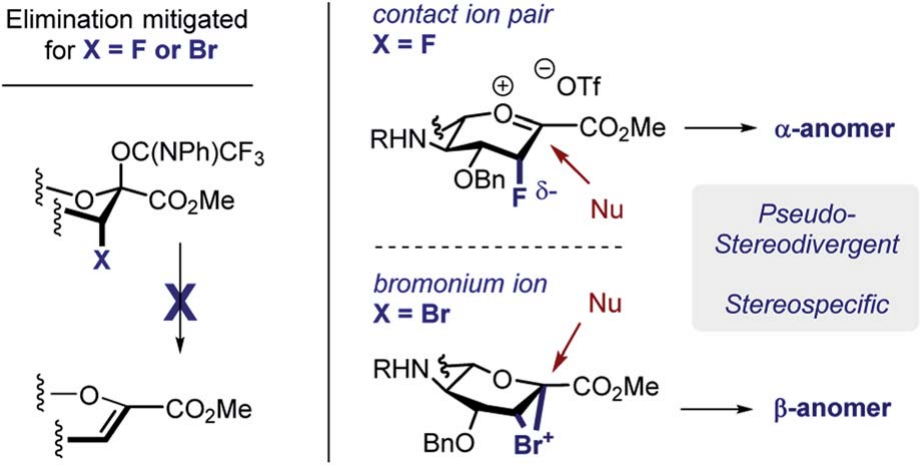
Pseudo-stereodivergent sialylation.

The concept of pseudo-stereodivergent sialylation is disclosed and applied to the synthesis of the Neu2,6Glu core of the marine gangliosides HLG-1, and Hp-s1. In the case of *N*-Gc sialic acid donors, the introduction of axial halogen substituents supresses competing elimination, and facilitates stereospecific sialylation (Scheme 8). It is postulated that in the case of the C-3 fluoro substituent, the close contact ion pair (CIP) is highly pre-organised with the triflate on the opposite face to mitigate electrostatic repulsion. Nucleophilic attack thus favours formation of the α-anomer in a stereospecific manner. Whilst an S_N_2-like process cannot be completely discounted, the steric demand of the d-glucose-derived acceptor renders it unlikely. In contrast, the C-3 bromo substituent can generate a bromonium ion intermediate upon imidate activation. The stereoelectronic requirements for productive bond formation ensure that the β-anomer is generated. Facile reduction of the C(sp^3^)–Br bond renders this process traceless, whereas the fluorine substituent provides a useful NMR probe for structural analyses.^19^ Exploring the biological effects on these halogenated epitopes on non-neuronal glial cell behaviour will be the subject of future research endeavours in our laboratory.

## Conflicts of interest

There are no conflicts to declare.

## Supplementary Material

SC-011-D0SC01219J-s001
