# Supplementary material for: Halogen-directed chemical sialylation: pseudo-stereodivergent access to marine ganglioside epitopes
Source: Chem Sci. 2020 Mar 26;11(25):6527–31. doi: 10.1039/d0sc01219j (PMC8152791; doi:10.1039/d0sc01219j)

Electronic Supplementary Material (ESI) for Chemical Science.
This journal is © The Royal Society of Chemistry 2020

Supporting Information

Halogen-Directed Chemical Sialylation: Pseudo-Stereodivergent Access to Marine Ganglioside Epitopes

Taiki Hayashi, Alexander Axer, Gerald Kehr, Klaus Bergander and Ryan Gilmour

Organisch-Chemisches Institut - Excellence Cluster "Cells in Motion", Westfälische Wilhelms-Universität Münster, Corrensstrasse 40, 48149 Münster, Germany

Table of contents

General Information S2
Experimental procedures and analytical data S3
- Carboxylic acid **2** and the respective methyl ester **3** S3
  - Amine **S1** S4
- Compound **4** S4
- Compounds **5** and **6** S5
- Compounds **7** and **8** S6
- Imidate **9** and epoxide **12** S7
- Compound **10** S9
- Compound **11** S10
- Preparation scheme for acceptors **13** and **24** S11
  - Compound **S3** S11
  - Compound **S4** S12
  - Compound **S5** S12
- Compound **13** S13
- Compound **24** S13
- Compound **14** S14
- Compound **15** S15
- Compounds **16** and **17** S16
- Compound **18** S18
- Compound **19** S18
- Compound **21** S19
- Compound **22** S20
- Compound **23** S20
- Compounds **25** and **26** S21
- Compound **27** S23
- Compound **28** S24

References S25
NMR spectra S26

General Information

All reactions were performed under an atmosphere of argon in dried glassware, except when using aqueous reagents. Dichloromethane was distilled successively from P_2O_5 and CaH_2 and stored over 4A molecular sieves. All chemicals were reagent grade and used as supplied unless stated otherwise. All reactions were magnetically stirred. Solvents for extractions and chromatography were technical grade and were distilled prior to usage. Extracts were dried over technical grade Na_2SO_4. Analytical thin layer chromatography (TLC) was performed on pre-coated *Merck* silica gel 60 F_{254} plates (0.25 mm) and visualized by UV or CAN stain. Column chromatography was carried out on *Fluka* silica gel 60 (230-400 mesh). Concentration *in vacuo* was performed at ca 10 mbar and 45 °C, drying at 10^{-2} mbar and room temperature. ^{1}H NMR, ^{13}C NMR and ^{19}F NMR spectra were recorded on a *Bruker AV 300 MHz, Bruker AV 400 MHz*, *Agilent DD2 500* and an *Agilent DD2 600* spectrometer. Chemical shifts (δ) are reported in ppm relative to the solvent residual peak (7.26 ppm for $CDCl_3$, 5.32 ppm for CD_2Cl_2, 4.87 ppm for CD_3OD). The multiplicities are reported as: s = singlet, d = doublet, t = triplet, q = quartet, quin = quintet, m = multiplet, br = broad. NMR assignments were supported by additional 2D NMR experiments. Melting points were measured on a *Büchi B540* melting point apparatus. IR spectra of the neat compounds were measured on a *Perkin-Elmer Spectrum 100 FTIR* spectrometer and are reported in cm-1. Optical rotations were obtained using a *JASCO P-2000* polarimeter. Mass spectra (ESI-MS, MALDI-TOF MS and NSI-FTMS) were performed by the MS service at the Organic Chemistry Institute of the WWU Münster.

Carboxylic acid **2** and the respective methyl ester **3**

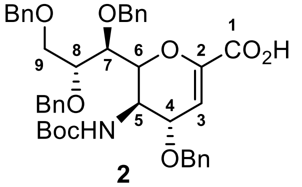

2

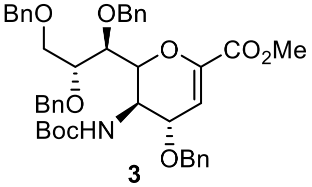

3

DMAP (86.0 mg, 0.704 mmol, 25 mol%) was added to a mixture of methyl 5-acetamido-4,7,8,9-tetra-*O*-benzyl-2,6-anhydro-3,5-dideoxy-D-glycero-D-galacto-non-2-enonate (**1**) (1.88 g, 2.82 mmol)[1] and Boc_2O (1.23 g, 5.64 mmol, 2.0 eq.) in THF (10 mL) at room temperature. Then the mixture was refluxed for 2.5 h. After cooling to room temperature, the mixture was concentrated in vacuo. The residue was purified by chromatographic purification (SiO_2, cHex:EtOAc = 8:2) to give crude material. Subsequently K_2CO_3 (782 mg, 5.66 mmol, 2.0 eq.) was added to a solution of the obtained crude material in MeOH (30 mL) at room temperature. After stirring for 16 h, the mixture was acidified by adding 1 M aqueous HCl at 0 ºC. The mixture was extracted with EtOAc (×3). The combined organic layer was successively washed with water and brine, dried (Na_2SO_4) and concentrated in vacuo. The residue was purified by chromatographic purification (SiO_2, cHex:EtOAc = 9:1 to CH_2Cl_2:MeOH = 9:1) to afford compound **2** (1.47 g, 2.07 mmol, 73%) as a white amorphous solid and compound **3** (497 mg, 0.687 mmol, 24%) as a colorless oil.

*Conversion of the carboxylic acid **2** to the methyl ester **3***

MeI (0.20 mL, 3.2 mmol, 1.6 eq.) was added to a solution of compound **2** (1.47 g, 2.07 mmol) and K_2CO_3 (430 mg, 3.11 mmol, 1.5 eq.) in DMF (15 mL) at room temperature. After stirring for 1 h, the mixture was diluted with Et_2O and acidified by adding 1 M aqueous HCl at 0 °C. The mixture was extracted with Et_2O (×3). The combined organic layer was successively washed with water, saturated aqueous $NaHCO_3$ and brine, dried (Na_2SO_4) and concentrated in vacuo. The residue was purified by chromatographic purification (SiO_2, cHex:EtOAc = 8:2) to afford compound **3** (1.47 g, 2.03 mmol, 98%) as a white amorphous solid.

2: $[\alpha]_D^{26}$ -2.7 (c 1.00, $CHCl_3$); ^1H NMR (500 MHz, $CDCl_3$) δ 7.39–7.18 (20H, overlapped, Bn), 6.19 (d, *J* = 3.1 Hz, 1H, C3), 5.47 (br, 1H, CO_2H), 4.70 (d, *J* = 11.7 Hz, 1H, O4-Bn), 4.63 (s, 2H, O7-Bn), 4.55 (s, 2H, O9-Bn), 4.59–4.38 (5H, overlapped, C6+O4-Bn+2×O8-Bn+NH), 4.27 (m, 1H, C4), 4.12 (m, 1H, C7), 4.00 (m, 1H, C5), 3.97–3.83 (2H, overlapped, C8+C9), 3.68 (m, 1H, C9), 1.43 (s, 9H, ^{t}Bu); $^{13}C\{^1H\}$ NMR (126 MHz, $CDCl_3$) δ 163.5 (C1), 155.1 (Boc), 143.4 (C2), [138.2, 138.1, 138.0, 137.9, 128.8, 128.59, 128.57, 128.55, 128.5, 128.14, 128.05, 127.9, 127.8] (Bn, 3 signals are missing, possibly due to overlapping), 110.2 (C3), 80.1 (Boc), 78.5 (C8), 78.1 (C6), 74.9 (C7), 74.3 (O7-Bn), 73.5 (O9-Bn), 73.0 (C4), 72.2 (O8-Bn), 70.8 (O4-Bn), 68.7 (C9), 49.2 (C5), 28.5 (Boc); ESI-MS found: 732.3131 calcd: 732.3143 for $C_{42}H_{47}NNaO_9$ $[M+Na]^+$; IR: 3065, 3032, 2978, 2928, 2869, 1708, 1653, 1497, 1454, 1392, 1367, 1287, 1243, 1216, 1155, 1121, 1088, 1071, 1027, 922, 884, 820, 748, 697, 667.

3: $[\alpha]_D^{28}$ +4.1 (c 1.00, $CHCl_3$); ^1H NMR (500 MHz, $CDCl_3$) δ 7.39–7.21 (20H, overlapped, Bn), 6.12 (d, *J* = 3.4 Hz, 1H, C3), 4.70 (d, *J* = 11.7 Hz, 1H, O4-Bn), 4.67 (d, *J* = 11.0 Hz, 1H, O7-Bn), 4.62 (d, *J* = 11.0 Hz, 1H, O7-Bn), 4.57 (d, *J* = 11.7 Hz, 1H, O4-Bn), 4.554 (s, 2H, O9-Bn), 4.550 (d, *J* = 11.9 Hz, 1H, O8-Bn), 4.490 (d, *J* = 11.9 Hz, 1H, O8-Bn), 4.485 (dd, *J* = 7.7, 4.4 Hz, 1H, C6), 4.43 (m, 1H, NH), 4.20 (dd, *J* = 3.6, 3.4 Hz, 1H, C4), 4.15 (dd, *J* = 6.0, 4.4 Hz, 1H, C7), 4.09 (m, 1H, C5), 3.97 (m, 1H, C8), 3.86 (dd, *J* = 10.3, 3.7 Hz, 1H, C9), 3.76 (s, 3H, Me), 3.71 (dd, *J* = 10.3, 4.5 Hz, 1H, C9), 1.44 (s, 9H, ^{t}Bu); $^{13}C\{^1H\}$ NMR (126 MHz, $CDCl_3$) δ 162.7 (C1), 155.0 (Boc), 144.3 (C2), [138.7, 138.6, 138.4, 138.0, 128.7, 128.54, 128.48, 128.41, 128.35, 128.2, 127.94, 127.85, 127.83, 127.79, 127.7, 127.6] (Bn), 109.3 (C3), 79.9 (Boc), 78.2 (C8), 78.0 (C6), 75.1 (C7), 74.5 (O7-Bn), 73.5 (O9-Bn), 73.1 (C4), 72.4 (O8-Bn), 70.7 (O4-Bn), 69.1 (C9), 52.4 (Me), 48.7 (C5), 28.5 (Boc); ESI-MS found: 746.3319 calcd: 746.3300 for

$C_{43}H_{49}NNaO_9$ [M+Na]$^+$; IR: 3360, 3064, 3032, 2976, 2867, 1713, 1653, 1606, 1587, 1497, 1455, 1439, 1391, 1365, 1304, 1250, 1158, 1089, 1072, 1027, 911, 883, 825, 794, 734, 696.

Amine **S1**

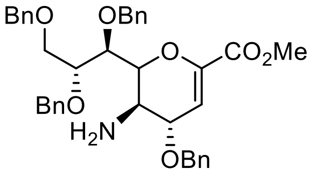

Phenol (3.80 g, 40.4 mmol, 15 eq.) and TMSCl (1.70 mL, 13.5 mmol, 5.0 eq.) were successively added to a solution of the methyl ester **3** (1.95 g, 2.69 mmol) in CH_2Cl_2 (10 mL) at room temperature. After stirring for 1 h, the reaction was quenched by adding saturated aqueous $NaHCO_3$ at 0 ºC. The mixture was extracted with CH_2Cl_2 (×3). The combined organic layer was dried (Na_2SO_4) and concentrated in vacuo. The residue was purified by chromatographic purification (SiO_2, cHex:EtOAc = 8:2 to 1:1) to give compound **S1** (1.62 g, 2.60 mmol, 97%) as a colorless oil.

S1: $[\alpha]_D^{28}$ +23.3 (c 1.00, $CHCl_3$); ^{1}H NMR (599 MHz, $CDCl_3$) δ 7.45–7.19 (20H, overlapped, Bn), 6.12 (m, 1H, C3), 4.75 (d, *J* = 11.6 Hz, 1H, O7-Bn), 4.72 (d, *J* = 11.6 Hz, 1H, O7-Bn), 4.70 (d, *J* = 11.5 Hz, 2H, O4-Bn+O8-Bn), 4.60 (d, *J* = 11.5 Hz, 1H, O8-Bn), 4.58 (s, 2H, O9-Bn), 4.50 (d, *J* = 11.5 Hz, 1H, O4-Bn), 4.15 (m, 1H, C7), 4.08 (brd, *J* = 9.8 Hz, 1H, C6), 4.04 (m, 1H, C8), 4.01 (m, 1H, C4), 3.90 (m, 1H, C9), 3.77 (s, 3H, Me), 3.73 (m, 1H, C9), 3.23 (dd, *J* = 9.8, 8.8 Hz, 1H, C5), 1.47 (br, 2H, NH_2); ^{13}C{^{1}H} NMR (151 MHz, $CDCl_3$) δ 162.8 (C1), 145.0 (C2), [138.7, 138.5, 138.2, 137.9, 128.7, 128.56, 128.55, 128.5, 128.4, 128.2, 128.13, 128.07, 128.0, 127.9, 127.7, 127.6] (Bn), 108.6 (C3), 79.9 (br, C6), 78.1 (C8), 77.7 (br, C4), 74.9 (C7), 73.7 (O7-Bn), 73.6 (O9-Bn), 72.9 (O8-Bn), 70.5 (O4-Bn), 69.7 (C9), 52.3 (Me), 48.9 (C5); ESI-MS found: 624.2976 calcd: 624.2956 for $C_{38}H_{42}NO_7$ [M+H]$^+$; IR: 3383, 3063, 3030, 2864, 1732, 1651, 1604, 1496, 1454, 1438, 1363, 1330, 1262, 1208, 1090, 1073, 1027, 987, 910, 881, 847, 821, 790, 737, 697.

Compound **4**

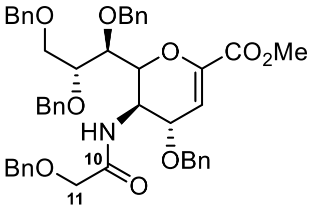

Benzyloxyacetyl chloride (0.62 mL, 3.93 mmol, 1.5 eq.) was added to a mixture of compound **S1** (1.62 g, 2.60 mmol) and NEt_3 (0.72 mL, 5.2 mmol, 2.0 eq.) at 0 ºC. After stirring for 45 min at room temperature, the reaction was quenched by adding saturated aqueous $NaHCO_3$ at 0 ºC. The mixture was extracted with CH_2Cl_2 (×3). The combined organic layer was dried (Na_2SO_4) and concentrated in vacuo. The residue was purified by chromatographic purification (SiO_2, cHex:EtOAc = 75:25 to 7:3) to give the glycal **4** (1.94 g, 2.51 mmol, 97%) as a colorless oil.

4: $[\alpha]_D^{27}$ +3.5 (c 1.00, $CHCl_3$); ^{1}H NMR (599 MHz, $CDCl_3$) δ 7.36– 7.21 (25H, overlapped, Bn), 6.63 (d, *J* = 8.2 Hz, 1H, NH), 6.16 (d, *J* = 3.8 Hz, 1H, C3), 4.69 (d, *J* = 11.7 Hz, 1H, O4-Bn), 4.66 (d, *J* = 10.9 Hz, 1H, O7-Bn), 4.63 (dd, *J* = 6.2, 5.6 Hz, 1H, C6), 4.61 (d, *J* = 10.9 Hz, 1H, O7-Bn), 4.57 (d, *J* = 11.7 Hz, 1H, O4-Bn), 4.53 (d, *J* = 12.0 Hz, 1H, O9-Bn), 4.51 (d, *J* = 12.0 Hz, 1H, O9-Bn), 4.50 (d, *J* = 11.8

Hz, 1H, O8-Bn), 4.47 (s, 2H, O11-Bn), 4.46 (d, J = 11.8 Hz, 1H, O8-Bn), 4.44 (ddd, J = 8.2, 5.6, 4.5 Hz, 1H, C5), 4.19 (dd, J = 6.2, 5.4 Hz, 1H, C7), 4.15 (dd, J = 4.5, 3.8 Hz, 1H, C4), 4.00 (ddd, J = 5.4, 5.1, 4.5 Hz, 1H, C8), 3.89 (dd, J = 10.1, 5.1 Hz, 1H, C9), 3.87 (d, J = 15.1 Hz, 1H, C11), 3.81 (d, J = 15.1 Hz, 1H, C11), 3.78 (s, 3H, Me), 3.69 (dd, J = 10.1, 4.5 Hz, 1H, C9); $^{13}C\{^1H\}$ NMR (151 MHz, $CDCl_3$) δ 169.4 (C10), 162.7 (C1), 143.9 (C2), [138.7, 138.6, 138.3, 137.9, 136.7, 128.8, 128.6, 128.42, 128.40, 128.35, 128.33, 128.31, 128.23, 128.20, 128.0, 127.8, 127.68, 127.67, 127.6, 127.5] (Bn), 109.0 (C3), 78.1 (C8), 77.9 (C6), 75.7 (C7), 75.0 (O7-Bn), 73.7 (O11-Bn), 73.6 (O9-Bn), 72.3 (O8-Bn), 71.7 (C4), 70.8 (O4-Bn), 69.4 (C11), 69.0 (C9), 52.5 (Me), 47.5 (C5); ESI-MS found: 794.3308 calcd: 794.3300 for $C_{47}H_{49}NNaO_9$ $[M+Na]^+$; IR: 3359, 3032, 2869, 1731, 1660, 1608, 1523, 1497, 1455, 1349, 1308, 1264, 1204, 1168, 1127, 1091, 1028, 913, 844, 820, 750, 715, 699, 671.

Compounds **5** (with the fluorine atom at the axial position) and **6** (with the fluorine atom at the equatorial position)

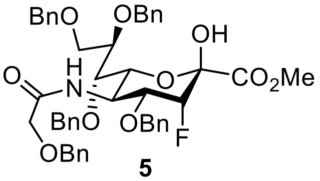

5

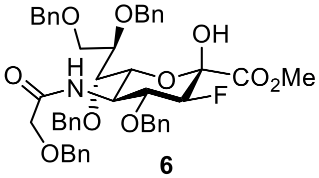

6

Selectfluor® (574 mg, 1.62 mmol, 4.1 eq.) was added to a mixture of **4** (303 mg, 0.393 mmol) in DMF/water (3/1, 8 mL) at room temperature. The reaction mixture was heated at 60 ºC for 3.25 h. After cooling to room temperature, the reaction was quenched by adding saturated aqueous $NaHCO_3$ at 0 ºC. The mixture was extracted with Et_2O (×3). The combined organic layer was washed with brine, dried (Na_2SO_4) and concentrated in vacuo. The residue was purified by chromatographic purification (SiO_2, cHex:EtOAc = 75:25 to 65:35) to afford compound **5** (193 mg, 0.239 mmol, 61%) and compound **6** (61.0 mg, 0.0750 mmol, 19%), both as a white amorphous solid.

5[2]: $[\alpha]_D^{26}$ -4.5 (c 1.00, $CHCl_3$); The solution of obtained **5** in $CDCl_3$ showed a mixture of compounds (93:7 based on ^{19}F NMR) tentatively assigned as isomers; ^1H NMR (599 MHz, $CDCl_3$, The signals for minor isomer are omitted) δ 7.37–7.18 (25H, overlapped, Bn), 6.28 (d, J = 8.7 Hz, 1H, NH), 4.87 (m, 1H, C3), 4.74 (d, J = 11.8 Hz, 1H, O8-Bn), 4.68 (d, J = 11.9 Hz, 1H, O4-Bn), 4.67 (d, J = 11.1 Hz, 1H, O7-Bn), 4.58 (d, J = 11.1 Hz, 1H, O7-Bn), 4.57 (s, 2H, O9-Bn), 4.54 (d, J = 11.8 Hz, 1H, O8-Bn), 4.504 (brd, J = 10.6 Hz, 1H, C6), 4.498 (d, J = 11.9 Hz, 1H, O4-Bn), 4.47 (s, 2H, O11-Bn), 4.31 (ddd, J = 10.6, 10.5, 8.7 Hz, 1H, C5), 4.23 (m, 1H, C4), 3.94 (m, 1H, C8), 3.89 (m, 1H, C9), 3.88 (d, J = 15.0 Hz, 1H, C11), 3.86 (dd, J = 8.1, 1.6 Hz, 1H, C7), 3.83 (d, J = 15.0 Hz, 1H, C11), 3.80 (s, 3H, Me), 3.69 (dd, J = 10.7, 4.2 Hz, 1H, C9), 3.23 (br, 1H, OH); $^{13}C\{^1H\}$ NMR (151 MHz, $CDCl_3$, The signals for minor isomer are omitted) δ 170.1 (C10), 168.6 (C1), [138.8, 138.33, 138.30, 137.8, 136.8, 128.7, 128.58, 128.57, 128.5, 128.42, 128.41, 128.37, 128.36, 128.1, 128.02, 127.95, 127.9, 127.81, 127.75] (Bn, one signal is missing, possibly due to overlapping), 94.2 (d, $^2J_{FC}$ = 24.5 Hz, C2), 85.6 (d, $^1J_{FC}$ = 185.7 Hz, C3), 77.5 (C8), 75.3 (C7), 74.8 (d, $^2J_{FC}$ = 17.8 Hz, C4), 74.0 (O7-Bn), 73.6 (2C, O9-Bn+O11-Bn), 72.4 (O8-Bn), 71.7 (O4-Bn), 70.7 (C6), 69.7 (C11), 69.3 (C9), 53.5 (Me), 47.7 (d, $^3J_{FC}$ = 2.2 Hz, C5); ^{19}F NMR (564 MHz, $CDCl_3$, The signal for minor isomer is marked with an asterisk) δ -206.3 (dd, $^2J_{FH}$ = 50.1 Hz, $^3J_{FH}$ = 27.5 Hz), -217.5* (m); ESI-MS found: 830.3323 calcd: 830.3311 for $C_{47}H_{50}FNNaO_{10}$ $[M+Na]^+$; IR: 3380, 3064, 3031, 2867, 1738, 1661, 1586, 1532, 1497, 1454, 1373, 1246, 1208, 1094, 1063, 1028, 911, 890, 802, 736, 697.

6: $[\alpha]_D^{25}$ -14.6 (c 1.00, $CHCl_3$); The solution of obtained **6** in $CDCl_3$ showed a mixture of compounds (96:4 based on ^{19}F NMR) tentatively assigned as isomers; ^1H NMR (599 MHz, $CDCl_3$, The signals for minor isomer are omitted) δ 7.40–7.20 (25H, overlapped, Bn), 6.38 (d, J = 8.9 Hz, 1H, NH), 4.91 (dd, $^2J_{FH}$ = 49.7 Hz, J = 9.9 Hz, 1H, C3), 4.85 (d, J = 11.3 Hz, 1H, O4-Bn), 4.68 (d, J = 10.4 Hz, 1H, O7-Bn),

4.67 (d, J = 11.5 Hz, 1H, O8-Bn), 4.574 (d, J = 12.2 Hz, 1H, O9-Bn), 4.567 (d, J = 11.3 Hz, 1H, O4-Bn), 4.55 (d, J = 12.2 Hz, 1H, O9-Bn), 4.51 (d, J = 11.7 Hz, 1H, O11-Bn), 4.48 (d, J = 11.5 Hz, 1H, O8-Bn), 4.46 (d, J = 11.7 Hz, 1H, O11-Bn), 4.44 (d, J = 10.4 Hz, 1H, O7-Bn), 4.46–4.37 (2H, overlapped, C5+C6), 4.01–3.94 (2H, overlapped, C4+OH), 3.92 (d, J = 15.2 Hz, 1H, C11), 3.89 (d, J = 15.2 Hz, 1H, C11), 3.85 (s, 3H, Me), 3.80 (d, J = 8.8 Hz, 1H, C7), 3.77 (dd, J = 10.6, 2.3 Hz, 1H, C9), 3.73 (m, 1H, C8), 3.65 (dd, J = 10.6, 3.1 Hz, 1H, C9); $^{13}C\{^1H\}$ NMR (151 MHz, $CDCl_3$, The signals for minor isomer are omitted) δ 169.8 (C10), 168.9 (d, $^3J_{FC}$ = 1.1 Hz, C1), [138.4, 138.20, 138.18, 138.17, 136.7, 128.8, 128.63, 128.60, 128.55, 128.5, 128.43, 128.41, 128.35, 128.3, 128.1, 128.0, 127.88, 127.86, 127.82, 127.80] (Bn), 93.7 (d, $^2J_{FC}$ = 21.9 Hz, C2), 91.3 (d, $^1J_{FC}$ = 192.5 Hz, C3), 78.7 (d, $^2J_{FC}$ = 16.6 Hz, C4), 76.9 (C8), 75.2 (C7), 74.8 (O7-Bn), 74.7 (d, $^4J_{FC}$ = 3.0 Hz, O4-Bn), 73.7 (O11-Bn), 73.6 (O9-Bn), 72.5 (O8-Bn), 70.5 (C6), 69.6 (C11), 68.2 (C9), 54.1 (Me), 49.9 (d, $^3J_{FC}$ = 8.4 Hz, C5); ^{19}F NMR (564 MHz, $CDCl_3$, The signal for minor isomer is marked with an asterisk) δ -197.4 (dd, $^2J_{FH}$ = 49.7 Hz, $^3J_{FH}$ = 11.9 Hz), -210.0* (dd, $^2J_{FH}$ = 49.0 Hz, $^3J_{FH}$ = 28.3 Hz).
ESI-MS found: 830.3334 calcd: 830.3311 for $C_{47}H_{50}FNNaO_{10}$ $[M+Na]^+$; IR: 3383, 3063, 3031, 2863, 1748, 1683, 1605, 1587, 1525, 1497, 1454, 1369, 1276, 1168, 1091, 1076, 1049, 1027, 913, 853, 822, 802, 778, 735, 696.

$^{13}C\{^1H_{sel},^{19}F_{sel}\}$ (δ^1H_{sel} = 3.85 (OMe), $\delta^{19}F_{sel}$ = -197.4)[3]: δ 168.9 (d, $^3J_{CH}$ ≈ 1.5 Hz, C1).

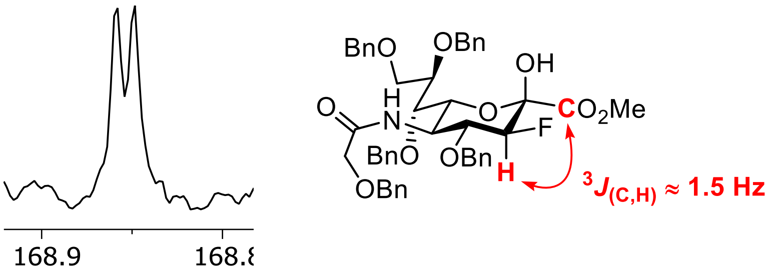

Compounds **7** (with the bromine atom at the axial position) and **8** (with the bromine atom at the equatorial position)

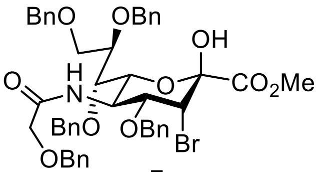

7

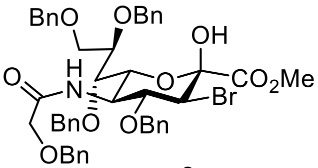

8

NBS (165 mg, 0.927 mmol, 2.0 eq.) was added to a mixture of compound **4** (357 mg, 0.462 mmol) in DMF/water (5/1, 9.6 mL) at room temperature. After stirring for 30 min, the reaction was quenched by adding saturated aqueous $NaHCO_3$ and saturated $Na_2S_2O_3$ at 0 ºC. The mixture was extracted with Et_2O (×3). The combined organic layer was washed with brine, dried (Na_2SO_4) and concentrated in vacuo. The residue was purified by chromatographic purification (SiO_2, cHex:EtOAc = 8:2 to 65:35) to afford compound **7** (231 mg, 0.266 mmol, 58%) and compound **8** (129 mg, 0.148 mmol, 32%), both as a white amorphous solid.

7: $[\alpha]_D^{27}$ +12.9 (c 1.00, $CHCl_3$); The solution of obtained **7** in $CDCl_3$ showed a mixture of compounds (9:1 based on ^1H NMR) tentatively assigned as isomers; ^1H NMR (599 MHz, $CDCl_3$, discernible signals for the minor isomer are marked with an asterisk) δ 7.40–7.16 (25H, overlapped, Bn), 6.22 (d, J = 7.9 Hz, 1H, NH), 6.12* (d, J = 8.4 Hz, 1H, NH), 4.74 (d, J = 11.9 Hz, 1H, O8-Bn), 4.67 (d, J = 11.1 Hz, 1H, O7-Bn), 4.65 (d, J = 11.8 Hz, 1H, O4-Bn), 4.62 (d, J = 11.1 Hz, 1H, O7-Bn), 4.60 (d, J = 2.9 Hz, 1H,

C3), 4.59 (s, 2H, O9-Bn), 4.53 (d, J = 11.9 Hz, 1H, O8-Bn), 4.49 (dd, J = 9.0, 1.8 Hz, 1H, C6), 4.47 (s, 2H, O11-Bn), 4.36 (d, J = 11.8 Hz, 1H, O4-Bn), 4.30 (dd, J = 9.9, 2.9 Hz, 1H, C4), 4.27 (ddd, J = 9.9, 9.0, 7.9 Hz, 1H, C5), 4.01 (ddd, J = 8.0, 4.1, 2.5 Hz, 1H, C8), 3.92 (dd, J = 10.8, 2.5 Hz, 1H, C9), 3.88 (d, J = 15.0 Hz, 1H, C11), 3.83 (dd, J = 8.0, 1.8 Hz, 1H, C7), 3.82 (d, J = 15.0 Hz, 1H, C11), 3.78 (s, 3H, Me), 3.71 (dd, J = 10.8, 4.1 Hz, 1H, C9), 3.62* (s, 3H, Me), 2.88 (br, 1H, OH); $^{13}C\{^{1}H\}$ NMR (151 MHz, $CDCl_3$, The signals for the minor isomer are omitted) δ 170.2 (C10), 168.1 (C1), [138.9, 138.4, 138.3, 137.7, 136.8, 128.7, 128.63, 128.56, 128.5, 128.4, 128.3, 128.14, 128.07, 127.9, 127.82, 127.81, 127.7] (Bn, 3 signals are missing, possibly due to overlapping), 96.3 (C2), 77.2 (C8), 75.2 (C7), 73.8 (O7-Bn), 73.59 (O9-Bn), 73.55 (O11-Bn), 73.4 (C4), 72.3 (O8-Bn), 70.8 (C6), 70.7 (O4-Bn), 69.6 (C11), 69.1 (C9), 53.1 (Me), 52.2 (C3), 48.4 (C5); ESI-MS found: 890.2503 calcd: 890.2510 for $C_{47}H_{50}BrNNaO_{10}$ $[M+Na]^+$; IR: 3386, 3185, 3088, 3064, 3030, 2918, 2864, 1747, 1661, 1606, 1586, 1533, 1497, 1454, 1394, 1332, 1305, 1257, 1216, 1208, 1092, 1051, 1027, 912, 869, 820, 802, 734, 696, 667.

8: mp 130-135 ºC (decomp.); $[\alpha]_D^{26}$ -43.8 (c 1.00, $CHCl_3$); ^{1}H NMR (599 MHz, $CDCl_3$) δ 7.40– 7.21 (25H, overlapped, Bn), 6.49 (d, J = 9.4 Hz, 1H, NH), 4.90 (d, J = 10.4 Hz, 1H, O4-Bn), 4.69 (d, J = 10.6 Hz, 1H, O7-Bn), 4.66 (d, J = 11.4 Hz, 1H, O8-Bn), 4.59 (d, J = 12.2 Hz, 1H, O9-Bn), 4.564 (d, J = 10.4 Hz, 1H, O4-Bn), 4.557 (d, J = 12.2 Hz, 1H, O9-Bn), 4.52 (d, J = 11.7 Hz, 1H, O11-Bn), 4.50 (brd, J = 10.9 Hz, 1H, C6), 4.50– 4.43 (1H, overlapped, C5), 4.47 (d, J = 11.4 Hz, 1H, O8-Bn), 4.46 (d, J = 10.6 Hz, 1H, O7-Bn), 4.46 (d, J = 11.7 Hz, 1H, O11-Bn), 4.38 (d, J = 10.5 Hz, 1H, C3), 4.16 (brs, 1H, OH), 4.02 (dd, J = 10.5, 9.7 Hz, 1H, C4), 3.91 (d, J = 15.0 Hz, 1H, C11), 3.851 (s, 3H, Me), 3.848 (d, J = 15.0 Hz, 1H, C11), 3.82 (d, J = 9.0 Hz, 1H, C7), 3.77 (dd, J = 10.7, 2.1 Hz, 1H, C9), 3.72 (ddd, J = 9.0, 3.3, 2.1 Hz, 1H, C8), 3.65 (dd, J = 10.7, 3.3 Hz, 1H, C9); $^{13}C\{^{1}H\}$ NMR (151 MHz, $CDCl_3$) δ 169.7 (C10), 168.8 (C1), [138.3, 138.2, 138.1, 137.8, 136.6, 128.8, 128.63, 128.55, 128.54, 128.49, 128.45, 128.41, 128.37, 128.3, 128.12, 128.10, 128.0, 127.90, 127.87, 127.8] (Bn), 95.7 (C2), 80.8 (C4), 76.7 (C8), 75.3 (C7), 75.1 (O4-Bn), 74.8 (O7-Bn), 73.63 (O11-Bn), 73.58 (O9-Bn), 72.5 (O8-Bn), 71.0 (C6), 69.5 (C11), 68.1 (C9), 54.2 (Me), 51.79 (C3), 51.75 (C5); ESI-MS found: 890.2506 calcd: 890.2510 for $C_{47}H_{50}BrNNaO_{10}$ $[M+Na]^+$; IR: 3491, 3388, 3088, 3064, 3032, 3010, 2920, 2865, 1748, 1679, 1606, 1587, 1521, 1497, 1454, 1397, 1357, 1286, 1237, 1216, 1154, 1091, 1042, 1027, 951, 912, 859, 805, 736, 696, 667.

$^{13}C\{^{1}H_{sel}\}$ ($\delta^{1}H_{sel}$ = 3.851 (OMe)): δ 168.8 ($\nu_{1/2} \approx$ 2 Hz, C1).

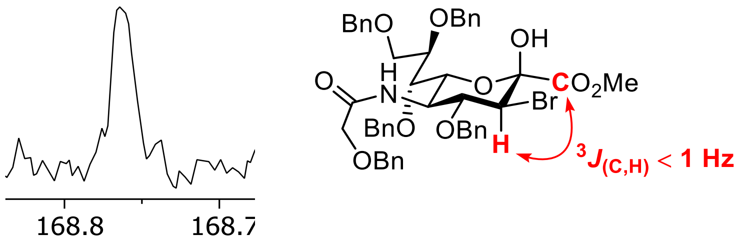

Imidate **9** and epoxide **12**

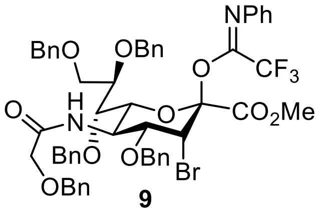

9

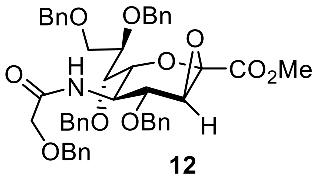

12

K_2CO_3 (40.9 mg, 0.296 mmol, 3.2 eq.) was added to a solution of compound **7** (80.9 mg, 0.0931 mmol) and 2,2,2-trifluoro-*N*-phenylacetimidoyl chloride (60.1 mg, 0.290 mmol, 3.1 eq.) in acetone (1 mL) at room temperature. After stirring for 45 min, the mixture was filtered through a Celite® pad (washed with acetone) and concentrated in vacuo. Chromatographic purification (SiO_2, cHex:EtOAc = 9:1 to 8:2) gave imidate **9** (15.5 mg, 0.0149 mmol, 16%) and epoxide **12** (48.2 mg, 0.0612 mmol, 66%), both as a colorless oil.

<u>Selective preparation of compound</u> ***<u>9</u>***

$^{i}Pr_2NEt$ (51 μL, 0.29 mmol, 3.0 eq.) and DMAP (2.5 mg, 0.020 mmol, 0.2 eq.) were successively added to a mixture of compound **7** (84.8 mg, 0.0976 mmol) and 2,2,2-trifluoro-*N*-phenylacetimidoyl chloride (40.6 mg, 0.196 mmol, 2.0 eq.) in CH_2Cl_2 (1 mL) at room temperature. After stirring for 2 h, the mixture was concentrated in vacuo. Chromatographic purification (SiO_2, cHex:EtOAc = 9:1 to 85:15) afforded imidate **9** (87.7 mg, 0.0843 mmol, 86%) as a colorless oil.

9: $[\alpha]_D^{24}$ -30.4 (c 1.00, MeCN); ^{1}H NMR (599 MHz, CD_2Cl_2) δ 7.40 (m, 2H, Bn), 7.36–7.22 (23H, overlapped, Bn), 7.14 (m, 2H, *m*-NPh), 7.05 (m, 1H, *p*-NPh), 6.61 (m, 2H, *o*-NPh), 6.29 (d, *J* = 8.6 Hz, 1H, NH), 4.75 (d, *J* = 3.5 Hz, 1H, C3), 4.74 (d, *J* = 10.9 Hz, 1H, O7-Bn), 4.72 (d, *J* = 11.8 Hz, 1H, O8-Bn), 4.70 (d, *J* = 11.5 Hz, 1H, O4-Bn), 4.64 (d, *J* = 10.9 Hz, 1H, O7-Bn), 4.58 (d, *J* = 11.8 Hz, 1H, O8-Bn), 4.51 (s, 2H, O11-Bn), 4.50 (dd, *J* = 10.6, 1.5 Hz, 1H, C6), 4.48 (d, *J* = 11.5 Hz, 1H, O4-Bn), 4.47 (d, *J* = 12.0 Hz, 1H, O9-Bn), 4.42 (d, *J* = 12.0 Hz, 1H, O9-Bn), 4.41 (dd, *J* = 10.3, 3.5 Hz, 1H, C4), 4.34 (ddd, *J* = 10.6, 10.3, 8.6 Hz, 1H, C5), 4.02 (dd, *J* = 10.8, 2.4 Hz, 1H, C9), 3.92 (ddd, *J* = 5.5, 5.3, 2.4 Hz, 1H, C8), 3.91 (d, *J* = 15.0 Hz, 1H, C11), 3.90 (dd, *J* = 5.5, 1.5 Hz, 1H, C7), 3.87 (d, *J* = 15.0 Hz, 1H, C11), 3.80 (dd, *J* = 10.8, 5.3 Hz, 1H, C9), 3.78 (s, 3H, Me); $^{13}C\{^1H\}$ NMR (151 MHz, CD_2Cl_2) δ 170.7 (C10), 165.4 (C1), 142.9 (*ipso*-NPh), 140.8 (q, $^2J_{FC}$ = 36.5 Hz, C=N), [139.3, 139.2, 138.7, 137.7, 137.5] (Bn), 129.3 (*m*-NPh), [129.2, 129.11, 129.05, 128.9, 128.80, 128.76, 128.70, 128.67, 128.63, 128.55, 128.4, 128.1, 128.0, 127.9] (Bn, one signal is missing, possibly due to overlapping), 125.2 (*p*-NPh), 119.8 (*o*-NPh), 116.0 (q, $^1J_{FC}$ = 287.9 Hz, CF_3), 100.7 (C2), 79.3 (C8), 76.2 (C7), 74.8 (O7-Bn), 74.6 (C6), 74.0 (O11-Bn), 73.8 (O9-Bn), 72.8 (C4), 72.5 (O8-Bn), 71.6 (O4-Bn), 70.4 (C9), 70.1 (C11), 53.6 (Me, overlapped with a signal of CD_2Cl_2), 52.6 (C3), 48.4 (C5); ^{19}F NMR (564 MHz, CD_2Cl_2) δ -65.7; ESI-MS found: 1061.2827 calcd: 1061.2806 for $C_{55}H_{54}BrF_3N_2NaO_{10}$ $[M+Na]^+$; IR: 3399, 3065, 3032, 2868, 1783, 1745, 1721, 1688, 1597, 1520, 1497, 1490, 1454, 1328, 1264, 1207, 1163, 1123, 1096, 1074, 1028, 1002, 979, 909, 851, 821, 802, 777, 735, 695.

12: $[\alpha]_D^{25}$ -15.5 (c 1.00, MeCN); ^{1}H NMR (599 MHz, CD_2Cl_2) δ 7.38–7.25 (25H, overlapped, Bn), 6.53 (d, *J* = 9.2 Hz, 1H, NH), 4.78 (d, *J* = 11.9 Hz, 1H, O4-Bn), 4.64 (d, *J* = 11.3 Hz, 1H, O8-Bn), 4.581 (d, *J* = 11.9 Hz, 1H, O4-Bn), 4.580 (s, 2H, O9-Bn), 4.54 (d, *J* = 10.3 Hz, 1H, O7-Bn), 4.53 (d, *J* = 11.3 Hz, 1H, O8-Bn), 4.51 (s, 2H, O11-Bn), 4.47 (d, *J* = 10.3 Hz, 1H, O7-Bn), 4.32 (dd, *J* = 9.7, 2.2 Hz, 1H, C6), 4.07 (ddd, *J* = 9.7, 9.2, 8.2 Hz, 1H, C5), 3.96 (d, *J* = 8.2 Hz, 1H, C4), 3.91 (d, *J* = 15.0 Hz, 1H, C11), 3.92–3.87 (2H, overlapped, C8+C9), 3.86 (d, *J* = 15.0 Hz, 1H, C11), 3.82 (dd, *J* = 7.7, 2.2 Hz, 1H, C7), 3.75 (s, 3H, Me), 3.72 (m, 1H, C9), 3.63 (s, 1H, C3); $^{13}C\{^1H\}$ NMR (151 MHz, CD_2Cl_2) δ 169.7 (C10), 166.2 (C1), [139.0 (2C), 138.8, 137.8, 137.4, 129.1, 129.0, 128.9 (2C), 128.81, 128.78, 128.71, 128.70 (2C), 128.62, 128.61, 128.4, 128.3, 128.11, 128.08] (Bn), 80.5 (C2), 77.7 (C8), 76.3 (C7), 75.7 (C4), 74.9 (O7-Bn), 74.0 (O11-Bn), 73.9 (O9-Bn), 72.9 (O8-Bn), 72.6 (O4-Bn), 71.3 (C6), 70.1 (C11), 69.1(C9), 57.6 (C3), 53.6 (Me), 47.8 (C5); ESI-MS found: 810.3263 calcd: 810.3249 for $C_{47}H_{49}NNaO_{10}$ $[M+Na]^+$; IR: 3384, 3064, 3032, 2865, 1754, 1683, 1606, 1587, 1520, 1497, 1454, 1396, 1344, 1310, 1262, 1199, 1169, 1090, 1074, 1027, 988, 913, 877, 821, 785, 733, 696.

Compound **10**

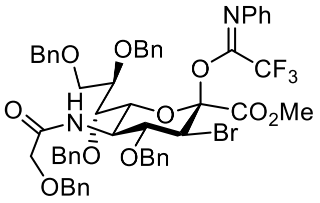

^{i}Pr$_2$NEt (38 μL, 0.22 mmol, 3.0 eq.) and DMAP (3.0 mg, 0.025 mmol, 0.3 eq.) were successively added to a mixture of compound **8** (63.2 mg, 0.0728 mmol) and 2,2,2-trifluoro-*N*-phenylacetimidoyl chloride (30.2 mg, 0.145 mmol, 2.0 eq.) in CH_2Cl_2 (1 mL) at room temperature. After stirring for 1 h, the mixture was concentrated in vacuo. Chromatographic purification (SiO_2, cHex:EtOAc = 9:1 to 85:15) afforded **10** (54.1 mg, 0.0520 mmol, 71%) as a yellow oil.

10: $[\alpha]_D^{25}$ -90.6 (c 1.00, MeCN); ^{1}H NMR (599 MHz, CD_2Cl_2) δ 7.39–7.14 (25H, overlapped, Bn), 7.20 (m, 2H, *m*-NPh), 7.07 (m, 1H, *p*-NPh), 6.72 (m, 2H, *o*-NPh), 6.54 (d, *J* = 8.8 Hz, 1H, NH), 4.92 (d, *J* = 10.4 Hz, 1H, O4-Bn), 4.75 (d, *J* = 10.9 Hz, 1H, O7-Bn), 4.73 (d, *J* = 11.8 Hz, 1H, O8-Bn), 4.59 (d, *J* = 10.9 Hz, 1H, O7-Bn), 4.585 (d, *J* = 10.4 Hz, 1H, O4-Bn), 4.583 (d, *J* = 11.8 Hz, 1H, O8-Bn), 4.50 (s, 2H, O11-Bn), 4.48 (dd, *J* = 10.6, 1.8 Hz, 1H, C6), 4.39 (d, *J* = 11.9 Hz, 1H, O9-Bn), 4.34 (d, *J* = 11.9 Hz, 1H, O9-Bn), 4.32 (dd, *J* = 10.2, 9.9 Hz, 1H, C4), 4.14 (ddd, *J* = 10.6, 9.9, 8.8 Hz, 1H, C5), 4.12 (d, *J* = 10.2 Hz, 1H, C3), 3.96–3.92 (2H, overlapped, C8+C9), 3.90 (dd, *J* = 4.7, 1.8 Hz, 1H, C7), 3.89 (d, *J* = 15.0 Hz, 1H, C11), 3.84 (d, *J* = 15.0 Hz, 1H, C11), 3.82 (s, 3H, Me), 3.77 (dd, *J* = 11.2, 6.6 Hz, 1H, C9); ^{13}C{^{1}H} NMR (151 MHz, CD_2Cl_2) δ 170.3 (C10), 164.4 (C1), 143.6 (*ipso*-NPh), 142.0 (q, $^2J_{FC}$ = 36.3 Hz, C=N), [139.5, 139.2, 138.7, 138.3, 137.4] (Bn), 129.3 (*m*-NPh), [129.2, 129.1, 128.93, 128.89, 128.82, 128.78, 128.7, 128.6, 128.5, 128.4, 128.1, 127.99, 127.96, 127.8] (Bn, one signa lis missing, possibly due to overlapping), 125.1 (*p*-NPh), 119.7 (q, J_{FC} = 1.6 Hz, *o*-NPh), 116.3 (q, $^1J_{FC}$ = 287.5 Hz, CF_3) 101.1 (C2), 79.8 (C8), 79.5 (C4), 76.8 (C7), 75.9 (O4-Bn), 74.9 (O7-Bn), 73.99 (O11-Bn), 73.95 (C6), 73.7 (O9-Bn), 72.4 (O8-Bn), 70.9 (C9), 70.0 (C11), 54.0 (Me), 53.3 (C5), 51.6 (C3); ^{19}F NMR (564 MHz, CD_2Cl_2) δ -65.5; MALDI-TOF MS {matrix = DHB (EtOAc)} found: 1061.39 calcd: 1061.28 for $C_{55}H_{54}BrF_3N_2NaO_{10}$ $[M+Na]^+$; IR: 3396, 3065, 3032, 2906, 2866, 1762, 1715, 1689, 1598, 1518, 1497, 1489, 1454, 1327, 1291, 1266, 1207, 1155, 1119, 1100, 1045, 1027, 941, 911, 847, 805, 776, 734, 694.

^{13}C{^{1}H$_{sel}$} (δ^{1}H$_{sel}$ = 3.82 (OMe)): δ 164.4 (d, $^3J_{CH}$ ≈ 0.9 Hz, C1).

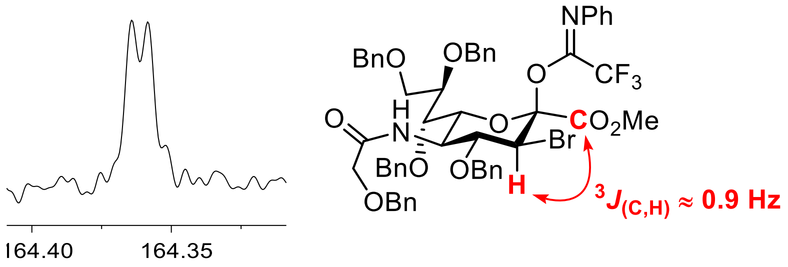

Compound **11**

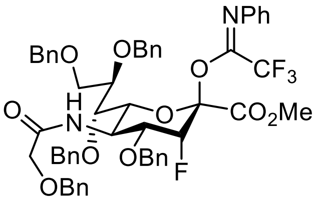

K_2CO_3 (65.0 mg, 0.470 mmol, 3.0 eq.) was added to a solution of compound **5** (127 mg, 0.157 mmol) and 2,2,2-trifluoro-*N*-phenylacetimidoyl chloride (98.2 mg, 0.473 mmol, 3.0 eq.) in acetone (1.2 mL) at room temperature. After stirring for 3 h, the mixture was filtered through a Celite® pad (washed with acetone) and concentrated in vacuo. Chromatographic purification (SiO_2, cHex:EtOAc = 9:1 to 85:15) afforded compound **11** (143 mg, 0.146 mmol, 93%) as a colorless oil.

11: $[\alpha]_D^{25}$ -56.8 (c 1.00, MeCN); ^{1}H NMR (599 MHz, CD_2Cl_2) δ 7.38–7.21 (25H, overlapped, Bn), 7.16 (m, 2H, *m*-NPh), 7.06 (m, 1H, *p*-NPh), 6.65 (m, 2H, *o*-NPh), 6.34 (d, *J* = 8.4 Hz, 1H, NH), 5.07 (dd, $^2J_{FH}$ = 49.0 Hz, *J* = 2.3 Hz, 1H, C3), 4.748 (d, *J* = 10.7 Hz, 1H, O7-Bn), 4.746 (d, *J* = 12.1 Hz, 1H, O8-Bn), 4.72 (d, *J* = 11.6 Hz, 1H, O4-Bn), 4.608 (d, *J* = 10.7 Hz, 1H, O7-Bn), 4.607 (d, *J* = 12.1 Hz, 1H, O8-Bn), 4.58 (d, *J* = 11.6 Hz, 1H, O4-Bn), 4.55 (brd, *J* = 10.8 Hz, 1H, C6), 4.52 (s, 2H, O11-Bn), 4.46 (d, *J* = 11.9 Hz, 1H, O9-Bn), 4.42 (d, *J* = 11.9 Hz, 1H, O9-Bn), 4.41 (ddd, $^3J_{FH}$ = 28.4 Hz, *J* = 10.6, 2.3 Hz, 1H, C4), 4.21 (ddd, *J* = 10.8, 10.6, 8.4 Hz, 1H, C5), 4.03 (dd, *J* = 10.8, 2.0 Hz, 1H, C9), 3.96–3.92 (2H, overlapped, C7+C8), 3.89 (d, *J* = 15.0 Hz, 1H, C11), 3.85 (d, *J* = 15.0 Hz, 1H, C11), 3.80 (dd, *J* = 10.8, 5.5 Hz, 1H, C9), 3.79 (s, 3H, Me); ^{13}C{^{1}H} NMR (151 MHz, CD_2Cl_2) δ 170.7 (C10), 165.3 (C1), 142.9 (*ipso*-NPh), 140.6 (q, $^2J_{FC}$ = 36.7 Hz, C=N), [139.3, 139.2, 138.7, 137.9, 137.5] (Bn), 129.3 (*m*-NPh), [129.13, 129.11, 129.06, 128.9, 128.82, 128.76, 128.7, 128.63 (2C), 128.56, 128.4, 128.1, 128.03, 127.98, 127.9] (Bn), 125.3 (*p*-NPh), 119.7 (q, J_{FC} = 1.5 Hz, *o*-NPh), 116.0 (q, $^1J_{FC}$ = 287.2 Hz, CF_3), 98.5 (d, $^2J_{FC}$ = 29.1 Hz, C2), 87.2 (d, $^1J_{FC}$ = 183.2 Hz, C3), 79.5 (C8), 76.3 (C7), 74.6 (O7-Bn), 74.1 (C6), 74.0 (O11-Bn), 73.80 (d, $^2J_{FC}$ = 17.4 Hz, C4), 73.79 (O9-Bn), 72.7 (O4-Bn), 72.6 (O8-Bn), 70.7 (C9), 70.1 (C11), 53.8 (Me), 47.9 (d, $^3J_{FC}$ = 3.2 Hz, C5); ^{19}F NMR (564 MHz, CD_2Cl_2) δ -65.82 (CF_3), -209.3 (dd, $^2J_{FH}$ = 49.0 Hz, $^3J_{FH}$ = 28.4 Hz, C3-F); ESI-MS found: 1001.3616 calcd: 1001.3607 for $C_{55}H_{54}F_4N_2NaO_{10}$ $[M+Na]^+$; IR: 3400, 3086, 3063, 3032, 2865, 1783, 1745, 1722, 1687, 1597, 1521, 1497, 1454, 1390, 1329, 1286, 1252, 1206, 1164, 1127, 1095, 1072, 1028, 1003, 996, 947, 908, 889, 821, 805, 778, 736, 696.

Preparation scheme for acceptors **13** and **24**

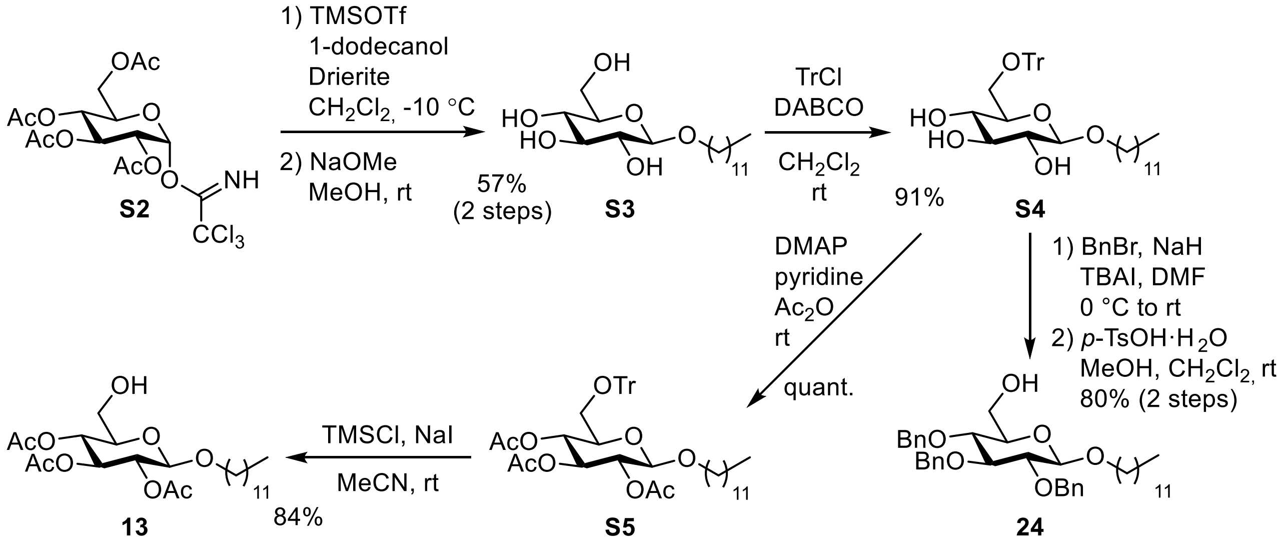

Compound **S3**[4a]

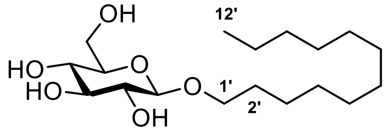

TMSOTf (0.76 mL, 4.13 mmol, 0.2 eq.) was added to a mixture of 2,3,4,6-tetra-*O*-acetyl-α-D-glucopyranosyl trichloroacetimidate (**S2**) (10.2 g, 20.7 mmol), 1-dodecanol (7.71 g, 41.4 mmol, 2.0 eq.) and Drierite™ (5.0 g) in CH_2Cl_2 (100 mL) at -10 °C. After stirring for 2.5 h, the reaction was quenched by adding NEt_3 (3 mL). The mixture was filtered through a Celite® pad (washed with EtOAc) and concentrated in vacuo. Chromatographic purification (SiO_2, cHex:EtOAc = 8:2 to 7:3) followed by chromatographic purification (SiO_2, CH_2Cl_2:EtOAc = 95:5 to 8:2) gave crude material. Subsequently a solution of NaOMe (0.44 M in MeOH, 12.0 mL, 5.28 mmol) was added to a mixture of the obtained crude material in MeOH (85 mL) at room temperature. After stirring 1 h, the mixture was neutralized with Amberlyst® 15 hydrogen form (dry, 8.77 g) at 0 °C. The mixture was filtered through a Celite® pad (washed with MeOH) and concentrated in vacuo. Chromatographic purification (SiO_2, cHex:EtOAc = 1:1 to CH_2Cl_2:MeOH = 85:15) gave compound **S3** (4.11 g, 11.8 mmol, 57%) as a white solid.

S3: $[\alpha]_D^{27}$ -22.7 (c 1.00, MeOH), lit. $[\alpha]_D^{25}$ -24.7 (MeOH),[4b]; ^{1}H NMR (400 MHz, CD_3OD) δ 4.25 (d, *J* = 7.8 Hz, 1H, C1), 3.90 (m, 1H, C1'), 3.87 (dd, *J* = 11.9, 1.9 Hz, 1H, C6), 3.67 (dd, *J* = 11.9, 5.0 Hz, 1H, C6), 3.54 (m, 1H, C1'), 3.35 (dd, *J* = 8.5, 8.4 Hz, 1H, C3), 3.34–3.22 (2H, overlapped, C4+C5), 3.17 (dd, *J* = 8.4, 7.8 Hz, 1H, C2), 1.69–1.56 (2H, overlapped, C2'), 1.52–1.09 (18H, overlapped, dodecyl), 0.91 (m, 3H, C12'); ^{13}C{^{1}H} NMR (101 MHz, CD_3OD) δ 104.3 (C1), 78.1 (C3), 77.9 (C5), 75.1 (C2), 71.6 (C4), 70.9 (C1'), 62.7 (C6), [33.1, 30.80 (2C), 30.75 (3C), 30.6, 30.5, 27.1, 23.7] (dodecyl), 14.4 (C12'); ESI-MS found: 371.2405 calcd: 371.2404 for $C_{18}H_{36}NaO_6$ $[M+Na]^+$; IR: 3320, 2919, 2851, 1673, 1467, 1456, 1416, 1362, 1303, 1286, 1235, 1203, 1159, 1103, 1082, 1066, 1025, 932, 897, 838, 721, 680.

Compound **S4**[5]

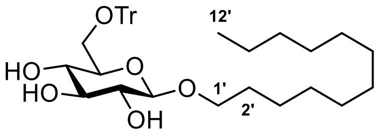

DABCO (430 mg, 3.83 mmol, 2.0 eq.) and trityl chloride (1.06 g, 3.80 mmol, 2.0 eq.) were successively added to a mixture of compound **S3** (664 mg, 1.91 mmol) in CH_2Cl_2 at room temperature. After stirring for 2 h, the reaction was quenched by adding water at 0 ºC. The mixture was extracted with CH_2Cl_2 (×3). The combined organic layer was dried (Na_2SO_4) and concentrated in vacuo. The residue was purified by chromatographic purification (SiO_2, cHex:acetone = 8:2 to 6:4) to afford compound **S4** (1.03 g, 1.74 mmol, 91%) as a white amorphous solid.

S4: $[\alpha]_D^{26}$ -21.7 (c 1.00, acetone); ^1H NMR (599 MHz, CD_2Cl_2) δ 7.48 (m, 6H, *o*-Ph), 7.32 (m, 6H, *m*-Ph), 7.26 (m, 3H, *p*-Ph), 4.28 (d, *J* = 7.7 Hz, 1H, C1), 3.91 (dt, *J* = 9.6, 6.9 Hz, 1H, C1'), 3.57 (dt, *J* = 9.6, 6.9 Hz, 1H, C1'), 3.51 (m, 1H, C4), 3.47 (m, 1H, C3), 3.42 (m, 1H, C5), 3.35 (dd, *J* = 10.0, 3.5 Hz, 1H, C6), 3.35–3.30 (2H, overlapped, C2+C6), 3.14 (m, 1H, C3-OH), 2.84–2.71 (2H, overlapped, C2-OH+C4-OH), 1.69–1.61 (2H, overlapped, C2'), 1.42–1.20 (18H, overlapped, dodecyl), 0.89 (t, *J* = 7.0 Hz, 3H, C12'); $^{13}C\{^1H\}$ NMR (151 MHz, CD_2Cl_2) δ 144.5 (*ipso*-Ph), 129.2 (*o*-Ph), 128.5 (*m*-Ph), 127.7 (*p*-Ph), 103.2 (C1), 87.3 (CPh_3), 77.1 (C3), 75.0 (C5), 74.4 (C2), 71.9 (C4), 70.6 (C1'), 64.4 (C6), [32.5, 30.33, 30.25, 30.22, 30.21, 30.20, 30.1, 29.9, 26.7, 23.3] (dodecyl), 14.5 (C12'); ESI-MS found: 613.3501 calcd: 613.3500 for $C_{37}H_{50}NaO_6$ $[M+Na]^+$; IR: 3388, 3063, 2924, 2854, 1705, 1598, 1490, 1449, 1376, 1317, 1220, 1160, 1047, 922, 899, 764, 745, 700.

Compound **S5**

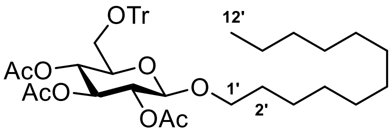

DMAP (20.3 mg, 0.166 mmol, 4 mol%) was added to a mixture of compound **S4** (2.40 g, 4.06 mmol) in Ac_2O (2 mL) and pyridine (8 mL) at room temperature. After stirring for 1 h, the reaction was quenched by adding saturated aqueous $NaHCO_3$ at 0 °C. The mixture was extracted with EtOAc (×3). The combined organic layer was washed with saturated aqueous $NaHCO_3$ and brine successively, dried (Na_2SO_4) and concentrated in vacuo. The residue was azeotropically distilled with toluene (×3) for removing pyridine. Chromatographic purification (SiO_2, cHex:EtOAc = 85:15 to 8:2) afforded **S5** (2.90 g, 4.05 mmol, quant.) as a white amorphous.

S5: $[\alpha]_D^{26}$ +23.9 (c 1.00, acetone); ^1H NMR (500 MHz, CD_2Cl_2) δ 7.47 (m, 6H, *o*-Ph), 7.31 (m, 6H, *m*-Ph), 7.25 (m, 3H, *p*-Ph), 5.20–5.10 (2H, overlapped, C3+C4), 4.99 (m, 1H, C2), 4.53 (d, *J* = 8.0 Hz, 1H, C1), 3.93 (dt, *J* = 9.7, 6.5 Hz, 1H, C1'), 3.61–3.54 (2H, overlapped, C5+C1'), 3.27 (dd, *J* = 10.5, 2.2 Hz, 1H, C6), 3.04 (dd, *J* = 10.5, 4.6 Hz, 1H, C6), 2.03 (s, 3H, Ac), 1.96 (s, 3H, Ac), 1.73 (s, 3H, Ac), 1.69–1.58 (2H, overlapped, C2'), 1.43–1.21 (18H, overlapped, dodecyl), 0.89 (t, *J* = 7.0 Hz, 3H, C12'); $^{13}C\{^1H\}$ NMR (126 MHz, CD_2Cl_2) δ [170.7, 169.8, 169.5] (Ac), 144.4 (*ipso*-Ph), 129.2 (*o*-Ph), 128.4 (*m*-Ph), 127.6 (*p*-Ph), 101.5 (C1), 87.1 (CPh_3), 73.8 (C5), 73.7 (C3), 72.1 (C2), 70.6 (C1'), 69.3 (C4), 62.5 (C6), [32.5, 30.3, 30.24, 30.22, 30.21, 30.16, 30.0, 29.9, 26.6, 23.3] (dodecyl), [21.1, 21.0, 20.8] (Ac), 14.5 (C12'); ESI-MS found: 739.3819 calcd: 739.3817 for $C_{43}H_{56}NaO_9$ $[M+Na]^+$; IR: 3058, 3032, 2925, 2854, 1756, 1598, 1491, 1449, 1366, 1318, 1243, 1214, 1169, 1033, 1003, 983, 945, 926, 900, 765, 746, 703, 658.

Compound **13**[6]

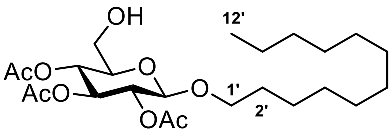

TMSCl (0.69 mL, 5.46 mmol, 1.5 eq.) was added to a mixture of compound **S5** (2.60 g, 3.63 mmol) and NaI (819 mg, 5.46 mmol, 1.5 eq.) in MeCN (35 mL) at room temperature. After stirring for 30 min, additional NaI (544 mg, 3.62 mmol, 1.0 eq.) and TMSCl (0.46mL, 3.64 mmol, 1.0 eq.) was successively added to the mixture. After further stirring for 30 min, water was added to the mixture at 0 ºC. After stirring for 20 min, the mixture was quenched by adding saturated aqueous $NaHCO_3$ and saturated aqueous $Na_2S_2O_3$ at 0 ºC. The mixture was extracted with CH_2Cl_2 (×3). The combined organic layer was dried (Na_2SO_4) and concentrated in vacuo. Chromatographic purification (SiO_2, CH_2Cl_2:Et_2O = 95:5 to 9:1) afforded compound **13** (1.45 g, 3.06 mmol, 84%) as a white solid.

13: mp 53-55 ºC; $[\alpha]_D^{27}$ -11.9 (c 1.00, acetone) ^{1}H NMR (500 MHz, CD_2Cl_2) δ 5.23 (dd, *J* = 9.8, 9.6 Hz, 1H, C3), 4.99 (dd, *J* = 9.7, 9.6 Hz, 1H, C4), 4.88 (dd, *J* = 9.8, 8.0 Hz, 1H, C2), 4.52 (d, *J* = 8.0 Hz, 1H, C1), 3.85 (dt, *J* = 9.7, 6.4 Hz, 1H, C1'), 3.71 (ddd, *J* = 12.5, 7.9, 2.4 Hz, 1H, C6), 3.57 (ddd, *J* = 12.5, 5.9, 4.6 Hz, 1H, C6), 3.51 (ddd, *J* = 9.7, 4.6, 2.4 Hz, 1H, C5), 3.48 (dt, *J* = 9.7, 6.8 Hz, 1H, C1'), 2.25 (dd, *J* = 7.9, 5.9 Hz, 1H, C6-OH), 2.03 (s, 3H, Ac), 2.01 (s, 3H, Ac), 1.98 (s, 3H, Ac), 1.60–1.48 (2H, overlapped, C2'), 1.35–1.20 (18H, overlapped, dodecyl), 0.88 (t, *J* = 7.0 Hz, 3H, C12'); ^{13}C{^{1}H} NMR (126 MHz, CD_2Cl_2) δ [170.7, 170.6, 169.8] (Ac), 101.4 (C1), 74.6 (C5), 73.2 (C3), 72.0 (C2), 70.8 (C1'), 69.3 (C4), 61.7 (C6), [32.5, 30.24, 30.21, 30.19 (2C), 30.0, 29.92, 29.91, 26.4, 23.3] (dodecyl), [21.04, 21.01, 21.00] (Ac), 14.5 (C12'); ESI-MS found: 497.2722 calcd: 497.2721 for $C_{24}H_{42}NaO_9$ $[M+Na]^+$; IR: 3556, 3442, 2920, 2852, 1740, 1468, 1427, 1369, 1256, 1231, 1163, 1127, 1084, 1057, 1038, 985, 911, 879, 720, 689.

Compound **24**

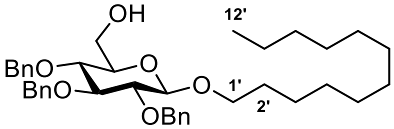

NaH (477 mg, 60% dispersion in mineral oil, 11.9 mmol, 4.0 eq.) was added to a mixture of compound **S4** (1.76 g, 2.98 mmol) in DMF (45 mL) at 0 °C. After stirring for 10 min, BnBr (1.42 mL, 12.0 mmol, 4.0 eq.) and TBAI (110 mg, 0.298 mmol, 0.1 eq.) were successively added. After stirring at 0 °C for 20 min, the mixture was warm up to 15 °C for 1 h and then to room temperature for 3.5 h. Additional NaH (359 mg, 60% dispersion in mineral oil, 8.98 mmol, 3.0 eq) and BnBr (1.06 mL, 8.92 mmol, 3.0 eq.) were successively added at 0 °C. After stirring at room temperature for 2 h, additional NaH (362 mg, 60% dispersion in mineral oil, 9.05 mmol, 3.0 eq) and BnBr (1.06 mL, 8.92 mmol, 3.0 eq.) were successively added at 0 °C. After stirring at room temperature for 15 h, the reaction was quenched by adding water at 0 ºC. The mixture was extracted with Et_2O (×3). The combined organic layer was washed with water and brine, dried (Na_2SO_4) and concentrated in vacuo. Then *p*-TsOH·H_2O (108 mg, 0.568 mmol, 0.2 eq.) was added to a solution of the obtained crude material in MeOH/CH_2Cl_2 (2/1, 60 mL) at room temperature. After stirring for 17 h, the reaction was quenched by adding saturated aqueous $NaHCO_3$ at 0 ºC. The mixture was extracted with CH_2Cl_2 (×3). The combined organic layer was dried (Na_2SO_4) and concentrated in vacuo. Chromatographic purification (SiO_2, cHex:EtOAc = 9:1 to 85:15) gave compound **24** (1.47 g, 2.38 mmol, 80%) as a white solid.

24: mp 66-67 ºC; $[\alpha]_D^{25}$ +3.2 (c 1.00, $CHCl_3$); ^{1}H NMR (599 MHz, $CDCl_3$) δ 7.37–7.27 (15H, overlapped, Bn), 4.96 (d, *J* = 10.9 Hz, 1H, O2-Bn), 4.95 (d, *J* = 10.9 Hz, 1H, O3-Bn), 4.87 (d, *J* = 10.9 Hz, 1H, O4-Bn), 4.82 (d, *J* = 10.9 Hz, 1H, O3-Bn), 4.73 (d, *J* = 10.9 Hz, 1H, O2-Bn), 4.65 (d, *J* = 10.9 Hz, 1H, O4-Bn), 4.45 (d, *J* = 7.8 Hz, 1H, C1), 3.93 (dt, *J* = 9.5, 6.5 Hz, 1H, C1'), 3.88 (brdd, *J* = 11.2, 2.8 Hz, 1H, C6), 3.72 (brd, *J* = 11.2 Hz, 1H, C6), 3.68 (dd, *J* = 9.2, 9.1 Hz, 1H, C3), 3.57 (dd, *J* = 9.4, 9.1 Hz, 1H, C4), 3.55 (dt, *J* = 9.5, 6.9 Hz, 1H, C1'), 3.43 (dd, *J* = 9.2, 7.8 Hz, 1H, C2), 3.37 (ddd, *J* = 9.4, 4.7, 2.8 Hz, 1H, C5), 1.94 (brs, 1H, C6-OH), 1.72–1.56 (2H, overlapped, C2'), 1.47–1.21 (18H, overlapped, dodecyl), 0.89 (t, *J* = 7.0 Hz, 3H, C12'); ^{13}C{^{1}H} NMR (151 MHz, $CDCl_3$) δ [138.7, 138.6, 138.1, 128.6, 128.52, 128.50, 128.24, 128.22, 128.1, 128.0, 127.82, 127.76] (Bn), 103.9 (C1), 84.7 (C3), 82.5 (C2), 77.8 (C4), 75.8 (O3-Bn), 75.2 (O4-Bn), 75.1 (C5), 75.0 (O2-Bn), 70.6 (C1'), 62.3 (C6), [32.1, 29.9, 29.81, 29.77, 29.76, 29.7, 29.6, 29.5, 26.3, 22.8] (dodecyl), 14.3 (C12'); ESI-MS found: 641.3824 calcd: 641.3813 for $C_{39}H_{54}NaO_6$ $[M+Na]^+$; IR: 3463, 3064, 3033, 2921, 2852, 1497, 1465, 1454, 1399, 1359, 1305, 1276, 1209, 1171, 1146, 1071, 1030, 1016, 1005, 911, 896, 829, 753, 734, 696, 668.

Compound **14**

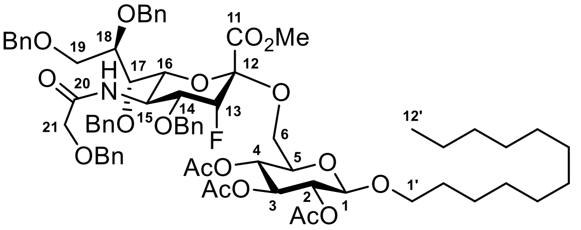

A solution of TMSOTf (0.10 M in CH_2Cl_2, 1.30 mL, 0.130 mmol, 1.1 eq.) was added to a mixture of compound **11** (116 mg, 0.118 mmol), compound **13** (113 mg, 0.238 mmol, 2.0 eq.) and Drierite™ (139 mg) in CH_2Cl_2 (2.8 mL) at 0 °C. After stirring for 1 h, the reaction was quenched by adding NEt_3 (0.2 mL). The mixture was filtered through a Celite® pad (washed with EtOAc) and concentrated in vacuo. Chromatographic purification (SiO_2, cHex:EtOAc = 8:2 to 7:3) followed by chromatographic purification (SiO_2, CH_2Cl_2:Et_2O = 95:5 to 8:2) gave compound **14** (91.0 mg, 0.072 mmol, 61%) as a colorless oil.

14: ^{1}H NMR (599 MHz, $CDCl_3$) δ 7.40–7.18 (25H, overlapped, Bn), 6.09 (d, *J* = 8.3 Hz, 1H, NH), 5.14 (dd, *J* = 9.7, 9.5 Hz, 1H, C3), 5.04 (dd, $^2J_{FH}$ = 51.4 Hz, *J* = 1.7 Hz, 1H, C13), 5.03 (dd, *J* = 9.6, 9.5 Hz, 1H, C4), 4.90 (dd, *J* = 9.7, 8.0 Hz, 1H, C2), 4.75 (d, *J* = 11.3 Hz, 1H, O18-Bn), 4.70 (d, *J* = 11.3 Hz, 1H, O18-Bn), 4.64 (d, *J* = 12.0 Hz, 1H, O14-Bn), 4.64 (d, *J* = 11.4 Hz, 1H, O17-Bn), 4.62 (d, *J* = 11.4 Hz, 1H, O17-Bn), 4.56 (d, *J* = 12.2 Hz, 1H, O19-Bn), 4.54 (d, *J* = 12.2 Hz, 1H, O19-Bn), 4.51 (d, *J* = 12.0 Hz, 1H, O14-Bn), 4.46 (s, 2H, O21-Bn), 4.35 (d, *J* = 8.0 Hz, 1H, C1), 4.32 (brd, *J* = 10.3 Hz, 1H, C16), 4.07 (m, 1H, C15), 4.03–3.94 (3H, overlapped, C6+C14+C18), 3.85 (dd, *J* = 10.7, 2.3 Hz, 1H, C19), 3.83 (d, *J* = 15.0 Hz, 1H, C21), 3.81–3.73 (3H, overlapped, C6+C17+C1'), 3.75 (d, *J* = 15.0 Hz, 1H, C21), 3.70 (dd, *J* = 10.7, 4.4 Hz, 1H, C19), 3.61 (s, 3H, Me), 3.58 (dt, *J* = 9.6, 4.1 Hz, 1H, C5), 3.36 (dt, *J* = 9.6, 6.7 Hz, 1H, C1'), 2.02 (s, 3H, Ac), 1.99 (s, 3H, Ac), 1.87 (s, 3H, Ac), 1.54–1.17 (20H, overlapped, dodecyl), 0.88 (t, *J* = 7.0 Hz, 3H, C12'); ^{13}C{^{1}H} NMR (151 MHz, $CDCl_3$) δ 170.5 (Ac), 170.2 (C20), [169.7, 169.5] (Ac), 166.3 (d, $^3J_{FC}$ = 3.9 Hz, C11) [139.0, 138.4, 138.3, 137.6, 136.8, 128.8, 128.6, 128.51, 128.50, 128.39, 128.37, 128.23, 128.15, 128.1, 127.97, 127.96, 127.9, 127.8, 127.6] (Bn, one signal is missing, possibly due to overlapping), 100.9 (C1), 97.7 (d, $^2J_{FC}$ = 16.4 Hz, C12), 86.5 (d, $^1J_{FC}$ = 190.5 Hz, C13), 78.1 (C18), 75.6 (C17), 74.0 (O17-Bn), 73.9 (d, $^2J_{FC}$ = 17.7 Hz, C14), 73.54 (O19-Bn), 73.51 (O21-Bn), 73.1 (C3), 72.7 (O18-Bn), 72.5 (C5), 72.4 (C16), 71.6 (C2), 71.5 (O14-Bn), 70.2 (C1'), 70.0 (C4), 69.6 (C21), 69.4 (C19), 64.3 (C6), 52.8 (Me), 47.7 (d, $^3J_{FC}$ = 3.3 Hz, C15), [32.1, 29.82, 29.77, 29.75, 29.50, 29.49, 29.46, 26.0, 22.8] (dodecyl, one signal is missing possibly due to overlapping), [20.81, 20.80, 20.7] (Ac), 14.3 (C12'); ^{19}F NMR (564 MHz, $CDCl_3$) δ -217.9 (dd, $^2J_{FH}$ = 51.4 Hz, $^3J_{FH}$ = 27.8 Hz); NSI-FTMS found: 1286.6035 calcd: 1286.6034 for $C_{71}H_{90}FNNaO_{18}$ $[M+Na]^+$; IR:

3398, 3089, 3063, 3031, 2924, 2854, 1754, 1683, 1523, 1497, 1454, 1436, 1366, 1333, 1304, 1246, 1217, 1163, 1089, 1067, 1037, 908, 810, 751, 698, 667.

Compound **15**

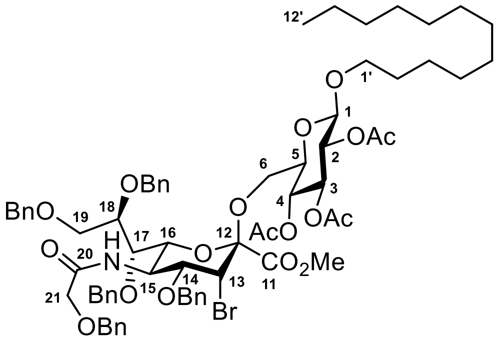

A solution of TMSOTf (0.10 M in CH_2Cl_2, 1.13 mL, 0.113 mmol, 1.1 eq.) was added to a mixture of compound **9** (107 mg, 0.103 mmol), **13** (97.5 mg, 0.205 mmol, 2.0 eq.) and Drierite™ (125 mg) in CH_2Cl_2 (2.5 mL) at 0 °C. After stirring for 1 h, the reaction was quenched by adding NEt_3 (0.2 mL). The mixture was filtered through a Celite® pad (washed with EtOAc) and concentrated in vacuo. Chromatographic purification (SiO_2, cHex:EtOAc = 9:1 to 75:25) followed by chromatographic purification (SiO_2, cHex:EtOAc = 6:4) afforded compound **15** (120 mg, 0.0905 mmol, 88%) as a white solid.

15: mp 92-97 ºC; $[\alpha]_D^{25}$ +20.5 (c 1.00, $CHCl_3$); ^1H NMR (599 MHz, $CDCl_3$) δ 7.40–7.16 (25H, overlapped, Bn), 6.18 (d, *J* = 9.2 Hz, 1H, NH), 5.05 (dd, *J* = 9.6, 9.5 Hz, 1H, C3), 4.95 (dd, *J* = 9.7, 9.5 Hz, 1H, C4), 4.84 (dd, *J* = 9.6, 8.1 Hz, 1H, C2), 4.78 (d, *J* = 11.9 Hz, 1H, O18-Bn), 4.75 (d, *J* = 11.0 Hz, 1H, O17-Bn), 4.73 (d, *J* = 12.0 Hz, 1H, O14-Bn), 4.70 (d, *J* = 3.5 Hz, 1H, C13), 4.62 (d, *J* = 11.0 Hz, 1H, O17-Bn), 4.55 (d, *J* = 11.9 Hz, 1H, O18-Bn), 4.52–4.44 (1H, overlapped, C15), 4.50 (s, 2H, O19-Bn), 4.47 (d, *J* = 12.1 Hz, 1H, O21-Bn), 4.44 (d, *J* = 12.1 Hz, 1H, O21-Bn), 4.41 (d, *J* = 12.0 Hz, 1H, O14-Bn), 4.24 (dd, *J* = 10.6, 1.8 Hz, 1H, C16), 4.23 (d, *J* = 8.1 Hz, 1H, C1), 4.11 (dd, *J* = 10.1, 3.5 Hz, 1H, C14), 4.00 (ddd, *J* = 5.9, 4.3, 2.9 Hz, 1H, C18), 3.96 (dd, *J* = 10.8, 2.9 Hz, 1H, C19), 3.85 (dd, *J* = 5.9, 1.8 Hz, 1H, C17), 3.84 (d, *J* = 14.9 Hz, 1H, C21), 3.81 (d, *J* = 14.9 Hz, 1H, C21), 3.78 (s, 3H, Me), 3.72 (dd, *J* = 10.8, 4.3 Hz, 1H, C19), 3.71 (dd, *J* = 10.4, 2.1 Hz, 1H, C6), 3.67 (dt, *J* = 9.9, 6.3 Hz, 1H, C1'), 3.28 (dt, *J* = 9.9, 6.7 Hz, 1H, C1'), 3.22 (ddd, *J* = 9.7, 4.3, 2.1 Hz, 1H, C5), 3.16 (dd, *J* = 10.4, 4.3 Hz, 1H, C6), 2.04 (s, 3H, Ac), 2.03 (s, 3H, Ac), 1.92 (s, 3H, Ac), 1.47–1.14 (20H, overlapped, dodecyl), 0.88 (t, *J* = 7.0 Hz, 3H, C12'); $^{13}C\{^1H\}$ NMR (151 MHz, $CDCl_3$) δ 170.5 (Ac), 170.1 (C20), [169.3, 169.2] (Ac), 166.1 (C11), [139.1, 138.6, 138.5, 137.3, 137.0, 128.8, 128.7, 128.50, 128.48, 128.42, 128.39, 128.34, 128.26, 128.2, 128.0, 127.8, 127.7, 127.61, 127.55, 127.5] (Bn), 100.6 (C1), 99.8 (C12), 79.2 (C18), 75.8 (C17), 74.0 (O17-Bn), 73.5 (O21-Bn), 73.41 (C3), 73.37 (O19-Bn), 72.6 (C14), 72.0 (O18-Bn), 72.0 (C5), 71.9 (C16), 71.5 (C2), 70.1 (O14-Bn), 69.9 (C1'), 69.54 (C19), 69.51 (C21), 68.3 (C4), 62.2 (C6), 52.8 (Me), 51.5 (C13), 47.5 (C15), [32.1, 29.84, 29.80 (2C), 29.79, 29.6, 29.49, 29.47, 26.0, 22.8] (dodecyl), [20.82, 20.80, 20.7] (Ac), 14.3 (C12'); NSI-FTMS found: 1324.5397 calcd: 1324.5414 for $C_{71}H_{91}BrNO_{18}$ $[M+H]^+$; IR: 3402, 3065, 3031, 2925, 2854, 1755, 1682, 1605, 1586, 1522, 1497, 1455, 1435, 1367, 1337, 1309, 1245, 1217, 1161, 1094, 1072, 1036, 1029, 909, 888, 807, 751, 698, 667.

Compounds **16** (α-anomer) and **17** (β-anomer)

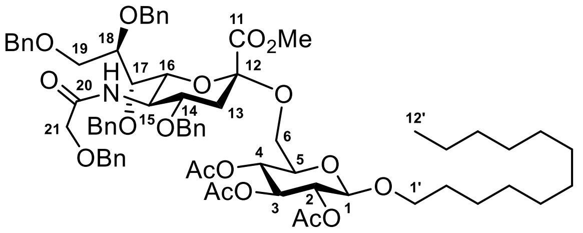

16

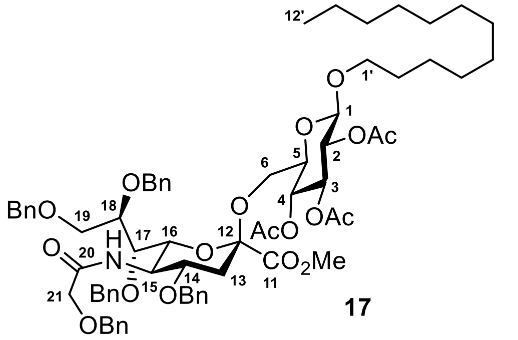

17

A solution of TMSOTf (0.10 M in CH_2Cl_2, 0.57 mL, 0.057 mmol, 1.1 eq.) was added to a mixture of compound **10** (54.1 mg, 0.0520 mmol), compound **13** (49.5 mg, 0.104 mmol, 2.0 eq.) and Drierite™ (70 mg) in CH_2Cl_2 (1.3 mL) at 0 °C. After stirring for 1 h, the reaction was quenched by adding NEt_3 (0.1 mL). The mixture was filtered through a Celite® pad (washed with EtOAc) and concentrated in vacuo. Chromatographic purification (SiO_2, cHex:EtOAc = 85:15 to 75:25) afforded crude material. The mixture of the obtained crude material, Bu_3SnH (97%, 0.030 mL, 0.11 mmol, 4.6 eq.) and AIBN (1.1 mg, 6.7 μmol, 0.3 eq.) in toluene (1 mL) was refluxed for 2 h. After cooling to room temperature, the mixture was purified by chromatographic purification (SiO_2, cHex:EtOAc = 9:1 to 1:1) to afford crude material. Further chromatographic purification [SiO_2/K_2CO_3 (v/v = 9/1),[7] cHex:EtOAc = 75:25 to 7:3] followed by chromatographic purification (SiO_2, cHex:EtOAc = 75:25) gave compound **16** (2.9 mg, 2.3 μmol, ca.4%) and compound **17** (19.3 mg, 0.0155 mmol, ca.30%), both as a colorless oil.

16: ^{1}H NMR (500 MHz, $CDCl_3$) δ 7.41–7.20 (25H, overlapped, Bn), 6.19 (d, *J* = 9.1 Hz, 1H, NH), 5.12 (dd, *J* = 9.5, 9.4 Hz, 1H, C3), 5.05 (dd, *J* = 9.6, 9.5 Hz, 1H, C4), 4.91 (dd, *J* = 9.4, 8.0 Hz, 1H, C2), 4.73 (d, *J* = 11.4 Hz, 1H, O18-Bn), 4.69 (d, *J* = 11.4 Hz, 1H, O18-Bn), 4.65 (d, *J* = 10.7 Hz, 1H, O17-Bn), 4.62 (d, *J* = 12.0 Hz, 1H, O14-Bn), 4.56 (d, *J* = 10.7 Hz, 1H, O17-Bn), 4.56 (d, *J* = 12.2 Hz, 1H, O19-Bn), 4.53 (d, *J* = 12.2 Hz, 1H, O19-Bn), 4.52 (d, *J* = 11.7 Hz, 1H, O21-Bn), 4.48 (d, *J* = 11.7 Hz, 1H, O21-Bn), 4.42 (d, *J* = 12.0 Hz, 1H, O14-Bn), 4.36 (d, *J* = 8.0 Hz, 1H, C1), 4.09 (ddd, *J* = 10.6, 9.7, 9.1 Hz, 1H, C15), 4.03 (dd, *J* = 10.6, 1.4 Hz, 1H, C16), 3.95–3.85 (2H, overlapped, C6+C18), 3.94 (d, *J* = 15.0 Hz, 1H, C21), 3.89 (d, *J* = 15.0 Hz, 1H, C21), 3.85 (dd, *J* = 10.6, 2.3 Hz, 1H, C19), 3.83–3.76 (1H, overlapped, C1'), 3.76 (dd, *J* = 7.5, 1.4 Hz, 1H, C17), 3.71 (dd, *J* = 10.6, 4.4 Hz, 1H, C19), 3.63 (s, 3H, Me), 3.60 (dd, *J* = 11.1, 3.4 Hz, 1H, C6), 3.59–3.49 (1H, overlapped, C14), 3.51 (ddd, *J* = 9.6, 3.9, 3.4 Hz, 1H, C5), 3.38 (dt, *J* = 9.6, 6.8 Hz, 1H, C1'), 2.73 (dd, *J* = 12.6, 4.4 Hz, 1H, C13), 2.02 (s, 3H, Ac), 1.98 (s, 3H, Ac), 1.77 (s, 3H, Ac), 1.71 (dd, *J* = 12.6, 12.2 Hz, 1H, C13), 1.56–1.19 (20H, overlapped, dodecyl), 0.88 (t, *J* = 6.9 Hz, 3H, C12'); ^{13}C{^{1}H} NMR (126 MHz, $CDCl_3$) δ 170.5 (Ac), 170.0 (C20), [169.6, 169.4] (Ac), 168.2 (C11), [139.1, 138.44, 138.41, 138.1, 136.8, 128.8, 128.7, 128.5 (2C), 128.40, 128.38, 128.37, 128.2, 128.02, 127.99, 127.91, 127.87, 127.8, 127.7, 127.5] (Bn), 100.9 (C1), 98.8 (C12), 78.4 (C18), 76.2 (C17), 74.64 (O17-Bn), 74.58 (C14), 73.64 (O21-Bn), 73.57 (C16), 73.5 (O19-Bn), 73.2 (C3), [72.64, 72.61] (C5+O18-Bn), 71.7 (C2), 70.8 (O14-Bn), 70.2 (C1'), 69.7 (C21), 69.5 (C19), 69.3 (C4), 63.2 (C6), 52.5 (Me), 50.8 (C15), 37.1 (C13), [32.1, 29.81, 29.77, 29.76, 29.50, 29.49, 26.0, 22.8] (dodecyl, 2 signals are missing, possibly due to overlapping), [20.81, 20.80, 20.6] (Ac), 14.3 (C12'); NSI-FTMS found: 1246.6313 calcd: 1246.6309 for $C_{71}H_{92}NO_{18}$ $[M+H]^+$;

$^{13}C\{^1H_{sel}\}$ (δ^1H_{sel} = 3.63 (OMe)): δ 168.2 (d, $^3J_{CH}$ ≈ 6.5 Hz, C11).

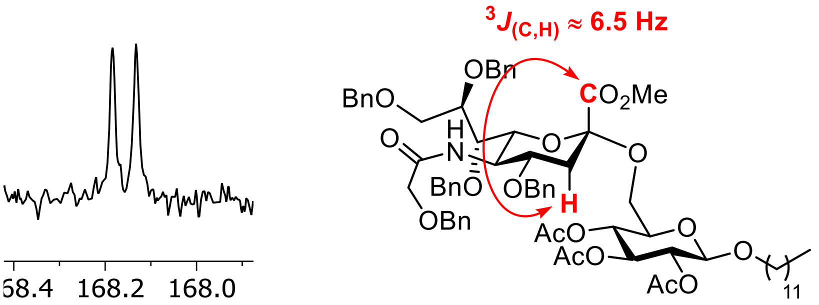

*Conversion of compound **15** to compound **17***

A mixture of compound **15** (94.6 mg, 0.0714 mmol), Bu_3SnH (97%, 0.050 mL, 0.19 mmol, 2.6 eq.) and AIBN (3.1 mg, 0.019 mmol, 0.3 eq.) in toluene (1.5 mL) was refluxed for 1.5 h. After cooling to room temperature, the mixture was purified by chromatographic purification (SiO_2, cHex:EtOAc = 9:1 to 1:1) to afford crude material. Further chromatographic purification [SiO_2/K_2CO_3 (v/v = 9/1),[7] cHex:EtOAc = 7:3] gave compound **17** (77.7 mg, 0.0623 mmol, 87%) as a colorless oil.

17: $[\alpha]_D^{25}$ -1.0 (c 1.00, $CHCl_3$); ^{1}H NMR (599 MHz, $CDCl_3$) δ 7.38–7.20 (25H, overlapped, Bn), 6.33 (d, *J* = 9.7 Hz, 1H, NH), 5.07 (dd, *J* = 9.6, 9.5 Hz, 1H, C3), 5.00 (dd, *J* = 9.7, 9.5 Hz, 1H, C4), 4.84 (dd, *J* = 9.6, 8.1 Hz, 1H, C2), 4.77 (d, *J* = 11.8 Hz, 1H, O18-Bn), 4.74 (d, *J* = 10.7 Hz, 1H, O17-Bn), 4.65 (d, *J* = 11.9 Hz, 1H, O14-Bn), 4.57 (d, *J* = 10.7 Hz, 1H, O17-Bn), 4.53 (d, *J* = 11.8 Hz, 1H, O18-Bn), 4.50 (s, 4H, O19-Bn+O21-Bn), 4.43 (d, *J* = 11.9 Hz, 1H, O14-Bn), 4.29 (ddd, *J* = 10.6, 9.8, 9.7 Hz, 1H, C15), 4.21 (d, *J* = 8.1 Hz, 1H, C1), 4.13 (dd, *J* = 10.6, 1.9 Hz, 1H, C16), 3.96 (dd, *J* = 10.7, 2.6 Hz, 1H, C19), 3.93 (m, 1H, C18), 3.91 (d, *J* = 15.2 Hz, 1H, C21), 3.88 (d, *J* = 15.2 Hz, 1H, C21), 3.87 (dd, *J* = 6.3, 1.9 Hz, 1H, C17), 3.84 (ddd, *J* = 11.1, 9.8, 4.6 Hz, 1H, C14), 3.72 (dd, *J* = 10.7, 4.3 Hz, 1H, C19), 3.72–3.66 (1H, overlapped, C1'), 3.71 (s, 3H, Me), 3.69 (dd, *J* = 10.8, 2.8 Hz, 1H, C6), 3.49 (dd, *J* = 10.8, 3.9 Hz, 1H, C6), 3.27 (dt, *J* = 10.0, 6.7 Hz, 1H, C1'), 3.23 (ddd, *J* = 9.7, 3.9, 2.8 Hz, 1H, C5), 2.53 (dd, *J* = 12.9, 4.6 Hz, 1H, C13), 2.030 (s, 3H, Ac), 2.027 (s, 3H, Ac), 1.92 (s, 3H, Ac), 1.78 (dd, *J* = 12.9, 11.1 Hz, 1H, C13), 1.48–1.14 (20H, overlapped, dodecyl), 0.88 (t, *J* = 7.0 Hz, 3H, C12'); $^{13}C\{^1H\}$ NMR (151 MHz, $CDCl_3$) δ 170.5 (Ac), 170.1, (C20) [169.42, 169.35] (Ac), 167.9 (C11), [139.1, 138.6, 138.5, 138.2, 136.9, 128.7, 128.63, 128.60, 128.5, 128.4, 128.3, 128.2, 128.1, 128.0, 127.9, 127.8, 127.7, 127.6, 127.54, 127.52] (Bn), 100.6 (C1), 98.5 (C12), 79.2 (C18), 76.2 (C17), 74.4 (O17-Bn), 74.1 (C14), 73.52 (O21-Bn), 73.47 (C3), 73.4 (O19-Bn), 72.21 (C5), 72.20 (C16), 71.8 (O18-Bn), 71.6 (C2), 70.6 (O14-Bn), 69.9 (C1'), 69.5 (C21), 69.4 (C19), 68.7 (C4), 61.2 (C6), 52.5 (Me), 50.2 (C15), 36.5 (C13), [32.1, 29.83, 29.79 (2C), 29.78, 29.6, 29.49, 29.46, 26.0, 22.8] (dodecyl), [20.83, 20.80, 20.77] (Ac), 14.3 (C12'); ESI-MS found: 1268.6149 calcd: 1268.6128 for $C_{71}H_{91}NNaO_{18}$ $[M+Na]^+$; IR: 3392, 3062, 3030, 2924, 2855, 1756, 1688, 1606, 1587, 1522, 1497, 1455, 1366, 1319, 1245, 1217, 1175, 1093, 1077, 1044, 985, 908, 884, 846, 821, 785, 751, 698, 666.

$^{13}C\{^1H_{sel}\}$ (δ^1H_{sel} = 3.71 (OMe)): δ 167.9 (d, $^3J_{CH}$ ≈ 1.1 Hz, C11).

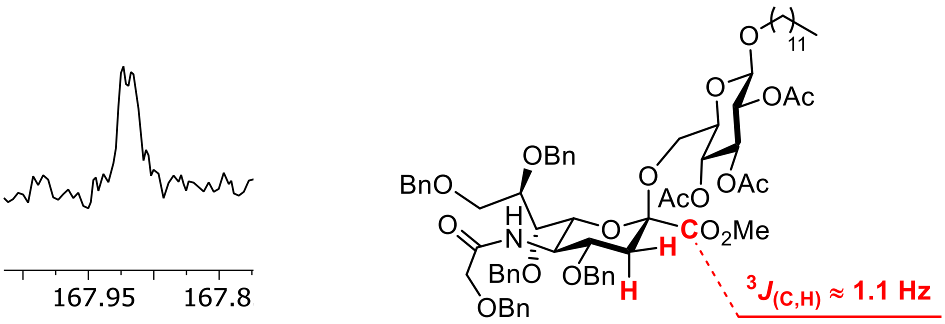

Compound **18**

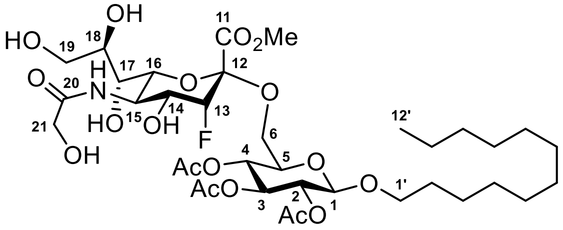

A flask, thoroughly purged with argon, was charged with 20% $Pd(OH)_2$ (18.6 mg). Then a solution of compound **14** (95.0 mg, 0.0751 mmol) in MeOH (7 mL) was added. Then the Ar atmosphere was replaced by H_2 gas (1 atm) and the mixture was stirred for 22 h at room temperature. After changing the atmosphere from H_2 back to argon, the mixture was filtered through a Celite® pad (washed with MeOH) and concentrated in vacuo. Chromatographic purification (SiO_2, CH_2Cl_2:MeOH = 9:1) followed by chromatographic purification (SiO_2, CH_2Cl_2:EtOH = 9:1 to 85:15) afforded compound **18** (49.2 mg, 0.0605 mmol, 81%) as a colorless oil.

18: $[\alpha]_D^{26}$ -4.2 (c 1.00, MeOH); ^{1}H NMR (599 MHz, CD_3OD) δ 5.23 (dd, *J* = 9.7, 9.5 Hz, 1H, C3), 5.05 (dd, *J* = 10.1, 9.5 Hz, 1H, C4), 5.01 (dd, $^2J_{FH}$ = 51.0 Hz, *J* = 2.2 Hz, 1H, C13), 4.84 (dd, *J* = 9.7, 8.0 Hz, 1H, C2), 4.61 (d, *J* = 8.0 Hz, 1H, C1), 4.24 (dd, *J* = 10.7, 10.6 Hz, 1H, C15), 4.02 (s, 2H, C21), 3.978 (ddd, $^3J_{FH}$ = 29.0 Hz, *J* = 10.7, 2.2 Hz, 1H, C14), 3.977 (dd, *J* = 11.2, 3.9 Hz, 1H, C6), 3.87 (s, 3H, Me), 3.88–3.79 (4H, overlapped, C6+C18+C19+C1'), 3.77 (ddd, *J* = 10.1, 3.9, 3.3 Hz, 1H, C5), 3.75 (dd, *J* = 10.6, 1.3 Hz, 1H, C16), 3.65 (dd, *J* = 11.5, 5.6 Hz, 1H, C19), 3.51 (dd, *J* = 9.1, 1.3 Hz, 1H, C17), 3.49 (dt, *J* = 9.7, 6.6 Hz, 1H, C1'), 2.02 (s, 3H, Ac), 2.01 (s, 3H, Ac), 1.96 (s, 3H, Ac), 1.59–1.24 (20H, overlapped, dodecyl), 0.90 (t, *J* = 7.1 Hz, 3H, C12'); ^{13}C{^{1}H} NMR (151 MHz, CD_3OD) δ 177.0 (C20), [171.7, 171.4, 171.1] (Ac), 168.5 (d, $^3J_{FC}$ = 3.9 Hz, C11), 101.8 (C1), 98.9 (d, $^2J_{FC}$ = 16.3 Hz, C12), 91.0 (d, $^1J_{FC}$ = 188.2 Hz, C3), 74.49 (C13), 74.46 (C16), 73.4 (C5), 72.9 (C2), 72.2 (C18), 71.1 (C1'), 70.7 (C4), 70.0 (C17), 69.8 (d, $^2J_{FC}$ = 18.6 Hz, C14), 64.8 (C19), 64.6 (C6), 62.6 (C21), 53.6 (Me), 48.3 (d, $^3J_{FC}$ = 3.4 Hz, C15), [33.1, 30.79, 30.76, 30.75, 30.7, 30.54, 30.46, 30.4, 27.0, 23.7] (dodecyl), [20.74, 20.66, 20.6] (Ac), 14.4 (C12'); ^{19}F NMR (564 MHz, CD_3OD) δ -219.4 (dd, $^2J_{FH}$ = 51.0 Hz, $^3J_{FH}$ = 29.0 Hz); ESI-MS found: 836.3704 calcd: 836.3687 for $C_{36}H_{60}FNNaO_{18}$ $[M+Na]^+$; IR: 3342, 2925, 2855, 1754, 1652, 1548, 1435, 1367, 1246, 1216, 1159, 1035, 959, 905, 864, 809, 764, 724.

Compound **19**

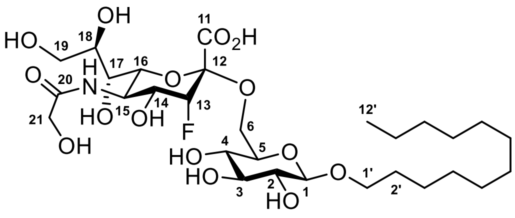

NaOMe (5.6 mg, 0.10 mmol, 5.0 eq.) was added to a solution of compound **18** (17 mg, 0.021 mmol) in MeOH (1 mL) at room temperature and stirred for 16 h. Then, H_2O (0.15 mL) was added and stirring was continued for 5 hours. After dilution with MeOH (1 mL), the mixture was neutralized with Amberlyst® 15 hydrogen form, filtered through a Celite® pad (washed with MeOH) and concentrated in vacuo. Chromatographic purification (SiO2, $CHCl_3$:MeOH:H_2O = 70:26:4) gave compound **19** (12.6 mg, 0.0197 mmol, 94%) as a colorless solid.

19: R_f 0.13 ($CHCl_3$:MeOH:H_2O = 70:26:4); ^{1}H NMR (599 MHz, CD_3OD) δ 5.17 (dd, $^2J_{FH}$ = 52.1 Hz, J = 2.2 Hz, 1H, C13), 4.22 (d, J = 7.8 Hz, 1H, C1), 4.18 (t, J = 10.6 Hz, 1H, C15), 4.11 (dd, J = 10.9, 4.3 Hz, 1H, C6), 4.07–4.01 (m, 2H, C21), 3.98 (ddd, $^3J_{FH}$ = 28.7 Hz, J = 10.6, 2.2 Hz, 1H, C14), 3.89 (ddd, J = 8.8, 5.7, 2.6 Hz, 1H, C18), 3.85–3.81 (1H, overlapped, C1'), 3.82 (dd, J = 11.5, 2.6 Hz, 1H, C19), 3.81 (dd, J = 10.9, 2.3 Hz, 1H, C6), 3.71 (dd, J = 10.6, 1.8 Hz, 1H, C16), 3.62 (dd, J = 11.5, 5.7 Hz, 1H, C19), 3.55 (t, J = 9.4 Hz, 1H, C4), 3.52 (dd, J = 9.1, 1.8 Hz, 1H, C17), 3.49 (dt, J = 9.5, 6.9 Hz, 1H, C1'), 3.34 (t, J = 9.2 Hz, 1H, C3), 3.34–3.30 (1H, overlapped, C5), 3.18 (dd, J = 9.3, 7.8 Hz, 1H, C2), 1.61 (dq, J = 8.7, 6.6 Hz, 2H, C2'), 1.40–1.24 (18H, overlapped, dodecyl), 0.90 (t, J = 7.0 Hz, 3H, C12'); ^{13}C{^{1}H} NMR (151 MHz, CD_3OD) δ 177.4 (C20), 172.0 (d, $^3J_{FC}$ = 3.0 Hz, C11), 104.4 (C1), 100.4 (d, $^2J_{FC}$ = 13.6 Hz, C12), 92.5 (d, $^1J_{FC}$ = 185.5 Hz, C13), 77.8 (C3), 76.4 (C5), 75.1 (C2), 74.1 (C16), 72.9 (C18), 71.3 (C4), 70.91 (d, $^2J_{FC}$ = 18.4 Hz, C14), 70.90 (C1'), 70.1 (C17), 64.5 (C6), 64.4 (C19), 62.6 (C21), 48.7 (d, $^3J_{FC}$ = 2.7 Hz, C15), [33.1, 30.80, 30.77, 30.75 (3C), 30.7, 30.5, 27.1, 23.7] (dodecyl), 14.4 (C12'); ^{19}F NMR (564 MHz, CD_3OD) δ -219.06 (dd, $^2J_{FH}$ = 52.1 Hz, $^3J_{FH}$ = 28.7 Hz); ESI-MS found: 672.32421 calcd: 672.32482 for $C_{29}H_{51}FNO_{15}^-$ [M-H]$^-$; IR: 3304, 2923, 2854, 1622, 1548, 1379, 1294, 1262, 1204, 1032, 953, 889, 800, 761, 720.

Compound **21**

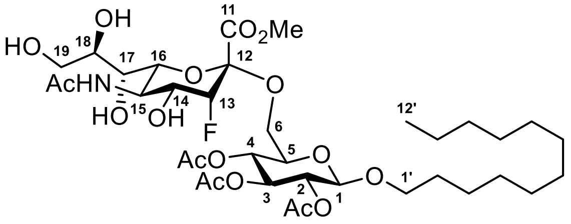

A solution of TMSOTf (0.10 M in CH_2Cl_2, 0.70 mL, 0.070 mmol, 1.1 eq.) was added to a mixture of compound **20**[1] (52.3 mg, 0.0636 mmol), compound **13** (60.3 mg, 0.127 mmol, 2.0 eq.) and Drierite™ (75 mg) in CH_2Cl_2 (1.5 mL) at 0 °C. After stirring for 1 h, the reaction was quenched by adding NEt_3 (0.1 mL). The mixture was filtered through a Celite® pad (washed with EtOAc) and concentrated in vacuo. Chromatographic purification (SiO_2, cHex:EtOAc = 75:25 to 65:35) afforded crude material. Then a flask was thoroughly purged with argon and charged with 20% $Pd(OH)_2$ (11.3 mg). Subsequently a solution of the obtained crude material in MeOH (4 mL) was added. The Ar atmosphere was replaced by H_2 gas(1 atm) and the mixture was stirred for 19 h at room temperature. After changing the atmosphere from H_2 back to argon, the mixture was filtered through a Celite® pad (washed with MeOH) and concentrated in vacuo. Chromatographic purification (SiO_2, cHex:acetone = 1:1 to 4:6 then CH_2Cl_2:MeOH = 9:1) afforded compound **21** (29.2 mg, 0.0366 mmol, 58%) as a colorless oil.

21: $[\alpha]_D^{27}$ -1.5 (c 1.00, MeOH); ^{1}H NMR (599 MHz, CD_3OD) δ 5.26 (dd, J = 9.7, 9.5 Hz, 1H, C3), 5.09 (dd, J = 9.6, 9.5 Hz, 1H, C4), 5.03 (dd, $^2J_{FH}$ = 50.9 Hz, J = 2.1 Hz, 1H, C13), 4.88 (dd, J = 9.7, 8.0 Hz, 1H, C2), 4.64 (d, J = 8.0 Hz, 1H, C1), 4.20 (dd, J = 10.7, 10.5 Hz, 1H, C15), 4.01 (dd, J = 11.3, 3.9 Hz, 1H, C6), 3.92–3.77 (6H, overlapped, C5+C6+C14+C18+C19+C1'), 3.91 (s, 3H, Me), 3.70 (dd, J = 11.5, 5.6 Hz, 1H, C19), 3.66 (dd, J = 10.5, 1.2 Hz, 1H, C16), 3.55 (dd, J = 9.1, 1.2 Hz, 1H, C17), 3.53 (dt, J = 9.7, 6.7 Hz, 1H, C1'), 2.054 (s, 3H, Ac), 2.047 (s, 3H, Ac), 2.03 (s, 3H, Ac), 2.00 (s, 3H, Ac), 1.65–1.26 (20H, overlapped, dodecyl), 0.94 (t, J = 7.0 Hz, 3H, C12'); ^{13}C{^{1}H} NMR (151 MHz, CD_3OD) δ [175.1, 171.7, 171.4, 171.1] (Ac), 168.6 (d, $^3J_{FC}$ = 3.9 Hz, C11), 101.8 (C1), 98.9 (d, $^2J_{FC}$ = 16.3 Hz, C12), 90.9 (d, $^1J_{FC}$ = 188.2 Hz, C13), 74.8 (C16), 74.5 (C3), 73.5 (C5), 72.9 (C2), 72.0 (C18), 71.1 (C1'), 70.7 (C4), 70.2 (d, $^2J_{FC}$ = 18.8 Hz, C14), 70.1 (C17), 64.8 (C19), 64.6 (C6), 53.6 (Me), 48.7 (d, $^3J_{FC}$ = 3.4 Hz, C15), [33.1, 30.78, 30.75, 30.74, 30.70, 30.54, 30.45, 30.4, 27.0, 23.7] (dodecyl), [22.7, 20.74, 20.66, 20.6] (Ac), 14.4 (C12'); ^{19}F NMR (564 MHz, CD_3OD) δ -220.8 (dd, $^2J_{FH}$ = 50.9 Hz, $^3J_{FH}$ = 28.7 Hz); ESI-MS found: 820.3748 calcd: 820.3737 for $C_{36}H_{60}FNNaO_{17}$ [M+Na]$^+$; IR: 3354, 2927, 2855, 1749, 1650, 1551, 1435, 1369, 1246, 1216, 1155, 1034, 966, 905, 806, 762, 725, 704.

Compound **22**

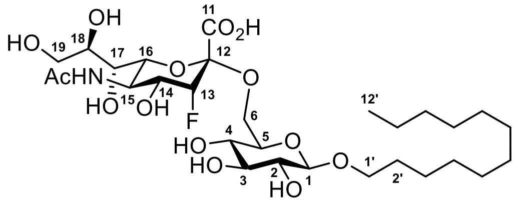

NaOMe (3.7 mg, 0.07 mmol, 5.0 eq.) was added to a solution of compound **21** (11 mg, 0.0138 mmol) in MeOH (1 mL) at room temperature and stirred for 16 h. Then, H_2O (0.15 mL) was added and stirring was continued for 5 hours. After dilution with MeOH (1 mL), the mixture was neutralized with Amberlyst® 15 hydrogen form, filtered through a Celite® pad (washed with MeOH) and concentrated in vacuo. Chromatographic purification (SiO2, $CHCl_3$:MeOH:H_2O = 70:26:4) gave compound **22** (7.7 mg, 0.0117 mmol, 85%) as a colorless solid.

22: R_f 0.13 ($CHCl_3$:MeOH:H_2O = 70:26:4); ^{1}H NMR (599 MHz, CD_3OD) δ 5.15 (dd, $^2J_{FH}$ = 52.1 Hz, J = 2.2 Hz, 1H, C13), 4.21 (d, J = 7.8 Hz, 1H, C1), 4.11 (dd, J = 10.9, 4.3 Hz, 1H, C6), 4.11 (t, J = 10.6 Hz, 1H, C15), 3.89 (ddd, J = 9.2, 5.5, 2.6 Hz, 1H, C18), 3.85–3.81 (1H, overlapped, C1'), 3.84 (ddd, $^3J_{FH}$ = 28.5 Hz, J = 10.6, 2.1 Hz, 1H, C14), 3.82–3.79 (1H, overlapped, C6), 3.81–3.79 (1H, overlapped, C19), 3.62 (dd, J = 11.5, 5.4 Hz, 1H, C19), 3.61 (dd, J = 10.6, 1.8 Hz, 1H, C16), 3.55 (t, J = 9.4 Hz, 1H, C4), 3.52 (dd, J = 9.1, 1.9 Hz, 1H, C17), 3.49 (dt, J = 9.6, 6.9 Hz, 1H, C1'), 3.34 (t, J = 9.1 Hz, 1H, C3), 3.34–3.30 (1H, overlapped, C5), 3.17 (dd, J = 9.3, 7.8 Hz, 1H, C2), 2.01 (s, 3H, NHAc), 1.60 (dq, J = 8.8, 6.6 Hz, 2H, C2'), 1.40–1.25 (18H, overlapped, dodecyl), 0.90 (t, J = 7.0 Hz, 3H, C12'); ^{13}C{^{1}H} NMR (151 MHz, CD_3OD) δ 175.5 (NHAc), 171.9 (d, $^3J_{FC}$ = 2.8 Hz, C11), 104.4 (C1), 100.4 (d, $^2J_{FC}$ = 13.7 Hz, C12), 92.4 (d, $^1J_{FC}$ = 185.7 Hz, C13), 77.8 (C3), 76.4 (C5), 75.1 (C2), 74.4 (C16), 72.8 (C17), 71.26 (C4), 71.22 (d, $^2J_{FC}$ = 18.6 Hz, C14), 70.9 (C1'), 70.1 (C17), 64.5 (C6), 64.4 (C19), 49.1 (C15), [33.1, 30.80, 30.77, 30.75 (3C), 30.7, 30.5, 27.1, 23.7] (dodecyl), 14.4 (C12'); ^{19}F NMR (564 MHz, CD_3OD) δ -218.95 (dd, $^2J_{FH}$ = 52.1 Hz, $^3J_{FH}$ = 28.5 Hz); ESI-MS found: 656.32927 calcd: 656.32991 for $C_{29}H_{51}FNO_{14}^-$ [M-H]$^-$; IR: 3298, 2924, 2854, 1622, 1563, 1377, 1311, 1261, 1204, 1033, 960, 890, 801, 762, 721.

Compound **23**[8]

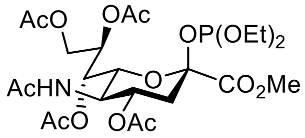

^{i}Pr_2NEt (0.22 mL, 1.26 mmol, 2.0 eq.) and $ClP(OEt)_2$ (0.14 mL, 0.98 mmol, 1.5 eq.) were successively added to a solution of 4,7,8,9-tetra-*O*-acetyl-*N*-acetylneuraminic acid methyl ester (314 mg, 0.639 mmol) in MeCN (3 mL) at room temperature. After stirring for 4 h, the mixture was concentrated in vacuo. Chromatographic purification (SiO_2, cHex:EtOAc = 3:7 to 2:8) afforded compound **23** (73.9 mg, 0.121 mmol, 19%) as colorless needles.

23: $[\alpha]_D^{26}$ -5.9 (c 1.00, MeCN); ^{1}H NMR (599 MHz, CD_2Cl_2) δ 5.41–5.36 (br, 1H, NH), 5.38 (dd, J = 4.5, 2.3 Hz, 1H, C7), 5.26 (ddd, J = 11.5, 10.5, 4.9 Hz, 1H, C4), 5.13 (ddd, J = 7.1, 4.5, 2.6 Hz, 1H, C8), 4.54 (dd, J = 12.3, 2.6 Hz, 1H, C9), 4.26 (dd, J = 10.7, 2.3 Hz, 1H, C6), 4.13 (dd, J = 12.3, 7.1 Hz, 1H, C9), 4.06 (ddd, J = 10.7, 10.5, 10.3 Hz, 1H, C5), 3.99 (m, 1H, Et), 3.95–3.87 (3H, overlapped, Et), 3.79 (s,

3H, Me), 2.49 (dd, J = 13.1, 4.9 Hz, 1H, C3), 2.10 (s, 3H, Ac), 2.03 (s, 3H, Ac), 2.00 (s, 3H, Ac), 1.99 (s, 3H, Ac), 1.96 (ddd, J = 13.1, 11.5, 1.5 Hz, 1H, C3), 1.85 (s, 3H, Ac), 1.27 (t, J = 7.1 Hz, 3H, Et), 1.24 (t, J = 7.0 Hz, 3H, Et); $^{13}C\{^1H\}$ NMR (151 MHz, CD_2Cl_2) δ [171.2, 170.9, 170.8, 170.51, 170.47] (Ac), 168.1 (d, $^3J_{PC}$ = 1.0 Hz, C1), 97.8 (d, $^2J_{FC}$ = 7.4 Hz, C2), 72.8 (d, $^4J_{FC}$ = 1.5 Hz, C6), 72.2 (d, J_{PC} = 1.5 Hz, C8), 69.1 (C4), 68.9 (C7), 62.9 (C9), 59.6 (d, $^2J_{PC}$ = 12.1 Hz, Et), 58.9 (d, $^2J_{PC}$ = 6.8 Hz, Et), 53.3 (Me), 49.9 (C5), 38.8 (d, $^3J_{PC}$ = 2.6 Hz, C3), [23.5, 21.4 (d, J_{PC} = 1.8 Hz), 21.23, 21.16, 21.1] (Ac), 17.2 (d, $^3J_{PC}$ = 4.5 Hz, Et), 17.1 (d, $^3J_{PC}$ = 5.2 Hz, Et); ^{31}P NMR (243 MHz, CD_2Cl_2) δ 136.7 (quin, J_{PH} = 7.9 Hz); ESI-MS found: 634.1873 calcd: 634.1871 for $C_{24}H_{38}NNaO_{15}P$ $[M+Na]^+$; IR: 3247, 3073, 2982, 2906, 1744, 1733, 1652, 1560, 1437, 1371, 1316, 1270, 1210, 1165, 1136, 1117, 1076, 1028, 996, 943, 931, 915, 904, 876, 857, 800, 760, 734, 684, 664.

$^{13}C\{^1H_{sel}\}$ (δ^1H_{sel} = 3.79 (OMe)): δ 168.1 ($\nu_{1/2} \approx$ 2 Hz, C1).

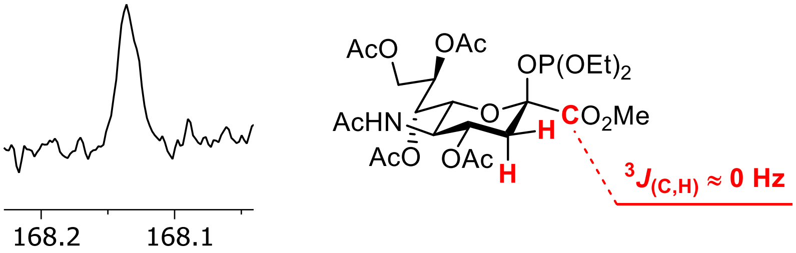

Compounds **25** (α-anomer) and **26** (β-anomer)

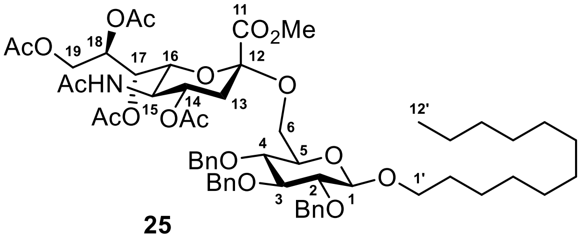

25

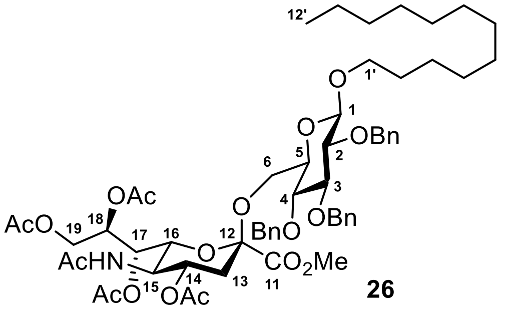

26

A solution of TMSOTf (0.10 M in MeCN, 0.45 mL, 0.045 mmol, 0.2 eq.) was added to a mixture of compound **23** (139 mg, 0.227 mmol) and **24** (282 mg, 0.456 mmol, 2.0 eq.) in MeCN (4.5 mL) and CH_2Cl_2 (2.5 mL) at 0 °C. After stirring for 1 h, the reaction was quenched by adding NEt_3 (0.15 mL). The mixture was concentrated in vacuo. Chromatographic purification (SiO_2, cHex:EtOAc = 1:1 to 3:7) afforded compound **25** (62.5 mg, 0.0572 mmol, 25%) and compound **26** (51.3 mg, 0.0470 mmol, 21%), both as a colorless oil.

25: $[\alpha]_D^{25}$ -6.6 (c 1.00, $CHCl_3$); ^1H NMR (599 MHz, $CDCl_3$) δ 7.35–7.24 (15H, overlapped, Bn), 5.41 (ddd, J = 9.2, 5.2, 2.7 Hz, 1H, C18), 5.31 (dd, J = 9.2, 2.2 Hz, 1H, C17), 5.12 (brd, J = 9.7 Hz, 1H, NH), 4.93 (d, J = 11.0 Hz, 1H, O2-Bn), 4.88 (d, J = 11.0 Hz, 1H, O3-Bn), 4.85 (ddd, J = 12.5, 9.9, 4.6 Hz, 1H, C14), 4.79 (d, J = 10.5 Hz, 1H, O4-Bn), 4.77 (d, J = 10.5 Hz, 1H, O4-Bn), 4.76 (d, J = 11.0 Hz, 1H, O3-Bn), 4.71 (d, J = 11.0 Hz, 1H, O2-Bn), 4.33 (d, J = 7.9 Hz, 1H, C1), 4.20 (dd, J = 12.6, 2.7 Hz, 1H, C19), 4.16

(dd, J = 11.1, 4.4 Hz, 1H, C6), 4.08 (dd, J = 10.7, 2.2 Hz, 1H, C16), 4.04 (ddd, J = 10.7, 9.9, 9.7 Hz, 1H, C15), 3.98 (dd, J = 12.6, 5.2 Hz, 1H, C19), 3.91 (dt, J = 9.5, 6.4 Hz, 1H, C1'), 3.74 (s, 3H, Me), 3.64 (dd, J = 9.3, 9.0 Hz, 1H, C4), 3.59 (dd, J = 9.1, 9.0 Hz, 1H, C3), 3.57 (dd, J = 11.1, 1.6 Hz, 1H, C6), 3.49 (dt, J = 9.5, 6.9 Hz, 1H, C1'), 3.41–3.37 (1H, overlapped, C5), 3.39 (dd, J = 9.1, 7.9 Hz, 1H, C2), 2.66 (dd, J = 12.8, 4.6 Hz, 1H, C13), 2.13 (s, 3H, Ac), 2.03 (s, 6H, Ac), 1.96 (dd, J = 12.8, 12.5 Hz, 1H, C13), 1.92 (s, 3H, Ac), 1.87 (s, 3H, Ac), 1.70–1.20 (Σ20H, dodecyl), 0.88 (t, J = 7.0 Hz, 3H, C12'); $^{13}C\{^1H\}$ NMR (151 MHz, $CDCl_3$) δ [171.1, 170.7, 170.4, 170.2, 170.0] (Ac), 168.1 (C11), [138.8, 138.63, 138.61, 128.49, 128.46 (2C), 128.3, 128.2, 128.0, 127.9, 127.8, 127.7] (Bn), 103.7 (C1), 98.9 (C12), 84.7 (C3), 82.2 (C2), 77.6 (C4), 75.9 (O3-Bn), 75.1 (O4-Bn), 74.9 (O2-Bn), 73.9 (C5), 72.4 (C16), 70.2 (C1'), 69.3 (C14), 68.0 (C18), 67.1 (C17), 63.8 (C6), 62.3 (C19), 52.7 (Me), 49.6 (C15), 38.3 (C13), [32.0, 29.9, 29.82, 29.77 (2C), 29.75, 29.6, 29.5, 26.4] (dodecyl), 23.3 (Ac), 22.8 (dodecyl), [21.3, 21.0, 20.9, 20.7] (Ac), 14.3 (C12'); MALDI-TOF MS {matrix = DHB (EtOAc)} found: 1114.55 calcd: 1114.53 for $C_{59}H_{81}NNaO_{18}$ $[M+Na]^+$; , IR: 3246, 3066, 3030, 2926, 2855, 1744, 1689, 1658, 1608, 1539, 1498, 1455, 1367, 1325, 1303, 1276, 1217, 1176, 1130, 1119, 1067, 1034, 973, 959, 941, 914, 879, 862, 821, 790, 751, 735, 698, 666.

$^{13}C\{^1H_{sel}\}$ (δ^1H_{sel} = 3.74 (OMe)): δ 168.1 (d, $^3J_{CH}$ ≈ 6.4 Hz, C11).

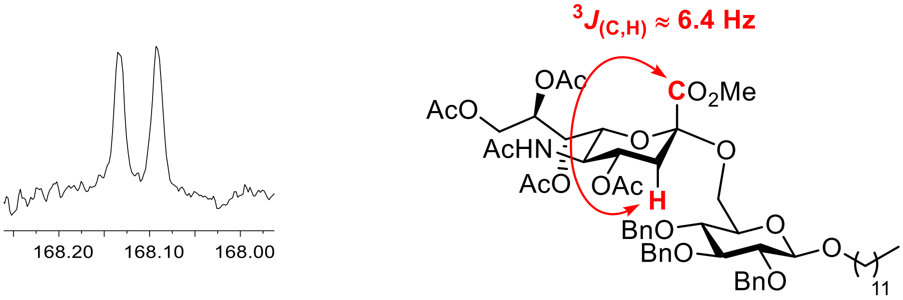

26: $[\alpha]_D^{24}$ -1.1 (c 1.00, $CHCl_3$); ^1H NMR (599 MHz, $CDCl_3$) δ 7.37–7.25 (15H, overlapped, Bn), 5.69 (m, 1H, NH), 5.38 (dd, J = 2.6, 2.4 Hz, 1H, C17), 5.27 (ddd, J = 11.5, 10.3, 4.9 Hz, 1H, C14), 5.24 (ddd, J = 8.7, 2.6, 2.5 Hz, 1H, C18), 4.97 (dd, J = 12.4, 2.5 Hz, 1H, C19), 4.96 (d, J = 10.9 Hz, 1H, O2-Bn), 4.92 (d, J = 10.9 Hz, 1H, O3-Bn), 4.87 (d, J = 10.8 Hz, 1H, O4-Bn), 4.81 (d, J = 10.9 Hz, 1H, O3-Bn), 4.75 (d, J = 10.9 Hz, 1H, O2-Bn), 4.71 (d, J = 10.8 Hz, 1H, O4-Bn), 4.44 (d, J = 7.8 Hz, 1H, C1), 4.29 (dd, J = 10.5, 2.4 Hz, 1H, C16), 4.17 (ddd, J = 10.5, 10.3, 10.0 Hz, 1H, C15), 4.10 (m, 1H, C19), 3.99 (dt, J = 9.7, 6.3 Hz, 1H, C1'), 3.85 (dd, J = 10.6, 2.1 Hz, 1H, C6), 3.78 (dd, J = 10.6, 2.4 Hz, 1H, C6), 3.77 (dd, J = 9.6, 9.2 Hz, 1H, C4), 3.70 (s, 3H, Me), 3.63 (dd, J = 9.2, 9.1 Hz, 1H, C3), 3.56 (dt, J = 9.7, 6.9 Hz, 1H, C1'), 3.46–3.42 (1H, overlapped, C5), 3.45 (dd, J = 9.1, 7.8 Hz, 1H, C2), 2.43 (dd, J = 12.9, 4.9 Hz, 1H, C13), 2.15 (s, 3H, Ac), 2.03 (s, 3H, Ac), 2.02 (s, 3H, Ac), 2.01 (s, 3H, Ac), 1.92 (dd, J = 12.9, 11.5 Hz, 1H, C13), 1.88 (s, 3H, Ac), 1.68–1.20 (Σ20H, dodecyl), 0.88 (t, J = 7.1 Hz, 3H, C12'); $^{13}C\{^1H\}$ NMR (151 MHz, $CDCl_3$) δ [171.0, 170.7, 170.5, 170.4, 170.2] (Ac), 167.2 (C11), [138.61, 138.55, 138.5, 128.54, 128.52, 128.51, 128.2, 128.1, 128.0, 127.84, 127.76] (Bn, one signal is missing, possibly due to overlapping), 103.9 (C1), 97.8 (C12), 84.5 (C3), 82.5 (C2), 77.0 (C4), 76.0 (O3-Bn), 75.3 (O2-Bn), 75.1 (O4-Bn), 73.6 (C5), 72.6 (C18), 71.6 (C16), 70.9 (C1'), 69.2 (C14), 68.8 (C17), 62.9 (C19), 61.1 (C6), 52.8 (Me), 48.9 (C15), 37.4 (C13), [32.0, 30.0, 29.83, 29.79, 29.77 (2C), 29.7, 29.5, 26.3] (dodecyl), 23.2 (Ac), 22.8 (dodecyl), [21.1, 21.04, 20.95 (2C)] (Ac), 14.3 (C12'); MALDI-TOF MS {matrix = DHB (EtOAc)} found: 1114.55 calcd: 1114.53 for $C_{59}H_{81}NNaO_{18}$ $[M+Na]^+$; IR: 3283, 3062, 3031, 2925, 2855, 1743, 1689, 1664, 1607, 1548, 1498, 1455, 1436, 1368, 1313, 1223, 1176, 1119, 1066, 1029, 944, 866, 824, 785, 752, 699, 667.

$^{13}C\{^1H_{sel}\}$ (δ^1H_{sel} = 3.70 (OMe)): δ 167.2 (d, $^3J_{CH}$ ≈ 1.2 Hz, C11).

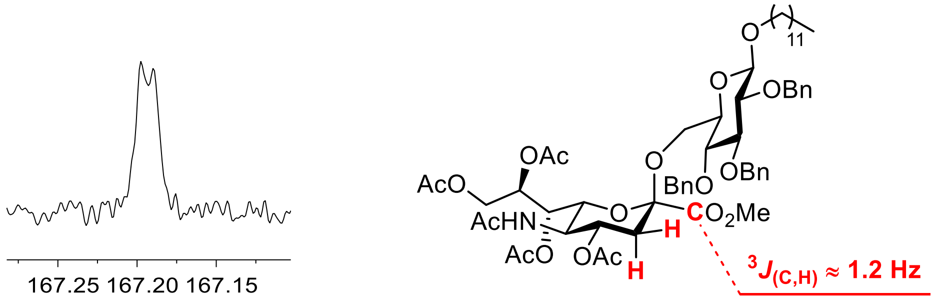

Compound **27**

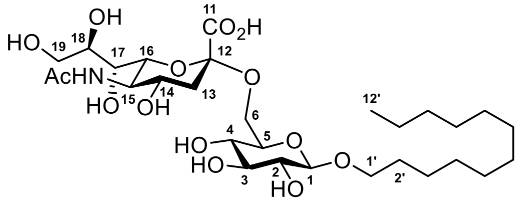

A flask with compound **25** (35 mg, 0.032 mmol and Pd/C (10% w/w) (34 mg, 0.032 mmol, 1.0 eq) was flushed with argon, then methanol (1.5 mL) and ethyl acetate (1.5 mL) were added. The argon atmosphere was replaced by hydrogen and the reaction mixture was allowed to stir at RT for 18 h. After this time, the reaction mixture was filtered through a Celite® pad (washed with MeOH) and the solvent was evaporated under reduced pressure. The resulting crude product was dissolved in methanol (1.5 mL) and NaOMe (7 mg, 0.128 mmol, 0.3 eq) was added. The reaction mixture was stirred at RT for 2 h. Then, H_2O was added and stirring was continued for 17 h. After this time, the reaction mixture was diluted with methanol (1.5 mL), neutralized with Amberlyst® 15 hydrogen form, filtered through a Celite® pad (washed with MeOH) and concentrated in vacuo. Chromatographic purification (SiO2, $CHCl_3$:MeOH:H_2O = 70:26:4) gave compound **27** (20 mg, 0.031 mmol, 97%) as a colorless solid.

27: R_f 0.12 ($CHCl_3$:MeOH:H_2O = 70:26:4); mp 215-225 °C (decomp.); $[\alpha]_D^{26}$ -23.9 (c 1.00, MeOH); ^1H NMR (599 MHz, CD_3OD) δ 4.21 (d, *J* = 7.8 Hz, 1H, C1), 4.05 (dd, *J* = 10.8, 4.4 Hz, 1H, C6), 3.88–3.81 (1H, overlapped, C18), 3.83 (dt, *J* = 9.9, 7.0 Hz, 1H, C1'), 3.82 (dd, *J* = 11.4, 2.6 Hz, 1H, C19), 3.73 (dd, *J* = 10.8, 2.3 Hz, 1H, C6), 3.73–3.68 (1H, overlapped, C14), 3.68 (t, *J* = 9.8 Hz, 1H, C15), 3.62 (dd, *J* = 11.4, 5.6 Hz, 1H, C19), 3.59 (dd, *J* = 10.0, 1.8 Hz, 1H, C16), 3.51 (dd, *J* = 9.1, 1.8 Hz, 1H, C17), 3.49 (dt, *J* = 9.5, 6.8 Hz, 1H, C1'), 3.46 (t, *J* = 9.4 Hz, 1H, C4), 3.34 (t, *J* = 9.2 Hz, 1H, C3), 3.32–3.28 (1H, overlapped, C5), 3.17 (dd, *J* = 9.3, 7.8 Hz, 1H, C2), 2.85 (dd, *J* = 12.4, 4.5 Hz, 1H, C13), 2.01 (s, 3H, NHAc), 1.61 (t, *J* = 11.7 Hz, 1H, C13), 1.64–1.58 (2H, overlapped, C2'), 1.43–1.19 (18H, overlapped, dodecyl), 0.90 (t, *J* = 7.0 Hz, 3H, C12'); $^{13}C\{^1H\}$ NMR (151 MHz, CD_3OD) δ 175.5 (NHAc), 174.3 (C11), 104.4 (C1), 101.6 (C12), 77.8 (C3), 76.3 (C5), 75.1 (C2), 74.3 (C16), 72.8 (C18), 71.5 (C4), 70.9 (C1'), 70.3 (C17), 69.5 (C14), 64.4 (C19), 64.3 (C6), 54.2 (C15), 42.5 (C13), [33.1, 30.79, 30.78, 30.74 (3C), 30.7, 30.5, 27.1, 23.7] (dodecyl), 22.6 (NHAc), 14.4 (C12'); ESI-MS found: 638.33891 calcd: 638.33997 for $C_{29}H_{52}NO_{14}^-$ [M-H]$^-$; IR: 3265, 2924, 2854, 2779, 1593, 1435, 1352, 1237, 1207, 1027, 897, 773, 765, 719.

Compound **28**

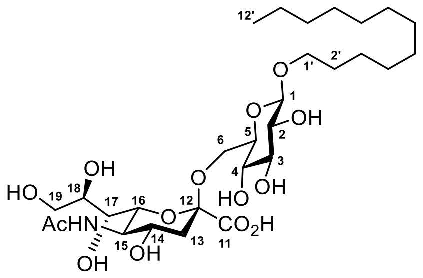

A flask with compound **26** (35 mg, 0.032 mmol and Pd/C (10% w/w) (34 mg, 0.032 mmol, 1.0 eq) was flushed with argon, then methanol (1.5 mL) and ethyl acetate (1.5 mL) were added. The argon atmosphere was replaced by hydrogen and the reaction mixture was allowed to stir at RT for 18 h. After this time, the reaction mixture was filtered through a Celite® pad (washed with MeOH) and the solvent was evaporated under reduced pressure. The resulting crude product was dissolved in methanol (1.5 mL) and NaOMe (7 mg, 0.128 mmol, 0.3 eq) was added. The reaction mixture was stirred at RT for 2 h. Then, H_2O was added and stirring was continued for 17 h. After this time, the reaction mixture was diluted with methanol (1.5 mL), neutralized with Amberlyst® 15 hydrogen form, filtered through a Celite® pad (washed with MeOH) and concentrated in vacuo. Chromatographic purification (SiO2, $CHCl_3$:MeOH:H_2O = 70:26:4) gave compound **28** (14 mg, 0.022 mmol, 68%) as a colorless solid.

28: R_f 0.13 ($CHCl_3$:MeOH:H_2O = 70:26:4); mp 220-230 °C (decomp.); $[\alpha]_D^{26}$ -26.0 (c 1.00, MeOH); ^{1}H NMR (599 MHz, CD_3OD) δ 4.21 (d, *J* = 7.9 Hz, 1H, C1), 4.02 (ddd, *J* = 11.3, 9.8, 4.9 Hz, 1H, C14), 3.93 (t, *J* = 10.1 Hz, 1H, C15), 3.90–3.84 (1H, overlapped, C1’), 3.87–3.84 (1H, overlapped, C16), 3.85 (d, *J* = 11.1 Hz, 1H, C6), 3.80–3.74 (2H, overlapped, C18+C19), 3.65 (dd, *J* = 11.3, 4.9 Hz, 1H, C19), 3.64 (t, *J* = 9.3 Hz, 1H, C4), 3.61 (dd, *J* = 11.1, 3.9 Hz, 1H, C6), 3.48 (dt, *J* = 9.3, 6.9 Hz, 1H, C1), 3.47–3.45 (1H, overlapped, C17), 3.37 (t, *J* = 9.3 Hz, 1H, C3), 3.29 (ddd, *J* = 9.9, 4.0, 1.4 Hz, 1H, C5), 3.18 (dd, *J* = 9.2, 7.9 Hz, 1H, C2), 2.36 (dd, *J* = 12.9, 4.9 Hz, 1H, C13), 1.66 (dd, *J* = 12.7, 11.5 Hz, 1H, C13), 1.64–1.59 (2H, overlapped, C2’), 1.41–1.26 (18H, overlapped, dodecyl), 0.90 (s, 3H, C12’); $^{13}C\{^1H\}$ NMR (151 MHz, CD_3OD) δ 176.1 (C11), 174.2 (NHAc), 104.8 (C1), 102.1 (C12), 77.2 (C3), 76.8 (C5), 75.3 (C2), 72.1 (C16), 71.4 (C18), 71.2 (C1’), 70.7 (C4), 70.3 (C17), 68.4 (C14), 65.3 (C19), 63.3 (C6), 53.6 (C15), 42.0 (C13), [33.1, 30.8, 30.76 (2C), 30.75 (2C), 30.72, 30.5, 27.1, 23.7] (dodecyl), 22.9 (NHAc), 14.4 (C12’); ESI-MS found: 638.33885 calcd: 638.33997 for $C_{29}H_{52}NO_{14}^-$ $[M\text{-}H]^-$; IR: 3272, 2925, 2854, 1595, 1411, 1375, 1352, 1167, 1068, 1022, 896, 841, 772, 720.

References

[1] T. Hayashi, G. Kehr, K. Bergander, R. Gilmour, *Angew. Chem. Int. Ed.* 2019, **58**, 3814-3818.

[2] The stereochemistry at the anomeric position of 3-F_{axial} sialosides was determined based on the $^3J(^{13}C1\text{-}C2\text{-}C3\text{-}^{19}F_{ax})$ coupling constant from the ^{13}C NMR. See; a) K. Suzuki, S. Daikoku, S.-H. Son, Y. Ito, O. Kanie, *Carbohydr. Res*. 2015, **406**,1-9; b) K. Bock, C. Pedersen, *Acta Chem. Scand. Ser. B* 1975, **29**, 682-686.

[3] The stereochemistry at the anomeric position of 3-H_{axial} sialosides was determined based on the $^3J(^{13}C1\text{-}C2\text{-}C3\text{-}^{1}H)$ coupling constant from the ^{13}C NMR. See; H. Hori, T. Nakajima, Y. Nishida, H. Ohrui, H. Meguro, *Tetrahedron Lett.* 1988, **29**, 6317-6320.

[4] a) S. Liu, R. Sang, S. Hong, Y. Cai, H. Wang, *Langmuir* 2013, **29**, 8511-8516; b) C. R. Noller, W. C. Rockwell, *J. Am. Chem. Soc.* 1938, **60**, 2076-2077.

[5] B. K. Gadakh, P. R. Patil, S. Malik, K. P. R. Kartha, *Synth. Commun*. 2009, **39**, 2430-2438.

[6] G. O. Aspinall, R. C. Carpenter, L. Khondo, *Carbohydr. Res.* 1987, **165**, 281-298.

[7] D. C. Harrowven, D. P. Curran, S. L. Kostiuk, I. L. Wallis-Guy, S. Whiting, K. J. Stenning, B. Tang, E. Packard, L. Nanson, *Chem. Commun*. 2010, **46**, 6335-6337.

[8] T. J. Martin, R. R. Schmidt, *Tetrahedron Lett*. 1992, **33**, 6123-6126.

Compound **2** (^{1}H NMR, 500 MHz, $CDCl_3$)

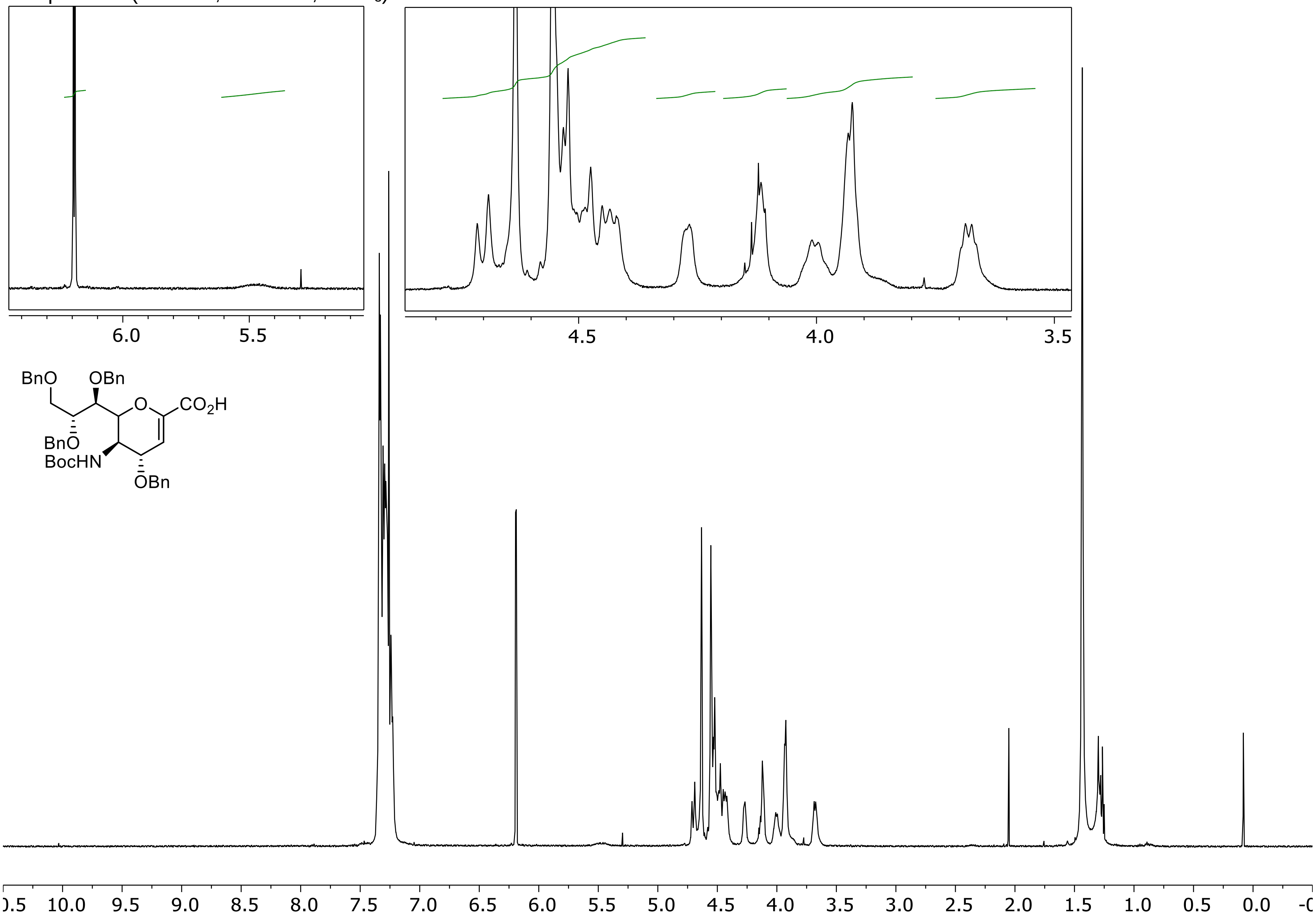

Compound **2** ($^{13}C\{^{1}H\}$ NMR, 126 MHz, $CDCl_3$)

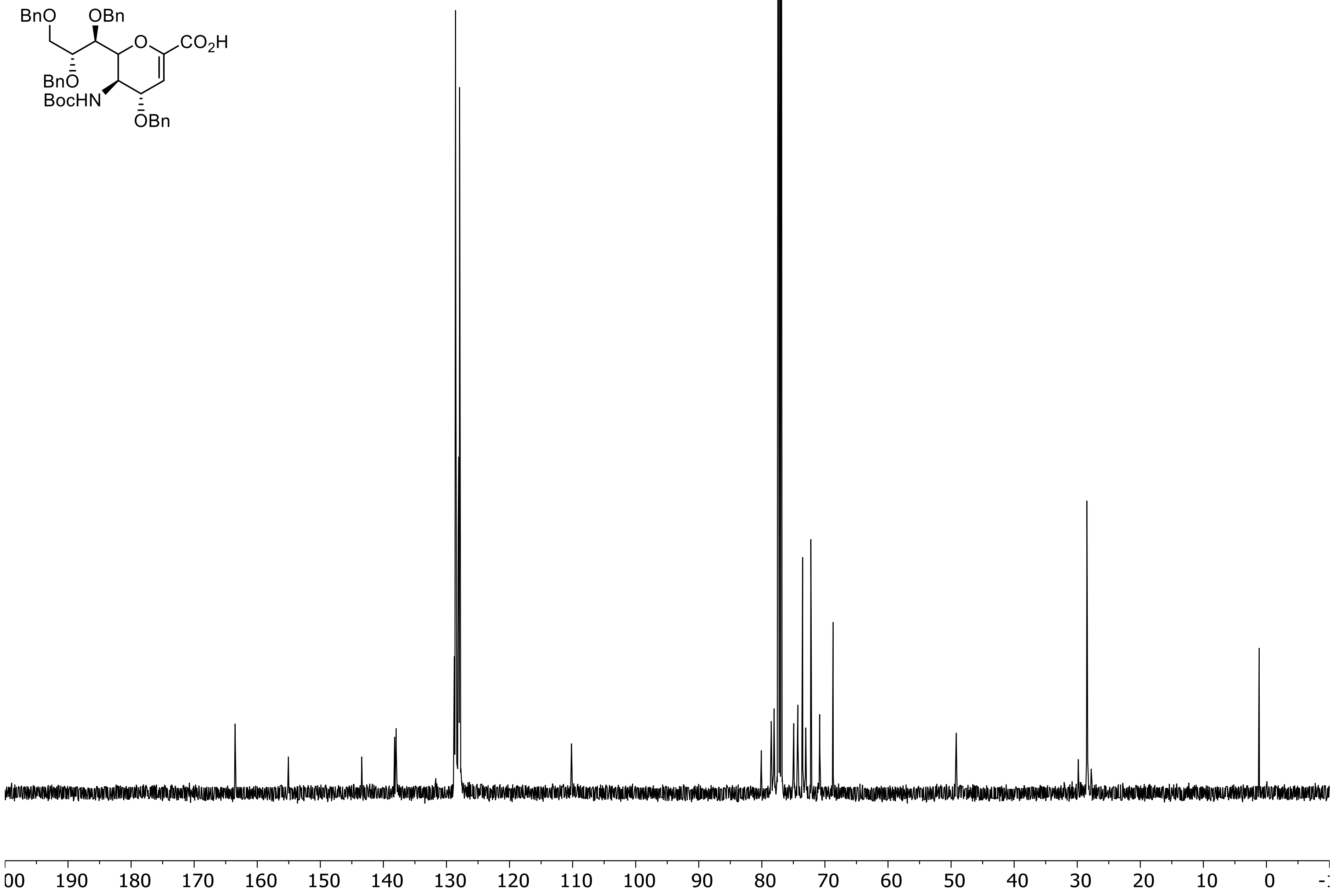

Compound **3** (^{1}H NMR, 500 MHz, $CDCl_3$)

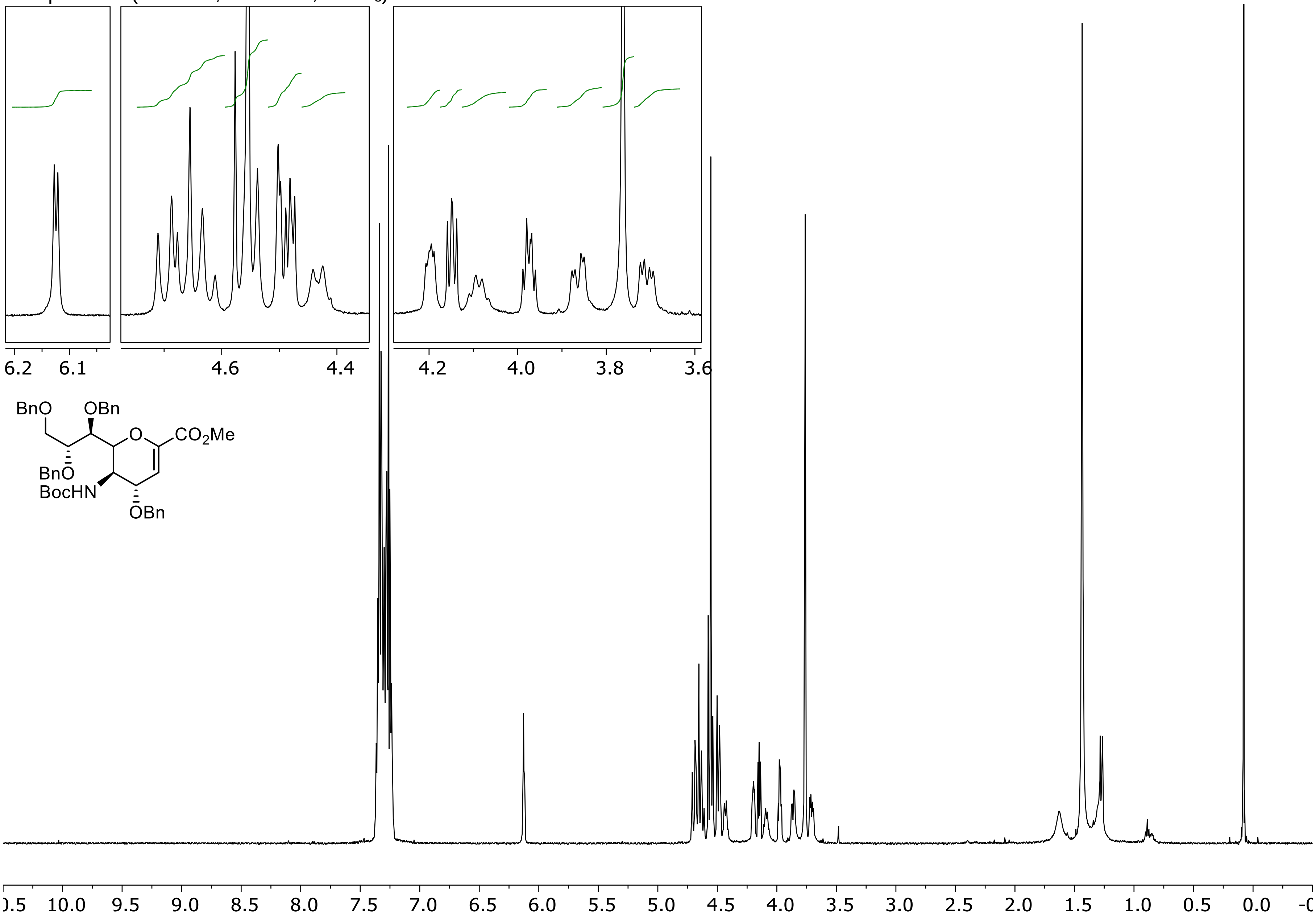

Compound **3** ($^{13}C\{^{1}H\}$ NMR, 126 MHz, $CDCl_3$)

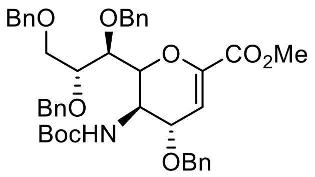

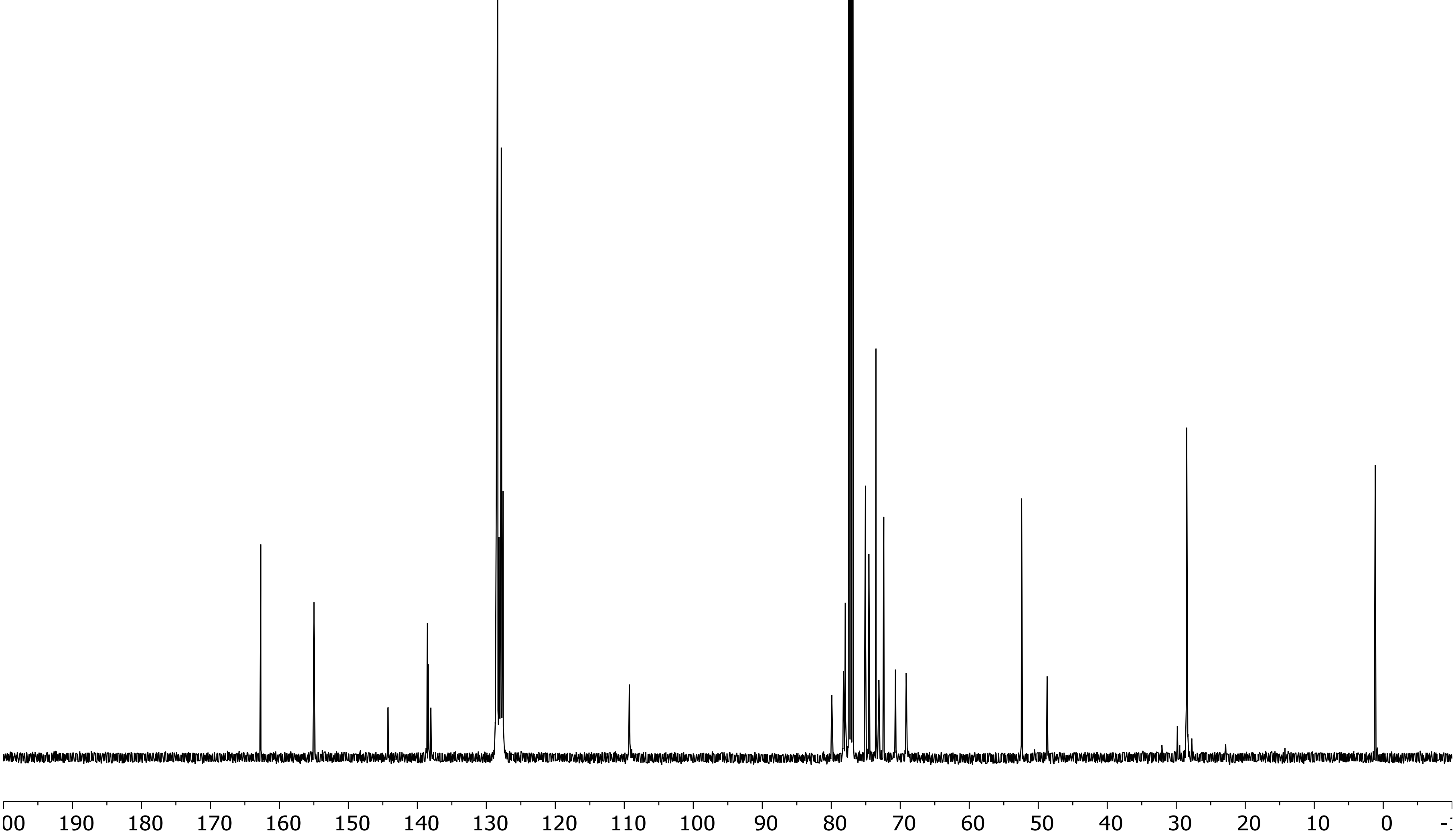

Compound **S1** (^{1}H NMR, 599 MHz, $CDCl_3$)

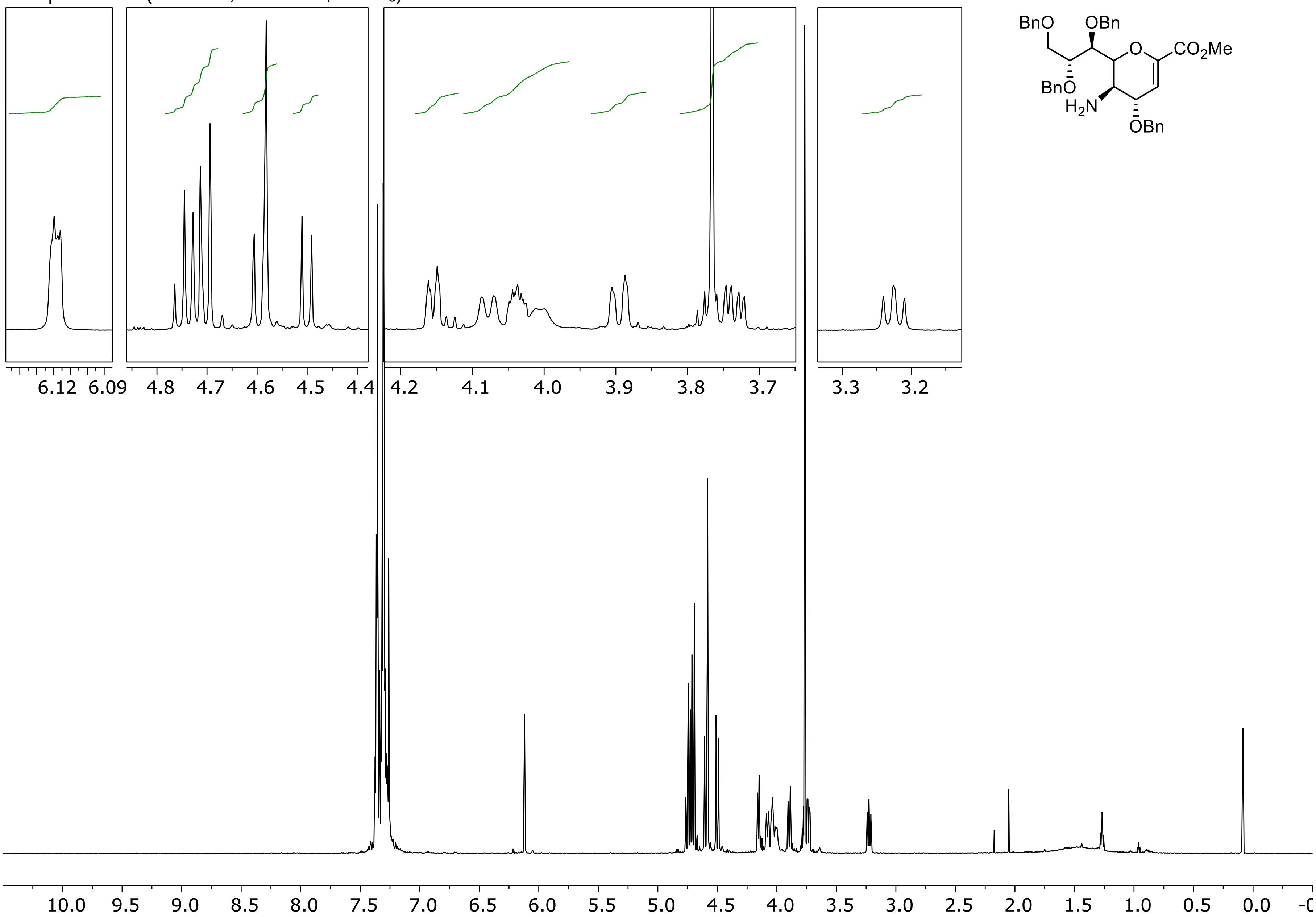

Compound **S1** ($^{13}C\{^{1}H\}$ NMR, 151 MHz, $CDCl_3$)

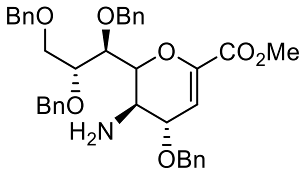

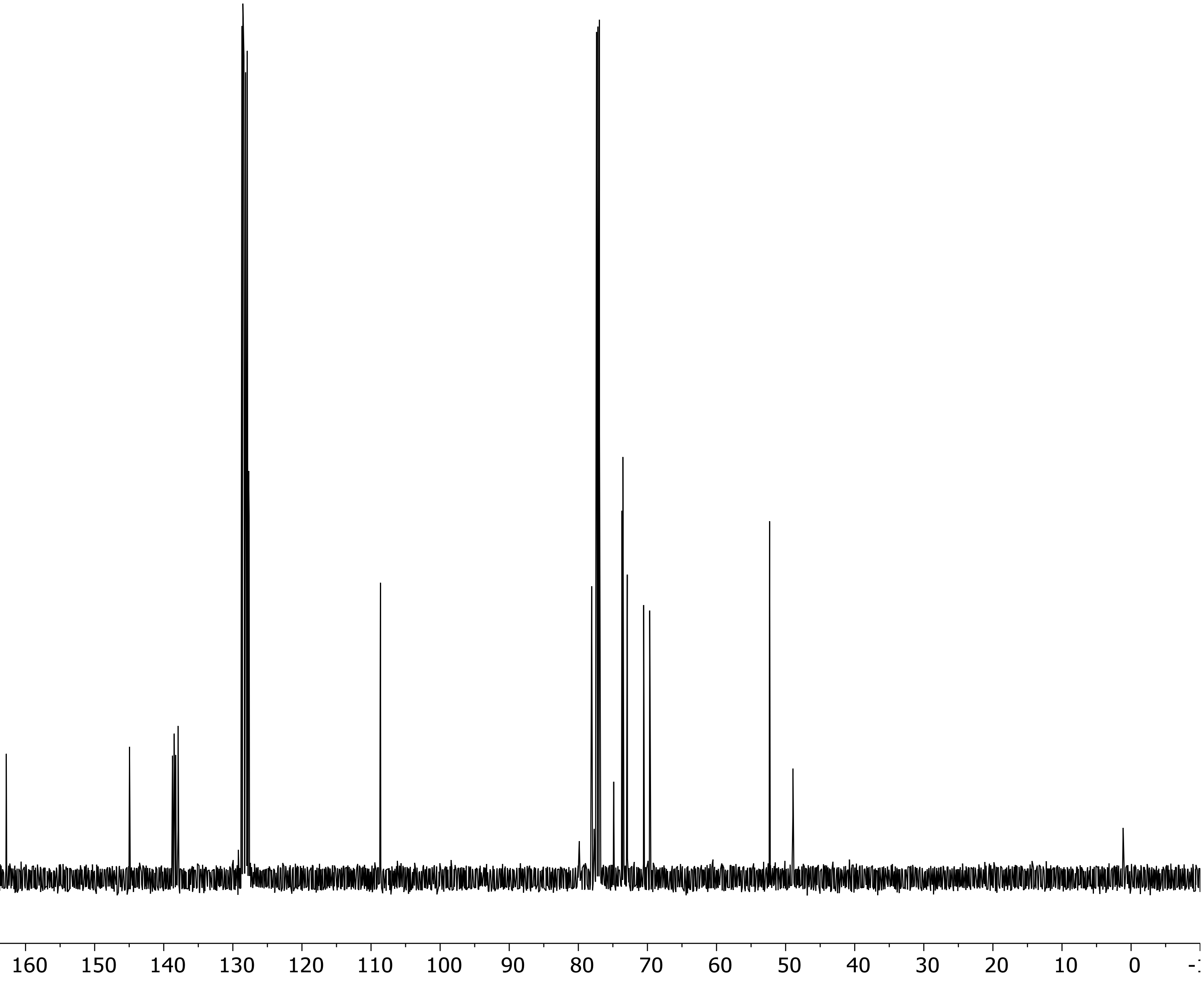

Compound **4** (^{1}H NMR, 599 MHz, $CDCl_3$)

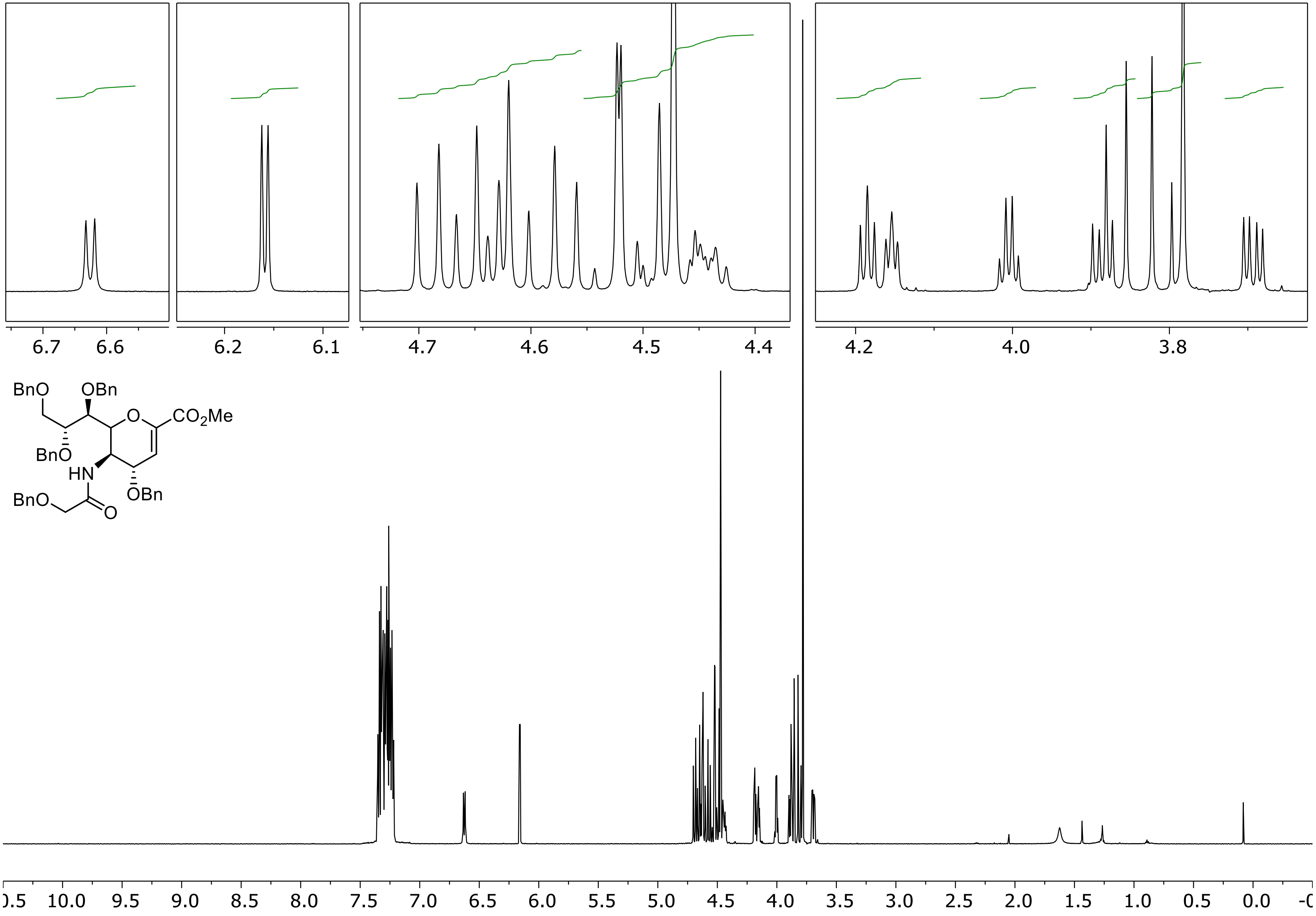

Compound **4** ($^{13}C\{^1H\}$ NMR, 151 MHz, $CDCl_3$)

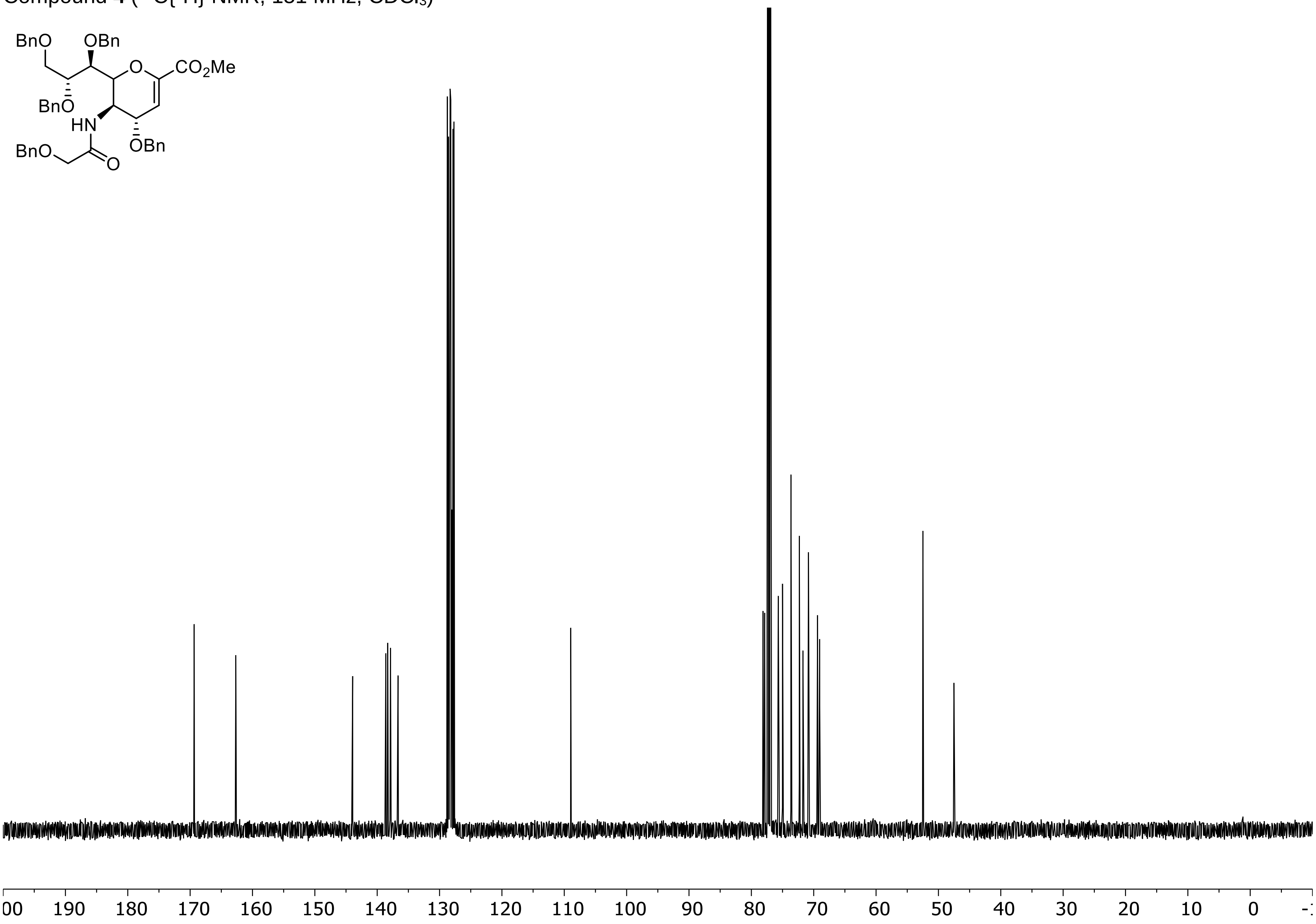

Compound **5** (^{1}H NMR, 599 MHz, $CDCl_3$)

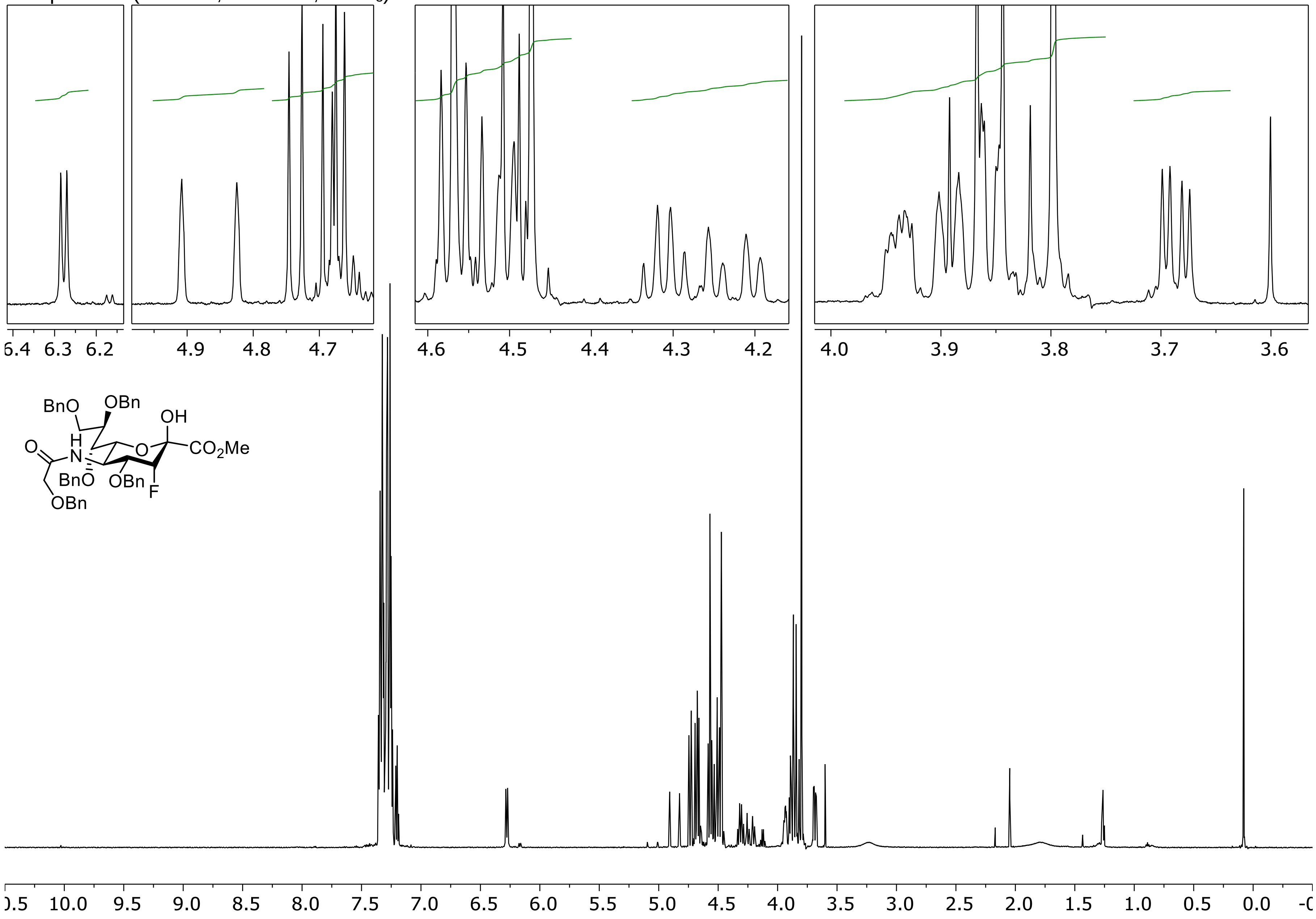

Compound **5** ($^{13}C\{^1H\}$ NMR, 151 MHz, $CDCl_3$)

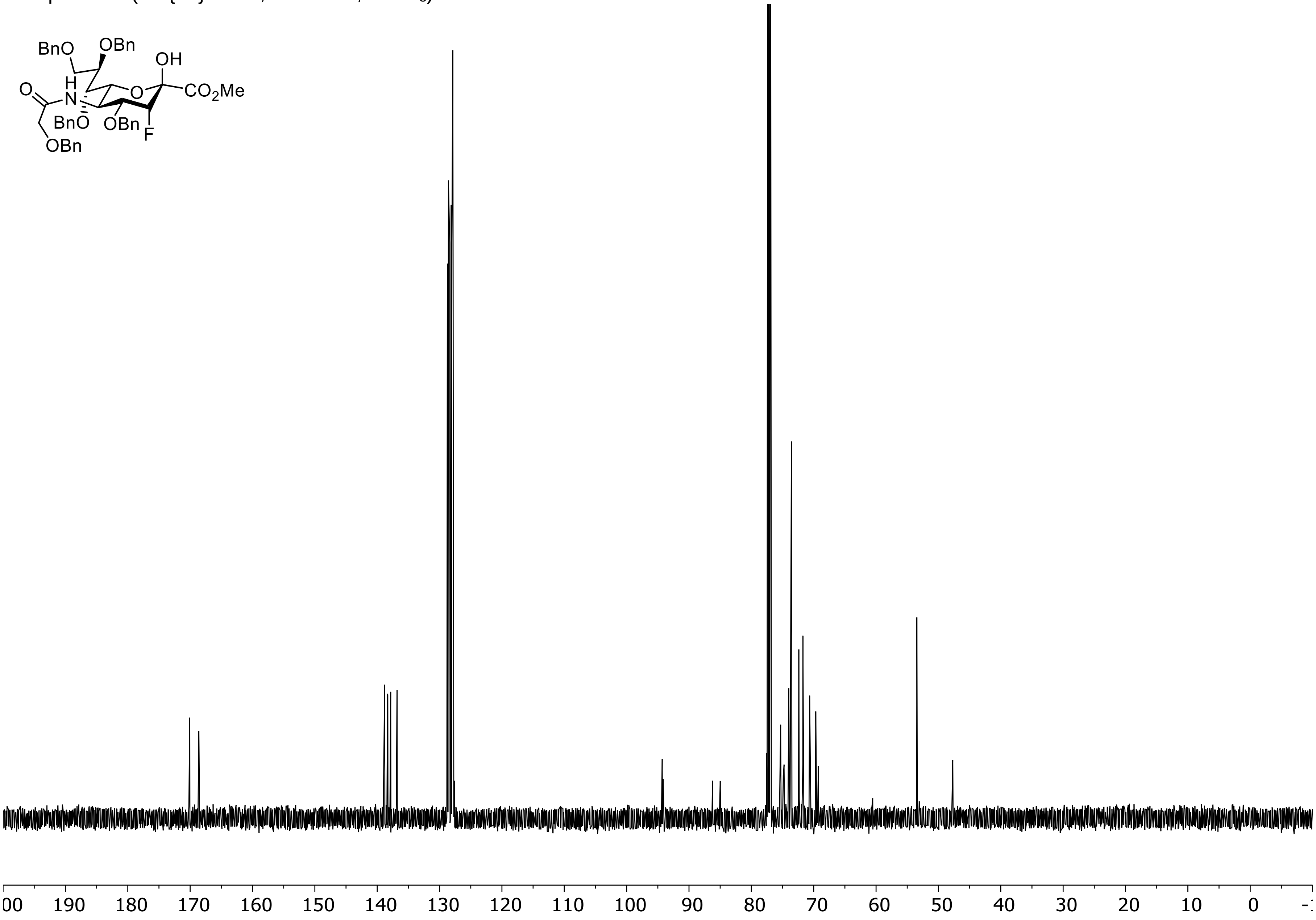

Compound **5** (^{19}F NMR, 564 MHz, $CDCl_3$)

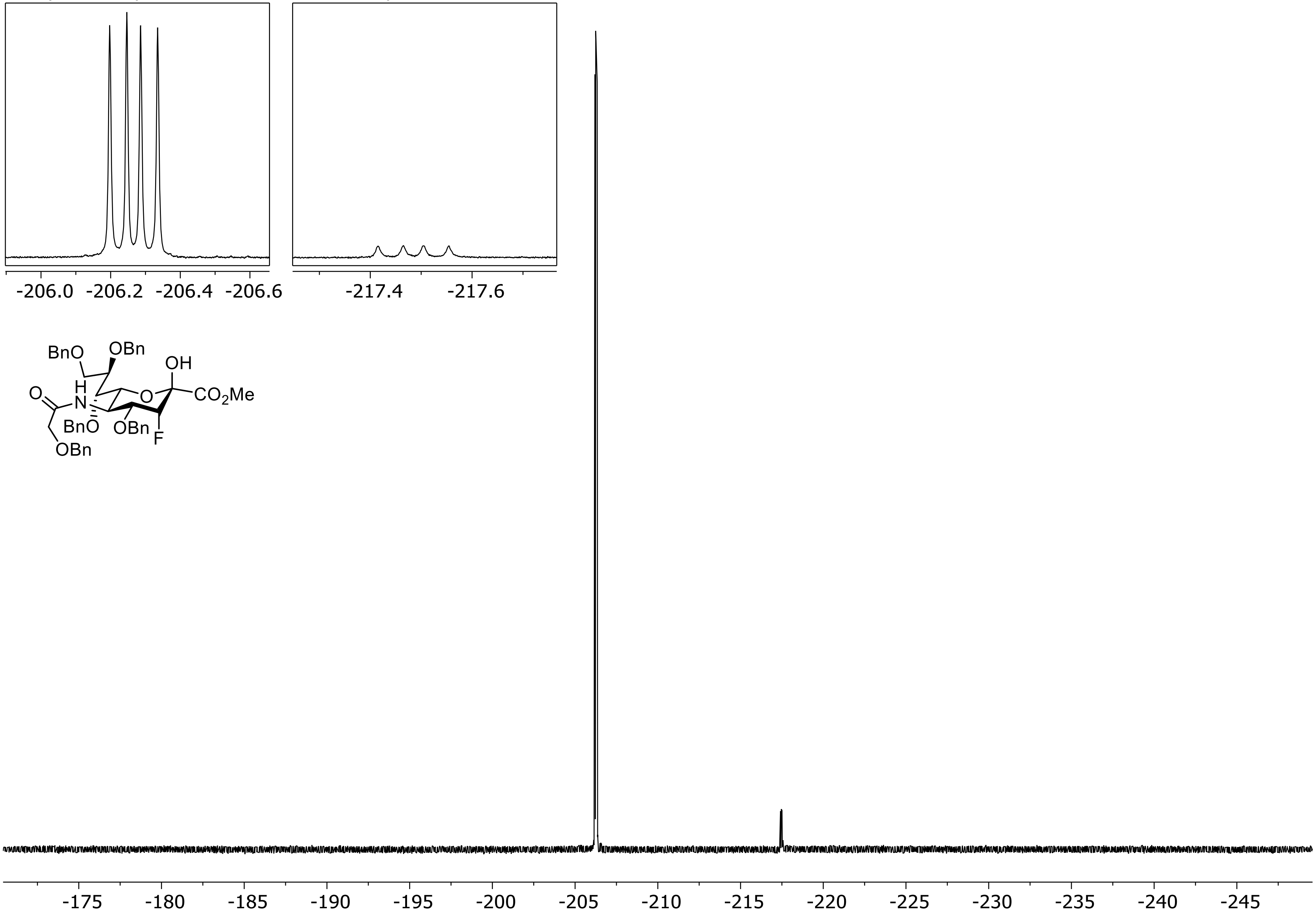

Compound **6** (^{1}H NMR, 599 MHz, $CDCl_3$)

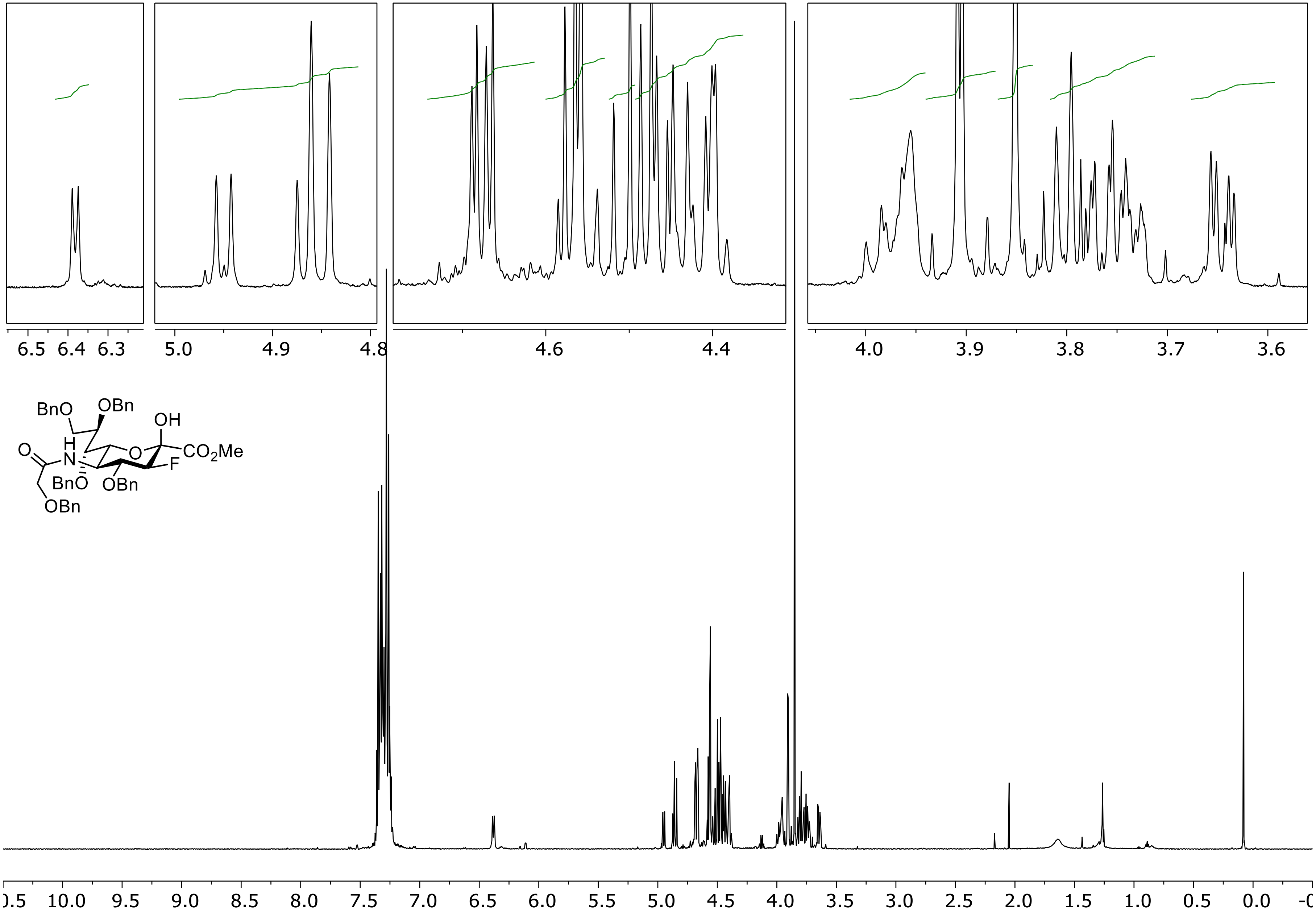

Compound **6** ($^{13}C\{^{1}H\}$ NMR, 151 MHz, $CDCl_3$)

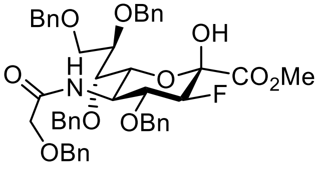

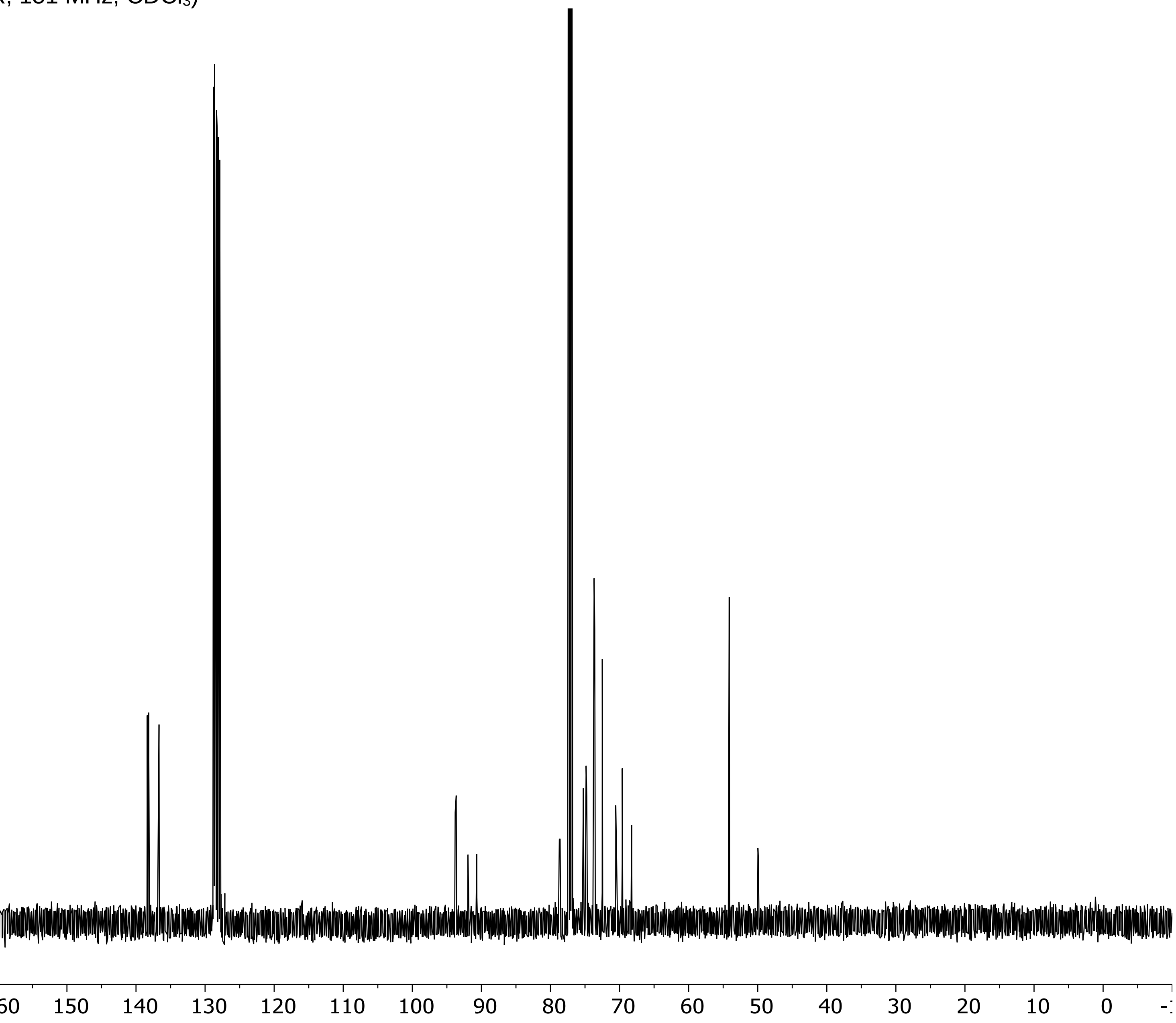

Compound **6** (^{19}F NMR, 564 MHz, $CDCl_3$)

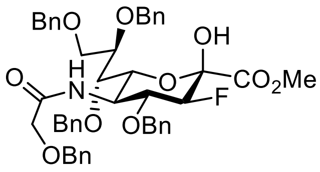

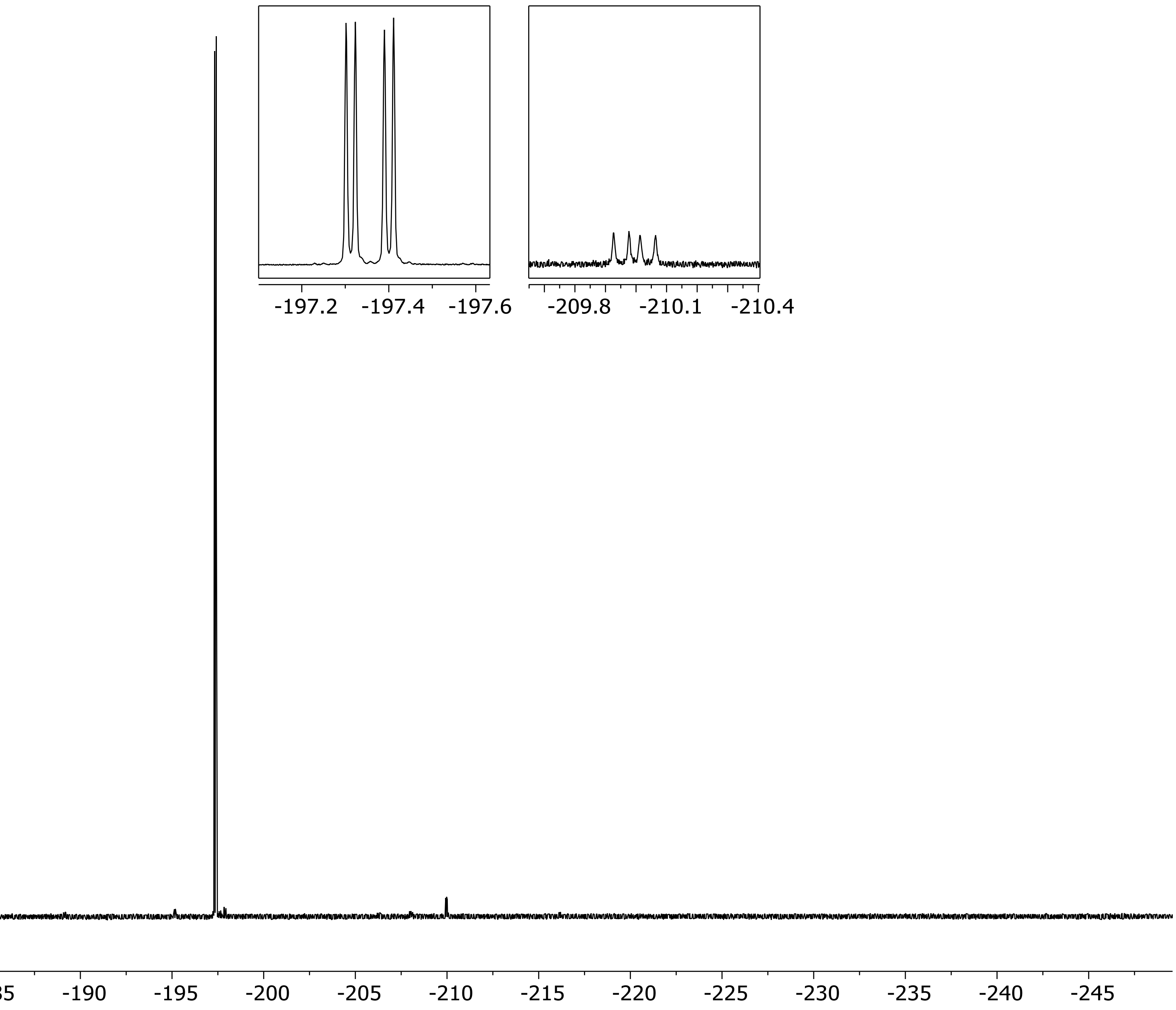

Compound **7** (^{1}H NMR, 599 MHz, $CDCl_3$)

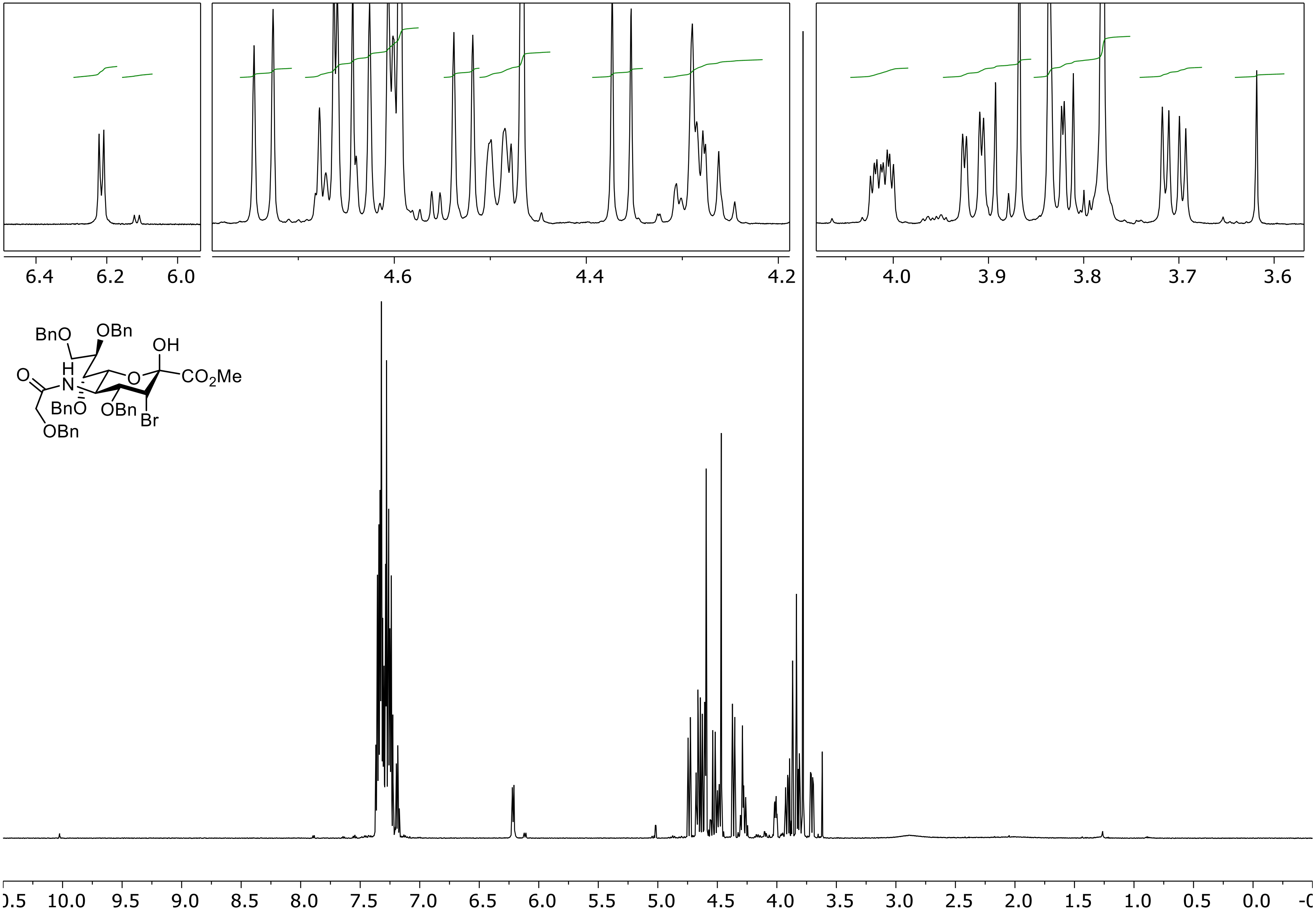

Compound **7** ($^{13}C\{^1H\}$ NMR, 151 MHz, $CDCl_3$)

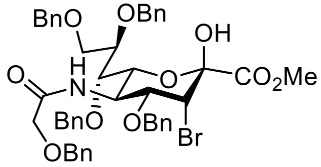

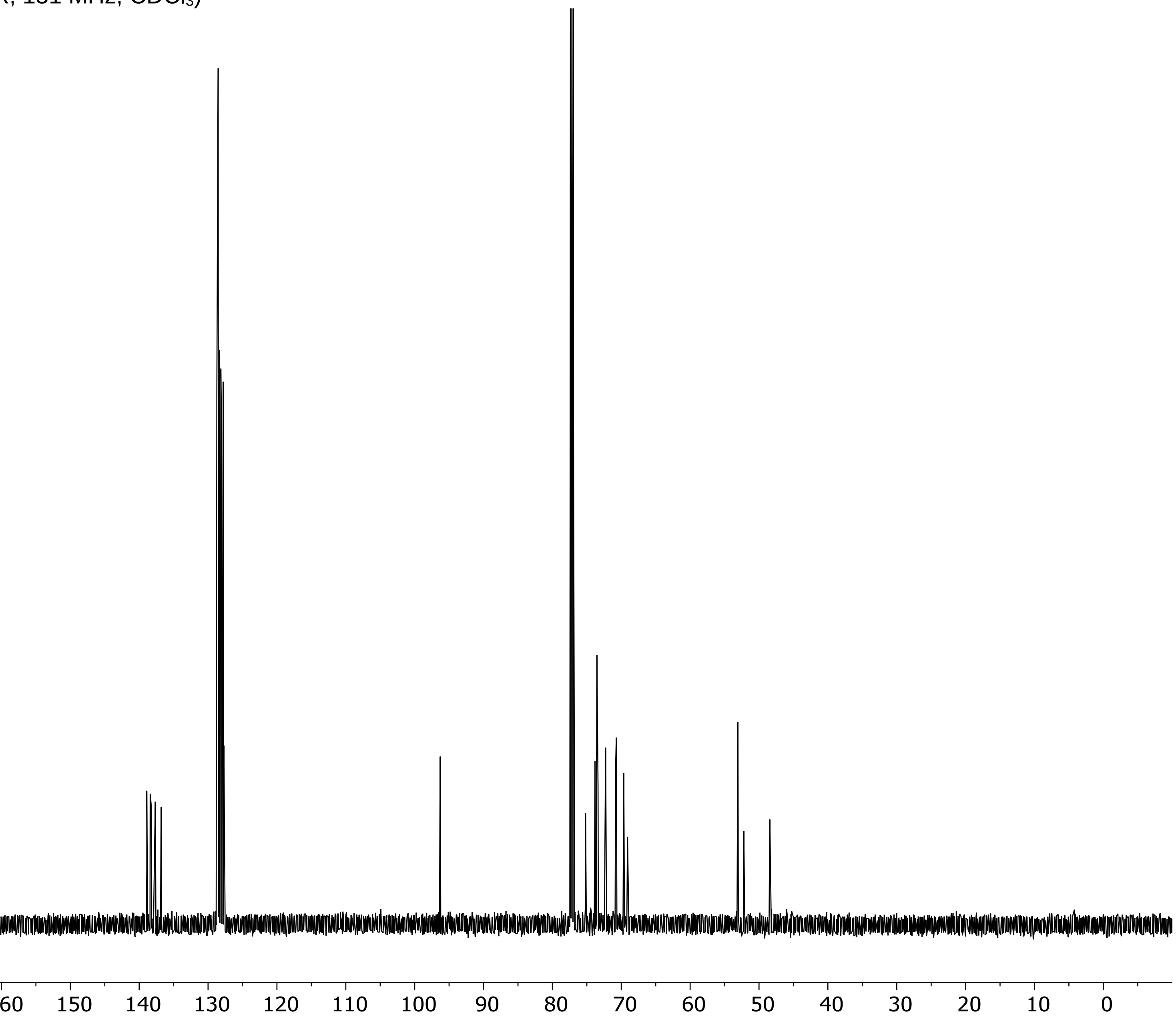

Compound **8** (^{1}H NMR, 599 MHz, $CDCl_3$)

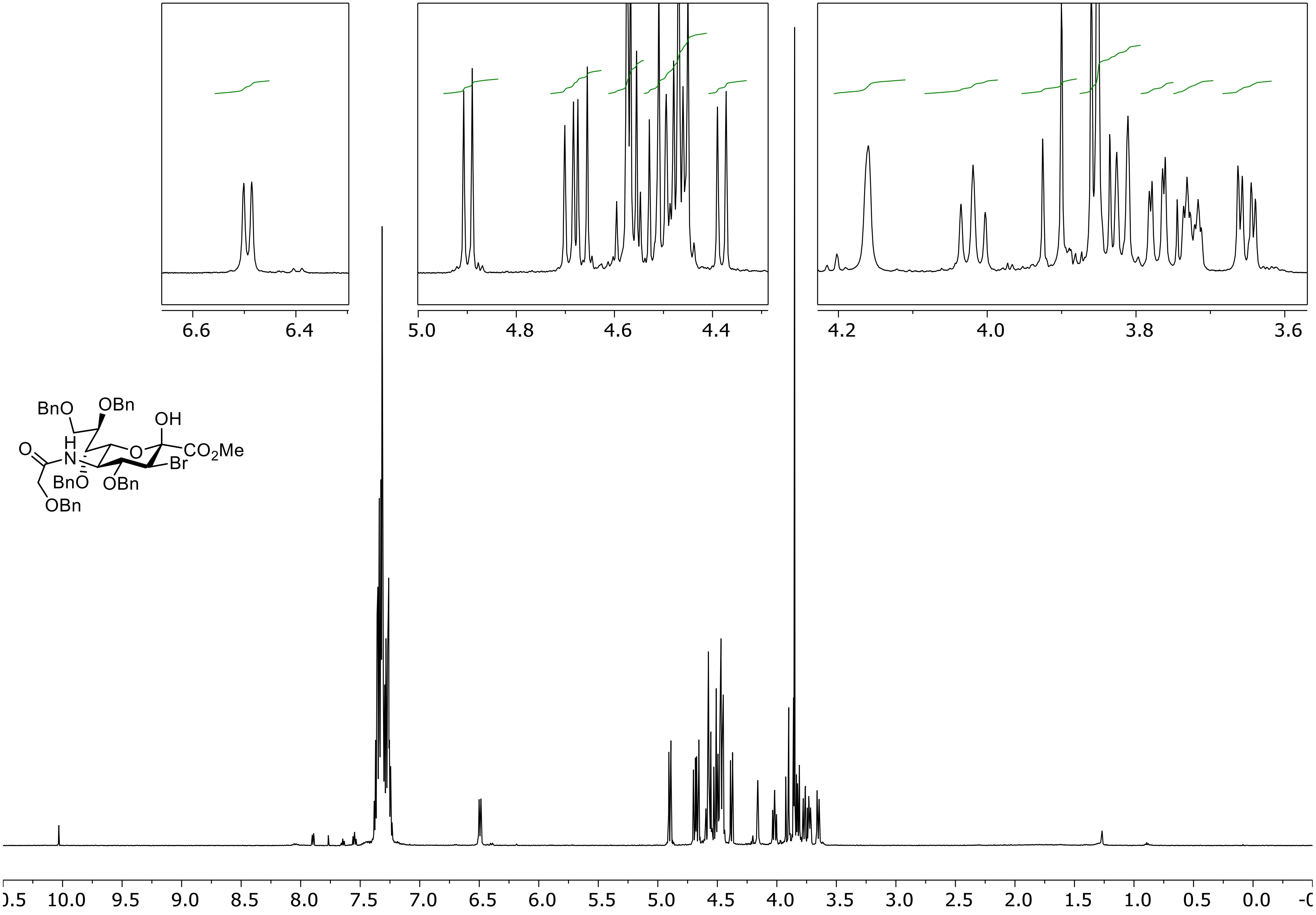

Compound **8** ($^{13}C\{^1H\}$ NMR, 151 MHz, $CDCl_3$)

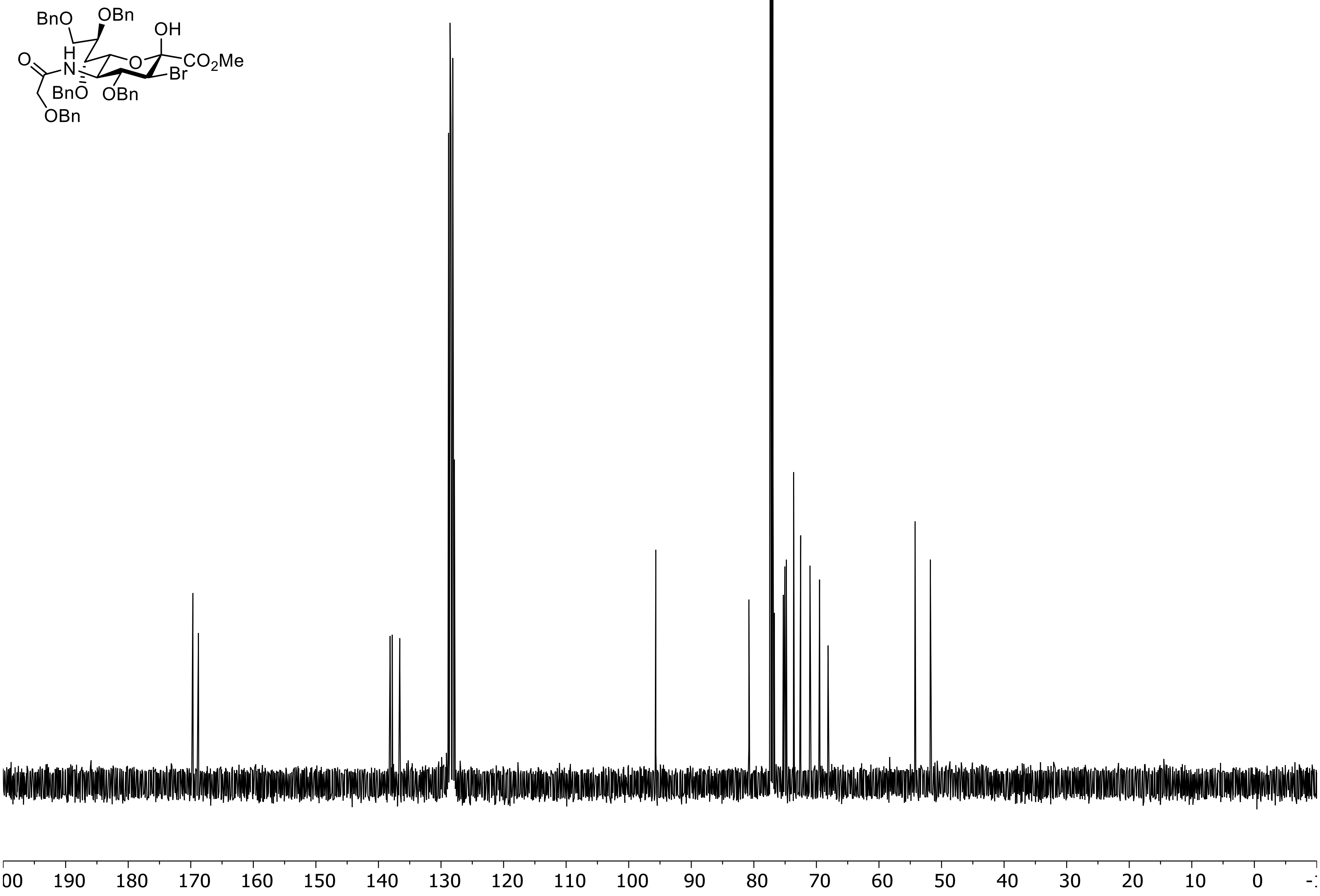

Compound **9** (^{1}H NMR, 599 MHz, CD_2Cl_2)

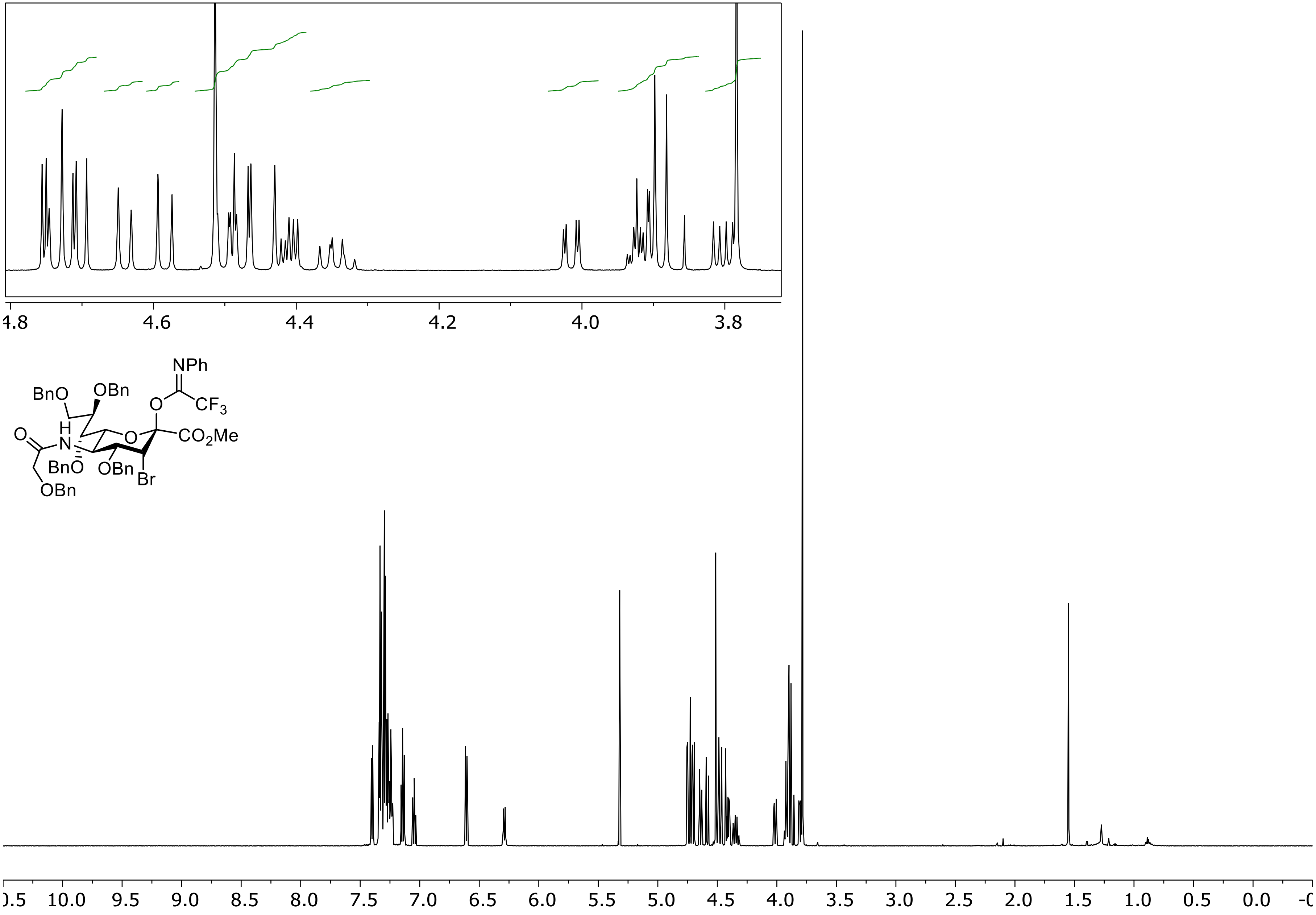

Compound **9** ($^{13}C\{^{1}H\}$ NMR, 151 MHz, CD_2Cl_2)

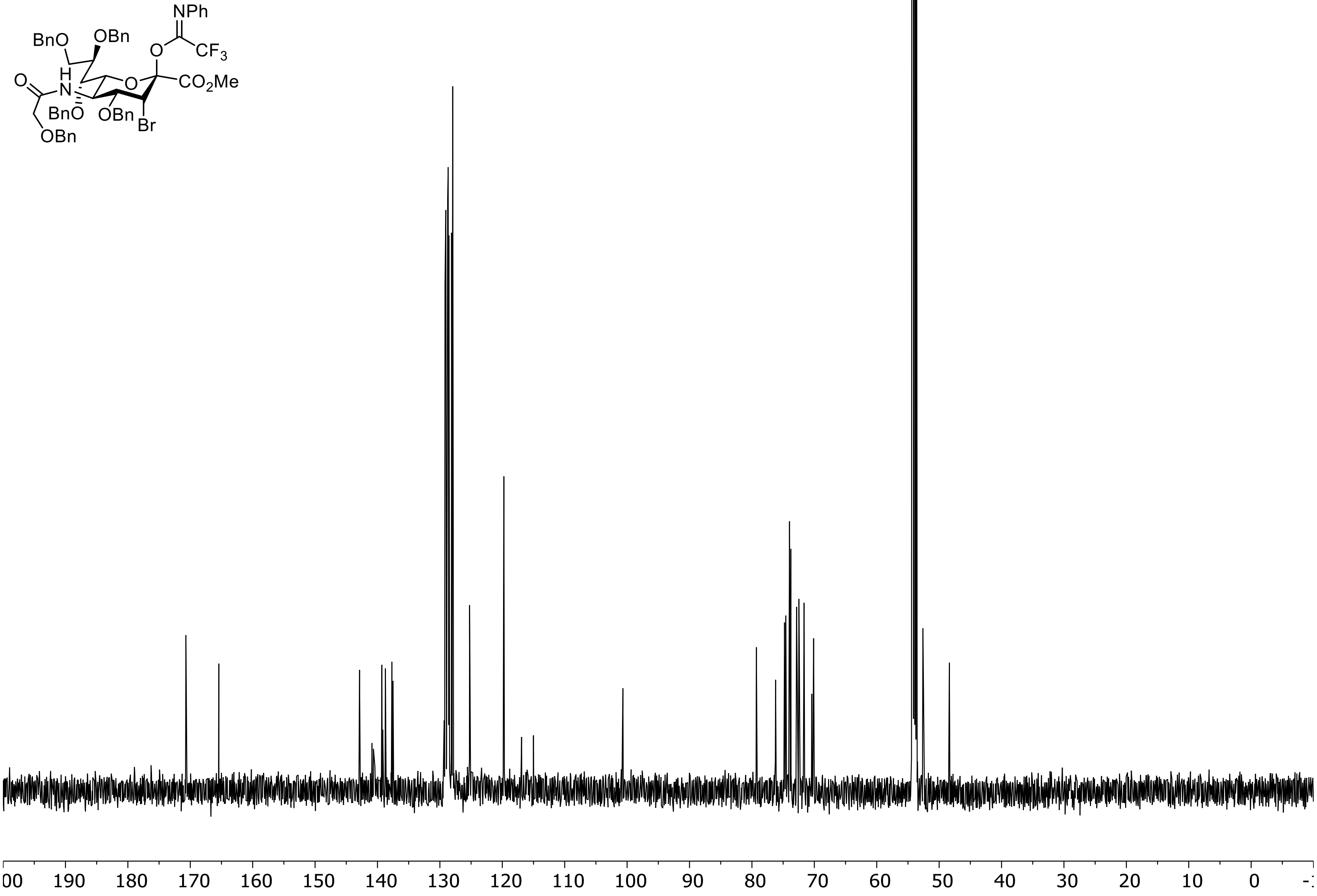

Compound **9** (^{19}F NMR, 564 MHz, CD_2Cl_2)

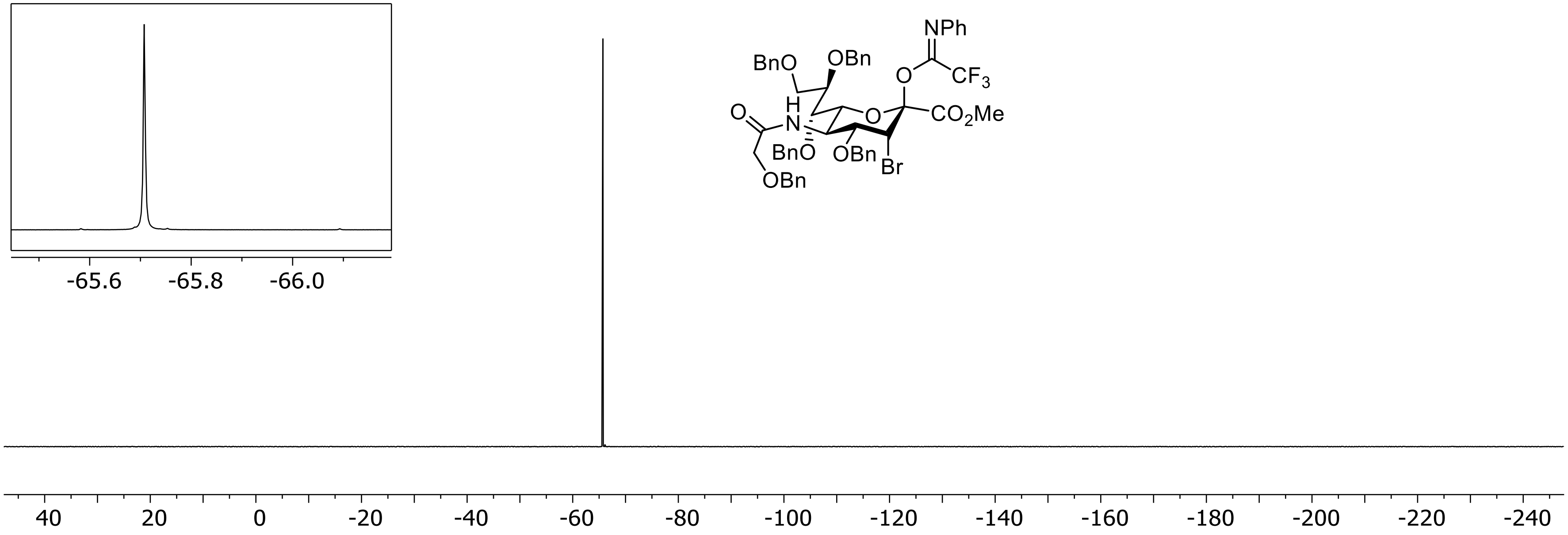

Compound **9** (HF-GHOESY, CD_2Cl_2)

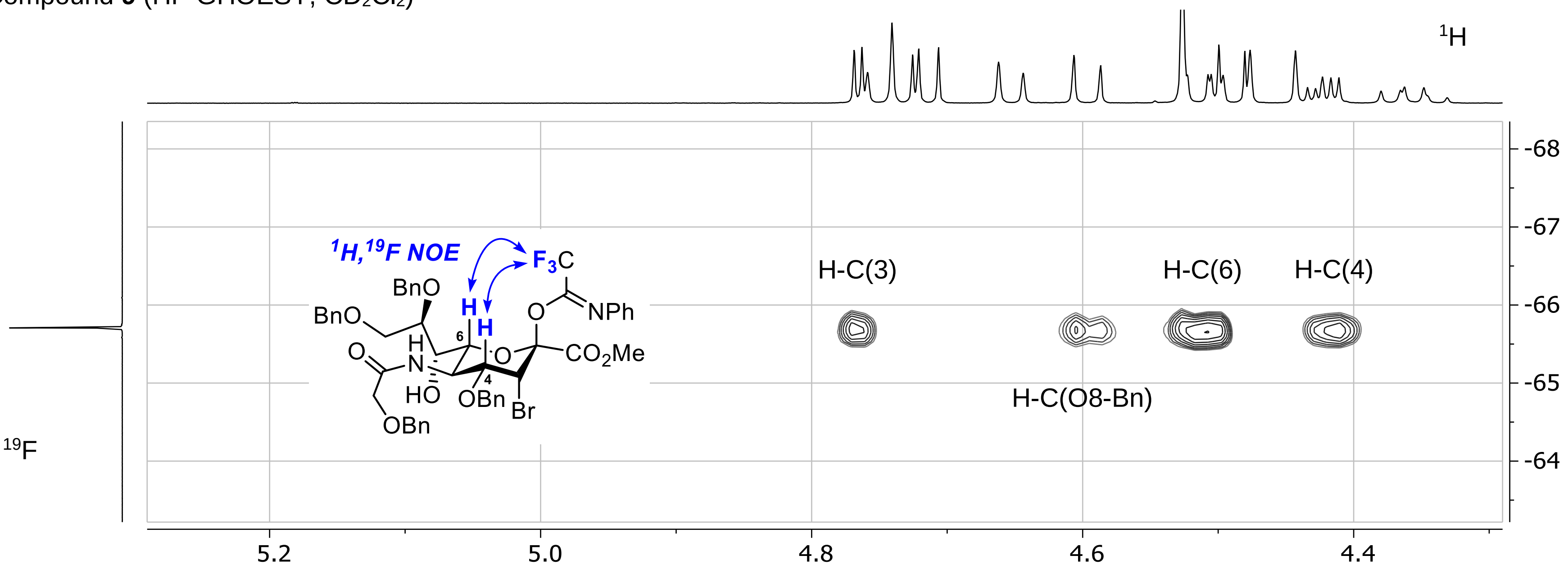

Compound **12** (^{1}H NMR, 599 MHz, CD_2Cl_2)

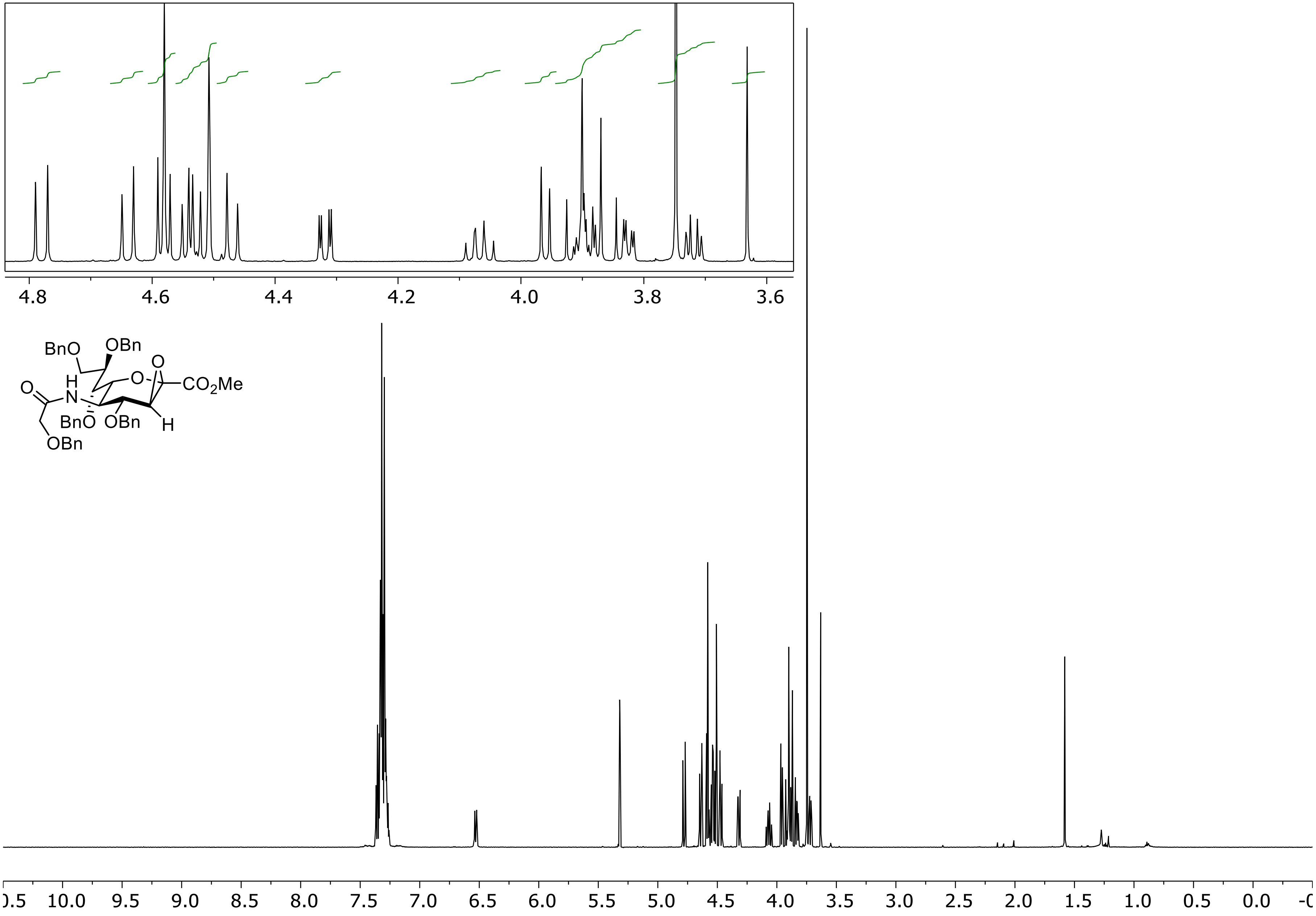

Compound **12** ($^{13}C\{^1H\}$ NMR, 151 MHz, CD_2Cl_2)

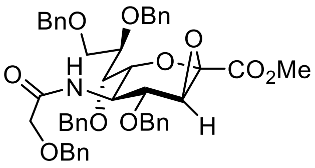

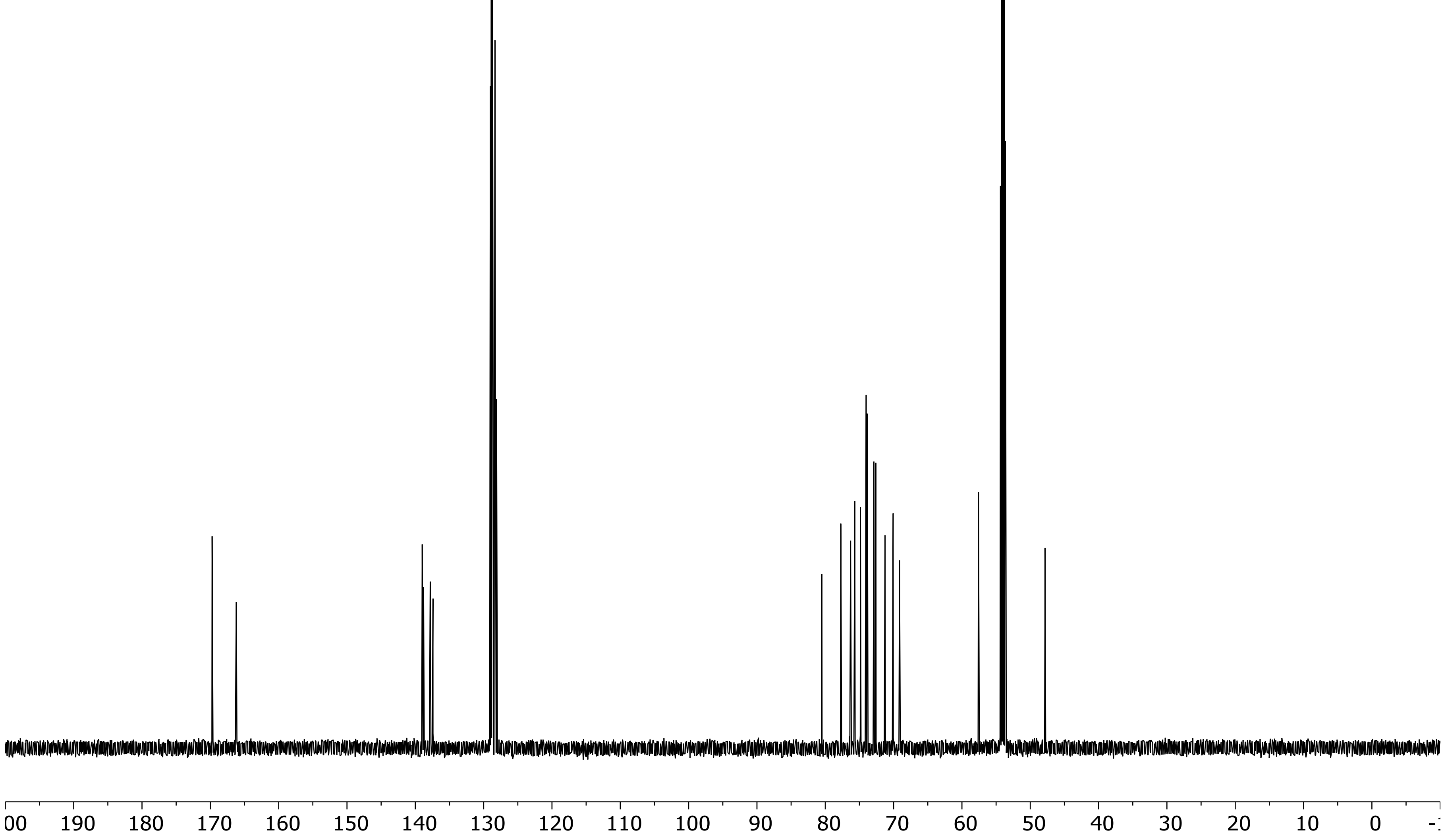

Compound **10** (^{1}H NMR, 599 MHz, CD_2Cl_2)

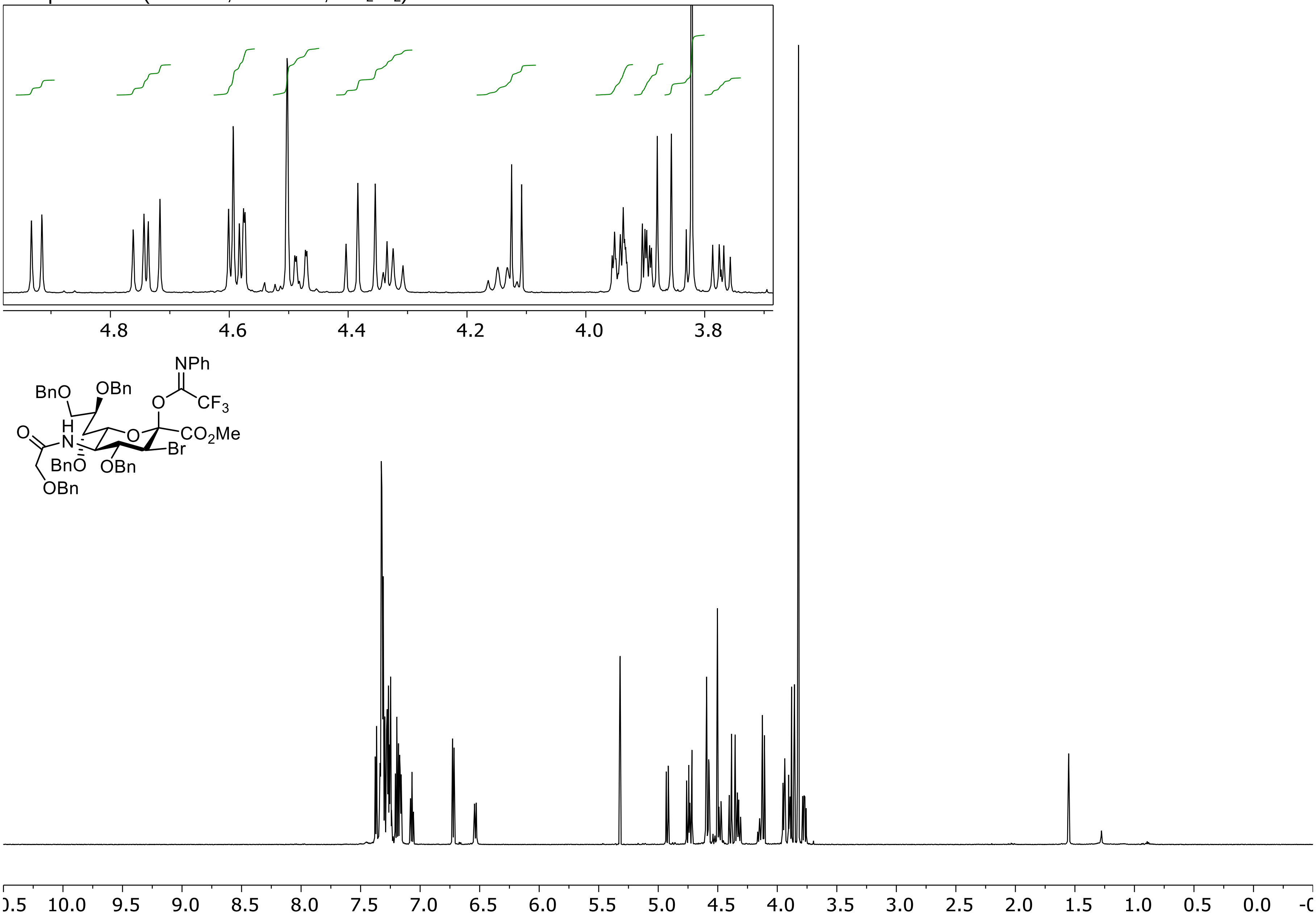

Compound **10** ($^{13}C\{^{1}H\}$ NMR, 151 MHz, CD_2Cl_2)

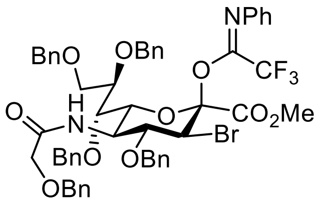

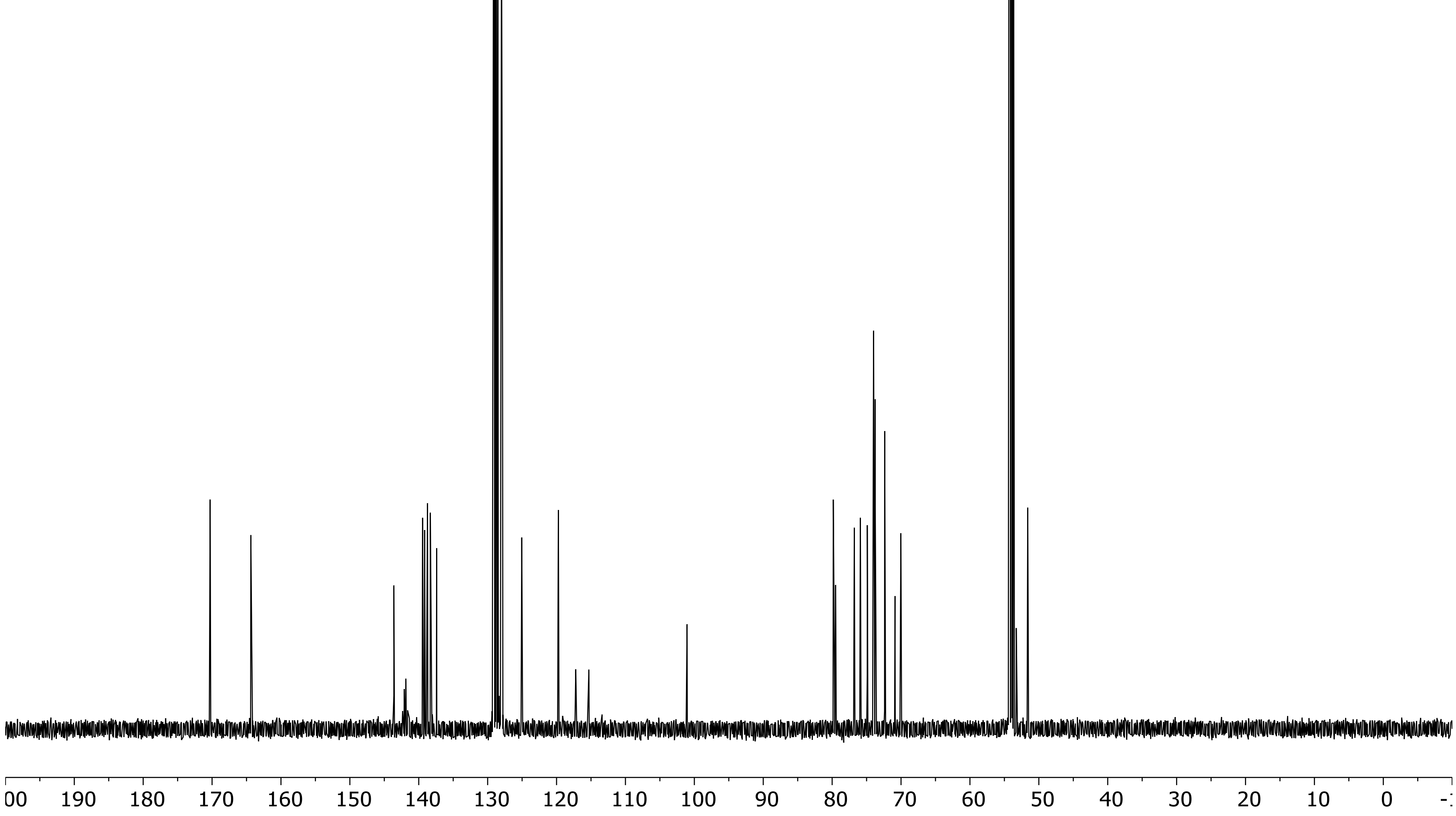

Compound **10** (^{19}F NMR, 564 MHz, CD_2Cl_2)

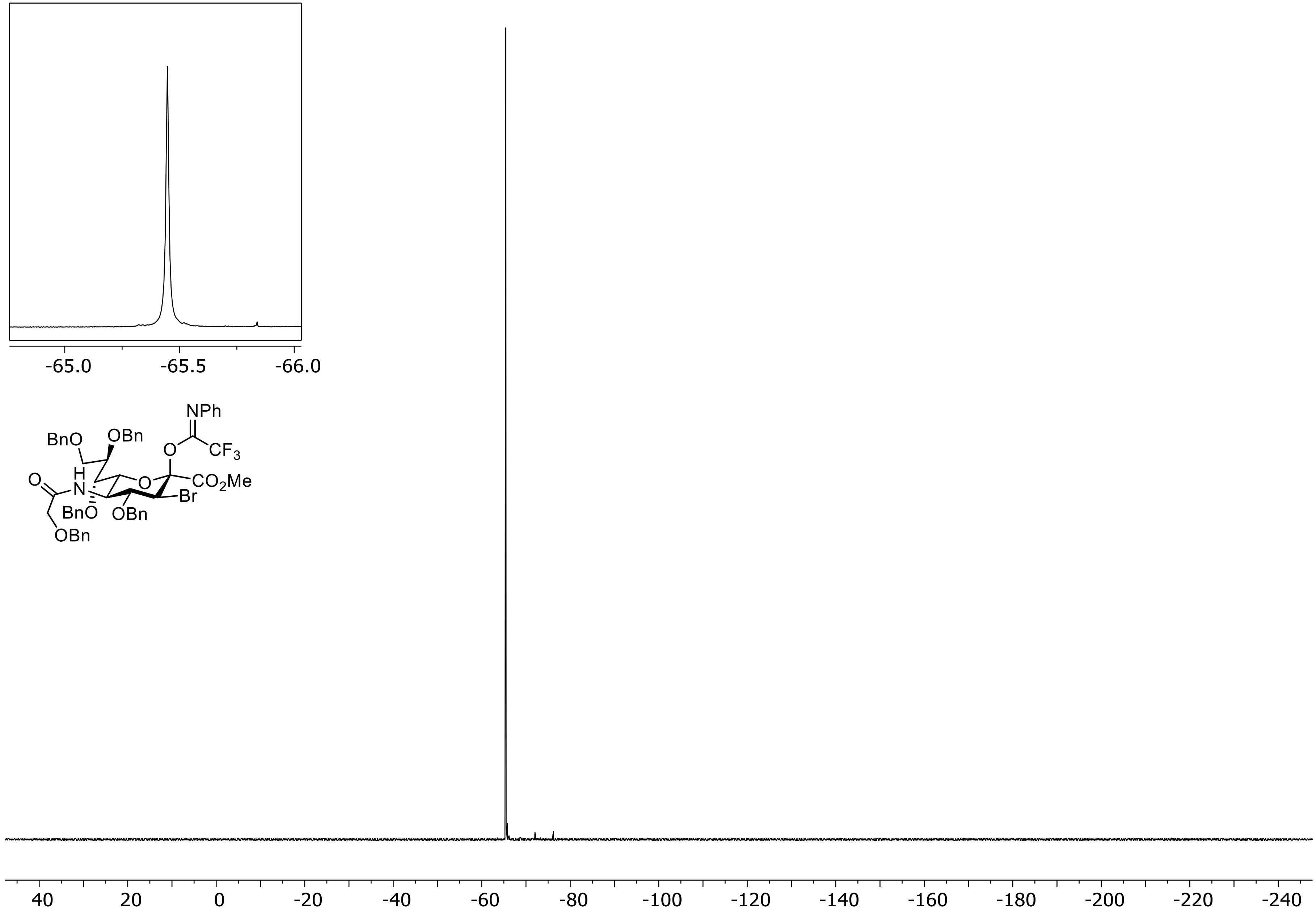

Compound **11** (^{1}H NMR, 599 MHz, CD_2Cl_2)

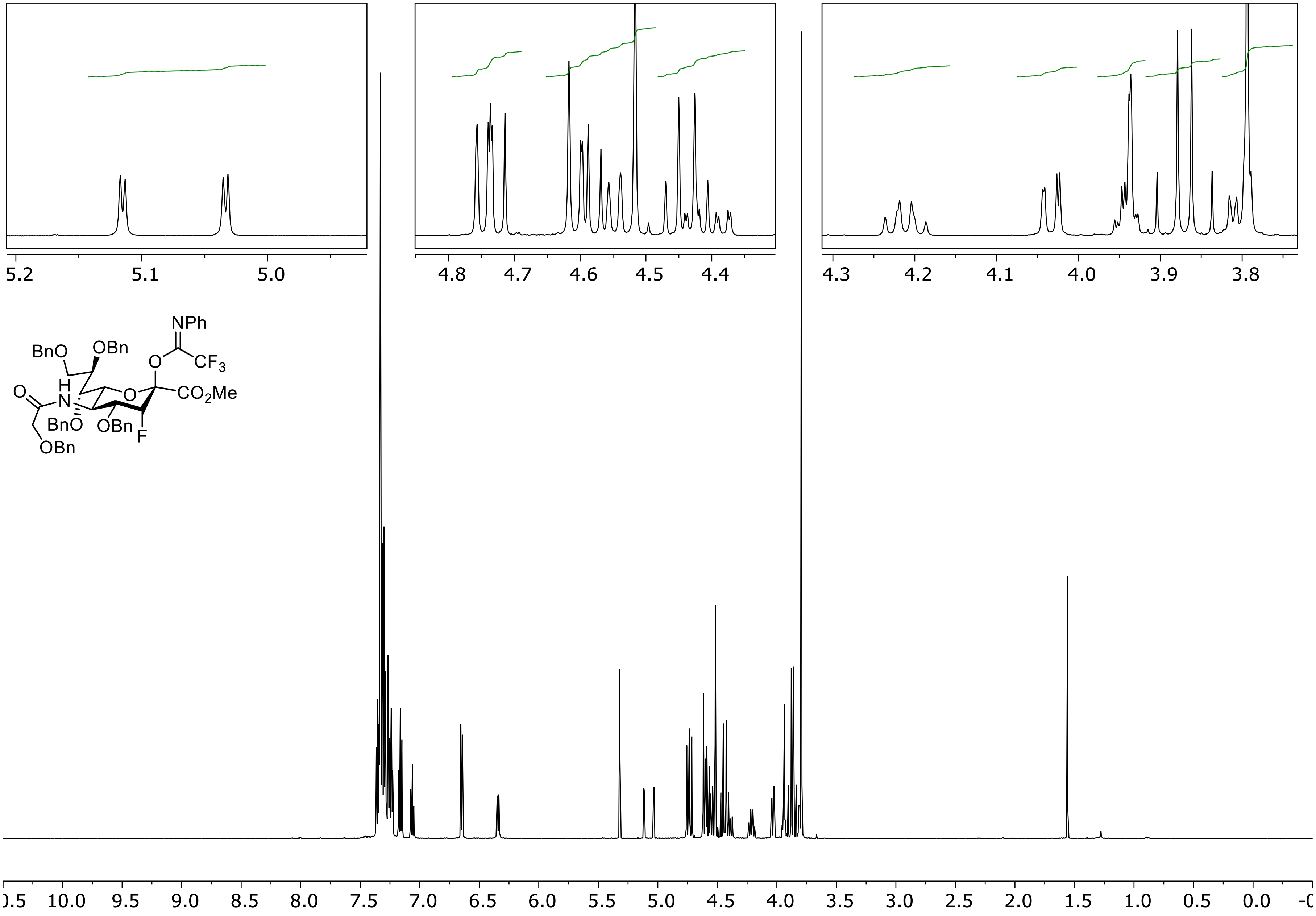

Compound **11** ($^{13}C\{^{1}H\}$ NMR, 151 MHz, CD_2Cl_2)

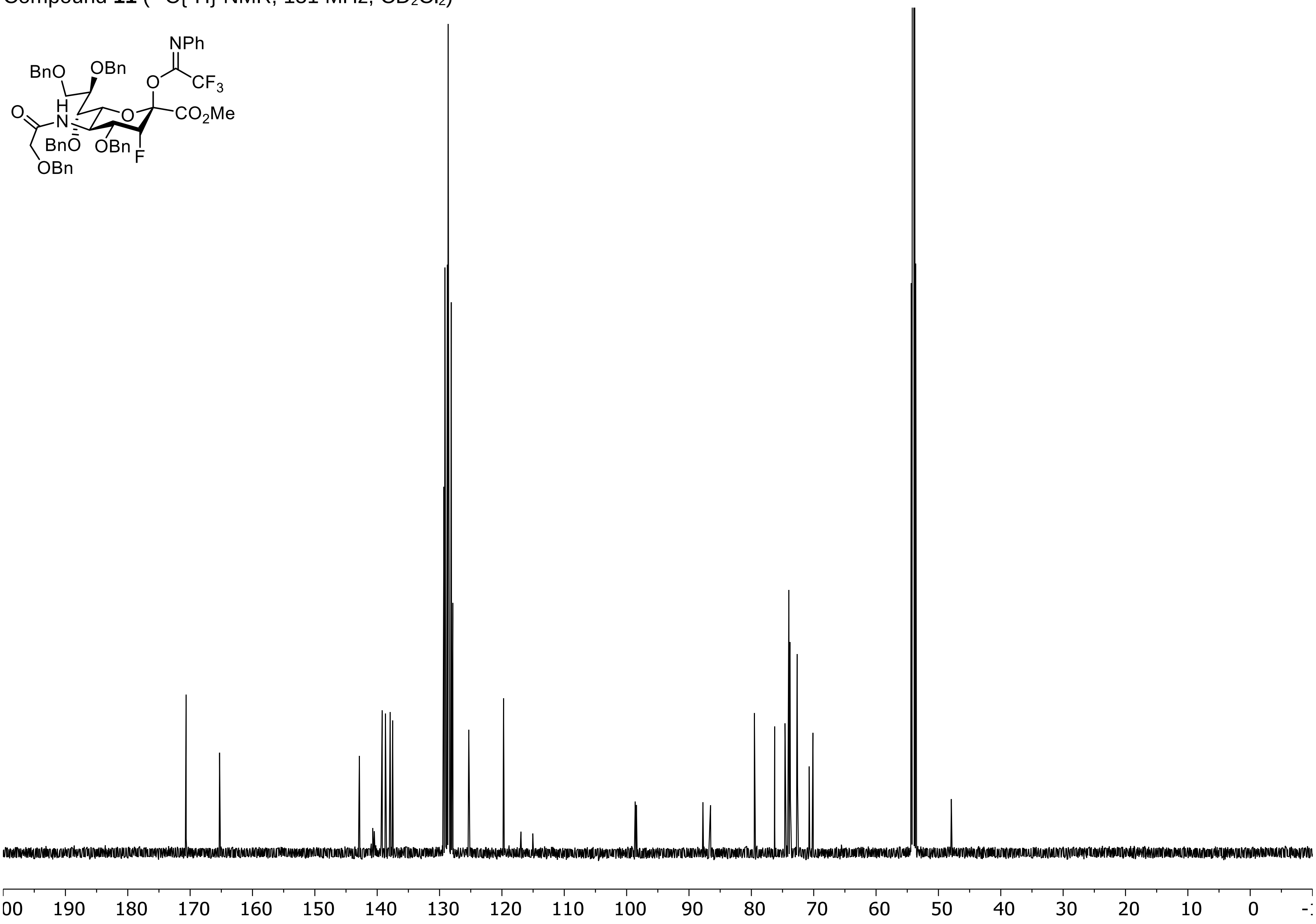

Compound **11** (^{19}F NMR, 564 MHz, CD_2Cl_2)

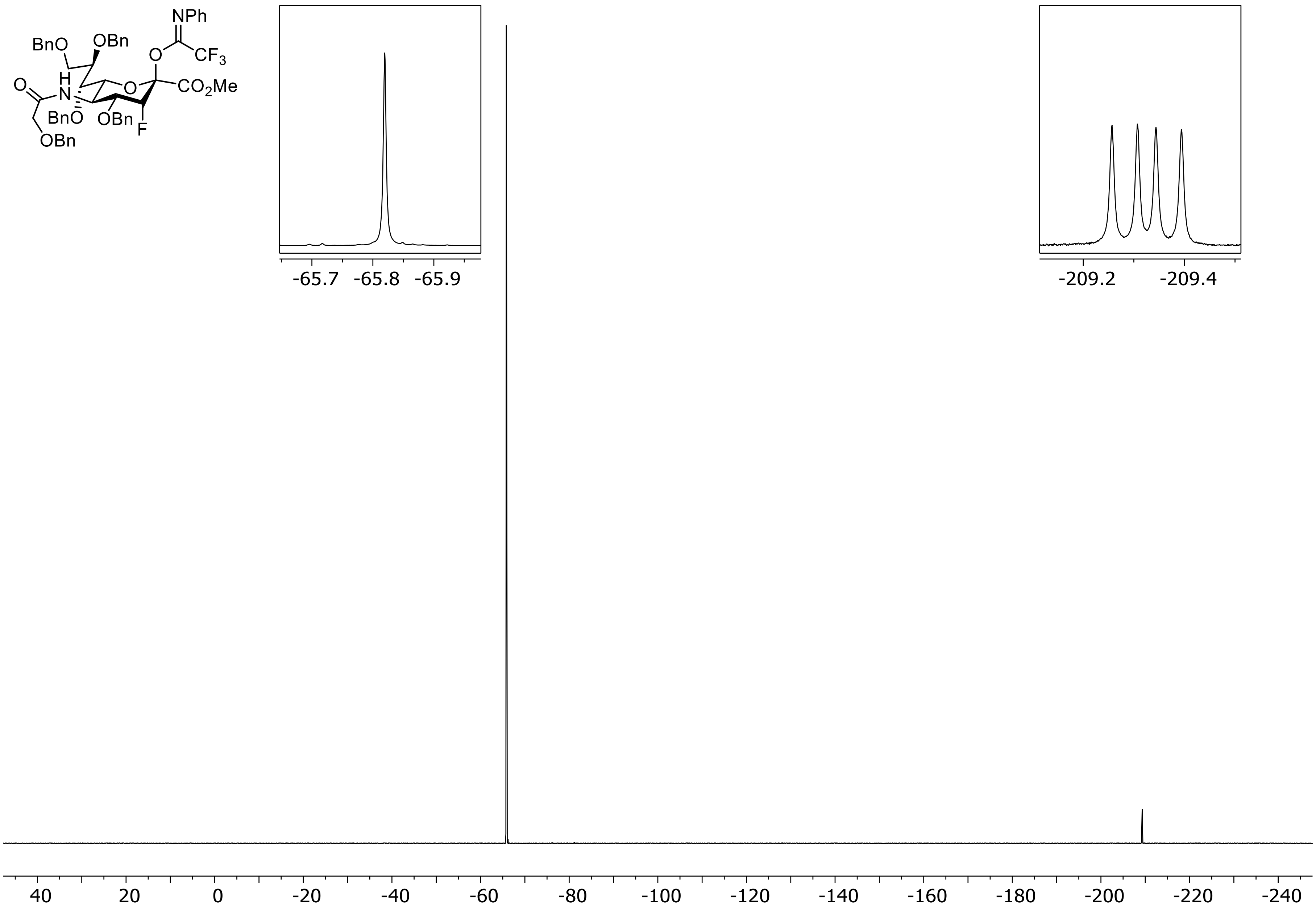

Compound **S3** (^{1}H NMR, 400 MHz, CD_3OD)

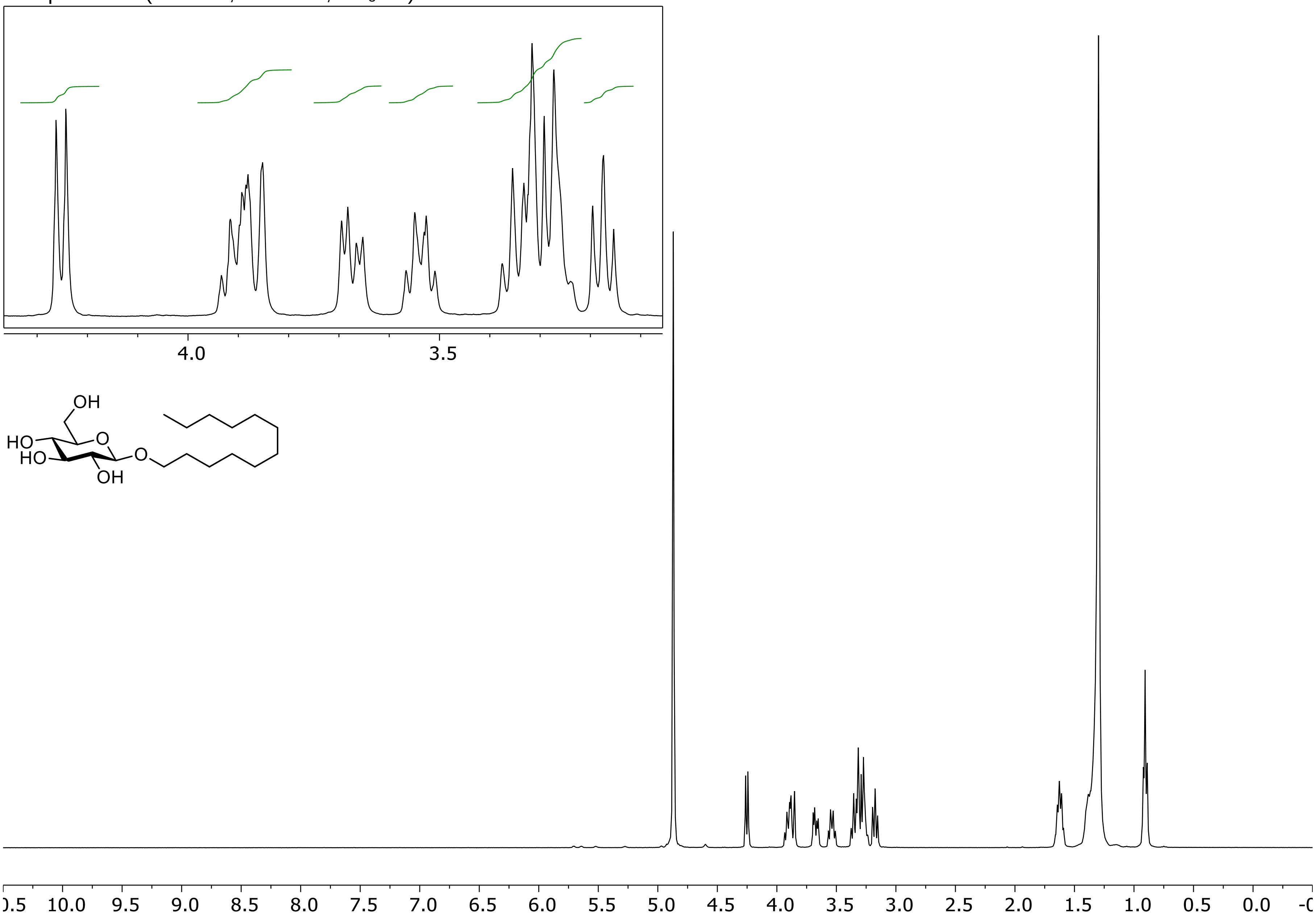

Compound **S3** ($^{13}C\{^1H\}$ NMR, 101 MHz, CD_3OD)

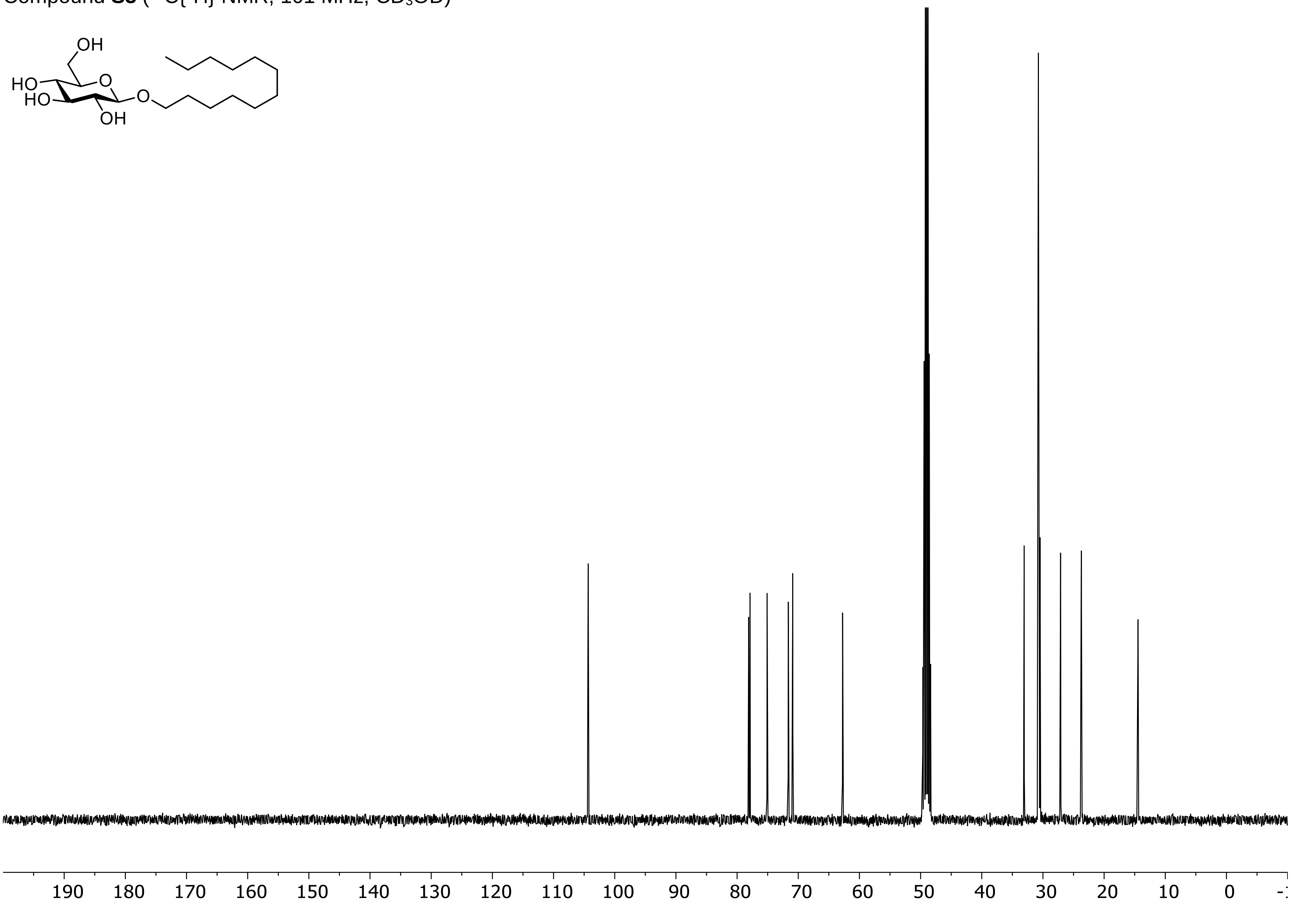

Compound **S4** (^{1}H NMR, 599 MHz, CD_2Cl_2)

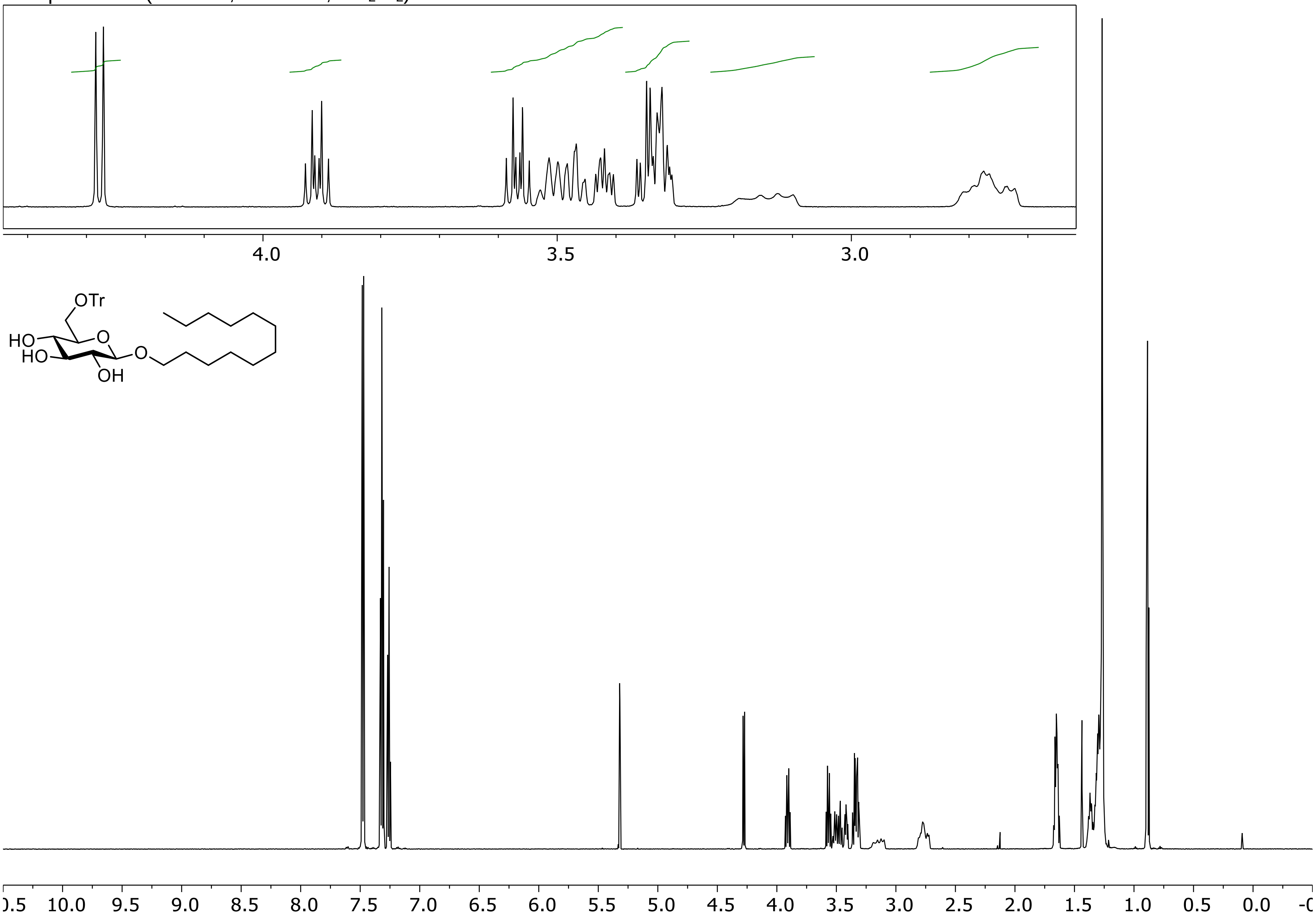

Compound **S4** ($^{13}C\{^{1}H\}$ NMR, 151 MHz, CD_2Cl_2)

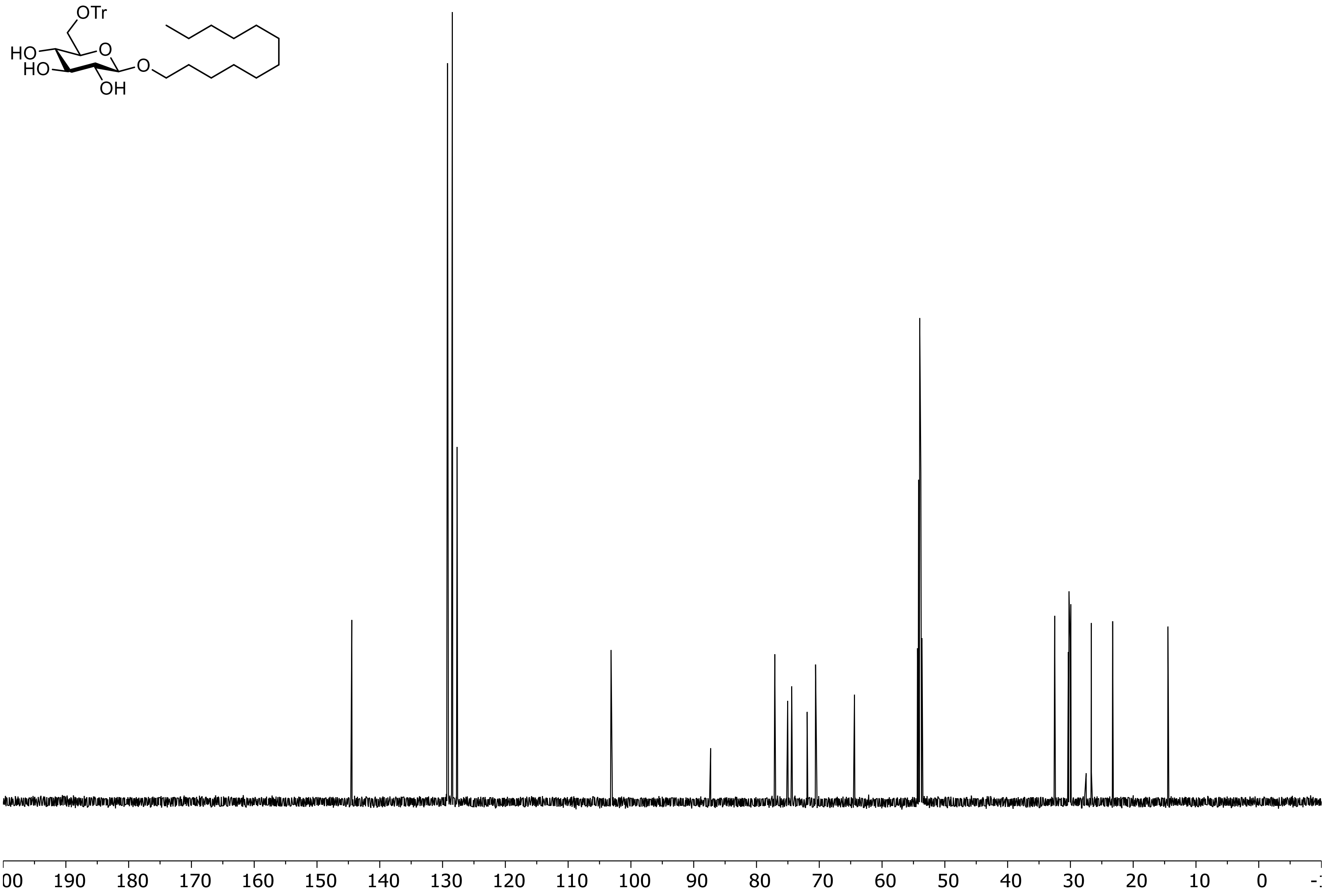

Compound **S5** (^{1}H NMR, 500 MHz, CD_2Cl_2)

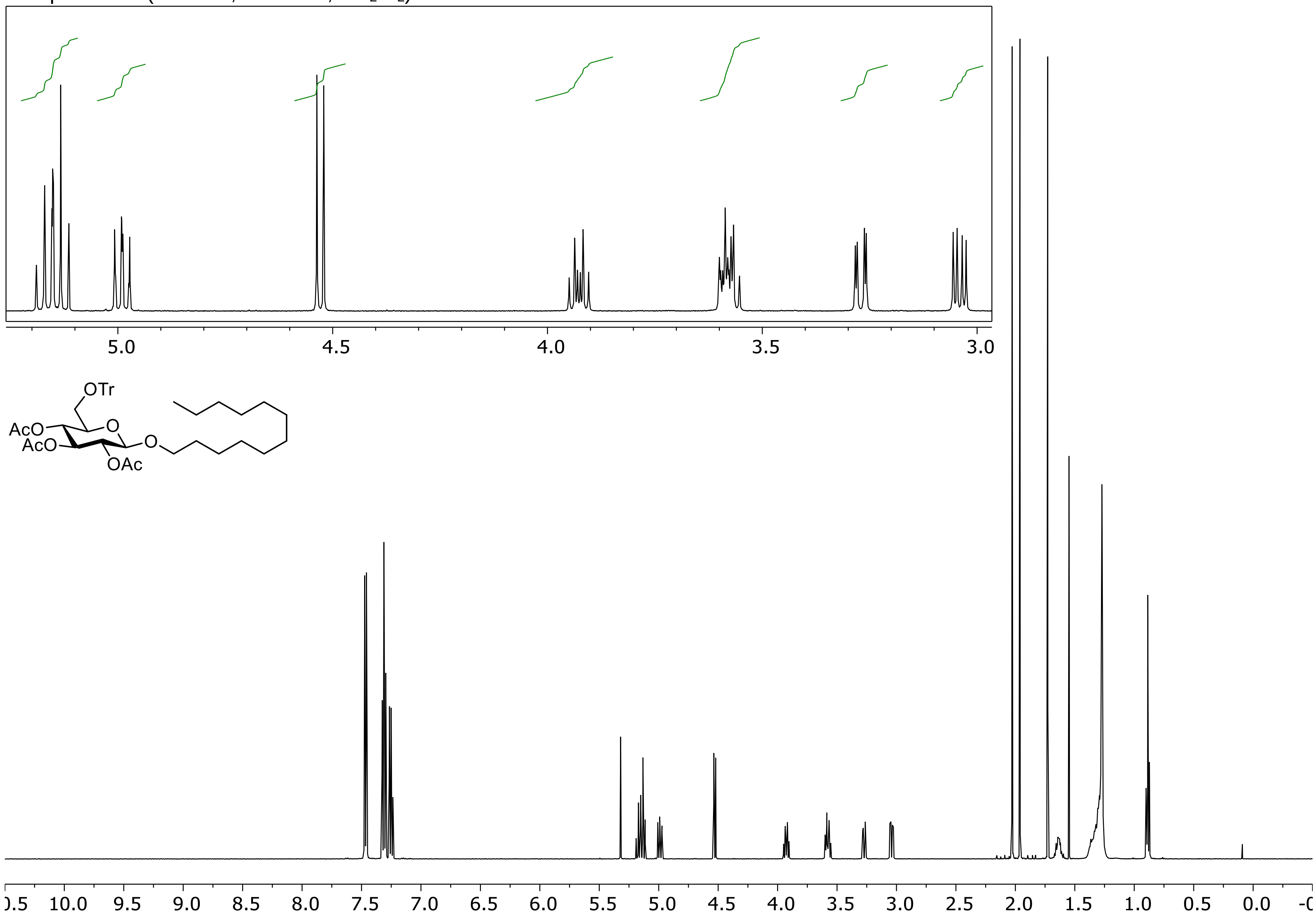

Compound **S5** ($^{13}C\{^1H\}$ NMR, 126 MHz, CD_2Cl_2)

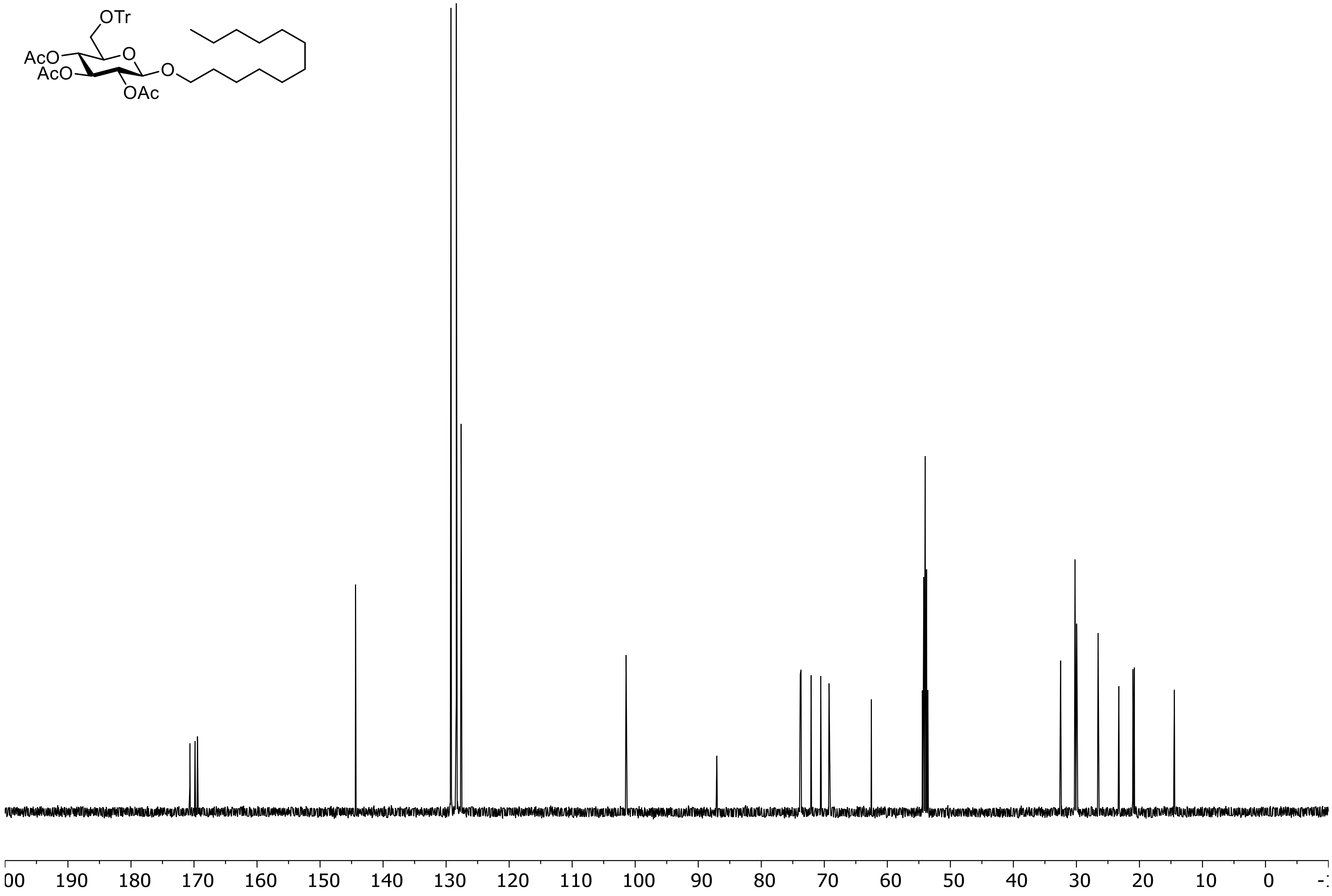

Compound **13** (^{1}H NMR, 500 MHz, CD_2Cl_2)

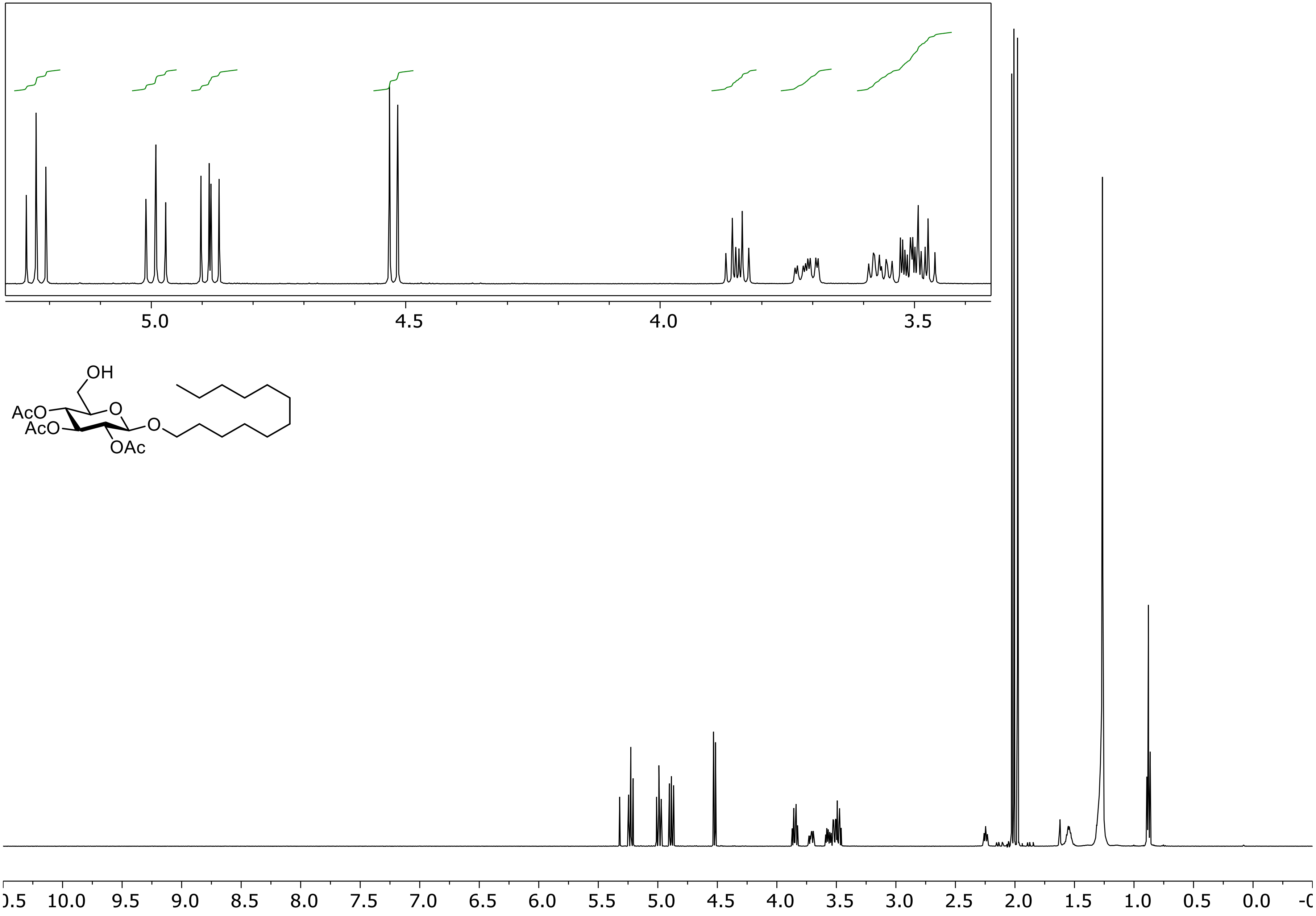

Compound **13** ($^{13}C\{^1H\}$ NMR, 126 MHz, CD_2Cl_2)

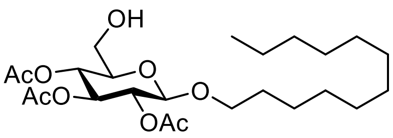

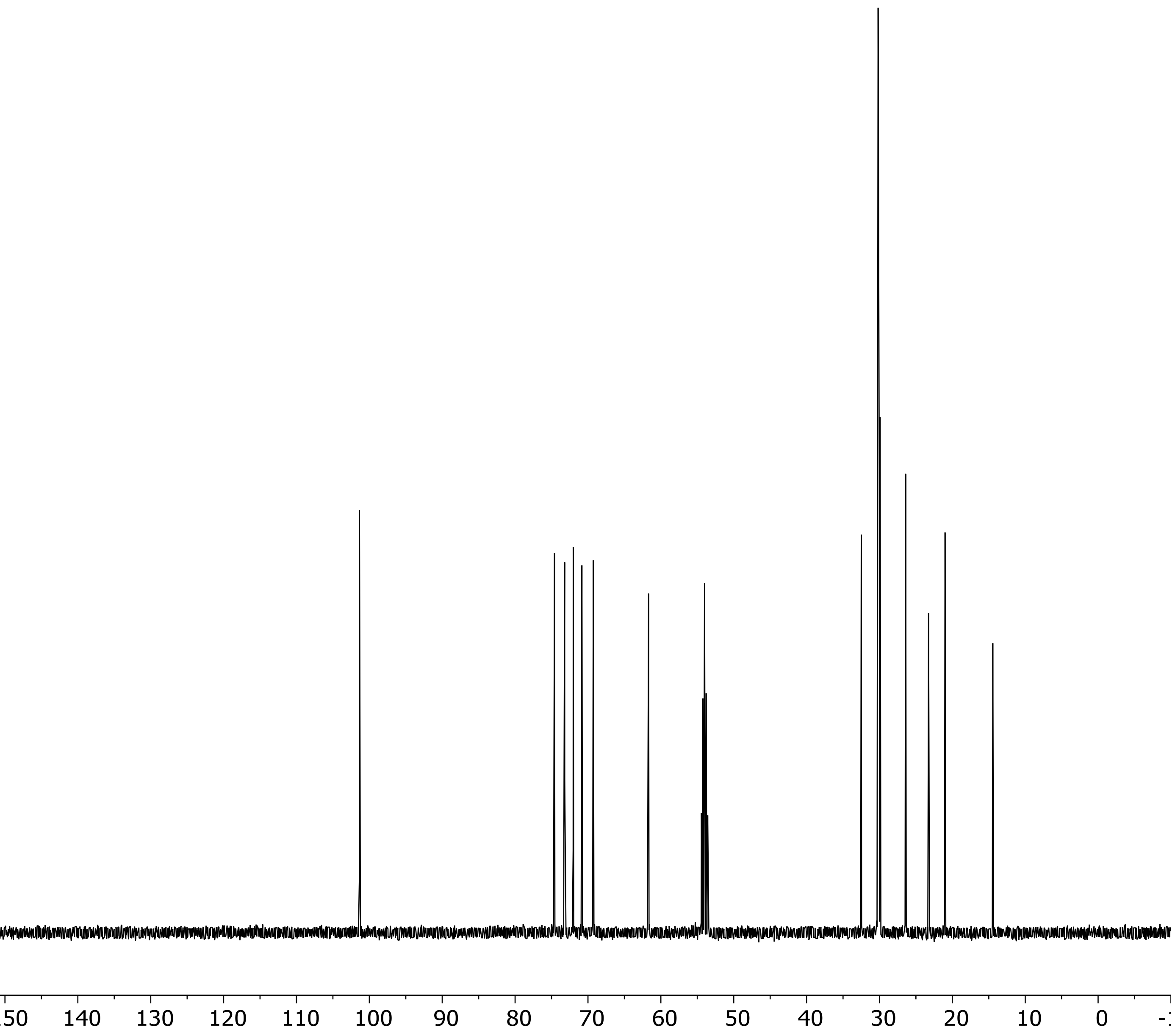

Compound **24** (^{1}H NMR, 599 MHz, $CDCl_3$)

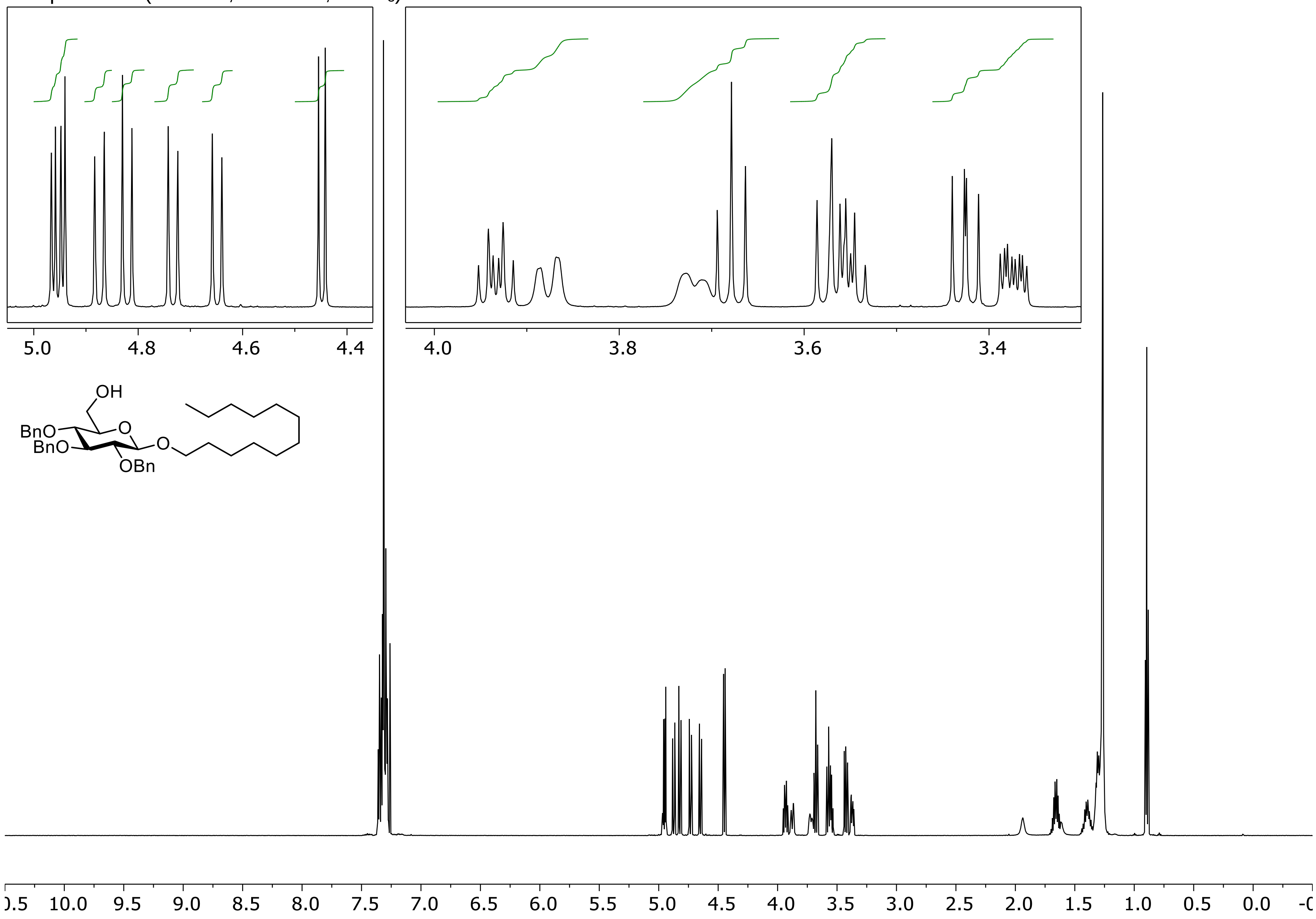

Compound **24** ($^{13}C\{^{1}H\}$ NMR, 151 MHz, $CDCl_3$)

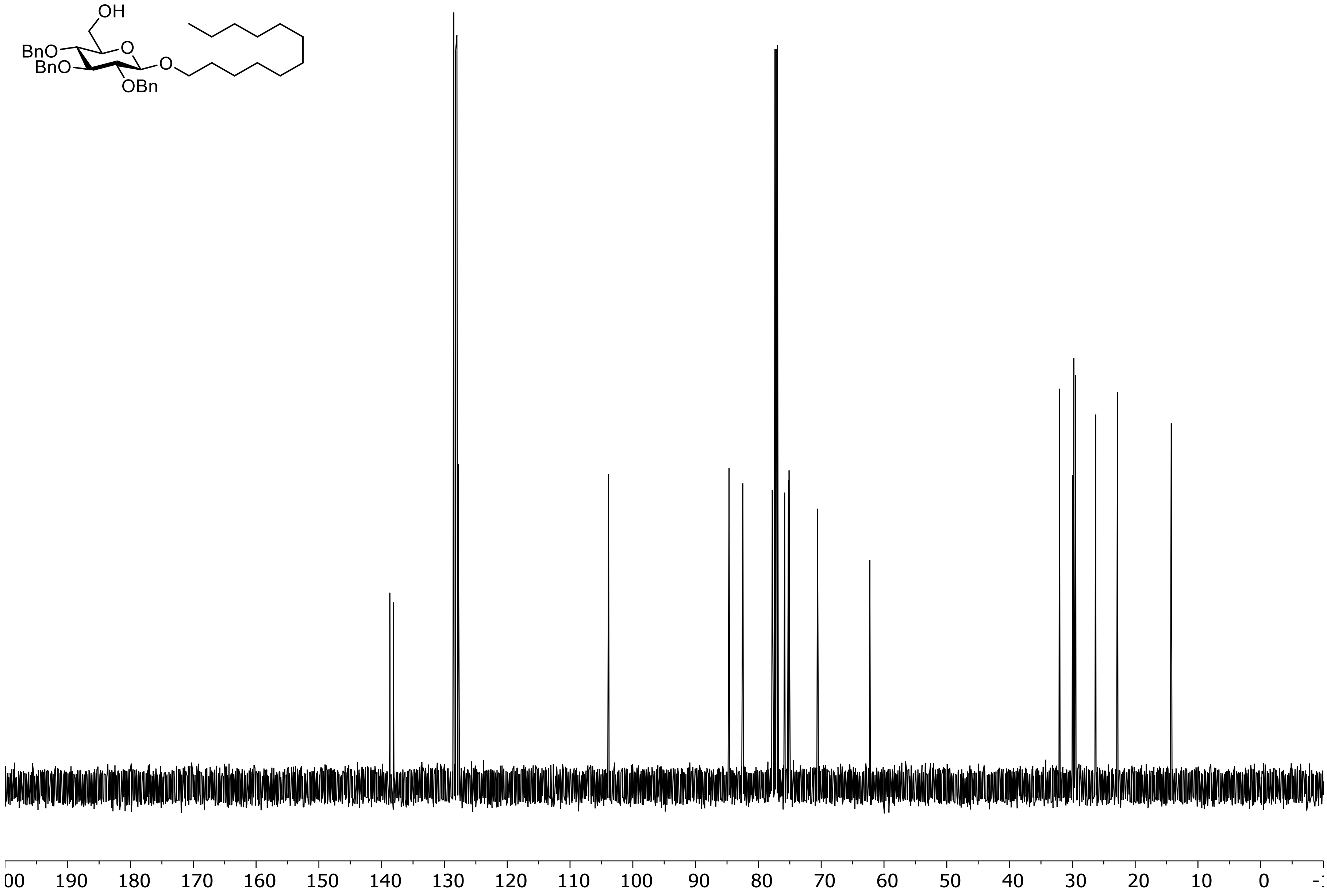

Compound **14** (^{1}H NMR, 599 MHz, $CDCl_3$)

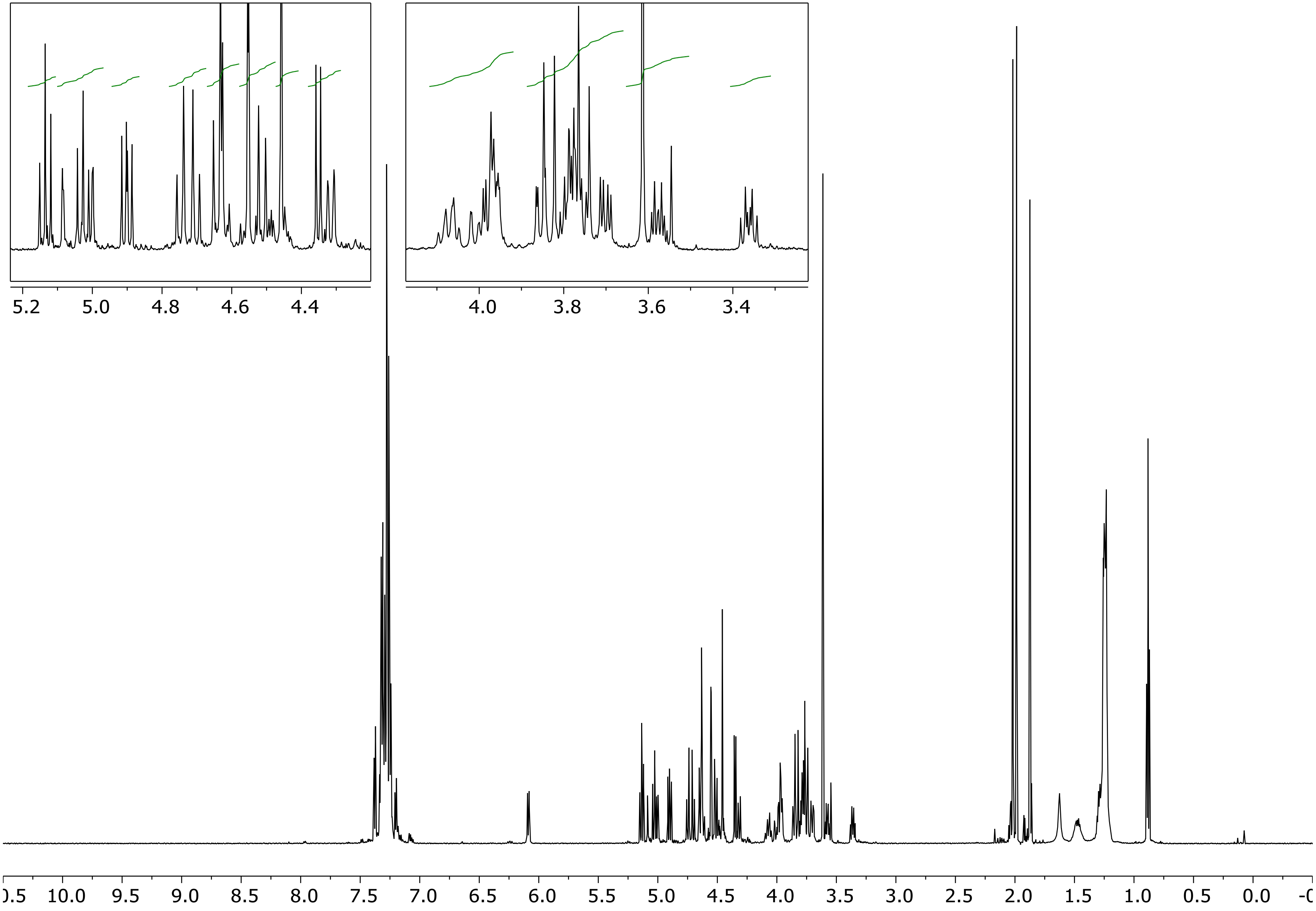

Compound **14** ($^{13}C\{^{1}H\}$ NMR, 151 MHz, $CDCl_3$)

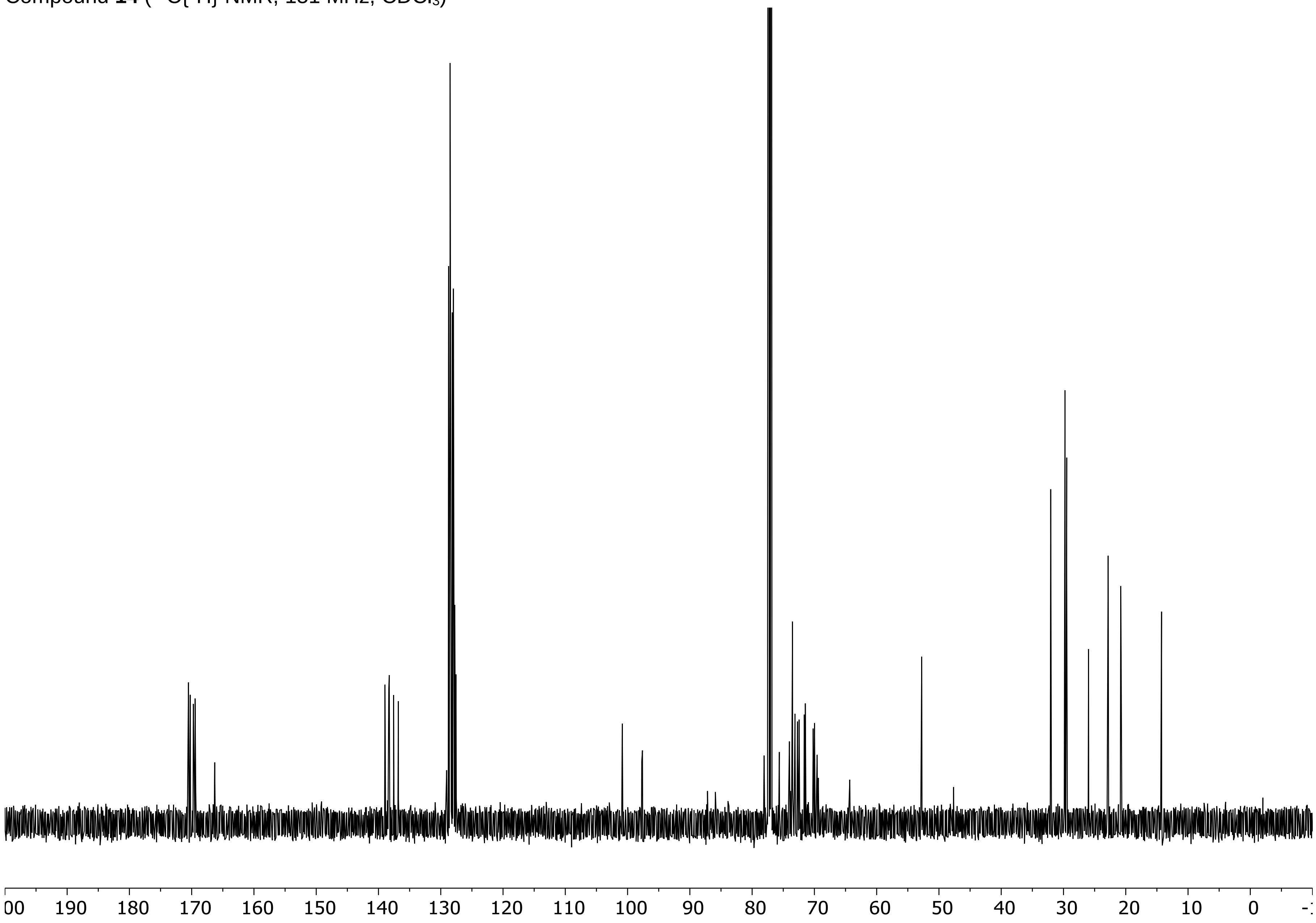

Compound **14** (^{19}F NMR, 564 MHz, $CDCl_3$)

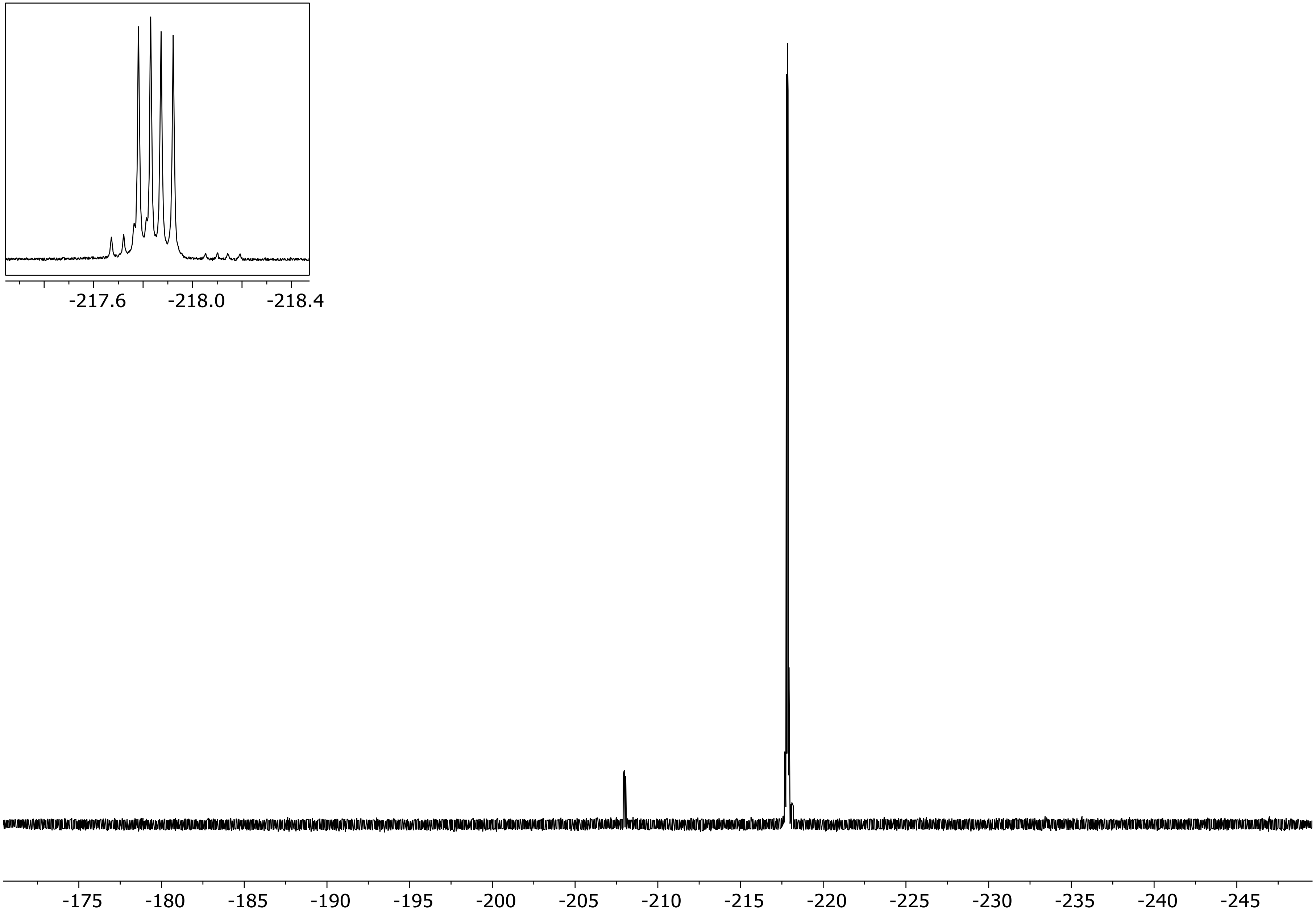

Compound **15** (^{1}H NMR, 599 MHz, $CDCl_3$)

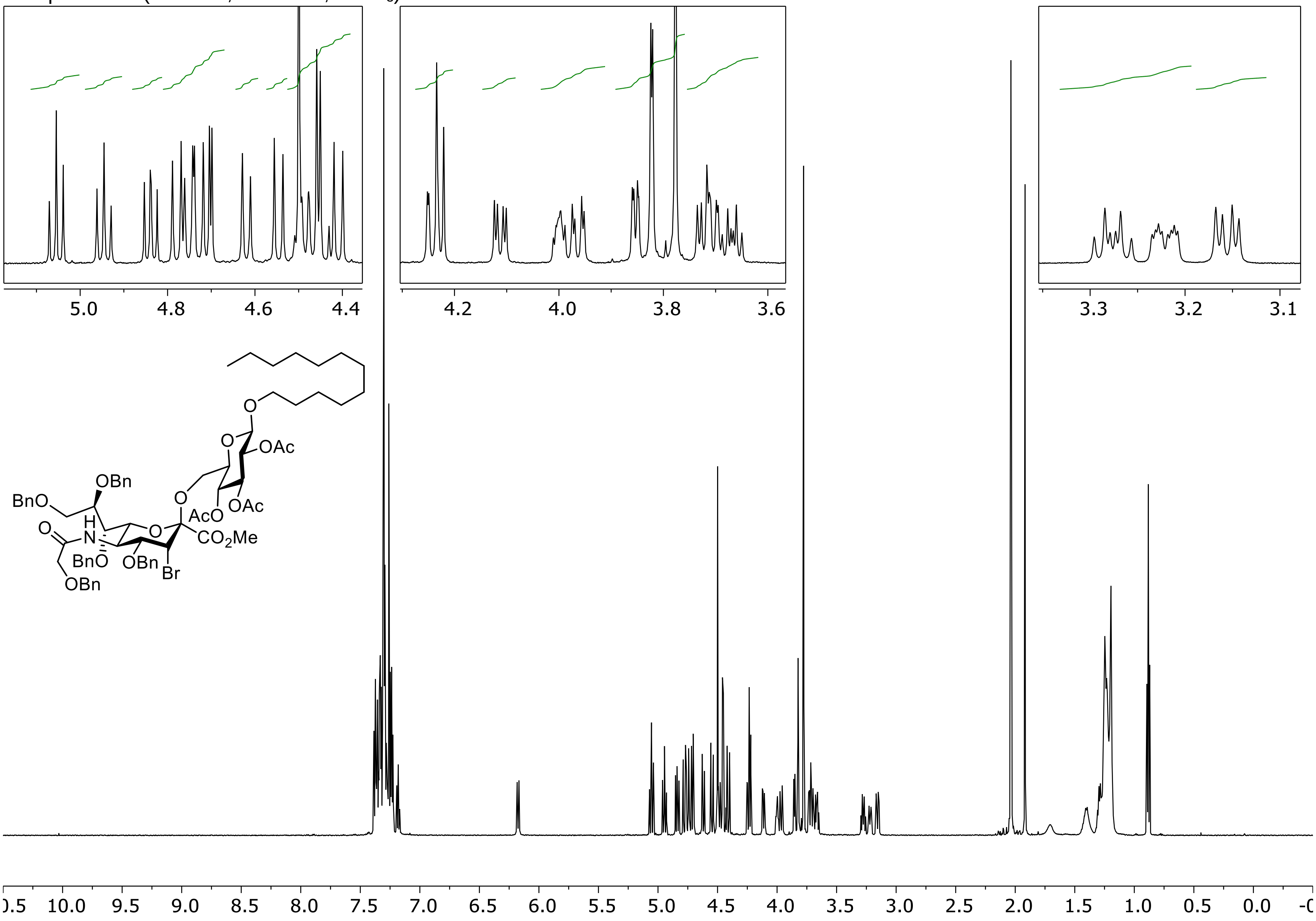

Compound **15** ($^{13}C\{^{1}H\}$ NMR, 151 MHz, $CDCl_3$)

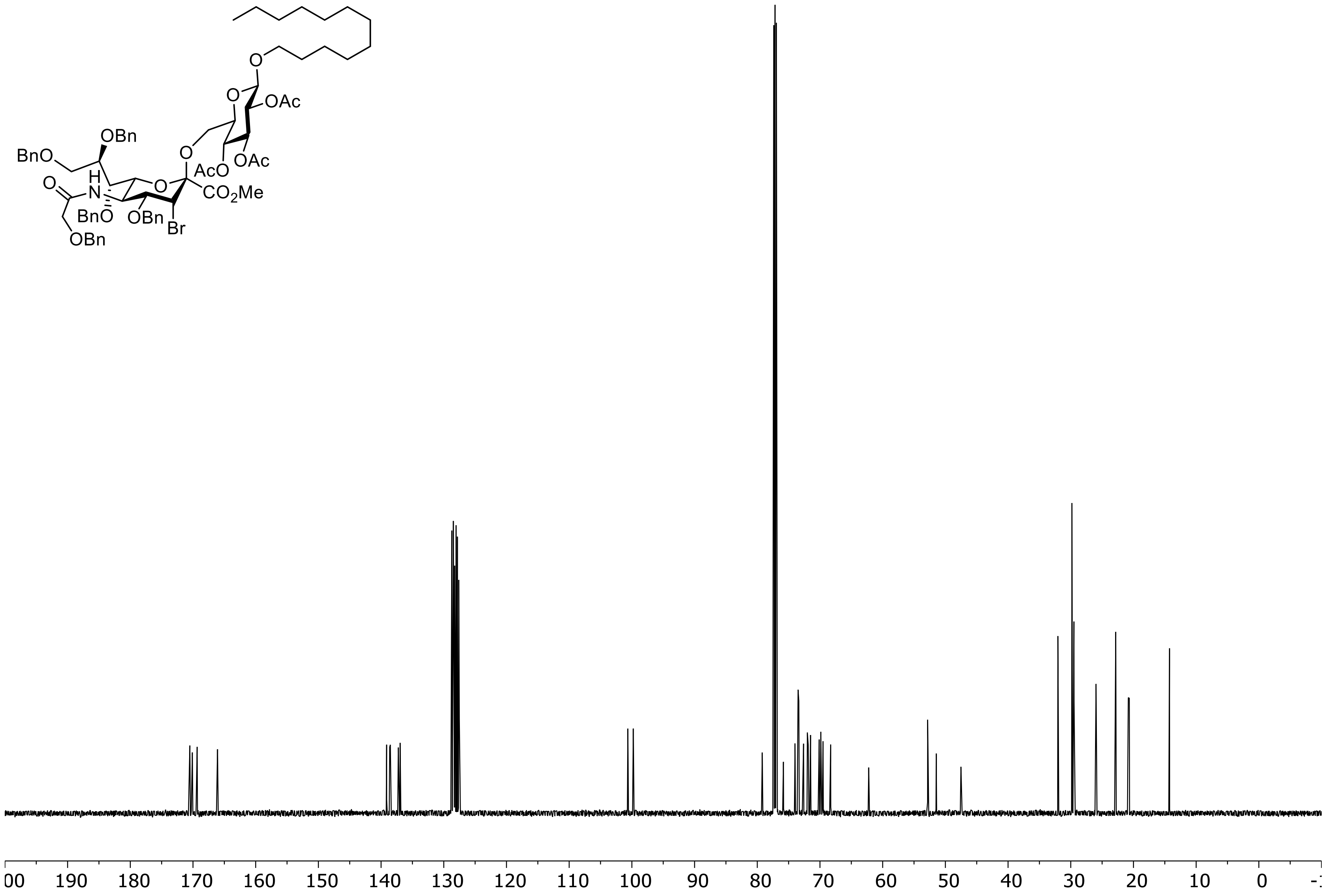

Compound **16** (^{1}H NMR, 500 MHz, $CDCl_3$)

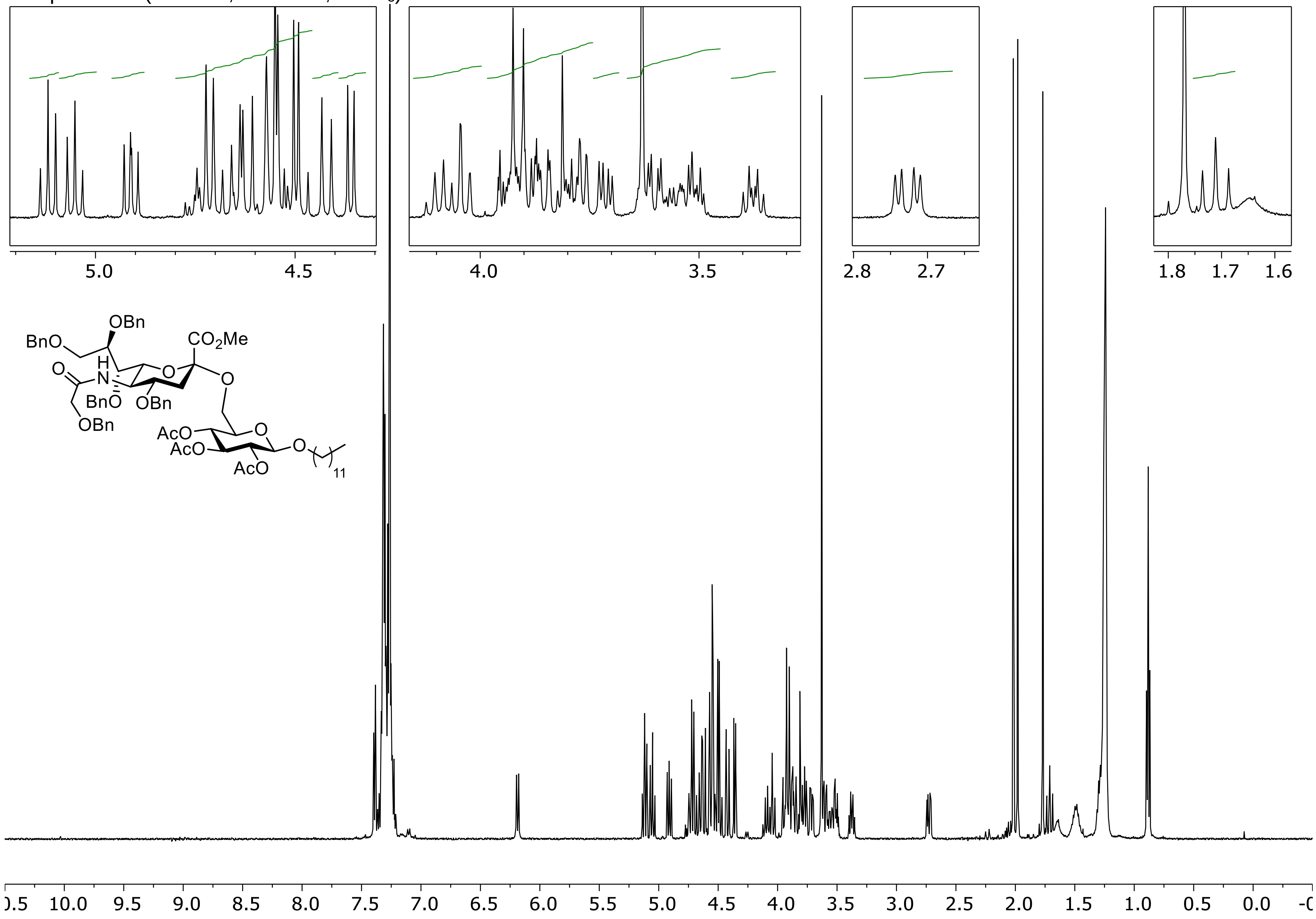

Compound **16** ($^{13}C\{^{1}H\}$ NMR, 126 MHz, $CDCl_3$)

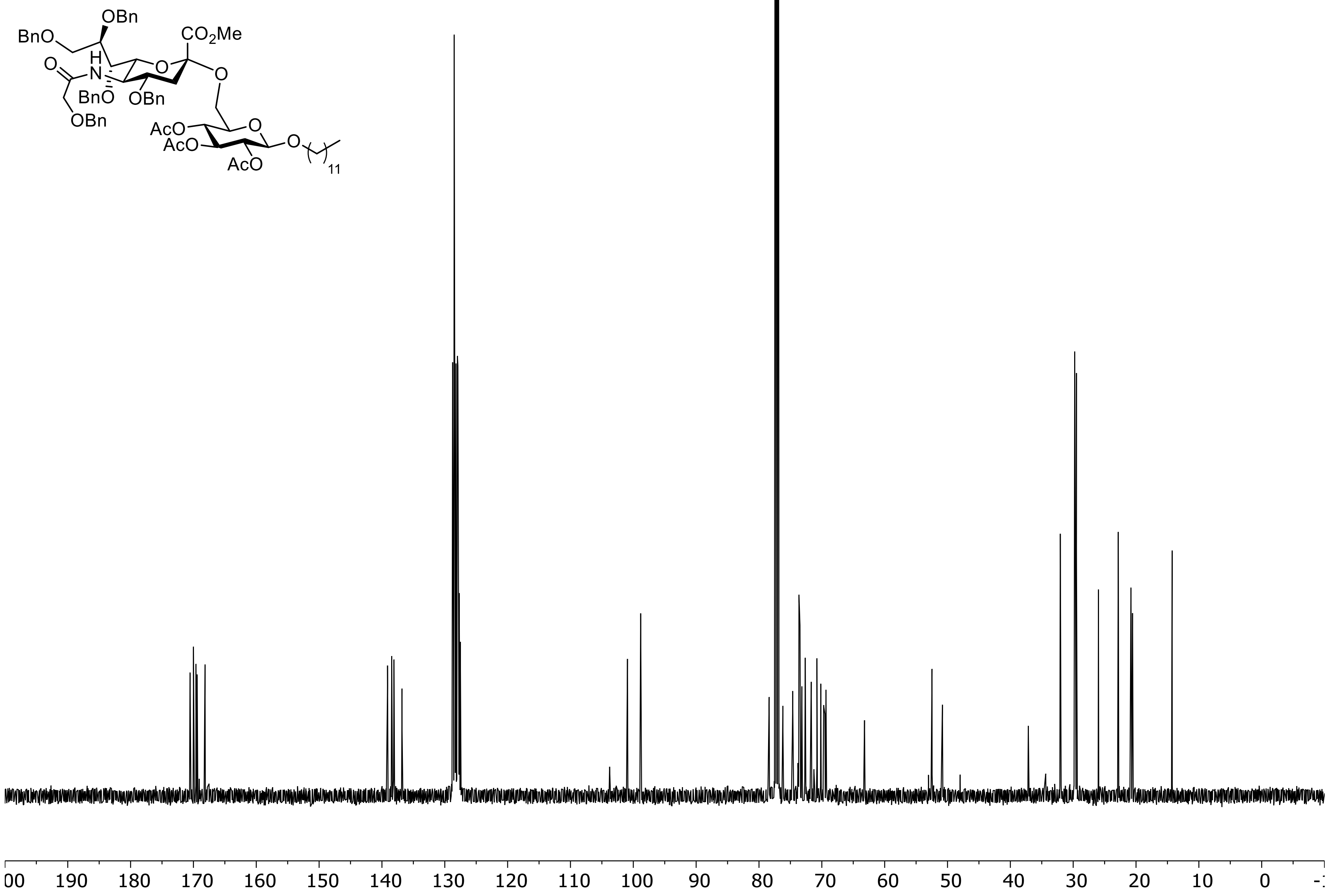

Compound **17** (^{1}H NMR, 599 MHz, $CDCl_3$)

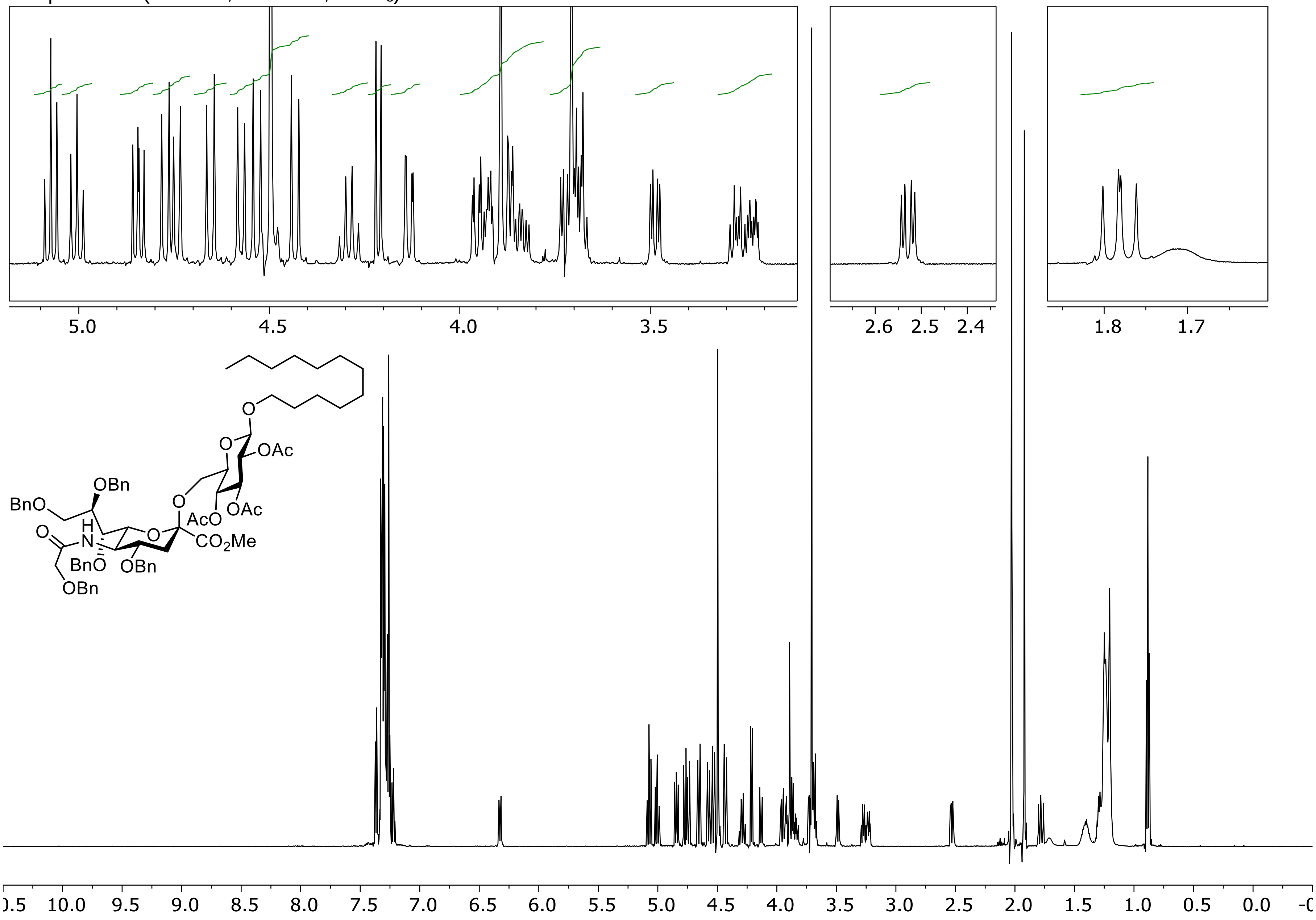

Compound **17** ($^{13}C\{^{1}H\}$ NMR, 151 MHz, $CDCl_3$)

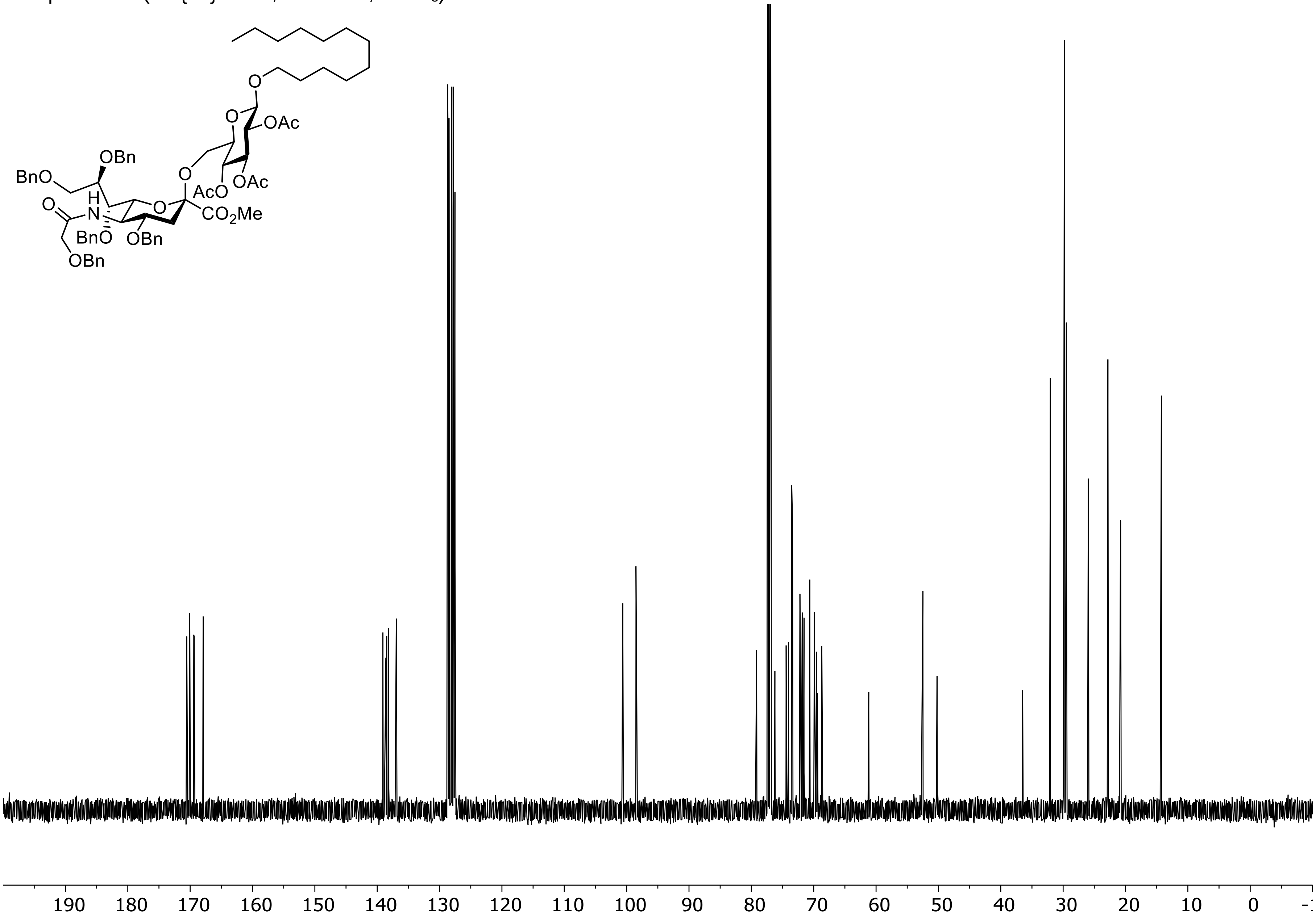

Compound **18** (^{1}H NMR, 599 MHz, CD_3OD)

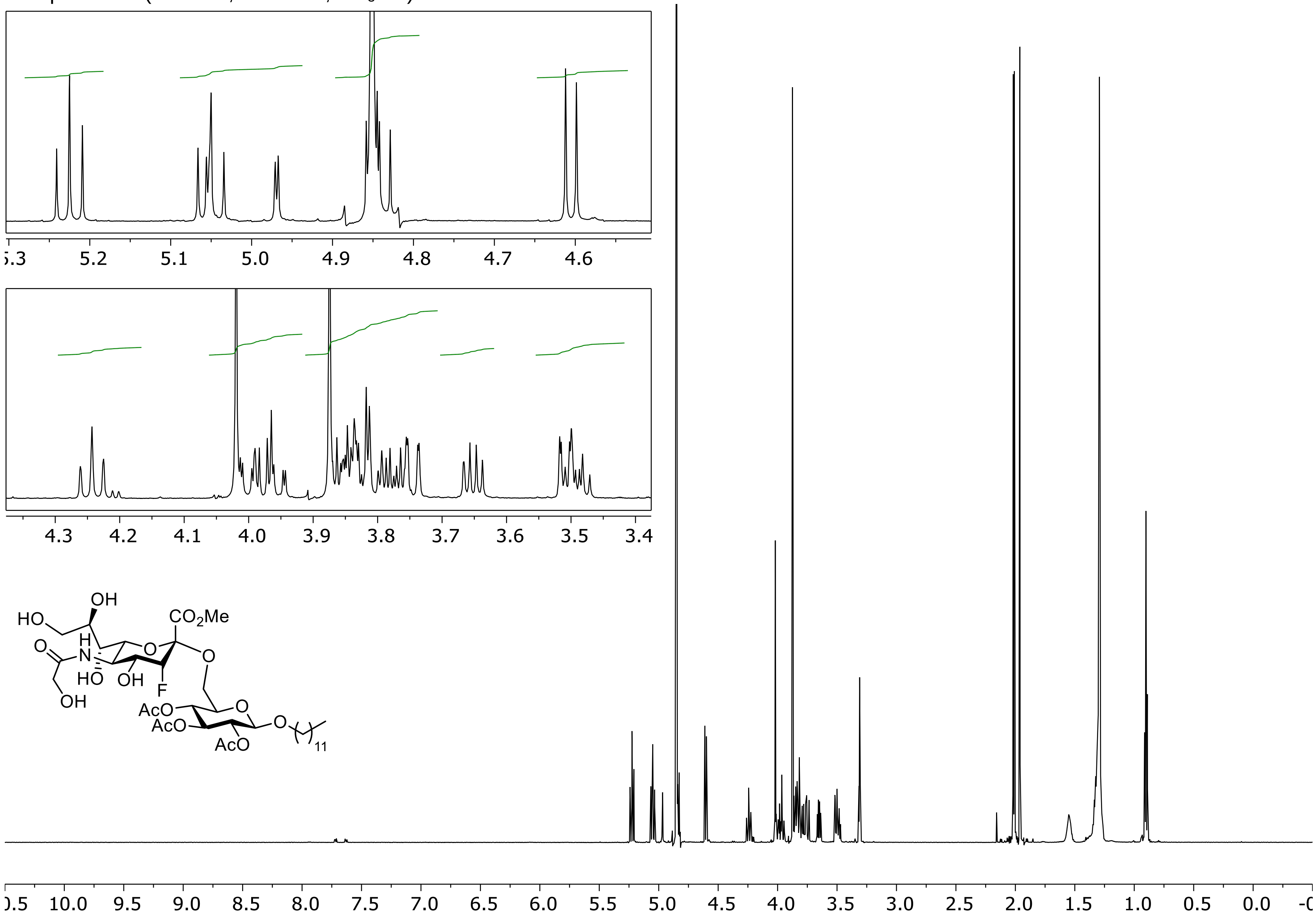

Compound **18** ($^{13}C\{^{1}H\}$ NMR, 151 MHz, CD_3OD)

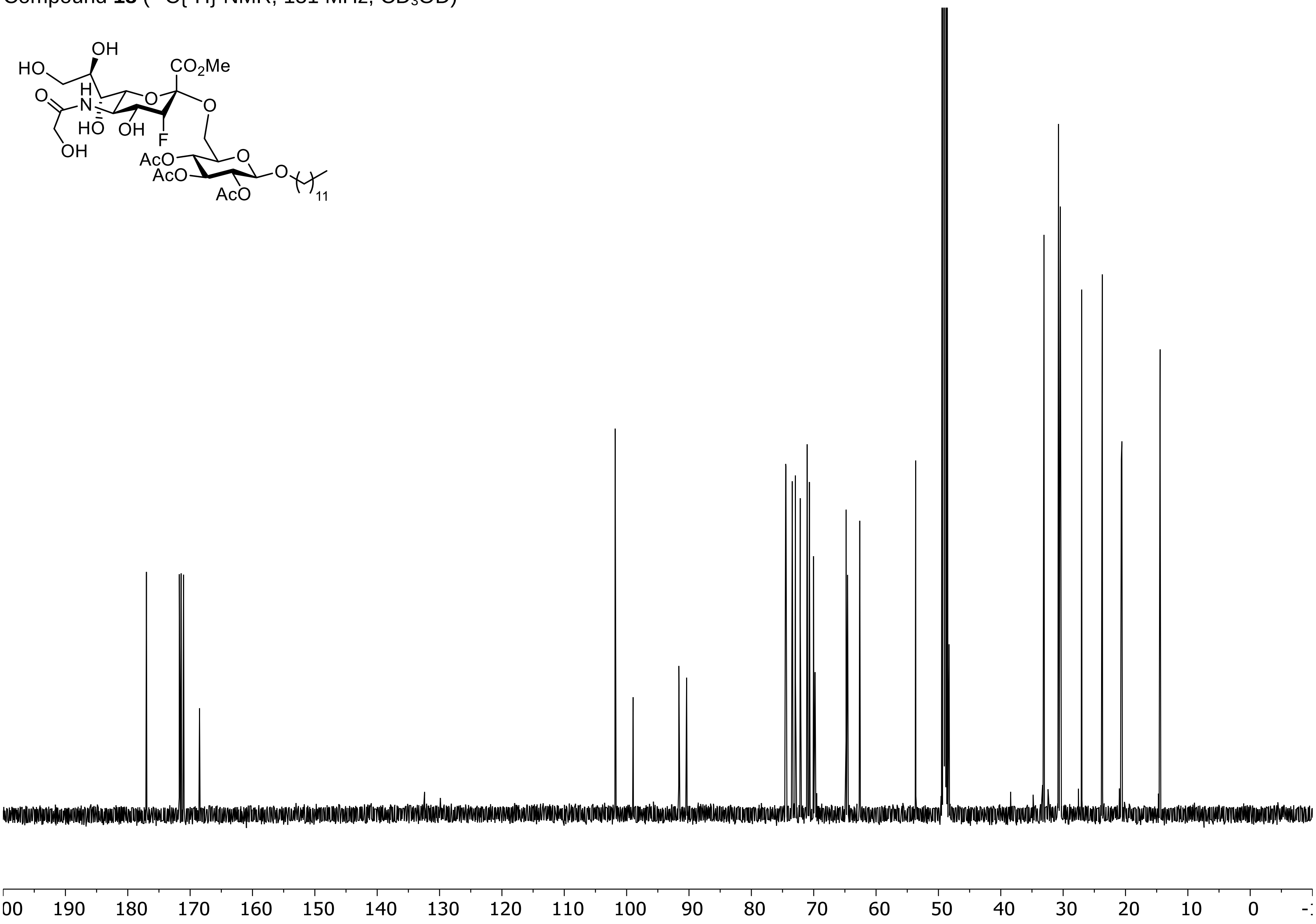

Compound **18** (^{19}F NMR, 564 MHz, CD_3OD)

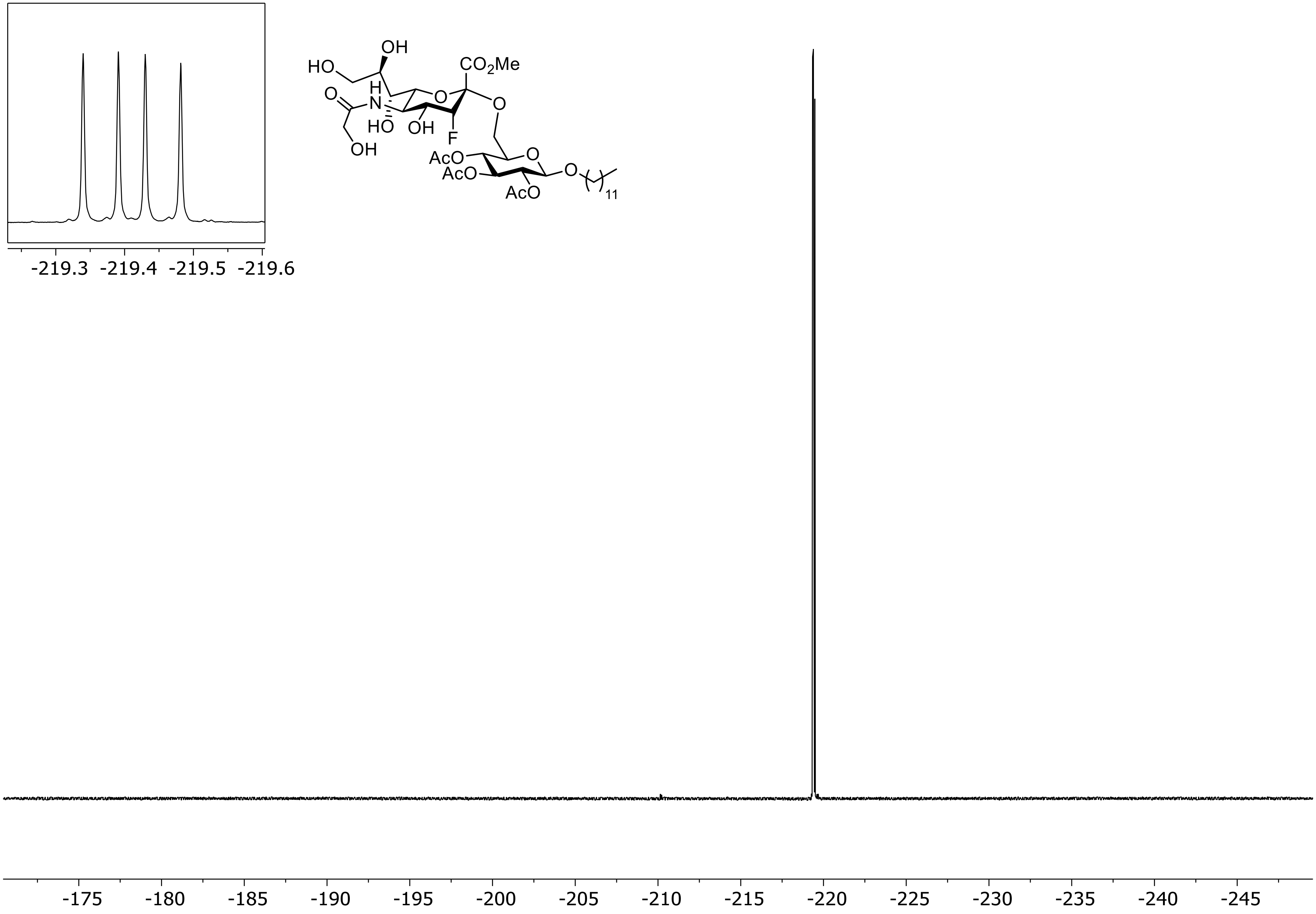

Compound **19** (^{1}H NMR, 599 MHz, CD_3OD)

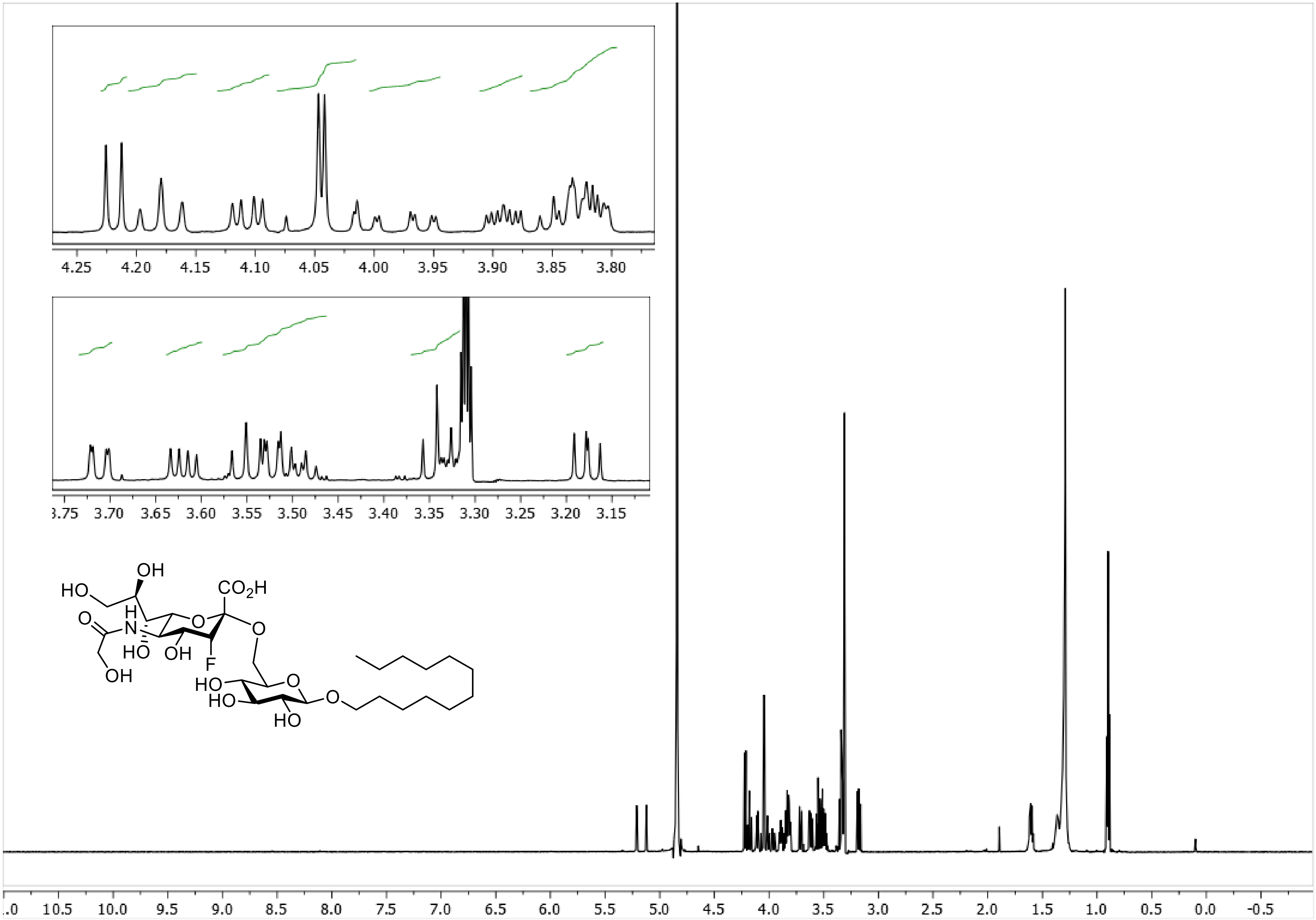

Compound **19** ($^{13}C\{^{1}H\}$ NMR, 151 MHz, CD_3OD)

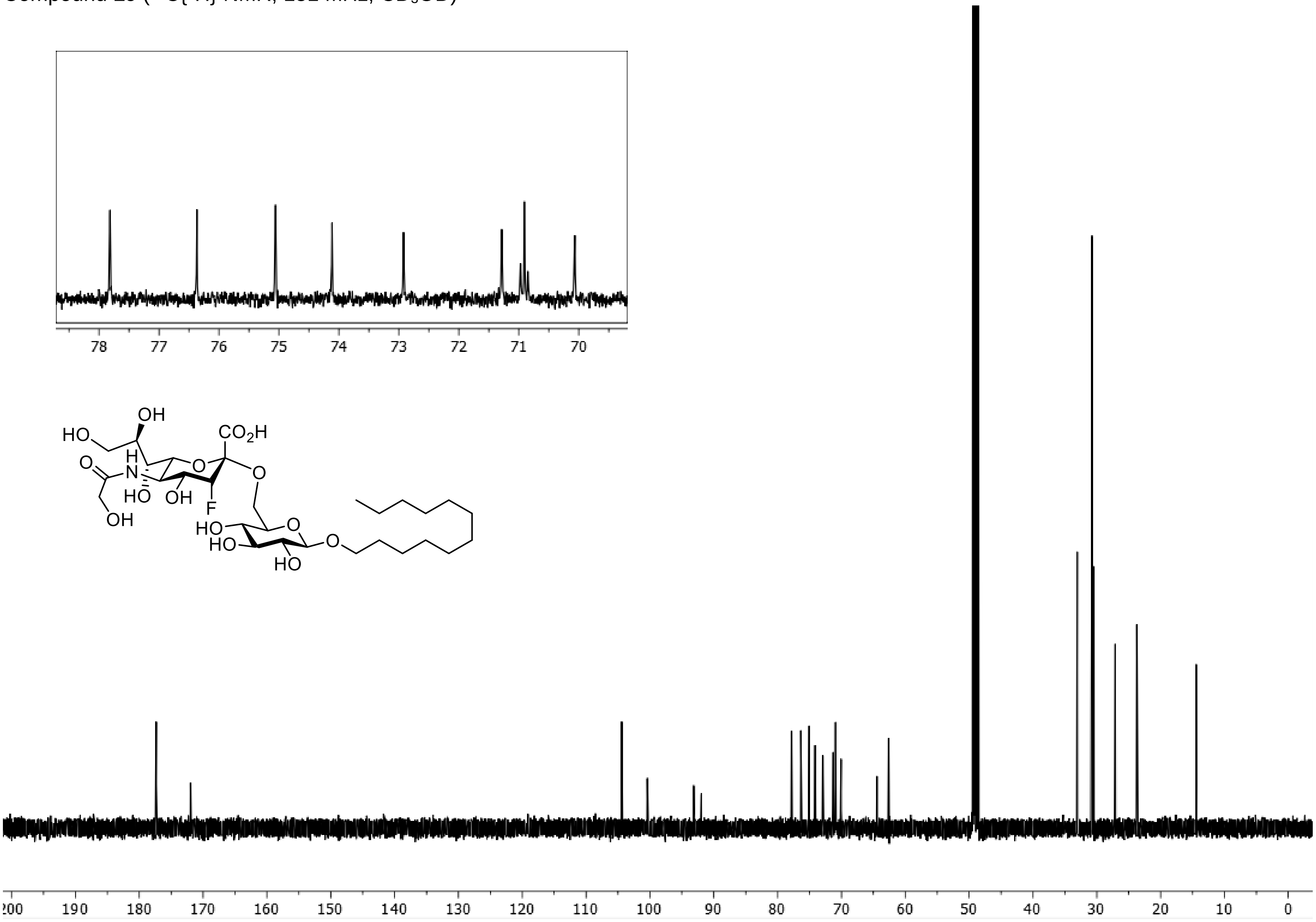

Compound **19** (^{19}F NMR, 564 MHz, CD_3OD)

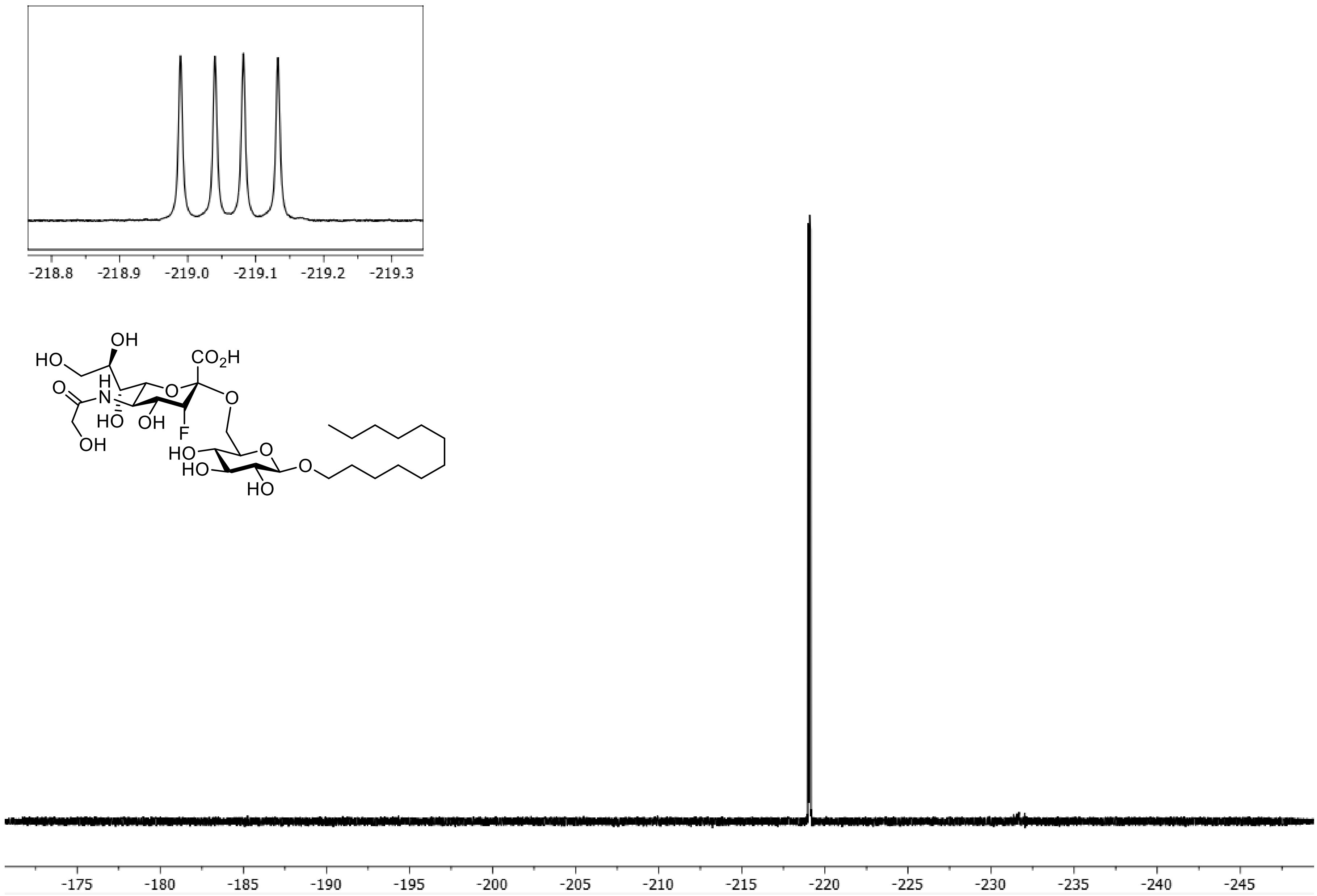

Compound **21** (^{1}H NMR, 599 MHz, CD_3OD)

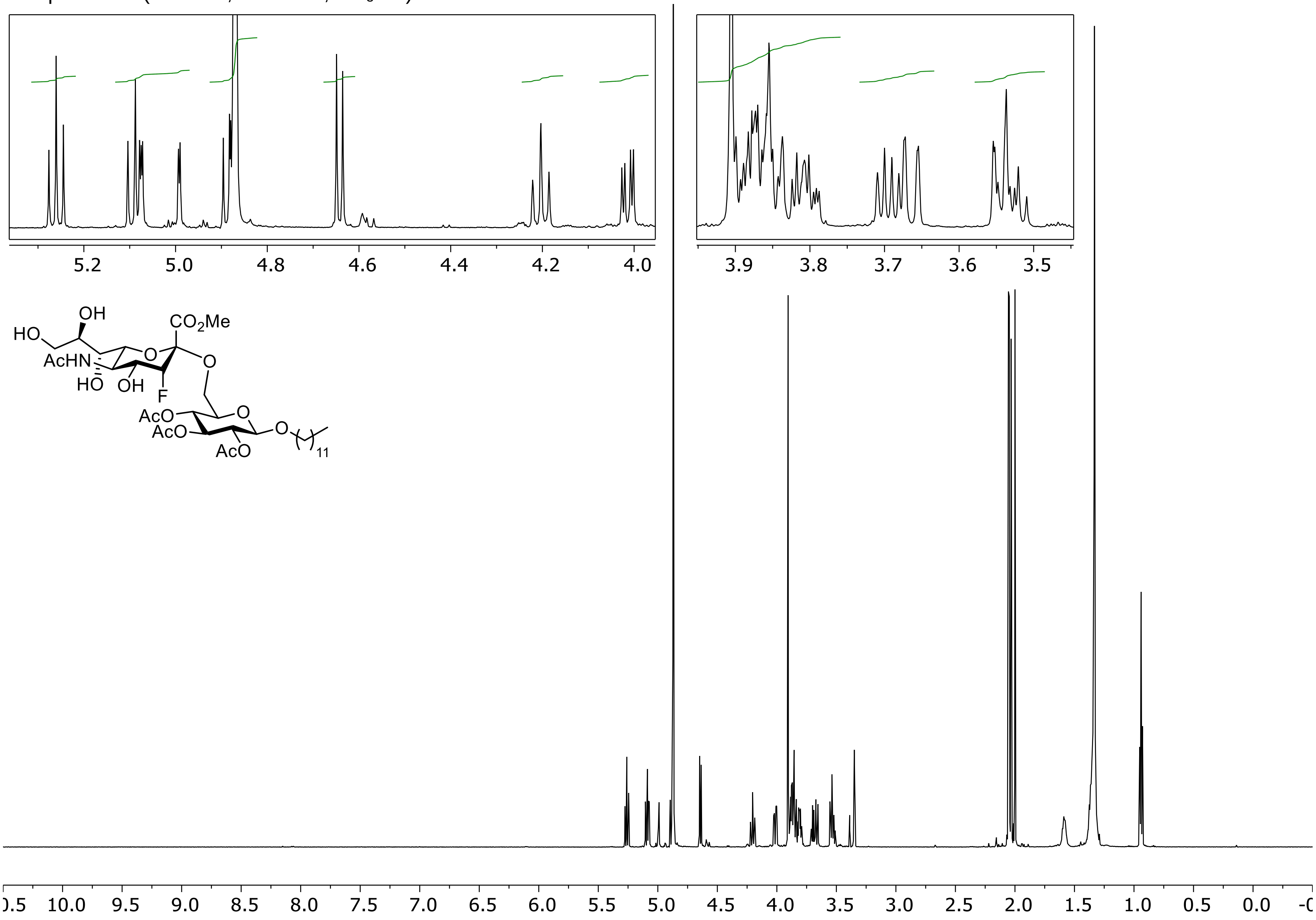

Compound **21** ($^{13}C\{^1H\}$ NMR, 151 MHz, CD_3OD)

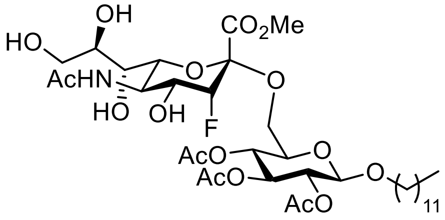

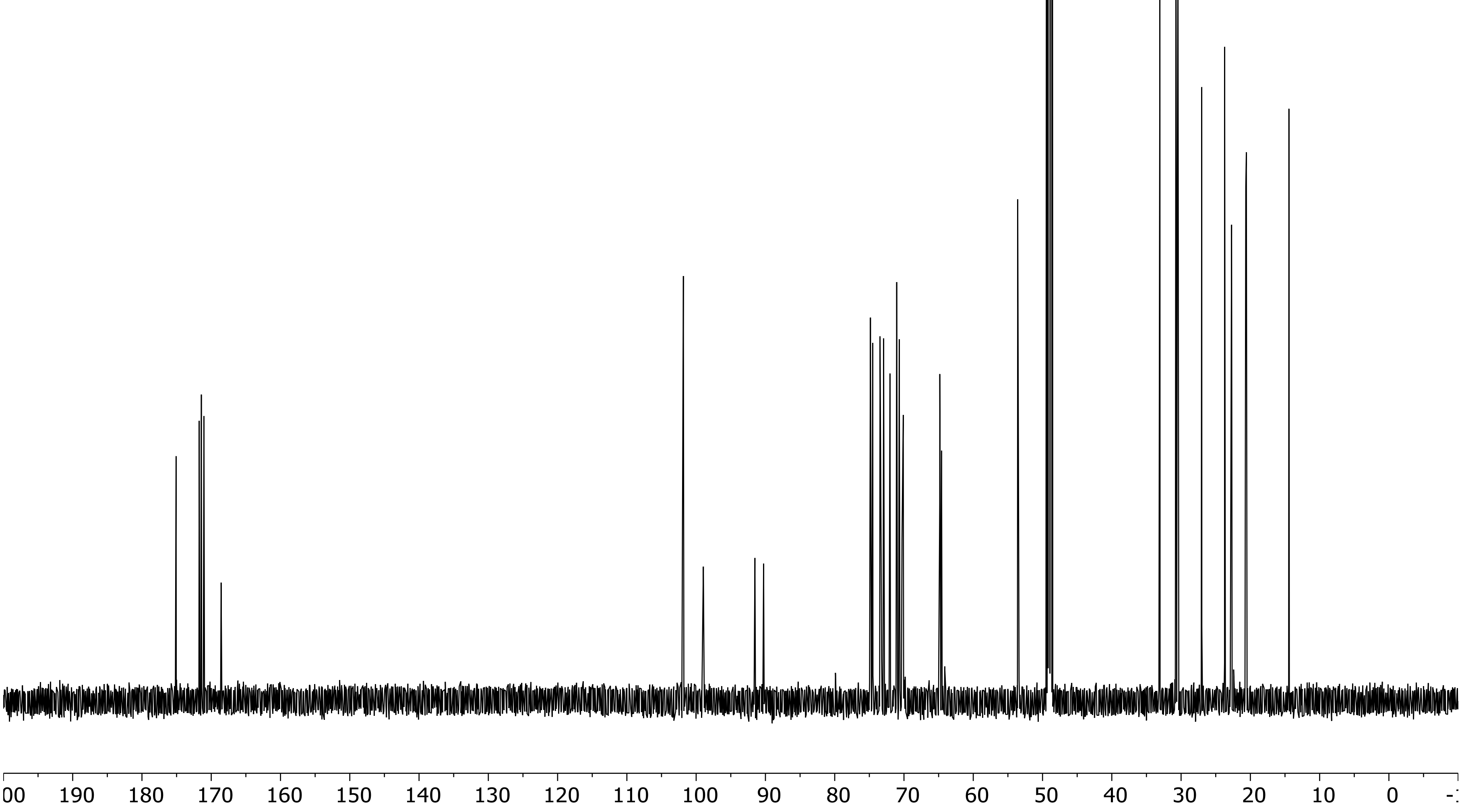

Compound **21** (^{19}F NMR, 564 MHz, CD_3OD)

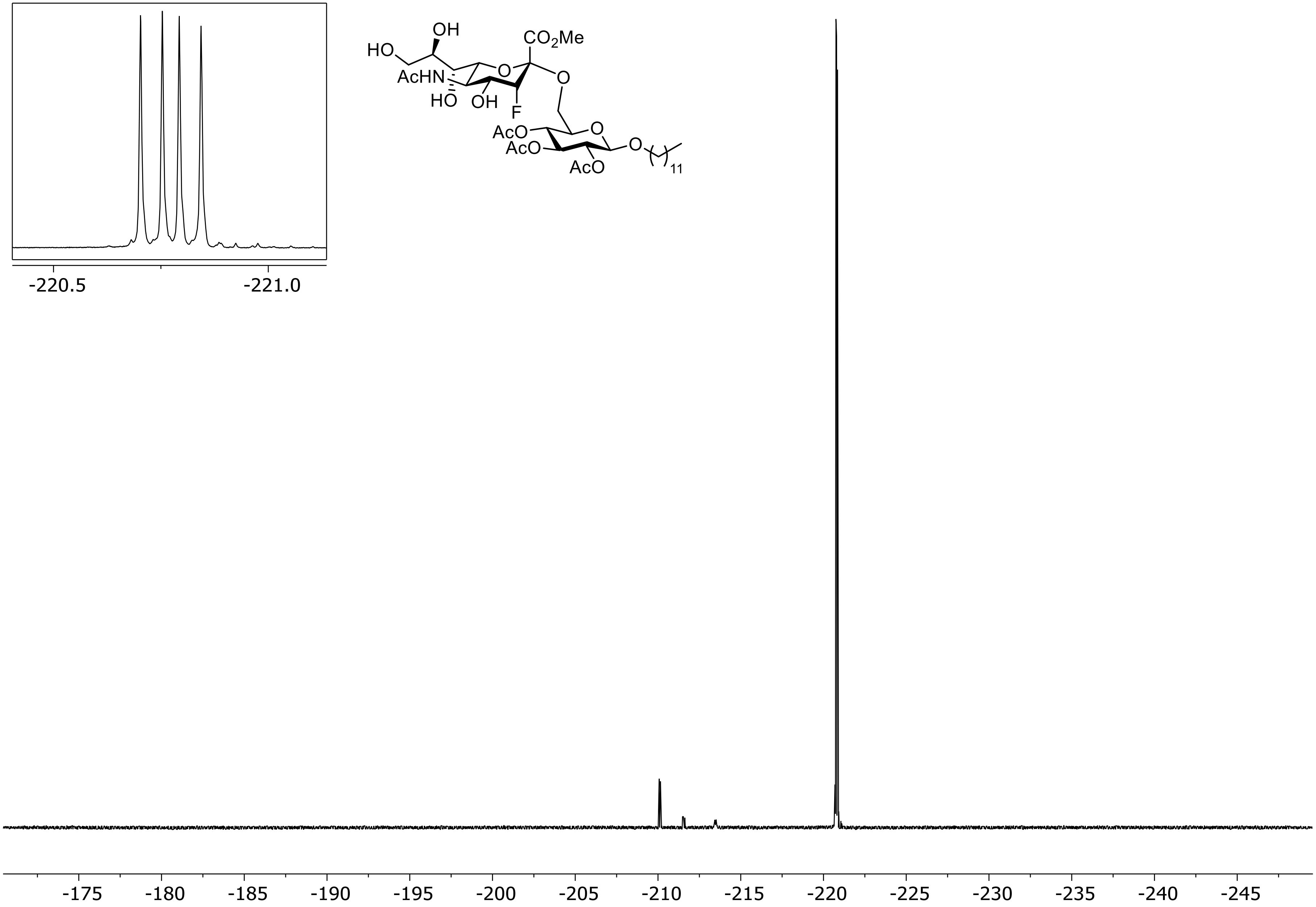

Compound **22** (^{1}H NMR, 599 MHz, CD_3OD)

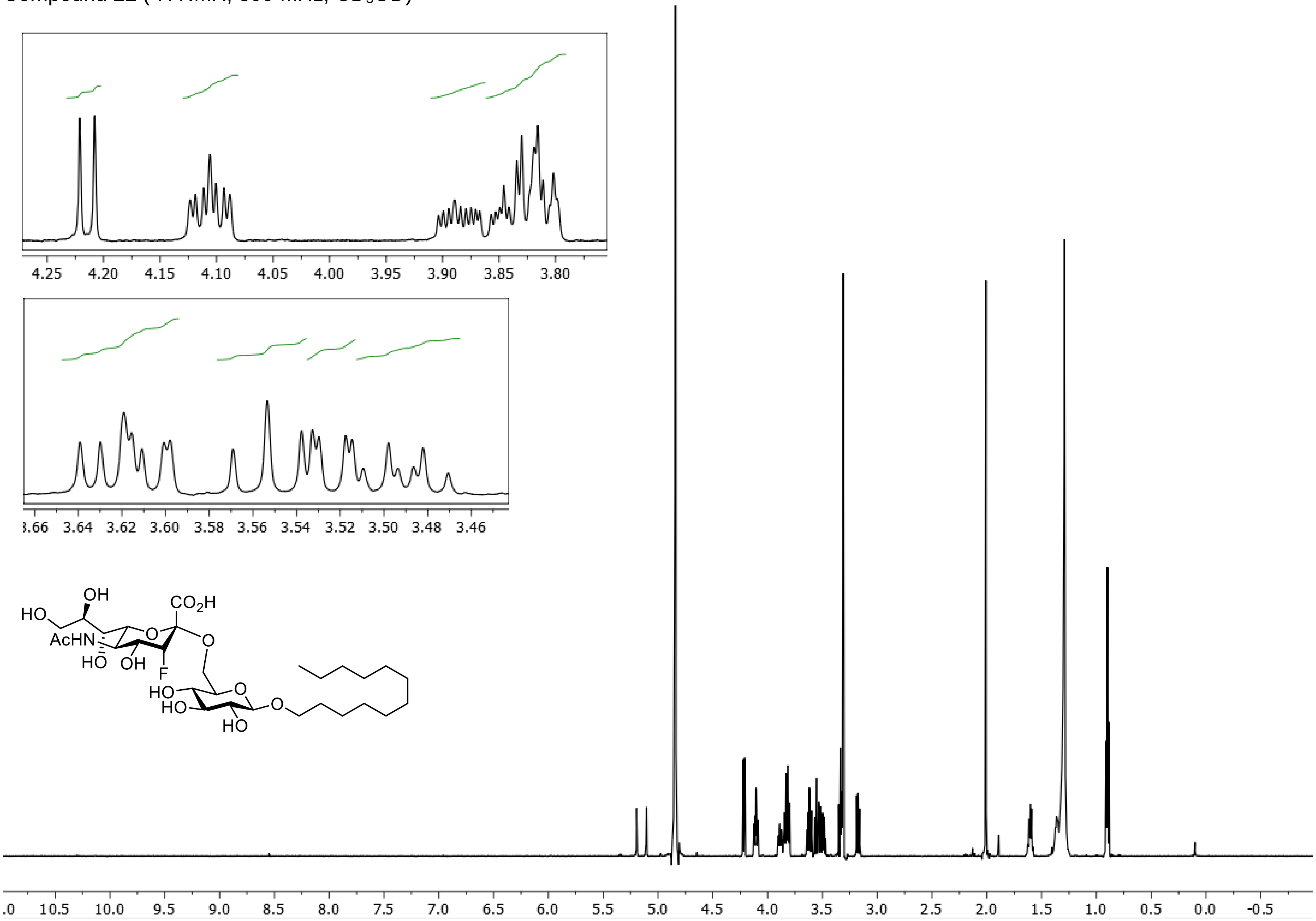

Compound **22** ($^{13}C\{^{1}H\}$ NMR, 151 MHz, CD_3OD)

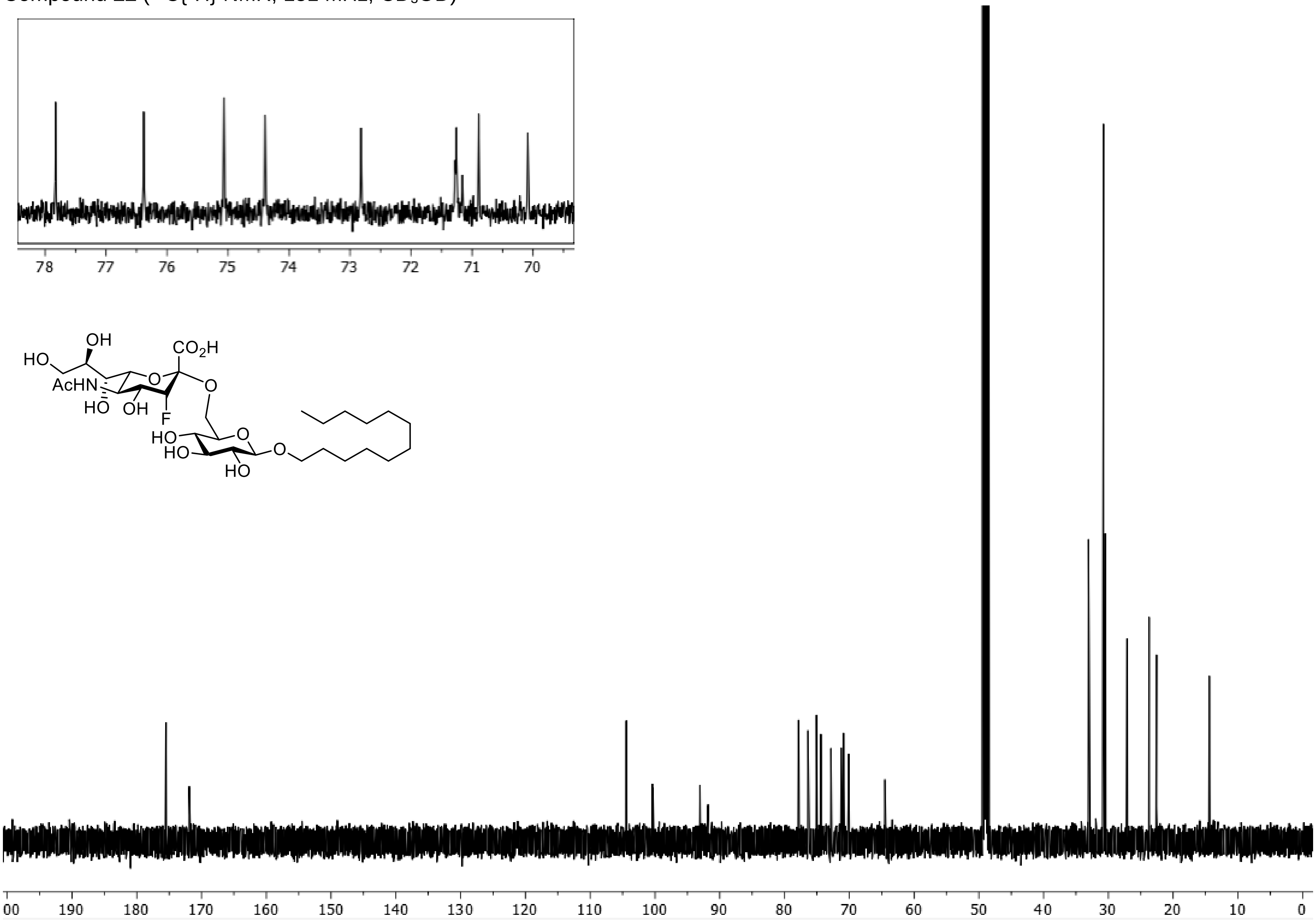

Compound **22** (^{19}F NMR, 564 MHz, CD_3OD)

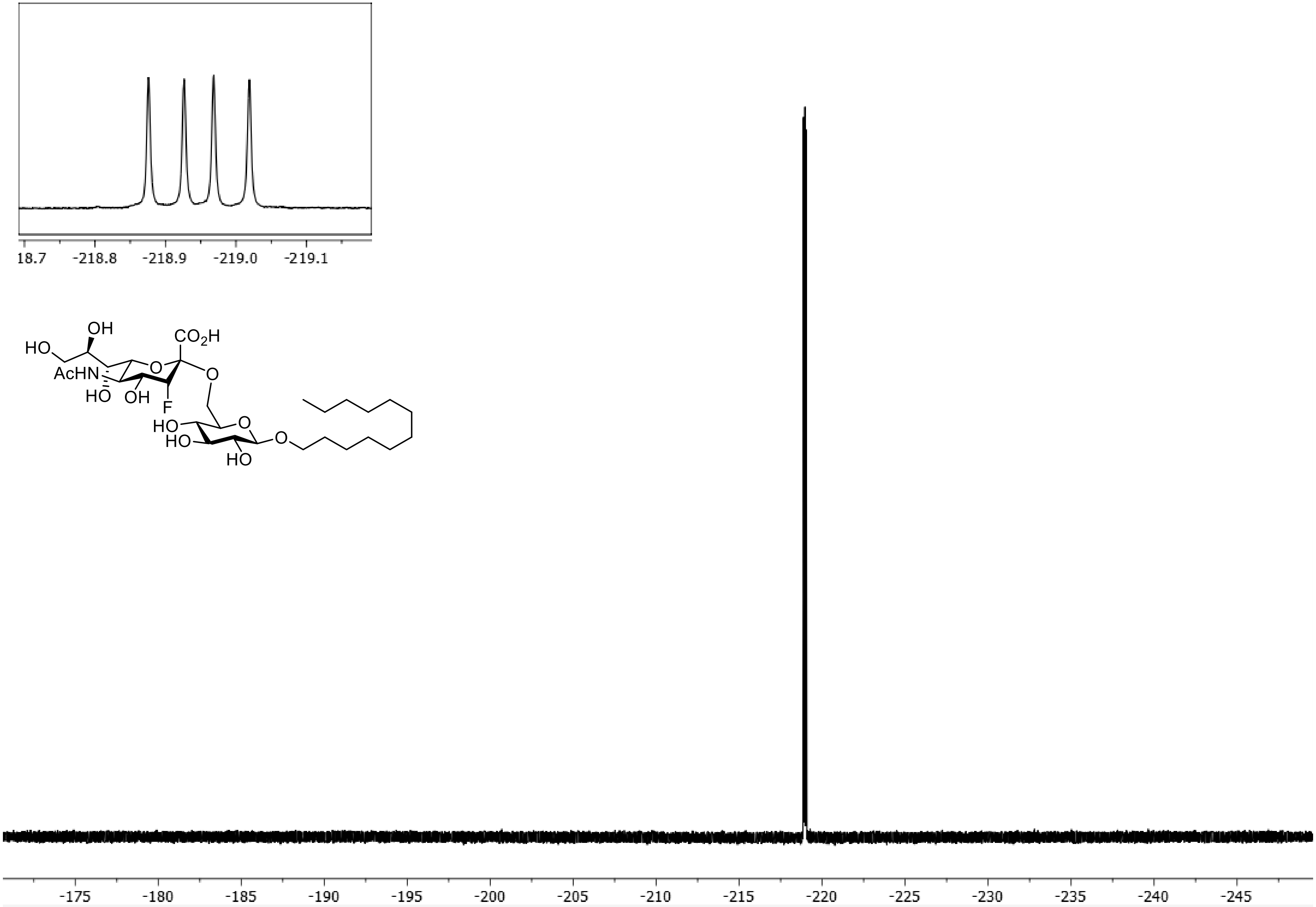

Compound **23** (^{1}H NMR, 599 MHz, CD_2Cl_2)

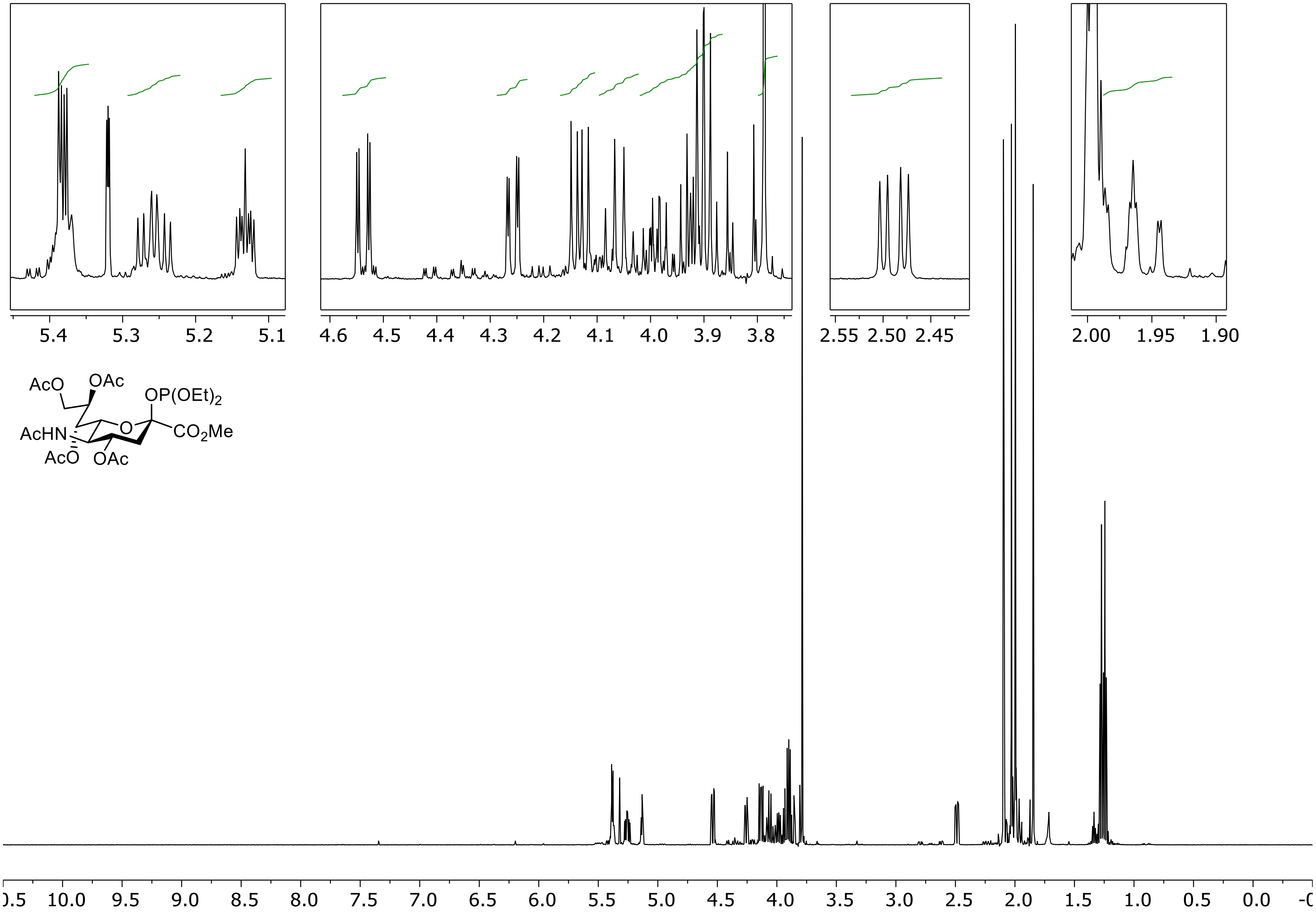

Compound **23** ($^{13}C\{^{1}H\}$ NMR, 151 MHz, CD_2Cl_2)

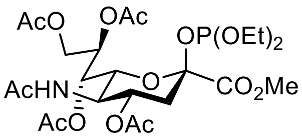

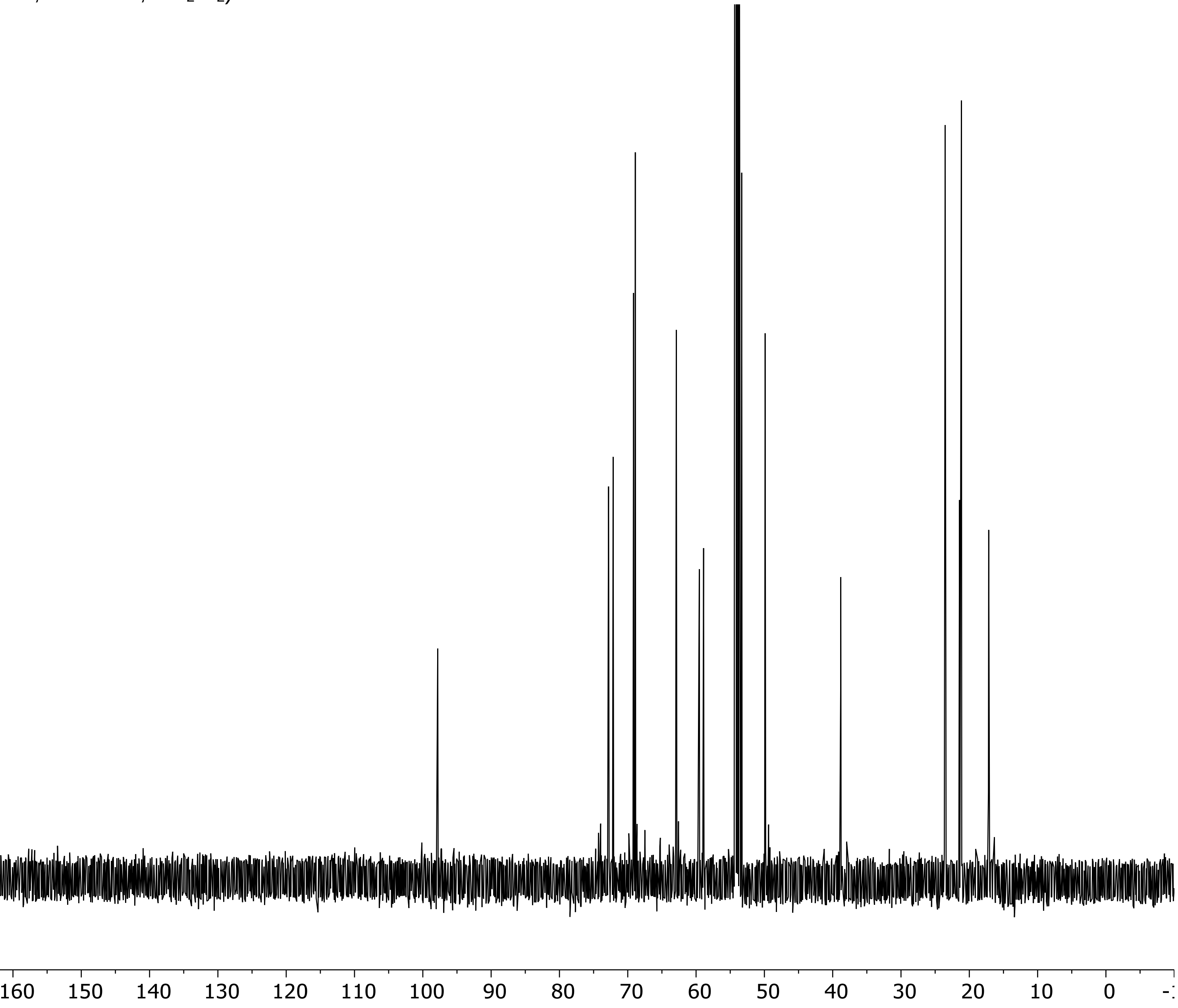

Compound **23** (^{31}P NMR, 243 MHz, CD_2Cl_2)

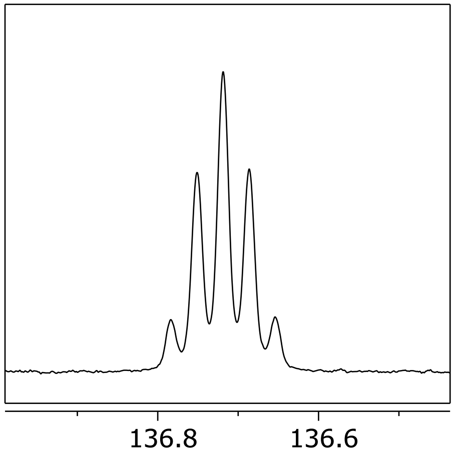

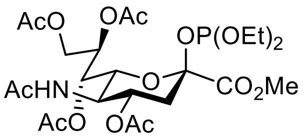

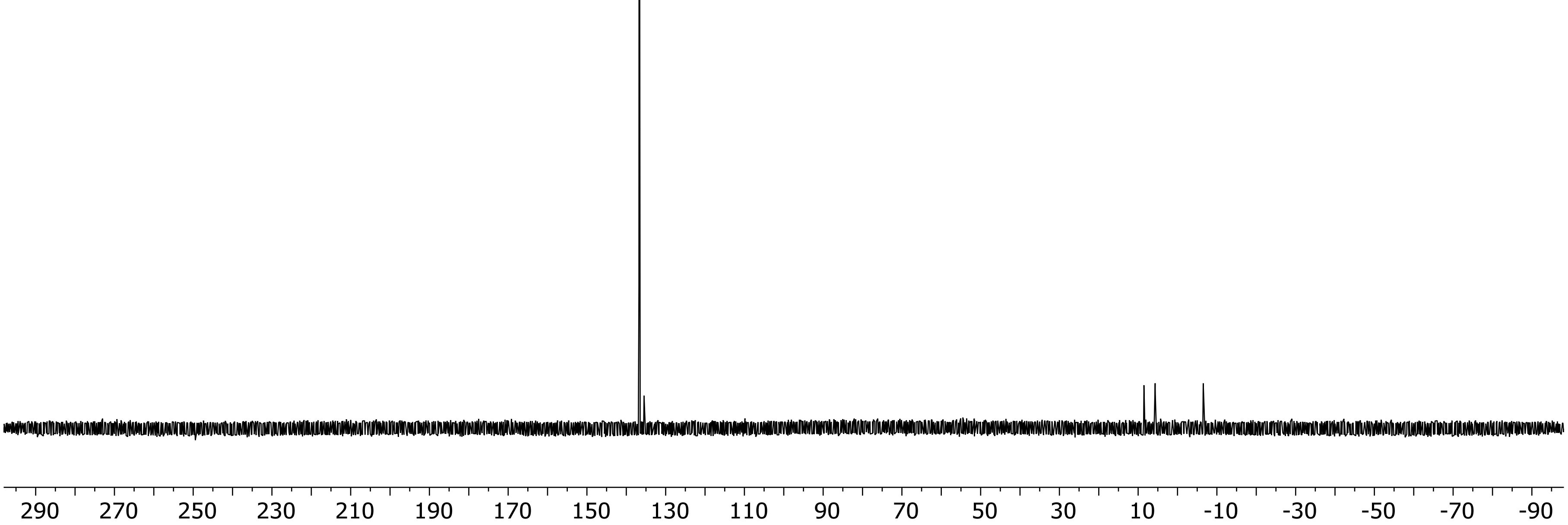

Compound **25** (^{1}H NMR, 599 MHz, $CDCl_3$)

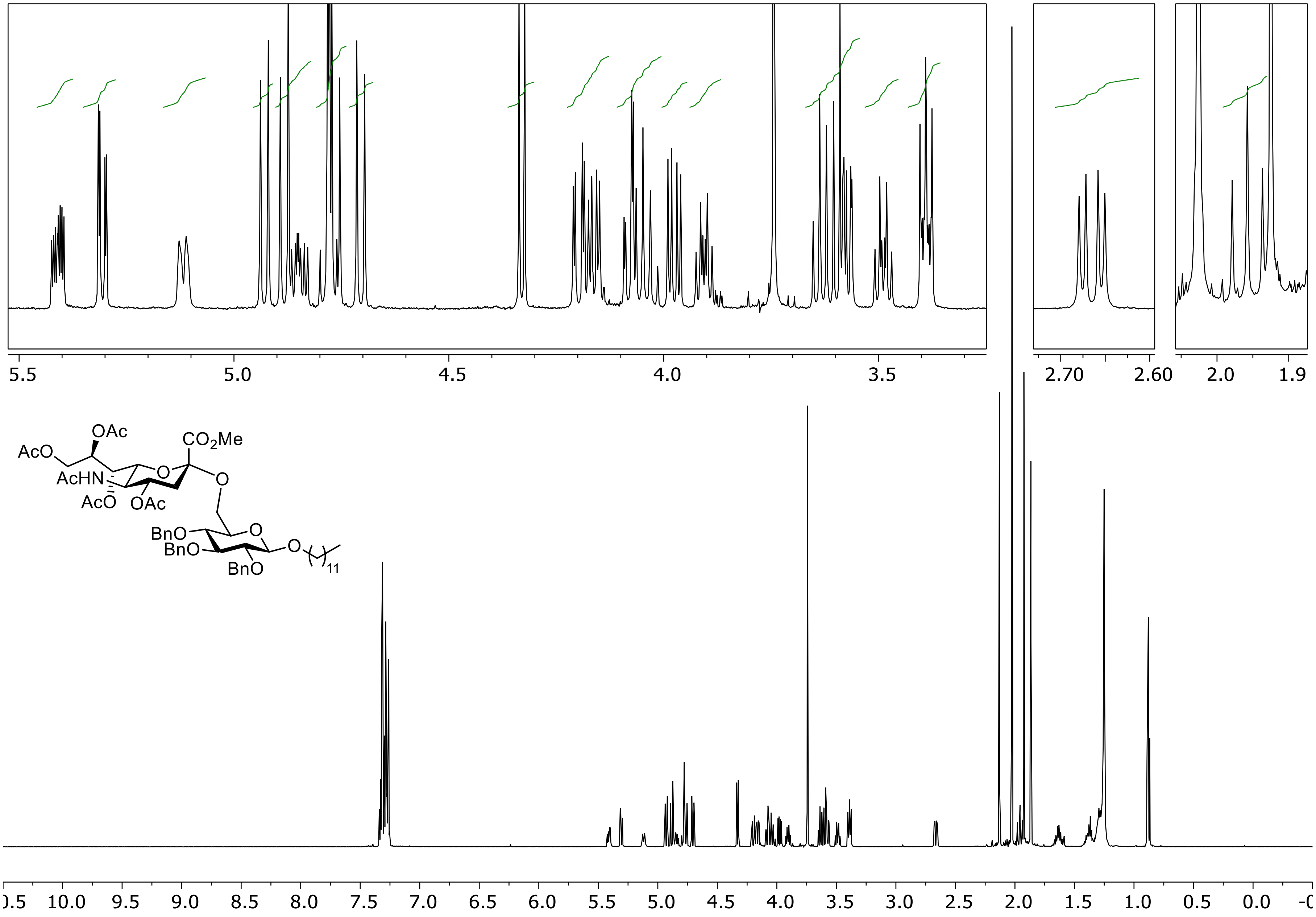

Compound **25** ($^{13}C\{^{1}H\}$ NMR, 151 MHz, $CDCl_3$)

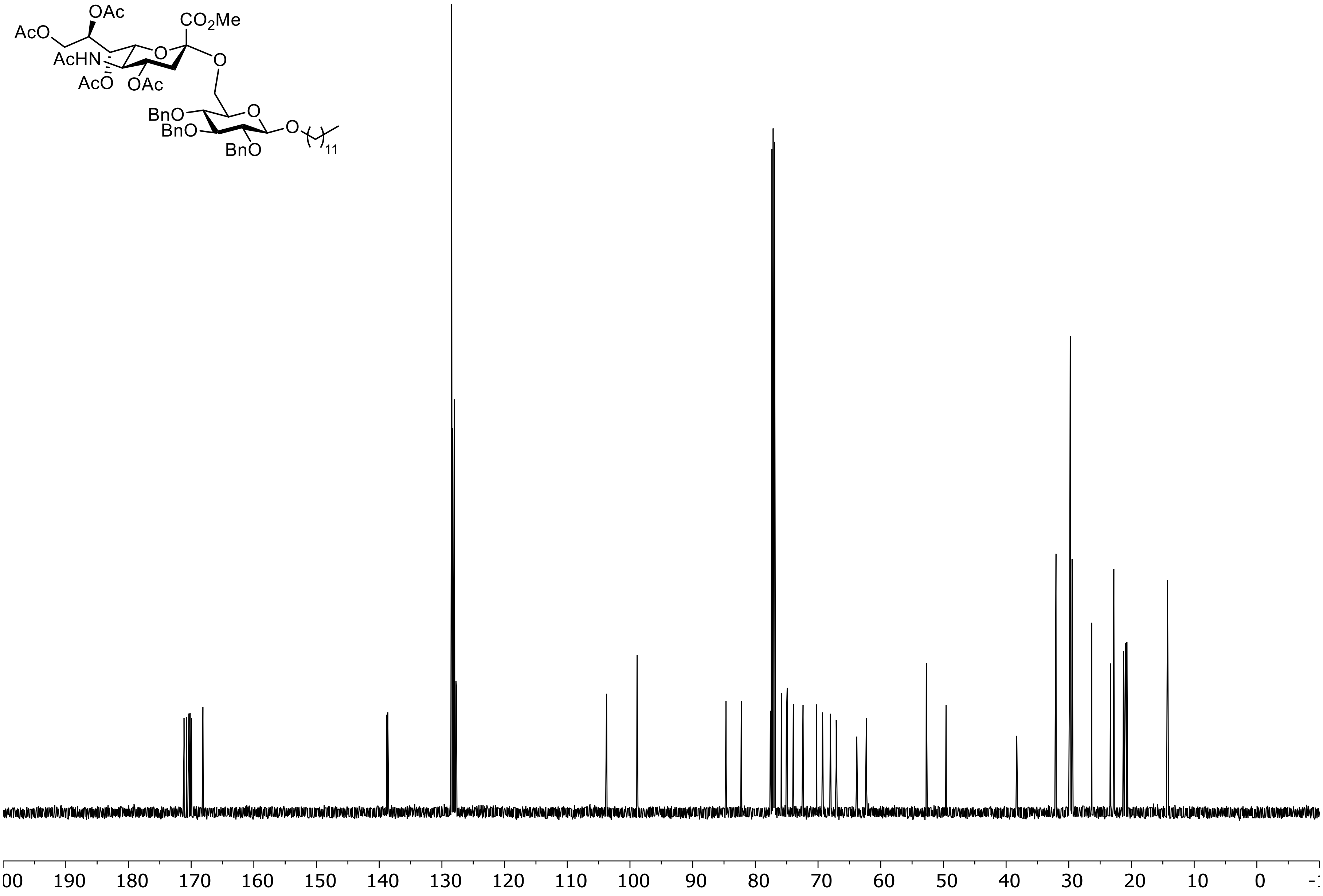

Compound **26** (^1H NMR, 599 MHz, $CDCl_3$)

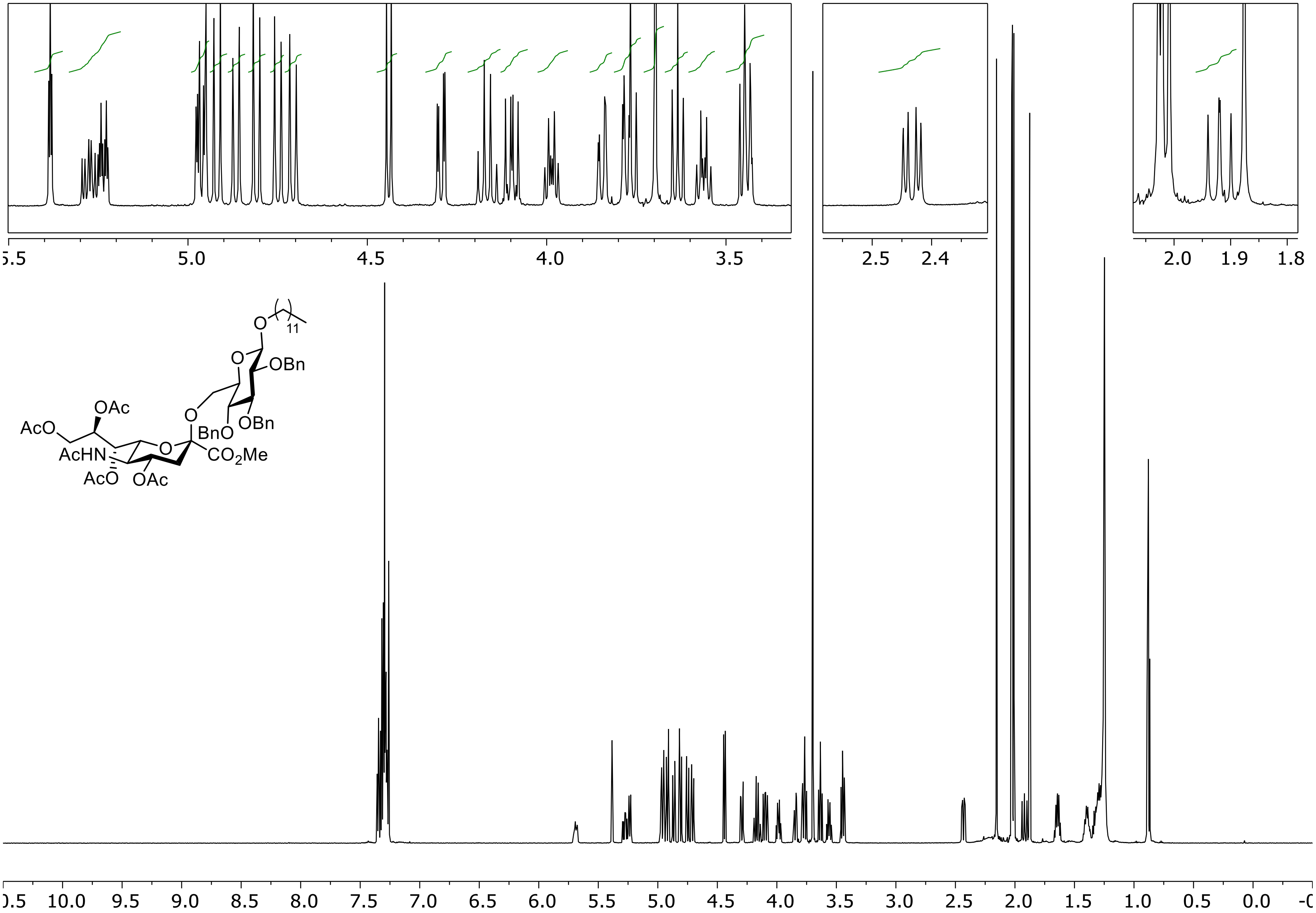

Compound **26** ($^{13}C\{^{1}H\}$ NMR, 151 MHz, $CDCl_3$)

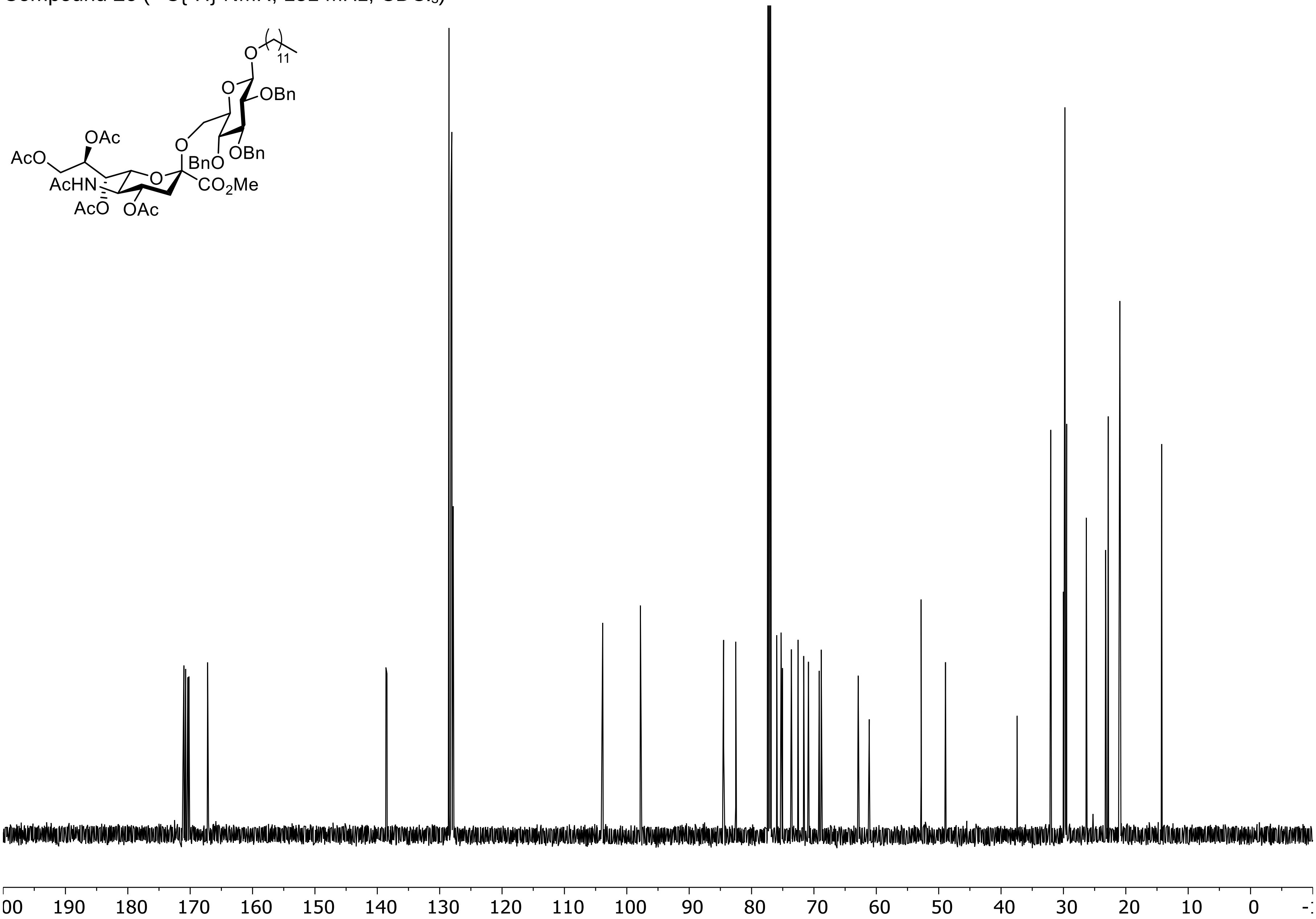

Compound **27** (^{1}H NMR, 599 MHz, CD_3OD)

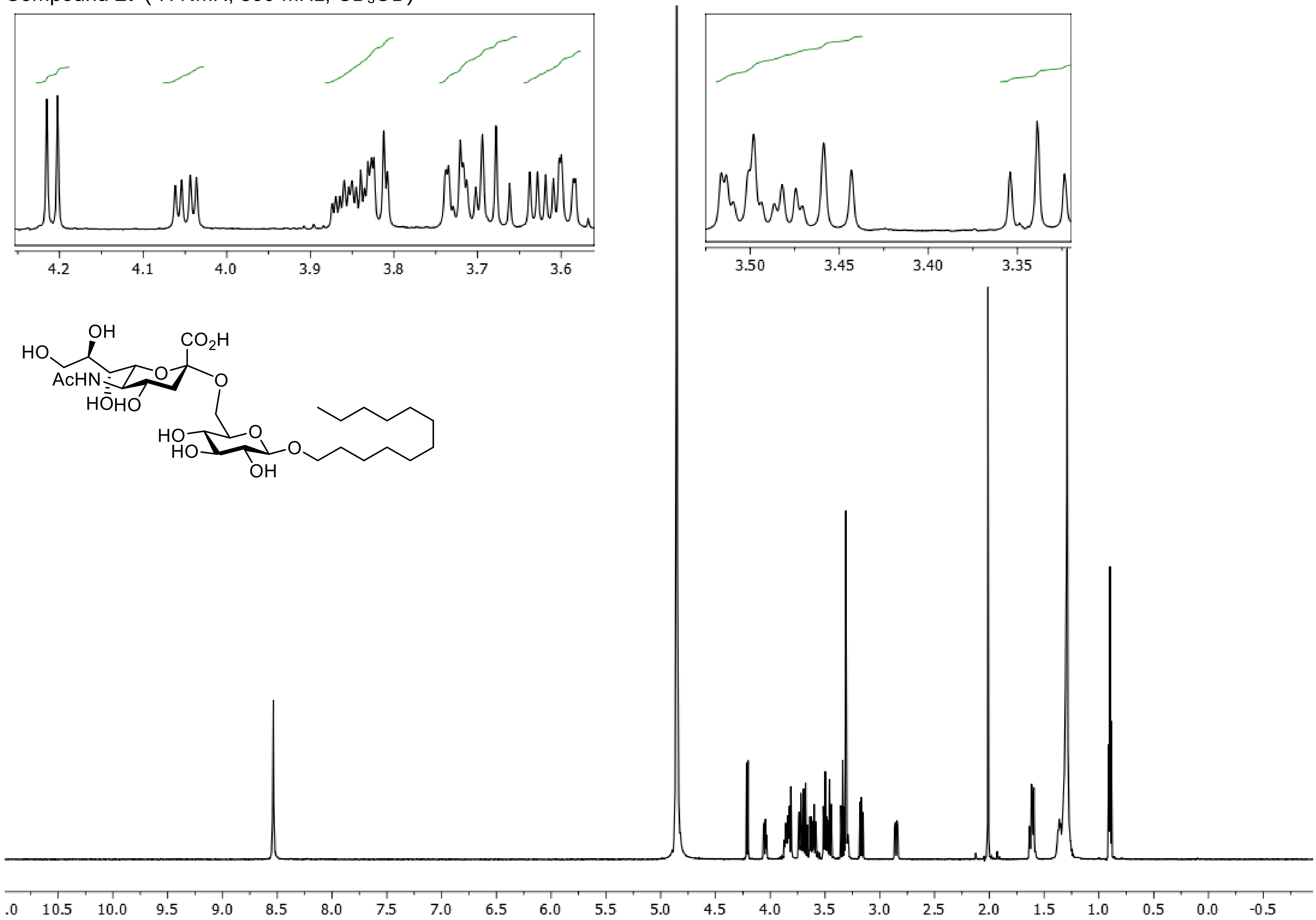

Compound **27** ($^{13}C\{^1H\}$ NMR, 151 MHz, CD_3OD)

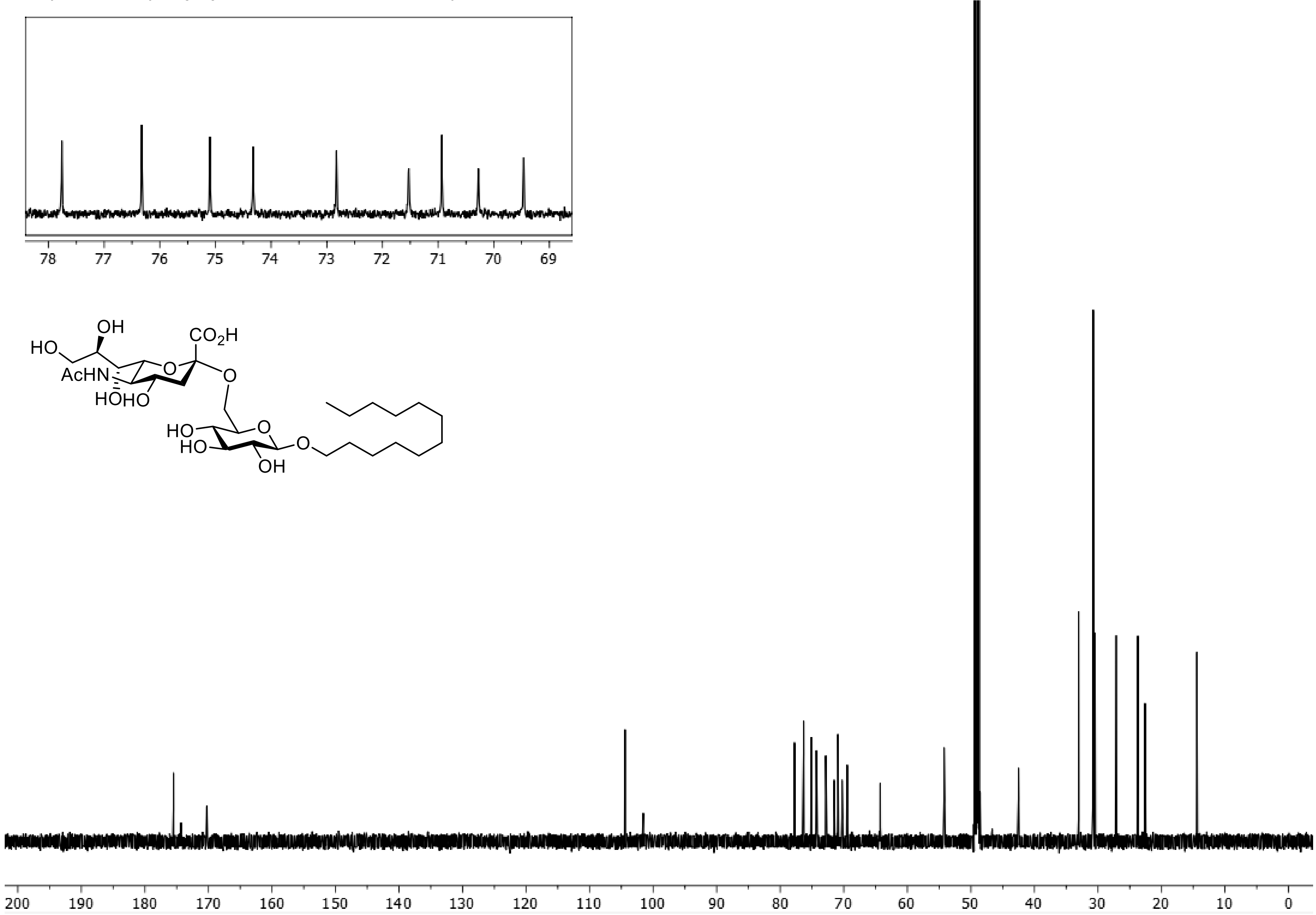

Compound **28** (^{1}H NMR, 599 MHz, CD_3OD)

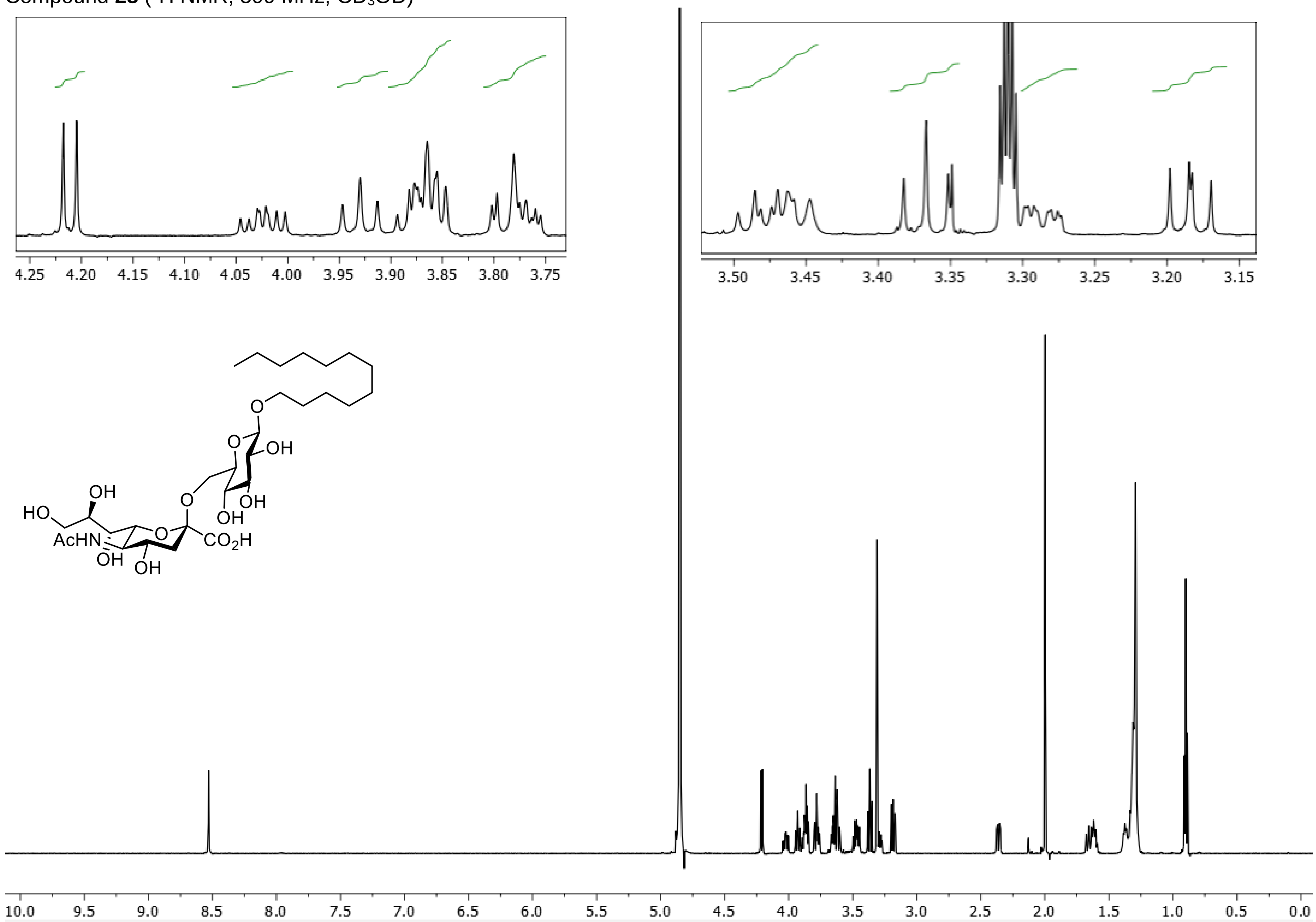

Compound **28** ($^{13}C\{^{1}H\}$ NMR, 151 MHz, CD_3OD)

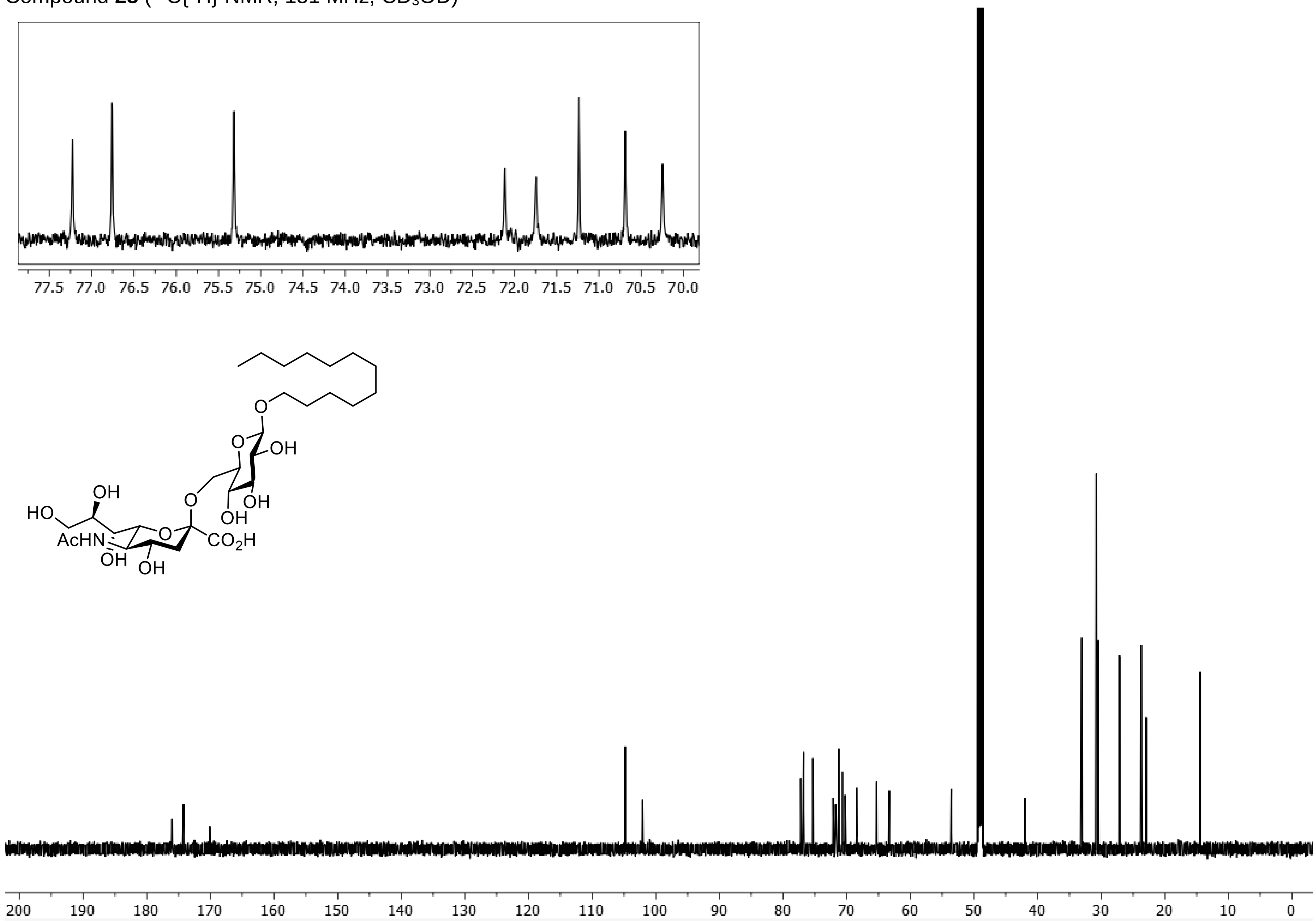

Supplement: SC-011-D0SC01219J-s001 [file SC-011-D0SC01219J-s001.pdf]
